# Direct Synthesis of α‐Amino Acid Derivatives by Hydrative Amination of Alkynes

**DOI:** 10.1002/anie.202212399

**Published:** 2022-11-29

**Authors:** Minghao Feng, Roberto Tinelli, Ricardo Meyrelles, Leticia González, Boris Maryasin, Nuno Maulide

**Affiliations:** ^1^ Institute of Organic Chemistry University of Vienna Währinger Strasse 38 1090 Vienna Austria; ^2^ Institute of Theoretical Chemistry University of Vienna Währinger Strasse 17 1090 Vienna Austria; ^3^ Vienna Doctoral School in Chemistry University of Vienna Währinger Strasse 42 1090 Vienna Austria

**Keywords:** Amination, Amino Acids, Reaction Mechanisms, Rearrangements, Ynamides

## Abstract

α‐Amino acid derivatives are key components of the molecules of life. The synthesis of α‐amino carbonyl/carboxyl compounds is a contemporary challenge in organic synthesis. Herein, we report a practical method for the preparation of α‐amino acid derivatives via direct hydrative amination of activated alkynes under mild conditions, relying on sulfinamides as the nitrogen source. Computational studies suggest that the reaction is enabled by a new type of sulfonium [2,3]‐sigmatropic rearrangement.

## Graphical abstract

α‐Amino acid derivatives are key components of the molecules of life. A hydrative amination of ynamides/thioalkynes under metal‐free and mild conditions has been developed. This practical strategy offers a new and convenient avenue for the synthesis of α‐amino acid derivatives using readily available sulfinamides as nitrogen sources. Computational studies suggest that the reaction is enabled by a new type of sulfonium [2,3]‐sigmatropic rearrangement.

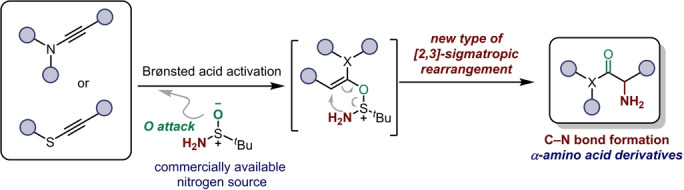

α‐Amino acid derivatives are key building blocks in nature and widely represented among pharmaceutically active compounds and complex natural products.^[1]^ Owing to their key role in modulating the properties of peptides, a variety of approaches toward the synthesis of non‐natural α‐amino carbonyl derivatives have been developed.[^2^, ^3^] The hydrative functionalisation of alkynes has emerged as an efficient strategy for the preparation of α‐functionalised carbonyl compounds.^[4]^ A range of methods relying on Brønsted acid[^5^, ^6^] or soft transition‐metal catalysis,^[7]^ leading to different α‐substituted carbonyl products, have been reported. However, syntheses of α‐amino carbonyls via these methods are either limited regarding the amine source^[8]^ or constitute multi‐step endeavours.[^7a^, ^9^]

Our group and others have developed methodologies for hydrative functionalisations of alkynes (such as hydrative arylation and alkylation) based on [3,3]‐sigmatropic rearrangement strategies (Scheme 1A).[^10^, ^11^] In contrast, [2,3]‐ sigmatropic rearrangement is less explored,^[12]^ despite an early report by Sharpless in 1976 (Scheme 1B).^[13]^ In that work, allylamines were prepared by a sequence of ene‐reaction/[2,3]‐rearrangement.^[14]^ In order to establish a general method to construct α‐amino carbonyls by a hydrative amination transformation, we envisioned the deployment of readily available sulfinamides (such as commercially available *t*‐butylsulfinamide **2**) with activated alkynes. As shown in our proposal (Scheme 1C), we speculated that key intermediate **Int I** could undergo a [2,3]‐sigmatropic rearrangement to afford α‐amino carbonyl derivatives. Although, at the outset, the compatibility of the sulfinamide reagent with the Brønsted acidic conditions, as well as the ultimate fate of the putative N−S bonded species that necessarily results from arrow‐pushing, were unclear, we were intrigued by the possibility that such a process would result in a direct synthesis of α‐amino carbonyl compounds from activated alkynes. Perhaps most intriguing was the consideration as to whether attack by *nitrogen* or *oxygen* of the sulfinamide partner would take place, a matter to be settled eventually in the course of this work.

**Scheme 1:**
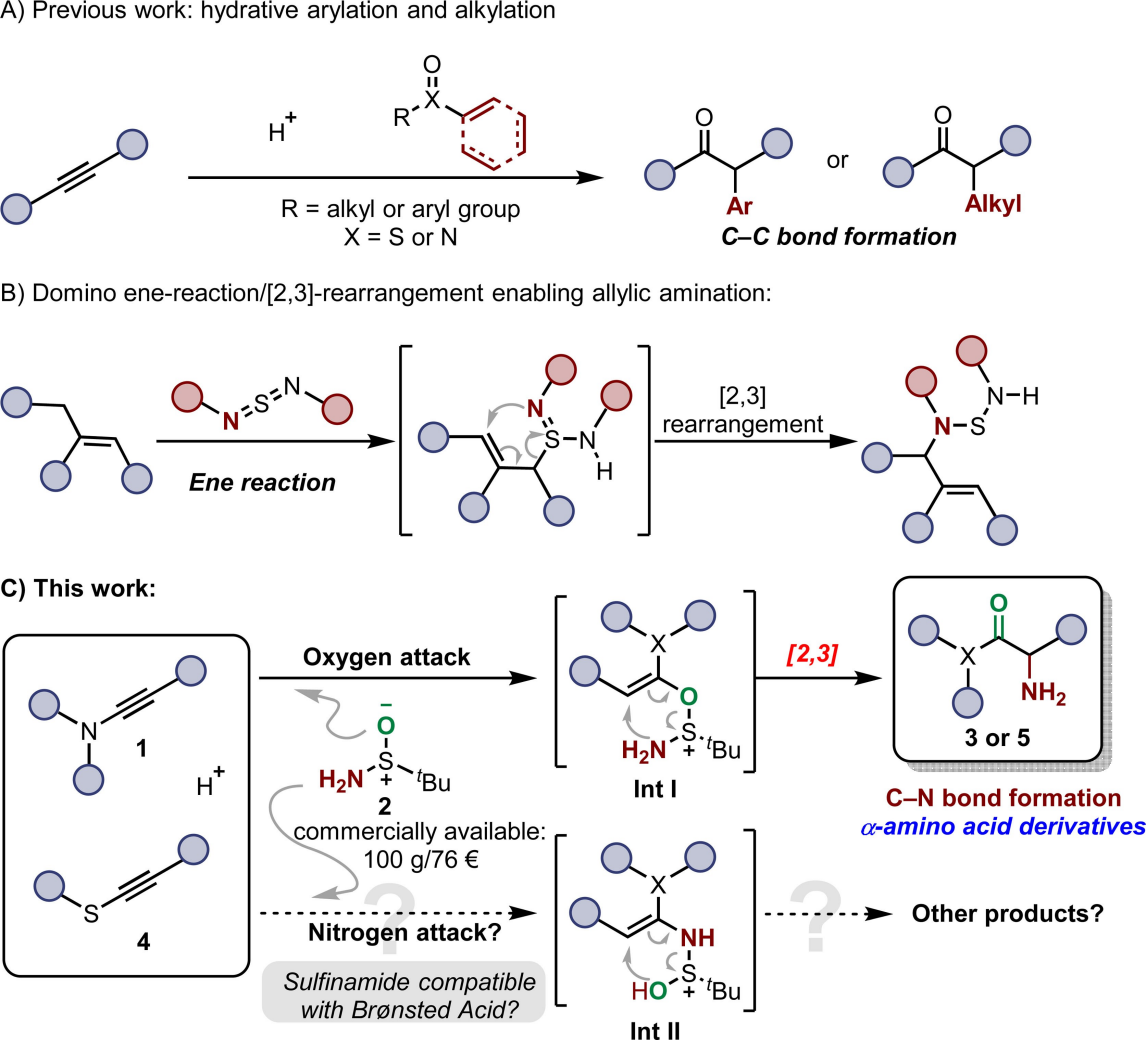
A) Previous work on hydrative arylation and alkylation of alkynes via [3,3]‐rearrangement. B) [2,3]‐Rearrangement enabling allylic amination. C) This work: synthesis of α‐amino acid derivatives by hydrative amination via sulfonium [2,3]‐rearrangement and possible competing attack through O/N.

At the start of our studies, we combined 3‐(hept‐1‐yn‐1‐yl)oxazolidin‐2‐one **1 a** with commercially available *t*‐butylsulfinamide **2** as model substrates, in the presence of a Brønsted acid. As shown in Table 1, while trifluoroacetic acid or *p*‐toluenesulfonic acid are ineffective for the activation of the ynamides (entry 1 & 2), the desired product **3 a** can be isolated in decent yield after in situ benzoyl protection when trifluoromethanesulfonic acid (table 1, entry 3) and trifluoromethanesulfonimide (table 1, entry 4) are utilized. Only trace amount of **3 a** were observed when lower reaction temperature (table 1, entry 5) or catalytic amount of trifluoromethanesulfonic acid (table 1, entry 6) were applied. Furthermore, owing to the basicity of the sulfinamide component, pre‐activation of the ynamide **1 a** with triflic acid was crucial for this transformation (table 1, entry 7).

**Table 1:**
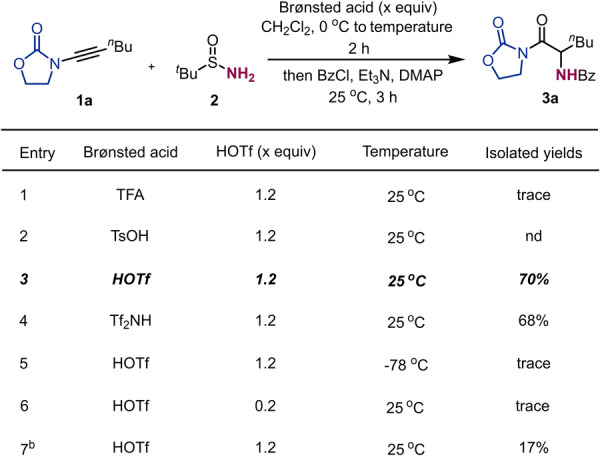
Optimisation of hydrative amination of ynamide **1 a**.^[a]^

[a] **1 a** (0.2 mmol,1.0 equiv in 1.0 mL of CH_2_Cl_2_), Brønsted acid (1.2 equiv) at 0 °C for 15 min, then **2** (2.0 equiv in 1.0 mL of CH_2_Cl_2_). After 2 h, Et_3_N (3.0 equiv), DMAP (5 mol %) and BzCl (3.0 equiv), 25 °C for 3 h. [b] Sulfinamide was added right after triflic acid was added. TFA: trifluoroacetic acid. HOTf: trifluoromethanesulfonic acid. Tf_2_NH: trifluoromethanesulfonimide.

Having suitable reaction conditions in hand, we investigated the scope of this transformation. As shown in Scheme 2, a wide range of ynamides bearing diverse linear chains afforded the desired products (**3 a**–**3 d**) in good yields. The structure of product **3 a** was confirmed by X‐ray diffraction analysis (CCDC 2171644).^[21]^ Importantly, the introduction of a halogen, phthalimide or conjugated alkene moiety to the ynamide partner (**3 e**, **3 f** and **3 i**) did not affect the efficiency of the process. Remarkably, the reaction was amenable to more hindered substrates, with successful installation of an amino group even at a neopentylic position (**3 g** and **3 h**). Ynamides bearing aromatic substituents afforded the desired products (**3 j**–**3 t**) in good to excellent yields. Worthy of note, an ynamide‐capped derivative of the drug febuxostat (**3 u**) could be tolerated in this chemistry.

**Scheme 2:**
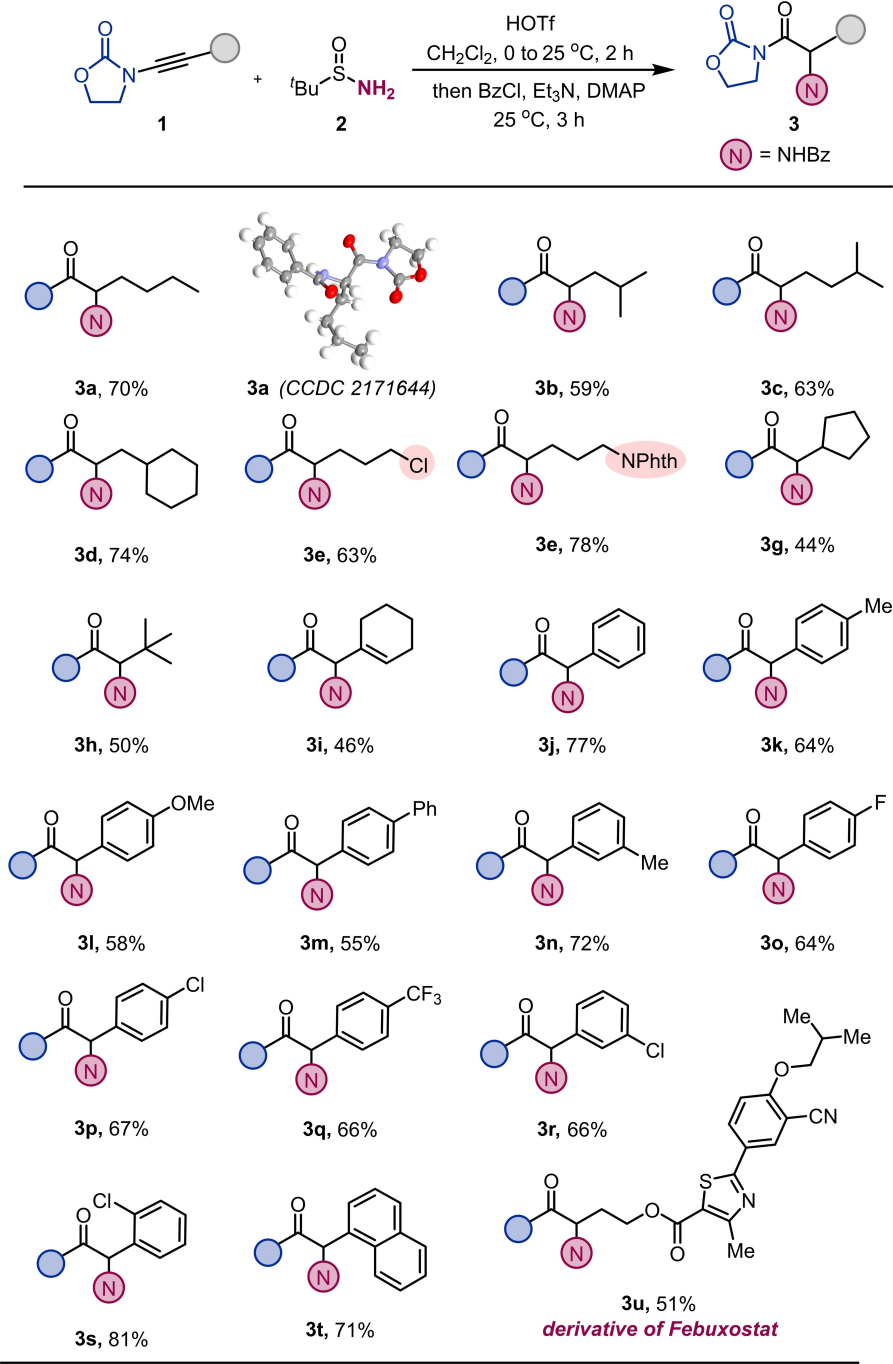
Hydrative amination of ynamides: **1** (0.2 mmol,1.0 equiv in 1.0 mL of CH_2_Cl_2_), HOTf (1.2 equiv) at 0 °C for 15 min, then **2** (2.0 equiv in 1.0 mL of CH_2_Cl_2_). After 2 h, Et_3_N (3.0 equiv), DMAP (5 mol %) and BzCl (3.0 equiv), 25 °C for 3 h.

Encouraged by the results obtained with ynamides, we next investigated thioalkynes. Considerable optimisation was needed at this stage (see Supporting Information for details). As a result, longer reaction times and lower amounts of triflic acid were found to be key to afford model product **5 a** in consistently high yields (Scheme 3). For some of the substrates, the use of trifluoromethanesulfonimide instead of triflic acid gave better yields of the corresponding α‐amino thioesters. Thioalkynes bearing a range of (cyclo)aliphatic residues smoothly afforded the desired products in very good yields (**5 a**–**5 g**). Once again, functional group tolerance was demonstrated with halide (**5 h**), acetate (**5 i**) and nitrogen‐containing substrates (**5 j**). A more hindered substrate (**4 k**), as well as such bearing aromatic substituents (**4 l**, **4 m**) and an *S*‐arylthioalkyne (**4 n**) all furnished the desired products (**5 k**–**5 n**) in moderate to good yields.

**Scheme 3:**
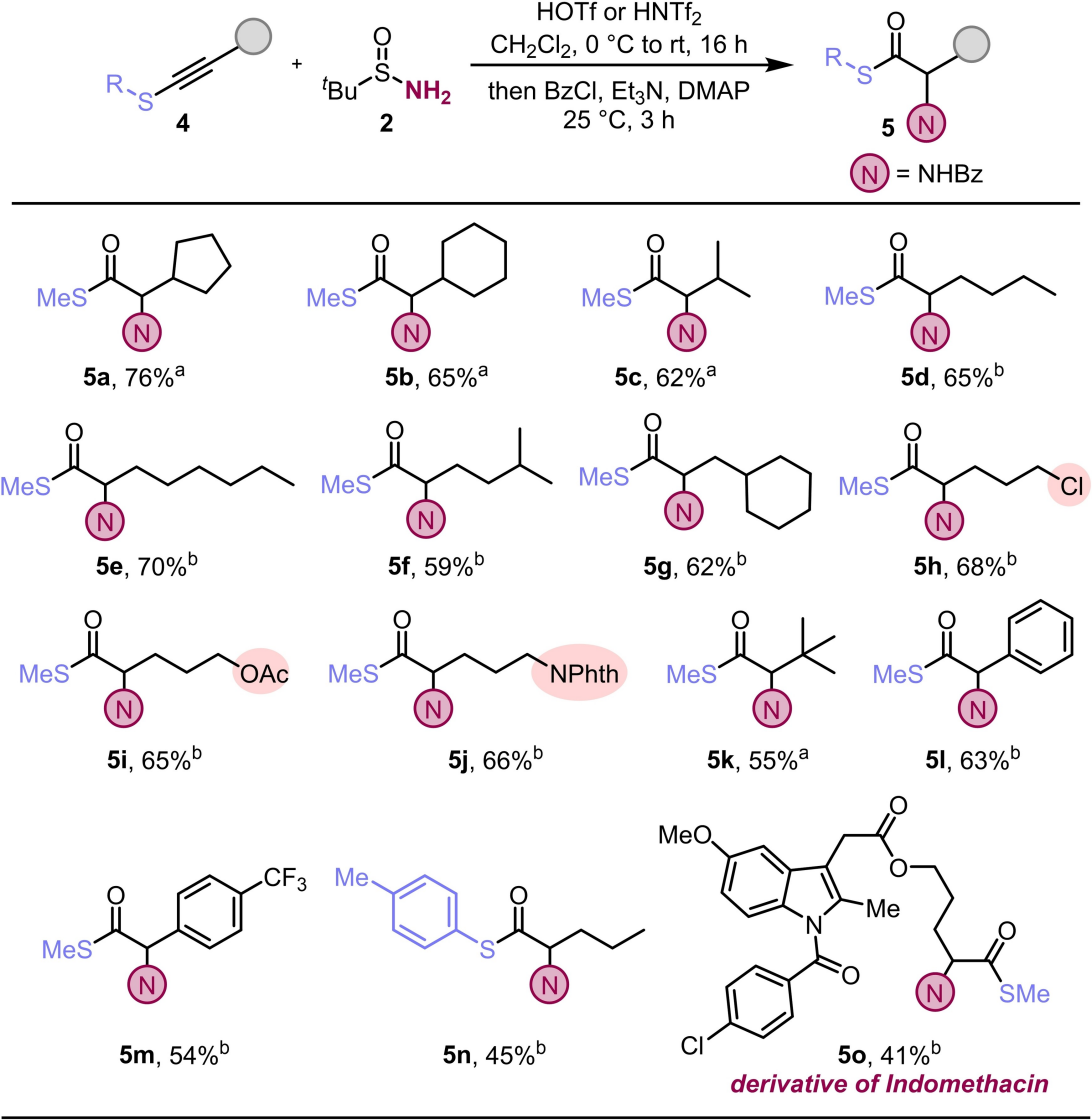
Hydrative amination of thioalkynes: a) **4** (0.2 mmol,1.0 equiv in 1.0 mL of CH_2_Cl_2_), HOTf at 0 °C for 15 min (1.2 equiv), then **2** (2.0 equiv in 1.0 mL of CH_2_Cl_2_). After 16 h, Et_3_N (3.0 equiv), DMAP (5 mol %) and BzCl (3.0 equiv) were added in sequence, 25 °C for 3 h. b) **4** (0.2 mmol,1.0 eq in 1.0 mL of CH_2_Cl_2_), HNTf_2_ at 0 °C for 15 min (0.6 equiv in 0.4 mL of CH_2_Cl_2_), then **2** (1.2 equiv in 0.4 mL of CH_2_Cl_2_). After 1 h, HNTf_2_ at 0 °C for 15 min (0.4 equiv in 0.3 mL of CH_2_Cl_2_), then **2** (0.8 equiv in 0.3 mL of CH_2_Cl_2_). After 16 h, Et_3_N (3.0 equiv), DMAP (5 mol %) and BzCl (3.0 equiv) were added in sequence, 25 °C for 3 h.

To elucidate the mechanism of this process, quantum chemical calculations were performed at the B3LYP‐D3BJ/def2‐TZVP,SMD(DCM)//B3LYP‐D3BJ/def2‐SVP,SMD(DCM) level of theory^[15]^ (see Supporting Information for details). The computed Gibbs free energy profile is presented in Figure 1. The first step, assuming transient formation of a keteniminium ion by ynamide activation, as previously demonstrated,^[16]^ is a nucleophilic attack which can result in an intermediate bearing either *Z‐* (profile in blue) or *E‐*double bond configuration (profile in purple). As the sulfinamide reactant contains two nucleophilic centres (oxygen and nitrogen), nucleophilic attack to the keteniminium ion was also considered by either O (steps **A**→ ***E*** **‐B** and **A**→ ***Z*** **‐B**, right) or N (steps **A**→ ***E*** **‐D** and **A**→ ***Z*** **‐D**, left). The results were unambiguousand in agreement with experiment: attack via the oxygen atom on the face of the keteniminium ion leading to an *E*‐configured intermediate (**A**→ ***E*** **‐B**) presents the lowest activation barrier (Δ*G* ^≠^(**A**→ ***E*** **‐B**)=5.5 kcal mol^−1^), while the corresponding attack resulting in a *Z‐*configured species has an activation barrier that lies 3.4 kcal mol^−1^ higher (Δ*G* ^≠^(**A**→ ***Z*** **‐B**)=8.9 kcal mol^−1^). Both of these steps are kinetically favoured when compared to the attack by nitrogen (Δ*G* ^≠^(**A**→ ***E*** **‐D**)=10.8 kcal mol^−1^ and Δ*G* ^≠^(**A**→ ***Z*** **‐D**)=15.2 kcal mol^−1^). In fact, the transition state structure **TS**_***E*** **‐AB**_ presents a distance of 2.96 Å between the carbon of the keteniminium ion and the oxygen of the sulfinamide, while in **TS**_***E*** **‐AD**_ the equivalent distance to the nitrogen is 2.59 Å, showing that the O attack can occur at longer distances between reagents. Furthermore, the N attack results in an S−N bond elongation of over 0.3 Å from structure **A** (*D*_S−N_=1.70 Å) to either intermediates ***E*** **‐D** or ***Z*** **‐D** (*D*_S−N_=2.01 Å and 2.07 Å in ***E*** **‐D** and ***Z*** **‐D** respectively), whereas this destabilising effect is less pronounced for the S−O bond in the formation of intermediates ***E*** **‐B** and ***Z*** **‐B**, obtained through oxygen attack (elongation of *D*_S−O_ by 0.21 Å from **A** to either ***E*** **‐B** or ***Z*** **‐B**). As a result, the formation of intermediates ***E*** **‐B** and ***Z*** **‐B** (via O attack) is thermodynamically favoured (Δ*G*(**A**→ ***E*** **‐D**)=−17.7 kcal mol^−1^ and Δ*G*(**A**→ ***Z*** **‐D**)=−16.2 kcal mol^−1^ while Δ*G*(**A**→**E‐B**)=−21.5 kcal mol^−1^ and Δ*G*(**A**→ ***Z*** **‐B**)=−21.7 kcal mol^−1^), resulting in the observed selectivity of the reaction. These have equivalent thermodynamic stability and can easily undergo a [2,3]‐sigmatropic rearrangement (steps ***E*** **‐B**→**C** and ***Z*** **‐B**→**C**) which occurs in a single step. This step cleaves the S−O bond, forms a C−N bond and leads to intermediate **C**. This step has a low activation barrier (Δ*G* ^≠^(***E*** **‐B**→**C**)=9.4 kcal mol^−1^ and Δ*G* ^≠^(***Z*** **‐B**→**C**)=12.7 kcal mol^−1^) and is highly exergonic. Intermediate **C** can be then converted into the experimentally observed product through sulfur extrusion which can be promoted by triethylamine (see Supporting Information for details), followed by the amine protection step.

**Figure 1:**
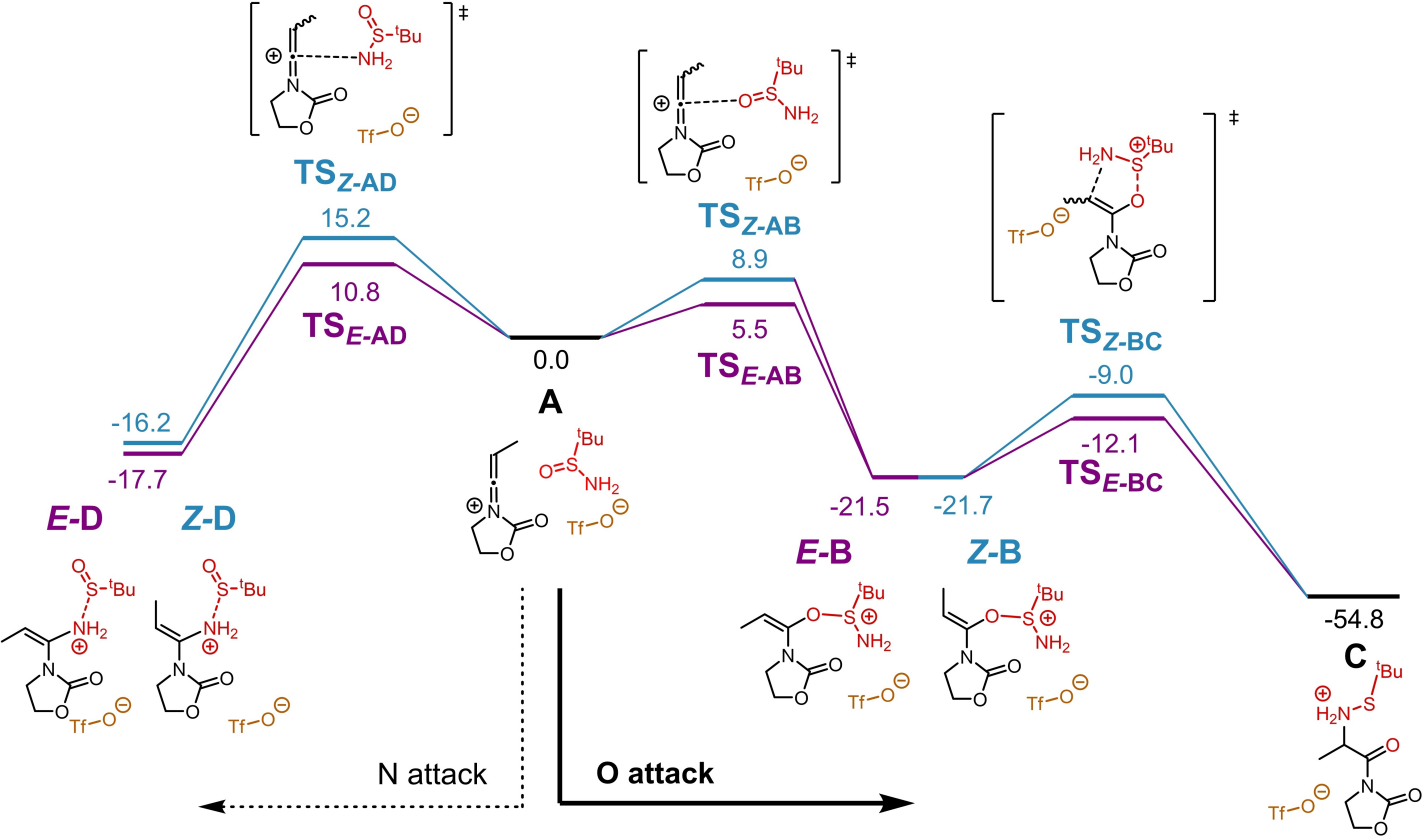
Computed Gibbs free energy profiles of the reaction of the sulfinamide with the keteniminium ion: leading to formation of a double bond with *Z*‐ (profile in blue) or *E*‐ (profile in purple) configuration. Relative Gibbs free energies are presented in kcal mol^−1^ (298 K). The reactant complex (**A**) is a reference (0.0 kcal mol^−1^).

The path leading to intermediates containing an *E‐*olefin (profile in purple) presents lower activation barriers than the path involving the formation of *Z‐*olefins for all computed profiles. This results mainly from the steric repulsion between the methyl group present in the keteniminium and the sulfinamide, which contains a bulky *t*Bu group. The proposed [2,3]‐sigmatropic rearrangement from structure ***E*** **‐B** was also compared to alternative routes, including S−O bond dissociations and proton transfers, all found to be less viable than the pathway presented herein (see Supporting Information, Figure S1 for details).

Enantiopure *tert*‐butylsulfinamide is known as a versatile auxiliary in asymmetric synthesis of amine building blocks.^[17]^ We thus examined the possibility of chirality transfer in this [2,3]‐rearrangement. Excellent enantioselectivities were obtained when sterically hindered ynamides/thioalkynes were involved (Scheme 4A, **(*S*)‐3 h** and **(*S*)‐5 k**), with decreased selectivity observed for less bulky substrates, particularly in the case of ynamides (**(*S*)‐3 g** and **(*S*)‐3 a**). The absolute configuration of the compound **(*S*)‐5 k** was confirmed by X‐ray diffraction analysis (CCDC 2208631).^[21]^ Interestingly, thioalkynes undergo this rearrangement with uniformly better enantioselectivity than ynamides, an observation with potential mechanistic implications. Indeed, considering that the pathway involving intermediates with an *E‐* configured carbon‐carbon double bond is favored (Figure 1), the step that determines the enantioselectivity of the product is the [2,3]‐sigmatropic rearrangement event. To understand the observed differences between ynamides and thioalkynes, we computed the Gibbs free energy gap between the transition states leading to (*S*)*‐* or (*R*)*‐*products (Scheme 4B). For ynamides (Scheme 4B left), only the (*R*) configured transition state, **TS**_***E‐*** **BC (*R*)**_, presents significant steric clash between the oxazolidinone and the *t*Bu groups (highlighted in orange). However, both (*S*)*‐* and (*R*)*‐*transition states benefit from intramolecular hydrogen bond stabilization (highlighted in yellow) to the oxazolidinone carbonyl, which is slightly stronger (0.12 Å shorter) in the (*R*)*‐*transition state, thus resulting in a very low difference in activation barriers (ΔΔ*G* ^≠^=1.2 kcal mol^−1^). This H‐bond is absent from the thioalkyne case (Scheme 4B right), in which the predominant factor for the enantioselectivity is steric clash between the SMe and the *t*Bu groups (highlighted in orange) in the transition state leading to the (*R*)*‐*product, **TS**_***E‐*** **S*M*e (*R*)**_. This effect results in a slightly higher activation energy gap (ΔΔ*G* ^≠^=1.8 kcal mol^−1^) which directly correlates with better enantioselectivity, as experimentally observed.

**Scheme 4:**
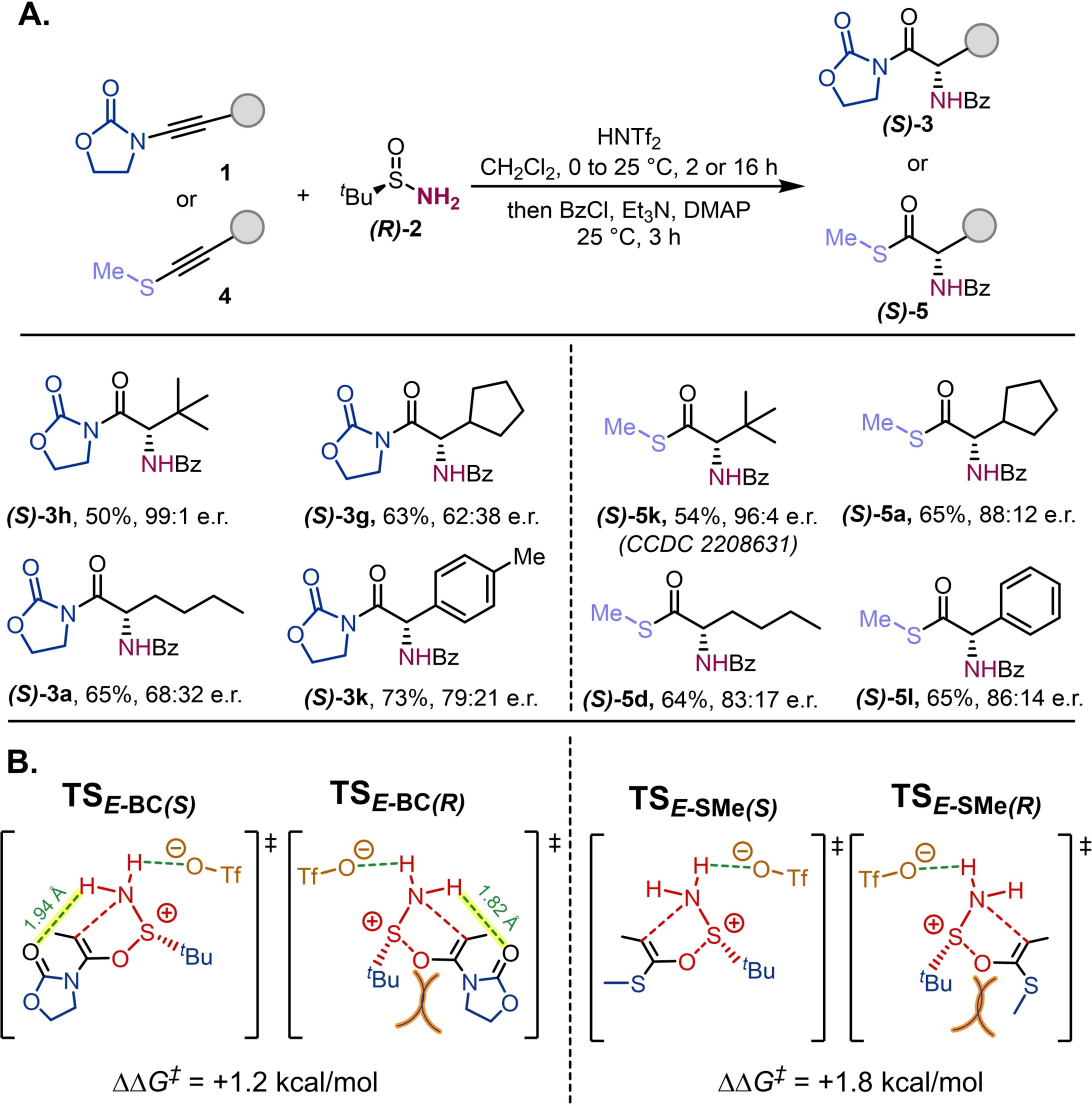
Chirality transfer study: A) Reaction with enantiopure (*R*)‐*tert*‐butylsulfinamide: **1** (0.2 mmol,1.0 equiv in 1.0 mL of CH_2_Cl_2_), HNTf_2_ (68.0 mg, 0.24 mmol, 1.2 equiv in 1.0 mL of CH_2_Cl_2_) at 0 °C for 15 min, then (*R*)‐**2** (48.5 mg, 0.40 mmol, 2.0 equiv). After 2 h (for ynamides) or 16 h (for thioalkynes), Et_3_N (3.0 equiv), DMAP (5 mol %) and BzCl (3.0 equiv), 25 °C for 3 h. Enantiomeric ratios (e.r.) determined by chiral HPLC. B) Computed Gibbs free energy energy gap between the transition states leading to (*R*)‐ or (*S*)‐products.

Keen to explore the utility of our products (Scheme 5), we recognised that the presence of an acyl oxazolidinone in structures such as **3 d** readily allows both hydrolysis and reduction to give the corresponding α‐amino acid **6** and β‐amino alcohol **7**. Conversely, thioester products such as **5 n** could be converted to α‐amino ester **8** ^[18]^ or ‐amide **9** ^[19]^ in very good yields. Finally, a direct cross‐coupling with boronic acid **11** afforded α‐amino ketone **10** in excellent yield.^[20]^

**Scheme 5:**
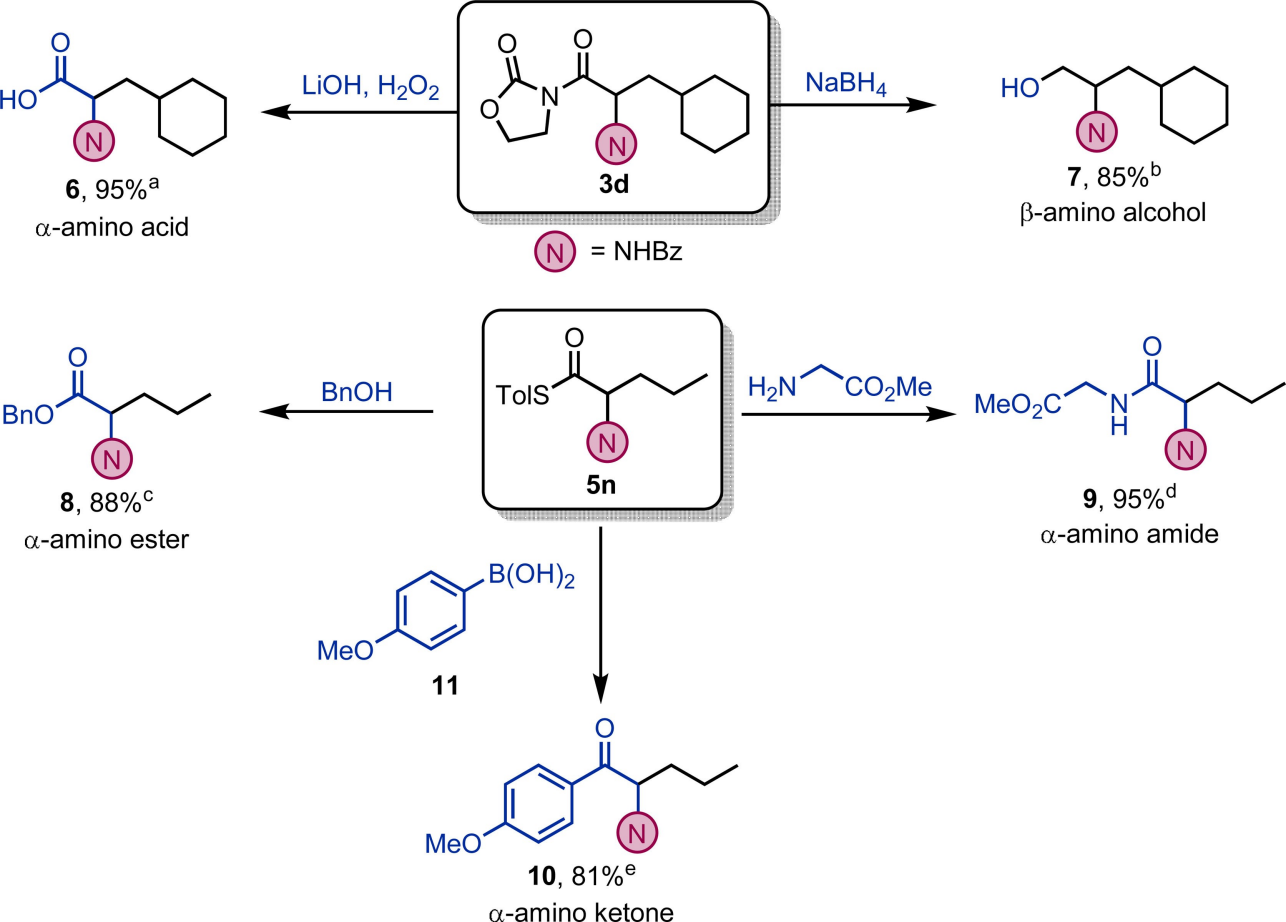
Post‐functionalisation reactions: a) **3 d** (1.0 equiv), LiOH (1.1 equiv) and aq. H_2_O_2_ 30 % (5.0 equiv), THF/H_2_O 3 : 1, 25 °C for 2 h. b) **3 d** (1.0 equiv), NaBH_4_ (5.0 equiv), MeOH, 0 to 25 °C for 4 h. c) **5 n** (1.0 equiv), BnOH (1.2 equiv), THF, 25 °C for 3 h. d) **5 n** (0.1 mmol), bis(trimethylsilyl)acetamide (1.5 equiv), glycine methyl ester hydrochloride (3.0 equiv), Et_3_N (3.0 equiv), THF, 40 °C for 14 h. e) **5 n** (1.0 equiv), boronic acid **11** (0.15 mmol), Pd(dba)_2_ (10 mol %), P(OEt)_3_ (20 mol %), CuTC (1.5 equiv), THF, 30 °C, 14 h.

In conclusion, we have reported herein a hydrative amination of ynamides and thioalkynes under metal‐free and mild conditions. This practical strategy offers a new and convenient avenue for the synthesis of α‐amino acid derivatives from readily available sulfinamides as nitrogen sources. Computational studies support a mechanism proceeding by an underexplored sulfonium [2,3]‐sigmatropic rearrangement, showcasing the immense untapped potential in this chemistry.

## Conflict of interest

The authors declare no conflict of interest.

## Supporting information

As a service to our authors and readers, this journal provides supporting information supplied by the authors. Such materials are peer reviewed and may be re‐organized for online delivery, but are not copy‐edited or typeset. Technical support issues arising from supporting information (other than missing files) should be addressed to the authors.

Supporting Information

Supporting Information

Supporting Information

Supporting Information

Supporting Information

## Data Availability

The data that support the findings of this study are available from the corresponding author upon reasonable request.
