# Supplementary material for: Direct Synthesis of α‐Amino Acid Derivatives by Hydrative Amination of Alkynes
Source: Angew Chem Int Ed Engl. 2022 Nov 29;62(1):e202212399. doi: 10.1002/anie.202212399 (PMC10098499; doi:10.1002/anie.202212399)
Supplement: Supplementary file 1 — Supporting Information [file ANIE-62-0-s001.pdf]

Angewandte Chemie
Eine Zeitschrift der Gesellschaft Deutscher Chemiker

Supporting Information

Direct Synthesis of α-Amino Acid Derivatives by Hydrative Amination of Alkynes

*M. Feng, R. Tinelli, R. Meyrelles, L. González, B. Maryasin, N. Maulide**

Supplementary Information

Contents

1. General Information 2
2. Substrate preparation 3
2.1 Preparation of the ynamides 3
2.2 Characterizations of the prepared ynamides 3
2.3 Preparation of the thioalkynes 9
2.4 Characterizations of the prepared thioalkynes 9
3. Optimizations of hydrative amination 14
3.1 Optimizations of hydrative amination of ynamide 14
3.2 Optimizations of hydrative amination of thioalkynes 15
4. Synthesis of α-amino amides and α-amino thioesters *via* hydrative amination 17
4.1 Hydrative amination of ynamides 17
4.2 Hydrative amination of thioalkynes 17
4.3 Characterizations of the α-amino amides 18
4.4 Characterizations of the α-amino thioesters 28
5. Chirality transfer study 35
5.1 Conditions optimization 35
5.2 Hydrative amination of ynamides with enantioenriched sulfinamide 36
5.3 Hydrative amination of thioalkynes with enantioenriched sulfinamide 39
6. Post-functionalizations of α-amino amides and α-amino thioesters 43
7. Computational details 47
7.1 Computational method 47
7.2 Additional computational results 47
7.3 Cartesian coordinates (XYZ) 50
8. X-Ray Crystallographic data for compound 3a and (*S*)-5k 58
9. NMR Spectra 65
10. References 112

1. General Information

Unless otherwise stated, all glassware was flame-dried before use and all reactions were performed under an atmosphere of argon. All solvents were distilled from appropriate drying agents prior to use or, if purchased in anhydrous form, used as received from commercial suppliers. Triflic anhydride was distilled over P_4O_{10} prior to use. All other reagents were used as received from commercial suppliers, unless otherwise stated. Reaction progress was monitored by thin layer chromatography (TLC) performed on aluminium plates coated with silica gel F_{254} with 0.2 mm thickness. Chromatograms were visualized by fluorescence quenching with UV light at 254 nm or by staining using potassium permanganate. Flash column chromatography was performed using silica gel 60 (230-400 mesh, Merck and co.). Neat infra-red spectra were recorded using a Perkin-Elmer Spectrum 100 FT-IR spectrometer. Wavenumbers (vmax) are reported in cm^{-1}. Mass spectra were obtained using a Finnigan MAT 8200 or (70 eV) or an Agilent 5973 (70 eV) spectrometer, using electrospray ionization (ESI). All ^{1}H NMR, ^{13}C NMR and ^{19}F NMR spectra were recorded using a Bruker AV-400, AV-600 or AV-700 spectrometer at 300K. Chemical shifts are given in parts per million (ppm, δ), referenced to the solvent peak of $CDCl_3$, defined at δ = 7.26 ppm (^{1}H NMR) and δ = 77.16 (^{13}C NMR). Coupling constants are quoted in Hz (*J*). ^{1}H NMR splitting patterns were designated as singlet (s), doublet (d), triplet (t), quartet (q), pentet (p). Splitting patterns that could not be interpreted or easily visualized were designated as multiplet (m) or broad (br). Selected ^{13}C NMR spectra were recorded using the attached proton test (APT) to facilitate the confirmation and assignment of the structure.

2. Substrate preparation

2.1 Preparation of the ynamides

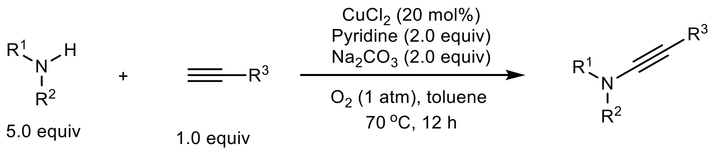

General procedure 1: $CuCl_2$ (20 mol%), amine (5.00 equiv.) and Na_2CO_3 (2.00 equiv.) were added to a round bottom flask. The flask was subsequently purged with oxygen for 15 min, after which a solution of pyridine (2.00 equiv.) in dry toluene (0.2 M) was added. A balloon filled with oxygen was connected to the flask and the flask was heated at 70 °C. After 15 min, a solution of the corresponding alkyne (1.00 equiv.) in dry toluene (0.2 M) was added over the course of 4 h using syringe pump addition. After completion of the addition, the mixture was stirred at 70 °C for another 12 h and was then cooled to ambient temperature. The reaction mixture was concentrated under reduced pressure after filtration through Celite and the residue was purified by flash chromatography on silica gel with hexane/ethyl acetate.

2.2 Characterizations of the prepared ynamides

3-(Hex-1-yn-1-yl)oxazolidin-2-one (1a)

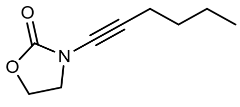

Prepared according to the General Procedure 1 in 60% yield. All analytical data were in good accordance with those reported in the literature.[1]

3-(4-Methylpent-1-yn-1-yl)oxazolidin-2-one (1b)

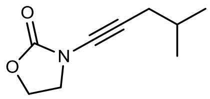

Prepared according to the General Procedure 1 in 57% yield. All analytical data were in good accordance with those reported in the literature.[2]

3-(5-Methylhex-1-yn-1-yl)oxazolidin-2-one (1c)

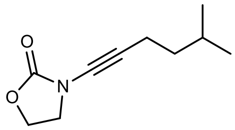

Prepared according to the General Procedure 1 in 40% yield. All analytical data were in good accordance with those reported in the literature.[3]

3-(3-Cyclohexylprop-1-yn-1-yl)oxazolidin-2-one (1d)

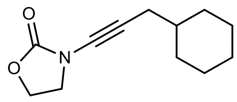

Prepared according to the General Procedure 1 in 50% yield. All analytical data were in good accordance with those reported in the literature.[2]

3-(5-Chloropent-1-yn-1-yl)oxazolidin-2-one (1e)

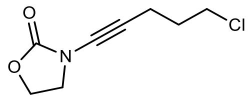

Prepared according to the General Procedure 1 in 52% yield. All analytical data were in good accordance with those reported in the literature.[4]

2-(5-(2-Oxooxazolidin-3-yl)pent-4-yn-1-yl)isoindoline-1,3-dione (1f)

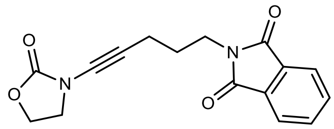

Prepared according to the General Procedure 1 in 70% yield. All analytical data were in good accordance with those reported in the literature.[5]

3-(Cyclopentylethynyl)oxazolidin-2-one (1g)

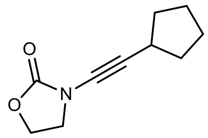

Prepared according to the General Procedure 1 in 79% yield. All analytical data were in good accordance with those reported in the literature.[2]

3-(3,3-Dimethylbut-1-yn-1-yl)oxazolidin-2-one (1h)

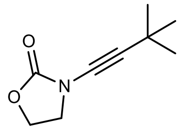

Prepared according to the General Procedure 1 in 44% yield. All analytical data were in good accordance with those reported in the literature.[4]

3-(Cyclohex-1-en-1-ylethynyl)oxazolidin-2-one (1i)

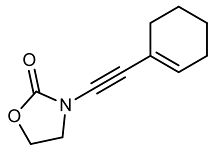

Prepared according to the General Procedure 1 in 71% yield. All analytical data were in good accordance with those reported in the literature.[5]

3-(Phenylethynyl)oxazolidin-2-one (1j)

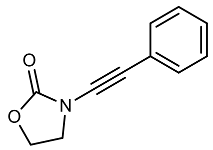

Prepared according to the General Procedure 1 in 81% yield. All analytical data were in good accordance with those reported in the literature.[6]

3-(*p*-Tolylethynyl)oxazolidin-2-one (1k)

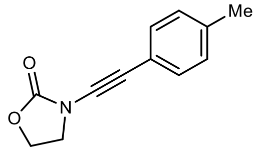

Prepared according to the General Procedure 1 in 70% yield. All analytical data were in good accordance with those reported in the literature.[6]

3-((4-Methoxyphenyl)ethynyl)oxazolidin-2-one (1l)

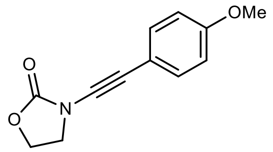

Prepared according to the General Procedure 1 in 88% yield. All analytical data were in good accordance with those reported in the literature.[7]

3-([1,1'-Biphenyl]-4-ylethynyl)oxazolidin-2-one (1m)

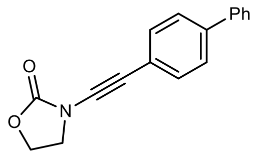

Prepared according to the General Procedure 1 in 41% yield. All analytical data were in good accordance with those reported in the literature.[8]

3-(*m*-Tolylethynyl)oxazolidin-2-one (1n)

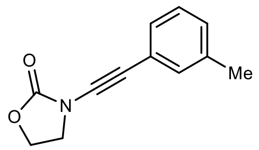

Prepared according to the General Procedure 1 in 84% yield. All analytical data were in good accordance with those reported in the literature.[7]

3-((4-Fluorophenyl)ethynyl)oxazolidin-2-one (1o)

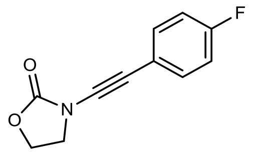

Prepared according to the General Procedure 1 in 63% yield. All analytical data were in good accordance with those reported in the literature.[6]

3-((4-Chlorophenyl)ethynyl)oxazolidin-2-one (1p)

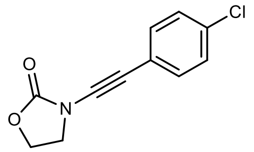

Prepared according to the General Procedure 1 in 55% yield. All analytical data were in good accordance with those reported in the literature.[9]

3-((4-(Trifluoromethyl)phenyl)ethynyl)oxazolidin-2-one (1q)

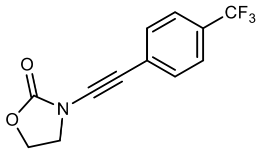

Prepared according to the General Procedure 1 in 44% yield. All analytical data were in good accordance with those reported in the literature.[8]

3-((3-Chlorophenyl)ethynyl)oxazolidin-2-one (1r)

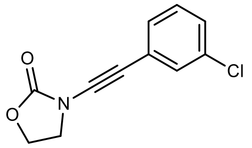

Prepared according to the General Procedure 1 in 70% yield. All analytical data were in good accordance with those reported in the literature.[9]

3-((2-Chlorophenyl)ethynyl)oxazolidin-2-one (1s)

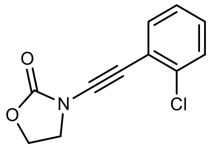

Prepared according to the General Procedure 1 in 75% yield. All analytical data were in good accordance with those reported in the literature.[9]

3-(Naphthalen-1-ylethynyl)oxazolidin-2-one (1t)

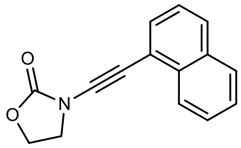

Prepared according to the General Procedure 1 in 77% yield. All analytical data were in good accordance with those reported in the literature.[10]

4-(2-Oxooxazolidin-3-yl)but-3-yn-1-yl 2-(3-cyano-4-isobutoxyphenyl)-4-methylthiazole-5-carboxylate (1u)

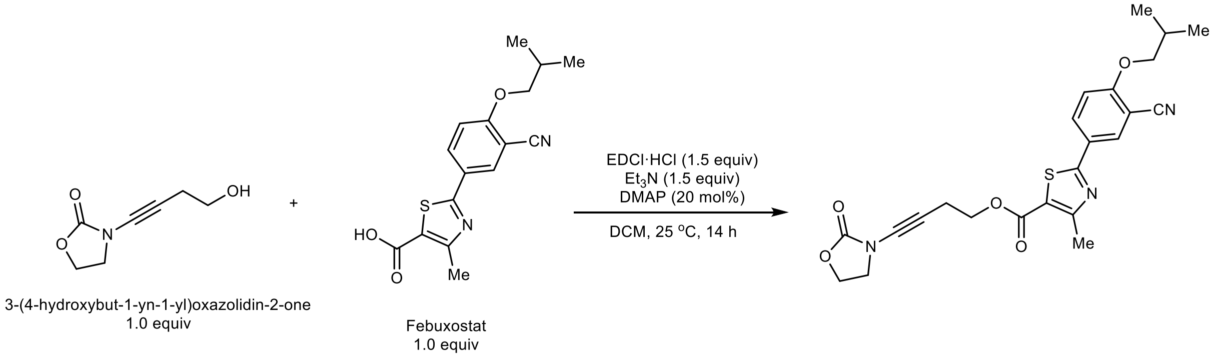

To a solution of Febuxostat (221 mg, 0.7 mmol, 1.00 equiv.) in dichloromethane (4.0 mL) were added 1-ethyl-3-(3-(dimethylamino)propyl)-carbodiimide hydrochloride (EDCI) (201 mg, 1.05 mmol, 1.50 equiv.), *N,N*-dimethylaminopyridine (17.5 mg, 0.14 mmol, 20 mol%) and then the 3-(4-hydroxybut-1-yn-1-yl)oxazolidin-2-one (prepared according to the literature[11], 109 mg, 0.7 mmol, 1.0 equiv.).The reaction mixture was stirred at 25 °C for 14 h. Dichloromethane (10 mL) was added, and the mixture was washed with 1% HCl (2 mL), brine solution (5 mL) and dried over Na_2SO_4. After removal of the solvent, the residue was purified by column chromatography on silica gel with EtOAc/heptane mixture as eluent to give the desired ynamide in 73% yield.

^{1}H NMR (600 MHz, $CDCl_3$) δ 8.16 (d, *J* = 2.2 Hz, 1H), 8.08 (dd, *J* = 8.8, 2.2 Hz, 1H), 7.00 (d, *J* = 8.9 Hz, 1H), 4.40 (dt, *J* = 13.5, 7.2 Hz, 4H), 3.88 (dd, *J* = 9.7, 7.1 Hz, 4H), 2.84 – 2.70 (m, 5H), 2.19 (dp, *J* = 13.3, 6.6 Hz, 1H), 1.08 (d, *J* = 6.7 Hz, 6H).

^{13}C NMR (150 MHz, $CDCl_3$) δ 167.5, 162.5, 161.7, 161.5, 156.4, 132.6, 132.0, 125.9, 121.4, 115.3, 112.6, 102.9, 75.7, 71.7, 66.7, 63.0, 62.9, 46.8, 28.1, 19.1, 19.0 (2C), 17.5.

IR (neat) ν: 2214, 1774, 1714, 1606, 1508, 1476, 1431, 1389, 1297, 1202, 1096, 1037, 1011, 751 cm^{-1}.

HRMS (ESI$^+$): exact mass calculated for $[M+Na]^+$ ($C_{23}H_{23}SN_3O_5Na^+$) requires m/z 476.1251, found m/z 476.1250.

2.3 Preparation of the thioalkynes

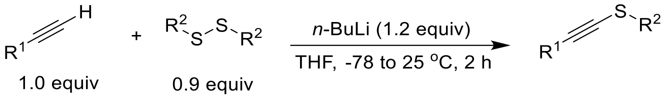

General procedure 2: To a solution of 1-alkyne (9.0 mmol, 1.00 equiv.) in of THF (0.3 M) in a flame dried Schlenk flask, a solution of *n*-BuLi in hexanes 1.6 M (10.8 mmol, 1.20 equiv, 6.75 ml) was added dropwise at -78 °C. The resulting reaction mixture was stirred at the same temperature for one hour, whereupon the disulfide (8.1 mmol, 0.90 eq, 0.73 ml) was added. The solution was allowed to warm to 25 °C while stirring over the course of 1 h. Subsequently, the reaction was cooled back at 0 °C, and quenched by the addition of a saturated aqueous solution of NH_4Cl. The product was extracted with Et_2O and the combined organic phase was dried over $MgSO_4$, filtered and the solvent was removed under reduced pressure. The product was purified by flash chromatography on silica gel with pentane/CH_2Cl_2.

2.4 Characterizations of the prepared thioalkynes

(Cyclopentylethynyl)(methyl)sulfane (4a)

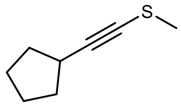

Prepared according to the General Procedure 2 in 88% yield. All analytical data were in good accordance with those reported in the literature.[12]

(Cyclohexylethynyl)(methyl)sulfane (4b)

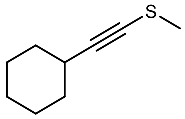

Prepared according to the General Procedure 2 in 75% yield. All analytical data were in good accordance with those reported in the literature.[13]

Methyl(3-methylbut-1-yn-1-yl)sulfane (4c)

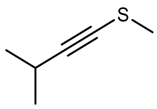

Prepared according to the General Procedure 2 in 42% yield. All analytical data were in good accordance with those reported in the literature.[13]

Hex-1-yn-1-yl(methyl)sulfane (4d)

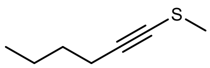

Prepared according to the General Procedure 2 in 54% yield. All analytical data were in good accordance with those reported in the literature. [14]

methyl(oct-1-yn-1-yl)sulfane (4e)

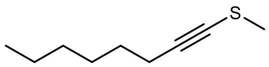

Prepared according to the General Procedure 2 in 52% yield. All analytical data were in good accordance with those reported in the literature. [14]

Methyl(5-methylhex-1-yn-1-yl)sulfane (4f)

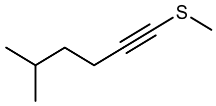

Prepared according to the General Procedure 2 in 65% yield. All analytical data were in good accordance with those reported in the literature.[15]

(3-Cyclohexylprop-1-yn-1-yl)(methyl)sulfane (4g)

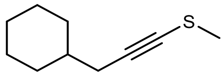

Prepared according to the General Procedure 2 in 32% yield.

^{1}H NMR (700 MHz, $CDCl_3$) δ 2.35 (s, 3H), 2.18 (d, *J* = 6.7 Hz, 2H), 1.81 – 1.75 (m, 2H), 1.73 – 1.69 (m, 2H), 1.65 (dddd, *J* = 11.5, 5.1, 3.3, 1.8 Hz, 1H), 1.49 – 1.42 (m, 1H), 1.27 – 1.20 (m, 2H), 1.17 – 1.10 (m, 1H), 0.97 (ddd, *J* = 24.5, 12.6, 3.3 Hz, 2H).

^{13}C NMR (175 MHz, $CDCl_3$) δ 92.4, 70.7, 37.6, 32.8 (2C), 28.0, 26.4 (2C), 26.3, 19.5.

IR (neat) ν: 2956, 2928 1467, 1433, 1344, 976 cm^{-1}.

HRMS (ESI^+): exact mass calculated for $[M+H]^+$ ($C_{10}H_{17}S^+$) requires m/z 169.1038, found m/z 169.1045.

(5-Chloropent-1-yn-1-yl)(methyl)sulfane (4h)

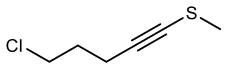

Prepared according to the General Procedure 2 in 51% yield. All analytical data were in good accordance with those reported in the literature. [14]

5-(Methylthio)pent-4-yn-1-yl acetate (4i)

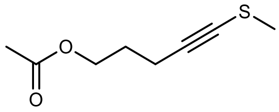

To a solution of (5-chloropent-1-yn-1-yl)(methyl)sulfane (297 mg, 2 mmol, 1.00 equiv.) in DMF (0.2 M) in a flame dried Schlenk flask, potassium acetate (585 mg, 6 mmol, 3.00 equiv.) was added and stirred at 25 °C followed by TLC monitoring. Upon completion, the reaction was quenched by addition of a saturated aqueous solution of $NaHCO_3$. Extraction of the product was carried out with Et_2O (3 x 20 mL) and the combined organic phase was washed multiple times with brine. Upon drying over $MgSO_4$ and filtration, the solvent was removed under reduced pressure. The product was purified by flash chromatography on silica gel (pentane/dichloromethane 9:1) to afford the title compound in 44% yield. All analytical data were in good accordance with those reported in the literature.[14]

2-(5-(Methylthio)pent-4-yn-1-yl)isoindoline-1,3-dione (4j)

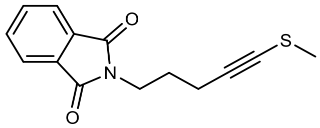

To a solution of (5-chloropent-1-yn-1-yl)(methyl)sulfane (297 mg, 2 mmol, 1.00 equiv.) in DMF (0.2 M) in a flame dried Schlenk flask, the potassium phthalimide (1.11 g, 6 mmol, 3.00 equiv.) was added and stirred at 25 °C, followed by TLC monitoring. Upon completion, the reaction was quenched by addition of a saturated aqueous solution of $NaHCO_3$. Extraction of the product was carried out with Et_2O (3 x 20 mL) and the combined organic phase was washed multiple times with brine. Upon drying over $MgSO_4$ and filtration, the solvent was removed under reduced pressure. The product was purified by flash chromatography on silica gel (pentane/dichloromethane 9:1) to afford the title compound in 68% yield. All analytical data were in good accordance with those reported in the literature.[14]

(3,3-Dimethylbut-1-yn-1-yl)(methyl)sulfane (4k)

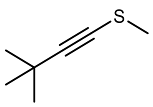

Prepared according to the General Procedure 2 in 21% yield. All analytical data were in good accordance with those reported in the literature. [13]

Methyl(phenylethynyl)sulfane (4l)

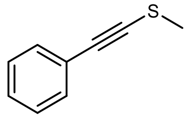

Prepared according to the General Procedure 2 in 90% yield. All analytical data were in good accordance with those reported in the literature.[16]

Methyl((4-(trifluoromethyl)phenyl)ethynyl)sulfane (4m)

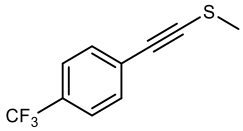

Prepared according to the General Procedure 2 in 40% yield. All analytical data were in good accordance with those reported in the literature.[3]

Hex-1-yn-1-yl(*p*-tolyl)sulfane (4n)

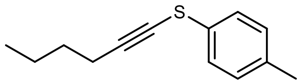

Preparation of the disulfide *in-situ*: In a flame dried flask, *p*-toluenethiol (2.73 g, 22 mmol, 2.20 equiv.) was dissolved in dry CH_2Cl_2 (1.0 M). The solution was stirred at 0 °C for 10 mins when sulfuryl chloride (1.62 g, 12 mmol, 1.20 equiv.) was added in dropwise. The solution was stirred for 30 mins at 25 °C and then concentrated under reduced pressure. Then, the content of the flask was solubilized in dry THF (0.5 M) to prepare the disulfide solution.

Preparation of the thioalkyne: In another flame-dried flask, 1-Hexyne (822 mg, 10 mmol, 1.00 equiv.) and *N,N,N',N*'-tetramethylethylenediamine (1.22 g, 10.5 mmol, 1.05 equiv.) were dissolved in dry THF (1 M). This solution was then cooled at 0 °C and a solution of *n*-BuLi 1.6 M in hexane was added dropwise (11 mmol, 1.10 eq, 6.88 mL). After stirring for 30 mins at 25 °C, the disulfide solution was added in dropwise with a syringe into this flask, and the new solution was stirred for additional 16 h. The reaction was stopped by the addition of 20 mL of H_2O and 10 mL of 1.0 M aqueous HCl. The formation of a white precipitate was observed. The organic phase was then extracted with a solution of Et_2O (3 x 20 mL) and dried over $MgSO_4$. The product was purified by column chromatography on silica gel (pentane/ethyl acetate 95:5), giving the **4n** with 87% of yield. All analytical data were in good accordance with those reported in the literature.[17]

5-(Methylthio)pent-4-yn-1-yl 2-(1-(4-chlorobenzoyl)-5-methoxy-2-methyl-1H-indol-3-yl)acetate (4o)

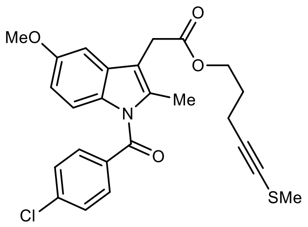

^{1}H NMR (400 MHz, CDCl$_3$) δ 7.66 (d, *J* = 8.5 Hz, 2H), 7.47 (d, *J* = 8.4 Hz, 2H), 6.96 (d, *J* = 2.5 Hz, 1H), 6.87 (d, *J* = 9.0 Hz, 1H), 6.67 (dd, *J* = 9.0, 2.5 Hz, 1H), 4.19 (t, *J* = 6.3 Hz, 2H), 3.84 (s, 3H), 3.66 (s, 2H), 2.38 (s, 3H), 2.36 – 2.30 (m, 5H), 1.82 (p, *J* = 6.7 Hz, 2H).

^{13}C NMR (100 MHz, CDCl$_3$) δ 170.7, 168.2, 156.0, 139.2, 135.8, 133.9, 131.1 (2C), 130.7, 130.6, 129.1 (2C), 114.9, 112.5, 111.7, 101.2, 91.2, 71.1, 63.6, 55.6, 30.3, 27.7, 19.1, 16.7, 13.3.

IR (neat) ν: 2924, 1738, 1591, 1478, 1399, 1356, 1314, 1221, 1165, 1142, 1089, 1067, 755, 530 cm^{-1}.

HRMS (ESI$^+$): exact mass calculated for $[M+Na]^+$ ($C_{25}H_{24}NO_4S^{35}ClNa^+$) requires m/z 492.1007, found m/z 492.1007.

3. Optimizations of hydrative amination

3.1 Optimizations of hydrative amination of ynamide

Supplementary Table 1 | Optimizations of ynamide amination [a]

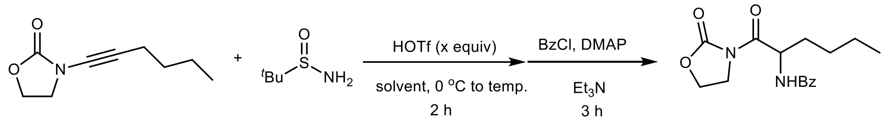

Entry	Solvent	HOTf (x equiv)	Temperature	Isolated yields
1	CH_2Cl_2	0.2	25 °C	trace
2	CH_2Cl_2	1.2	25 °C	70%
3	CH_2Cl_2	1.2	-78 °C	trace
4	DCE	1.2	25 °C	68%
5	toluene	1.2	25 °C	trace
6[b]	CH_2Cl_2	1.2	25 °C	66%
7[c]	CH_2Cl_2	1.2	25 °C	17%

[a] To a mixture of ynamide (33 mg, 0.20 mmol, 1.00 equiv.) in the given solvent (1.0 mL) in a flame-dried Schlenk tube under argon at 0 °C, triflic acid (x equiv.) was added dropwise. After stirring for 15 min at 0 °C, sulfinamide (48 mg, 0.4 mmol, 2.00 equiv.) in 1.0 mL of solvent was added and the reaction stirred for a further 5 min at 0 °C. After stirring for 2 h at the indicated temperature, triethylamine (83 µL, 0.6 mmol, 3.00 equiv.), 4-dimethylaminopyridine (1.2 mg, 0.04 mmol, 5 mol%) and benzoyl chloride (70 µL, 0.6 mmol, 5.00 equiv.) were added to the reaction. After stirring for another 3 h at 25 °C, the reaction was basified by the addition of a saturated aqueous solution of sodium bicarbonate (5.0 mL) and subsequently extracted twice with CH_2Cl_2 (5.0 mL). The combined organic layers were dried over anhydrous magnesium sulfate and the dried solution was filtered and subsequently concentrated under reduced pressure. The resulting crude product was purified by flash column chromatography on silica gel. [b] The reaction time was 24 h instead of 2 h. [c] Sulfinamide was added right after triflic acid was added.

3.2 Optimizations of hydrative amination of thioalkynes

Supplementary Table 2 | Optimizations of thioalkynes amination (I) [a]

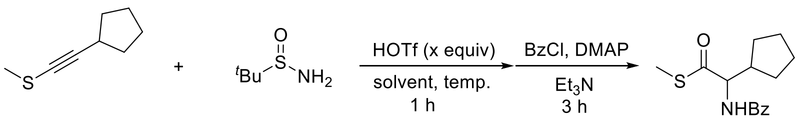

	Temperature	TfOH (x equiv.)	Solvent	Conc. [M]	Yield (%)
1	From 0°C to r.t.	1.0	CH_2Cl_2	0.1	57
2	0 °C	1.0	CH_2Cl_2	0.1	42
3	From 0°C to r.t.	0.6	CH_2Cl_2	0.1	43
4	From 0°C to r.t.	2.0	CH_2Cl_2	0.1	traces
5	From 0°C to r.t.	1.0	Toluene	0.1	traces
6	From 0°C to r.t.	1.0	Chloroform	0.1	50
7[b]	From 0°C to r.t.	1.0	CH_2Cl_2	0.1	42
8	From 0°C to r.t.	1.0	CH_2Cl_2	0.2	55
9	From 0°C to r.t.	1.0	CH_2Cl_2	0.06	57
10[c]	From 0°C to r.t.	1.0	CH_2Cl_2	0.1	73
11[d]	From 0°C to r.t.	1.0	CH_2Cl_2	0.1	73

[a] To a mixture of thioalkyne (0.20 mmol, 1.00 equiv.) in CH_2Cl_2 (0.2 M) in a flame-dried Schlenk tube under argon at 0 °C, triflic acid (x equiv.) was added dropwise. After stirring for 15 min at 0 °C, sulfinamide (48 mg, 0.4 mmol, 2.00 equiv.) in CH_2Cl_2 (0.4 M) was added and the reaction stirred for a further 5 min at 0 °C. After stirring for 1 h at 25 °C, triethylamine (83 µL, 0.6 mmol, 3.00 equiv.), 4-dimethylaminopyridine (1.2 mg, 0.04 mmol, 0.05 equiv.) and benzoyl chloride (70 µL, 0.6 mmol, 3.00 equiv.) were added to the reaction. After stirring for another 3 h at 25 °C, the reaction was basified by the addition of a saturated aqueous solution of sodium bicarbonate (5.0 mL) and subsequently extracted with CH_2Cl_2 (2 x 5.0 mL). The combined organic layers were dried over anhydrous magnesium sulfate and the dried solution was filtered and subsequently concentrated under reduced pressure. The resulting crude product was purified by flash column chromatography on silica gel (heptane/ethyl acetate) to obtain the final product. [b] 0.5 equiv. of sulfinamide **2**; [c] reaction time of 16 h; [d] reaction time of 24 h.

Supplementary Table 3 | Optimizations of thioalkynes amination (II) [a]

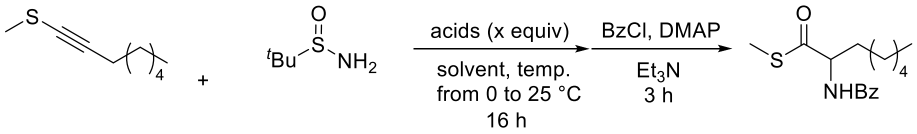

Entry	acids	x equiv.	solvent	Notes	yield (%)
1	HOTf	1.0	CH_2Cl_2	/	28
2	HOTf	0.6	CH_2Cl_2	/	28
3[b]	HOTf	1.0	CH_2Cl_2	**2** dropwise	19
4[c]	HOTf	1.0	CH_2Cl_2	reverse addition	0
5	HOTf	1.0	Chloroform	/	27
6	HOTf	1.0	DCE	/	28
7[d]	HOTf	1.0	CH_2Cl_2	portion-wise	40
8	Tf_2NH	1.0	CH_2Cl_2	/	44
9[d]	Tf_2NH	1.0	CH_2Cl_2	portion-wise	66
10[e]	Tf_2NH	1.0	CH_2Cl_2	portion-wise	71

[a] To a mixture of thioalkyne (0.20 mmol, 1.00 equiv.) in CH_2Cl_2 (0.2 M) in a flame-dried Schlenk tube under argon at 0 °C, triflic acid (17 μL, 0.20 mmol, 1.00 equiv.) was added dropwise. After stirring for 15 min at 0 °C, sulfinamide **2** (48 mg, 0.4 mmol, 2.00 equiv.) in CH_2Cl_2 (0.4 M) was added and the reaction stirred for a further 5 min at 0 °C. The reaction mixture was stirred for 16 h before the protection step. After stirring for 16 h at 25 °C, triethylamine (83 μL, 0.6 mmol, 3.00 equiv.), 4-dimethylaminopyridine (1.2 mg, 0.04 mmol, 0.05 equiv.) and benzoyl chloride (70 μL, 0.6 mmol, 3.00 equiv.) were added to the reaction. After stirring for another 3 h at 25 °C, the reaction was basified by the addition of a saturated aqueous solution of sodium bicarbonate (5.0 mL) and subsequently extracted with CH_2Cl_2 (2 x 5.0 mL). The combined organic layers were dried over anhydrous magnesium sulfate and the dried solution was filtered and subsequently concentrated under reduced pressure. The resulting crude product was purified by flash column chromatography on silica gel (heptane/ethyl acetate) to obtain the final product. [b] To a solution of preactivated thioalkynes, a solution of sulfinamide **2** in CH_2Cl_2 (0.2 M) was added in dropwise over 1 h; [c] to a solution of **2** in CH_2Cl_2 (0.2 M), a solution of pre-activated thioalkyne (1 equiv. of thioalkyne and 1.00 equiv. of HOTf) in CH_2Cl_2 (0.1 M) was added in dropwise over 1 h; [d] to a solution of thioalkyne in at 0 °C CH_2Cl_2 (0.3 M), a solution of 0.60 equiv. of acid in CH_2Cl_2 (0.3 M). After 15 mins, 1.20 equiv. of **2** in CH_2Cl_2 (0.6 M) were added and the reaction mixture was let it warm to 25°C. After 1 h, the solution was brought back to 0 °C and 0.40 equiv. of acid in CH_2Cl_2 (0.25 M) were added. After 15 mins, 0.80 equiv. of **2** in CH_2Cl_2 (0.5 M) were added. Then, the reaction was stirred at 25 °C for 16 h. [e] to a solution of thioalkyne in at 0°C CH_2Cl_2 (0.3 M), a solution of 0.60 equiv. of acid in CH_2Cl_2 (0.3 M). After 15 mins 1.20 equiv. of **2** in CH_2Cl_2 (0.6 M) were added and the reaction mixture was let it warm to 25 °C. After 1 h, the solution was brought back to 0 °C and 0.40 equiv. of acid in CH_2Cl_2 (0.25 M) were added. After 5 mins, 0.80 equiv. of **2** in CH_2Cl_2 (0.5 M) were added. Then, the reaction was stirred at 25 °C for 16 h.

4. Synthesis of α-amino amides and α-amino thioesters *via* hydrative amination

4.1 Hydrative amination of ynamides

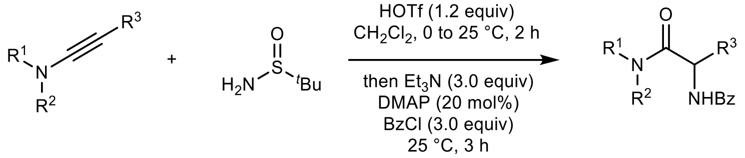

General procedure 3: To a flame-dried Schlenk tube, the CH_2Cl_2 (1.0 mL) solution of ynamide (0.20 mmol) was added under Ar atmosphere. Then the solution was cooled down to 0 °C before HOTf (21 µL, 0.24 mmol, 1.20 equiv.) was added in dropwise. The reaction mixture was then stirred for 15 min at the same temperature. A solution of 2-methylpropane-2-sulphinamide (48.5 mg, 0.40 mmol, 2.00 equiv.) in CH_2Cl_2 (1.0 mL) was then added to the reaction in one portion before it's allowed to stirred at room temperature for 2 h. Triethylamine (84 µL, 0.60 mmol, 3.00 equiv.), 4-dimethylaminopyridine (DMAP, 5.0 mg, 0.04 mmol, 0.20 equiv.) and benzoyl chloride (70 µL, 0.60 mmol, 3.00 equiv.) were then added in sequence. The reaction was stirred at room temperature for another 3 h before the addition of a saturated aqueous solution of sodium bicarbonate. The combined organic layers were dried over anhydrous magnesium sulfate, the dried solution was filtered and the filtrate was concentrated under reduced pressure to afford the crude product. The crude product was then purified by column to give the desired aminated amide.

4.2 Hydrative amination of thioalkynes

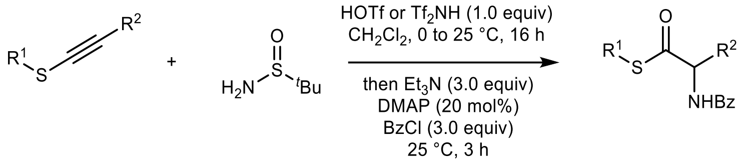

General procedure 4: To a mixture of thioalkyne (0.20 mmol, 1.00 equiv.) in CH_2Cl_2 (0.2 M) in a flame-dried Schlenk tube under argon at 0 °C, HOTf (17 µL, 0.20 mmol, 1.00 equiv.) was added dropwise. After stirring for 15 min at 0 °C, sulfinamide (48 mg, 0.4 mmol, 2.00 equiv.) in CH_2Cl_2 (0.4 M) was added and the reaction stirred for a further 5 min at 0 °C. After stirring for 16 h at 25 °C, triethylamine (83 µL, 0.60 mmol, 3.00 equiv.), 4-dimethylaminopyridine (1.2 mg, 0.04 mmol, 0.05 equiv.) and benzoyl chloride (70 µL, 0.60 mmol, 3.00 equiv.) were added to the reaction. After stirring for another 3 h at 25 °C, the reaction was basified by the addition of a saturated aqueous solution of sodium bicarbonate (5.0 mL) and subsequently extracted with CH_2Cl_2 (2 x 5.0 mL). The combined organic layers were dried over anhydrous magnesium sulfate and the dried solution was filtered and subsequently concentrated under reduced pressure. The resulting crude product was purified by flash column chromatography on silica gel (heptane/ethyl acetate) to obtain the final product.

General procedure 5: To a mixture of thioalkyne (0.20 mmol, 1.00 equiv.) in CH_2Cl_2 (0.3 M) in a flame-dried Schlenk tube under argon at 0 °C, Tf_2NH (34.0 mg, 0.12 mmol, 0.60 equiv.) in CH_2Cl_2 (0.3 M) was added dropwise. After stirring for 15 min at 0 °C, sulfinamide (29 mg, 0.24 mmol, 1.20 equiv.) in CH_2Cl_2 (0.6 M) was added and the reaction stirred for a further 5 min at 0 °C. After stirring for 1 h at 25 °C, the

solution was cooled to 0 °C and stirred for 5 min. Then, Tf_2NH (23.0 mg, 0.08 mmol, 0.4 equiv.) in CH_2Cl_2 (0.25 M) was added dropwise. After stirring for 5 min at 0 °C, sulfinamide (19 mg, 0.16 mmol, 0.8 equiv.) in CH_2Cl_2 (0.5) was added and the reaction stirred for a further 5 min at 0 °C. After stirring for 16 h at 25°C, triethylamine (83 µL, 0.6 mmol, 3.00 equiv.), 4-dimethylaminopyridine (1.2 mg, 0.04 mmol, 0.05 equiv.) and benzoyl chloride (70 µL, 0.6 mmol, 3.00 equiv.) were added to the reaction. After stirring for another 3 h at 25 °C, the reaction was basified by the addition of a saturated aqueous solution of sodium bicarbonate (5.0 mL) and subsequently extracted with CH_2Cl_2 (2 x 5.0 mL). The combined organic layers were dried over anhydrous magnesium sulfate and the dried solution was filtered and subsequently concentrated under reduced pressure. The resulting crude product was purified by flash column chromatography on silica gel (heptane/ethyl acetate) to obtain the final product.

4.3 Characterizations of the α-amino amides

***N*-(1-oxo-1-(2-oxooxazolidin-3-yl)hexan-2-yl)benzamide (3a)**

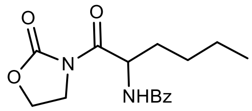

Prepared according to the General Procedure 3 in 70% yield as a white solid.

^{1}H NMR (600 MHz, CDCl₃) δ 7.87 – 7.76 (m, 2H), 7.57 – 7.49 (m, 1H), 7.44 (dd, *J* = 10.6, 4.7 Hz, 2H), 6.73 (d, *J* = 7.9 Hz, 1H), 5.83 (td, *J* = 8.7, 3.9 Hz, 1H), 4.52 – 4.42 (m, 2H), 4.10 (ddd, *J* = 10.9, 8.7, 7.4 Hz, 1H), 4.05 – 3.95 (m, 1H), 2.05 – 1.93 (m, 1H), 1.74 – 1.65 (m, 1H), 1.53 – 1.32 (m, 4H), 0.90 (t, *J* = 7.2 Hz, 3H).

^{13}C NMR (150 MHz, $CDCl_3$) δ 173.3, 167.4, 152.3, 134.1, 131.9, 128.7 (2C), 127.2 (2C), 62.6, 52.4, 42.7, 32.4, 27.9, 22.5, 14.0.

IR (neat) v: 1784, 1708, 1657, 1534, 1528, 1488, 1390, 1364, 1265, 1224, 1113, 1042, 760, 715 cm^{-1}.

HRMS (ESI$^+$): exact mass calculated for $[M+Na]^+$ ($C_{16}H_{20}N_2O_4Na^+$) requires m/z 327.1315, found m/z 327.1314.

***N*-(4-methyl-1-oxo-1-(2-oxooxazolidin-3-yl)pentan-2-yl)benzamide (3b)**

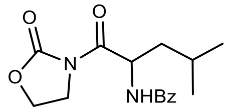

Prepared according to the General Procedure 3 in 59% yield as a white solid.

^{1}H NMR (400 MHz, $CDCl_3$) δ 7.83 – 7.78 (m, 2H), 7.54 – 7.48 (m, 1H), 7.48 – 7.39 (m, 2H), 6.69 (d, *J* = 8.4 Hz, 1H), 5.90 (ddd, *J* = 11.5, 8.6, 3.2 Hz, 1H), 4.46 (ddd, *J* = 8.7, 7.3, 1.4 Hz, 2H), 4.09 (ddd, *J* = 10.9, 8.8, 7.2 Hz, 1H), 4.04 – 3.93 (m, 1H), 1.90 – 1.78 (m, 1H), 1.78 – 1.70 (m, 1H), 1.65 – 1.57 (m, 1H), 1.06 (d, *J* = 6.4 Hz, 3H), 0.97 (d, *J* = 6.5 Hz, 3H).

^{13}C NMR (101 MHz, CDCl$_3$) δ 173.6, 167.3, 152.8, 133.9, 131.7, 128.6 (2C), 127.1 (2C), 62.4, 51.0, 42.6, 41.2, 25.3, 23.5, 21.1.

IR (neat) ν: 1784, 1710, 1641, 1535, 1488, 1390, 1284, 1260, 1226, 1170, 1041, 717, 692 cm^{-1}.

HRMS (ESI$^+$): exact mass calculated for $[M+Na]^+$ ($C_{16}H_{20}N_2O_4Na^+$) requires m/z 327.1315, found m/z 327.1318.

***N*-(5-Methyl-1-oxo-1-(2-oxooxazolidin-3-yl)hexan-2-yl)benzamide (3c)**

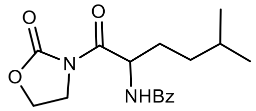

Prepared according to the General Procedure 3 in 63% yield as a white solid.

^{1}H NMR (700 MHz, CDCl$_3$) δ 7.82 – 7.79 (m, 2H), 7.51 (ddd, *J* = 6.9, 4.0, 1.2 Hz, 1H), 7.47 – 7.42 (m, 2H), 6.72 (d, *J* = 7.9 Hz, 1H), 5.81 (td, *J* = 8.6, 3.9 Hz, 1H), 4.49 – 4.46 (m, 2H), 4.13 – 4.07 (m, 1H), 4.04 – 3.98 (m, 1H), 2.04 – 1.97 (m, 1H), 1.71 – 1.64 (m, 1H), 1.64 – 1.53 (m, 1H), 1.46 – 1.37 (m, 1H), 1.36 – 1.28 (m, 1H), 0.89 (dd, *J* = 6.6, 2.1 Hz, 6H).

^{13}C NMR (175 MHz, CDCl$_3$) δ 173.3, 167.4, 153.0, 134.1, 131.9, 128.7 (2C), 127.2 (2C), 62.6, 52.6, 42.7, 34.7, 30.7, 28.0, 22.7, 22.5.

IR (neat) ν: 3340, 2955, 2925, 1777, 1704, 1643, 1529, 1388, 1266, 1222, 715 cm^{-1}.

HRMS (ESI$^+$): exact mass calculated for $[M+Na]^+$ ($C_{17}H_{22}N_2O_4Na^+$) requires m/z 341.1472, found 341.1473 m/z.

***N*-(3-cyclohexyl-1-oxo-1-(2-oxooxazolidin-3-yl)propan-2-yl)benzamide (3d)**

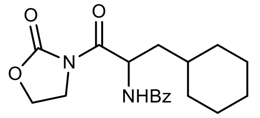

Prepared according to the General Procedure 3 in 74% yield as a white solid.

^{1}H NMR (600 MHz, CDCl$_3$) δ 7.81 (d, *J* = 7.7 Hz, 2H), 7.52 (t, *J* = 7.3 Hz, 1H), 7.45 (t, *J* = 7.6 Hz, 2H), 6.64 (d, *J* = 8.1 Hz, 1H), 5.98 – 5.82 (m, 1H), 4.46 (dd, *J* = 12.2, 5.5 Hz, 2H), 4.11 – 3.97 (m, 2H), 2.02 (d, *J* = 12.9 Hz, 1H), 1.84 – 1.76 (m, 1H), 1.76 – 1.62 (m, 4H), 1.60 – 1.54 (m, 1H), 1.53 – 1.46 (m, 1H), 1.28 – 1.14 (m, 3H), 1.06 – 0.97 (m, 2H).

^{13}C NMR (150 MHz, CDCl$_3$) δ 173.8, 167.3, 152.8, 133.9, 131.7, 128.6 (2C), 127.1 (2C), 62.5, 50.4, 42.6, 39.8, 34.5, 34.1, 31.7, 26.3, 26.3, 25.9.

IR (neat) ν: 1785, 1706, 1640, 1527, 1448, 1264, 1160, 1115, 1042, 758, 692, 668 cm^{-1}.

HRMS (ESI+): exact mass calculated for $[M+Na]^+$ ($C_{19}H_{24}N_2O_4Na^+$) requires m/z 367.1628, found m/z 367.1626.

***N*-(5-chloro-1-oxo-1-(2-oxooxazolidin-3-yl)pentan-2-yl)benzamide (3e)**

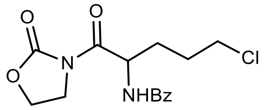

Prepared according to General Procedure 3 in 61% yield as a white solid.

^{1}H NMR (600 MHz, CDCl$_3$) δ 7.81 (dt, *J* = 8.5, 1.6 Hz, 2H), 7.55 – 7.50 (m, 1H), 7.47 – 7.43 (m, 2H), 6.83 (d, *J* = 8.2 Hz, 1H), 5.86 (td, *J* = 9.2, 3.4 Hz, 1H), 4.49 (t, *J* = 8.1 Hz, 2H), 4.11 (ddd, *J* = 16.2, 8.8, 6.2 Hz, 1H), 4.03 (dt, *J* = 11.0, 8.1 Hz, 1H), 3.69 – 3.55 (m, 2H), 2.15 (dddd, *J* = 13.4, 9.6, 5.9, 3.5 Hz, 1H), 2.10 – 2.02 (m, 1H), 2.00 – 1.91 (m, 1H), 1.87 (dtd, *J* = 14.3, 9.5, 4.9 Hz, 1H).

^{13}C NMR (151 MHz, CDCl$_3$) δ 172.7, 167.5, 153.0, 133.8, 132.1, 128.8 (2C), 127.3 (2C), 62.7, 51.8, 44.6, 42.7, 30.4, 29.1.

IR (neat) ν: 3328, 2923, 1773, 1703, 1643, 1524, 1481, 1387, 1263, 1220, 712 cm^{-1}.

HRMS (ESI+): exact mass calculated for $[M+Na]^+$ ($C_{15}H_{17}N_2O_4ClNa^+$) requires m/z 347.0770, found 347.0768 m/z.

***N*-(5-(1,3-dioxoisoindolin-2-yl)-1-oxo-1-(2-oxooxazolidin-3-yl)pentan-2-yl)benzamide (3f)**

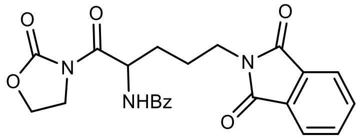

Prepared according to General Procedure 3 in 78% yield as a white solid.

^{1}H NMR (600 MHz, CDCl$_3$) δ 7.80 (dt, *J* = 7.1, 6.6 Hz, 4H), 7.72 – 7.66 (m, 2H), 7.48 (t, *J* = 7.4 Hz, 1H), 7.40 (t, *J* = 7.6 Hz, 2H), 7.08 (d, *J* = 7.9 Hz, 1H), 5.82 (td, *J* = 8.5, 3.8 Hz, 1H), 4.50 – 4.42 (m, 2H), 4.10 – 4.04 (m, 1H), 4.04 – 3.97 (m, 1H), 3.77 – 3.68 (m, 2H), 2.04 (ddd, *J* = 14.1, 9.8, 5.0 Hz, 1H), 1.95 – 1.85 (m, 2H), 1.80 – 1.72 (m, 1H).

^{13}C NMR (150 MHz, CDCl$_3$) δ 172.6, 168.4 (2C), 167.3, 152.9, 134.0 (2C), 133.6, 131.9, 131.7 (2C), 128.5 (2C), 127.2 (2C), 123.2 (2C), 62.5, 52.1, 42.5, 37.5, 29.3, 25.2.

IR (neat) ν: 1771, 1707, 1658, 1526, 1436, 1361, 1268, 1227, 1188, 1116, 1042, 1011, 758, 718, 530 cm^{-1}.

HRMS (ESI+): exact mass calculated for $[M+Na]^+$ ($C_{23}H_{21}N_3O_6Na^+$) requires m/z 458.1323, found m/z 458.1323.

***N*-(1-cyclopentyl-2-oxo-2-(2-oxooxazolidin-3-yl)ethyl)benzamide (3g)**

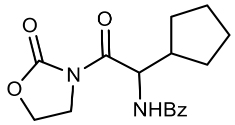

Prepared according to General Procedure 3 in 44% yield as a white solid.

^{1}H NMR (600 MHz, $CDCl_3$) δ 7.79 (d, *J* = 7.6 Hz, 2H), 7.50 (t, *J* = 7.4 Hz, 1H), 7.43 (t, *J* = 7.7 Hz, 2H), 6.84 (d, *J* = 6.9 Hz, 1H), 5.93 (t, *J* = 7.1 Hz, 1H), 4.50 – 4.40 (m, 2H), 4.16 – 4.05 (m, 1H), 4.05 – 3.96 (m, 1H), 2.45 (dd, *J* = 15.3, 8.0 Hz, 1H), 1.86 – 1.80 (m, 1H), 1.72 – 1.61 (m, 3H), 1.61 – 1.52 (m, 2H), 1.52 – 1.37 (m, 2H).

^{13}C NMR (150 MHz, $CDCl_3$) δ 172.9, 167.3, 153.0, 134.0, 131.8, 128.6 (2C), 127.1 (2C), 62.4, 54.2, 42.7, 42.1, 29.0, 27.8, 25.2, 25.1.

IR (neat) ν: 2957, 2869, 1776, 1701, 1658, 1519, 1485, 1389, 1264, 1219, 1117, 1041, 759, 714 cm^{-1}.

HRMS (ESI$^+$): exact mass calculated for $[M+Na]^+$ ($C_{17}H_{20}N_2O_4Na^+$) requires m/z 339.1315, found m/z 339.1318.

***N*-(3,3-dimethyl-1-oxo-1-(2-oxooxazolidin-3-yl)butan-2-yl)benzamide (3h)**

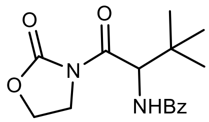

Prepared according to General Procedure 3 in 50% yield as a white solid.

^{1}H NMR (600 MHz, $CDCl_3$) δ 7.80 – 7.77 (m, 2H), 7.50 (dd, *J* = 10.6, 4.2 Hz, 1H), 7.43 (t, *J* = 7.6 Hz, 2H), 6.71 (d, *J* = 8.5 Hz, 1H), 6.21 (d, *J* = 8.9 Hz, 1H), 4.47 – 4.37 (m, 2H), 4.05 (dddd, *J* = 15.8, 11.1, 9.2, 7.0 Hz, 2H), 1.10 (s, 9H).

^{13}C NMR (151 MHz, $CDCl_3$) δ 172.7, 167.3, 153.1, 134.1, 131.9, 128.8 (2C), 127.2 (2C), 62.0, 56.5, 42.7, 35.6, 26.4 (3C).

IR (neat) ν: 3364, 2961, 2923, 1774, 1696, 1658, 1514, 1481, 1381, 1216, 1113, 709 cm^{-1}.

HRMS (ESI$^+$): exact mass calculated for $[M+Na]^+$ ($C_{16}H_{20}N_2O_4Na^+$) requires m/z 327.1315, found 327.1315 m/z.

***N*-(1-(cyclohex-1-en-1-yl)-2-oxo-2-(2-oxooxazolidin-3-yl)ethyl)benzamide (3i)**

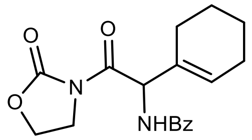

Prepared according to General Procedure 3 in 46% yield as a white solid.

^{1}H NMR (600 MHz, CDCl$_3$) δ 7.92 – 7.76 (m, 2H), 7.60 – 7.50 (m, 1H), 7.50 – 7.41 (m, 2H), 6.64 (d, *J* = 8.1 Hz, 1H), 6.22 (d, *J* = 8.2 Hz, 1H), 5.71 (d, *J* = 3.6 Hz, 1H), 4.52 – 4.43 (m, 2H), 4.13 (ddd, *J* = 11.0, 8.7, 7.3 Hz, 1H), 4.08 – 3.97 (m, 1H), 2.38 – 2.25 (m, 1H), 2.22 – 2.10 (m, 1H), 2.10 – 2.02 (m, 2H), 1.72 (dd, *J* = 9.8, 5.5 Hz, 2H), 1.59 (dd, *J* = 10.4, 4.9 Hz, 2H).

^{13}C NMR (150 MHz, CDCl$_3$) δ 170.8, 167.3, 152.6, 134.3, 133.8, 131.7, 128.5 (2C), 127.1 (2C), 126.6, 62.3, 57.0, 42.5, 26.8, 25.2, 22.6, 21.7.

IR (neat) ν: 2930, 1778, 1706, 1642, 1579, 1483, 1388, 1263, 1226, 1156, 1114, 1041, 758, 716 cm^{-1}.

HRMS (ESI$^+$): exact mass calculated for [M+Na]$^+$ ($C_{18}H_{20}N_2O_4Na^+$) requires m/z 351.1315, found m/z 351.1320.

***N*-(2-oxo-2-(2-oxooxazolidin-3-yl)-1-phenylethyl)benzamide (3j)**

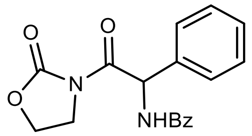

Prepared according to General Procedure 3 in 77% yield as a white solid.

^{1}H NMR (600 MHz, CDCl$_3$) δ 7.81 – 7.76 (m, 2H), 7.55 (dd, *J* = 8.0, 1.2 Hz, 2H), 7.49 (t, *J* = 7.4 Hz, 1H), 7.44 – 7.34 (m, 5H), 7.04 (d, *J* = 6.6 Hz, 1H), 6.95 (d, *J* = 7.0 Hz, 1H), 4.43 (td, *J* = 9.2, 6.4 Hz, 1H), 4.33 (td, *J* = 9.1, 7.2 Hz, 1H), 4.16-4.12 (m, 1H), 3.93-3.98 (m, 1H).

^{13}C NMR (150 MHz, CDCl$_3$) δ 170.8, 166.6, 152.5, 135.4, 133.5, 131.8, 129.1 (2C), 128.9, 128.6 (2C), 128.5 (2C), 127.1 (2C), 62.2, 55.8, 42.6.

IR (neat) ν: 1746, 1642, 1556, 1521, 1484, 1434, 1389, 1222, 716, 698 cm^{-1}.

HRMS (ESI$^+$): exact mass calculated for [M+Na]$^+$ ($C_{18}H_{16}N_2O_4Na^+$) requires m/z 347.1002, found m/z 347.1006.

N-(2-oxo-2-(2-oxooxazolidin-3-yl)-1-(p-tolyl)ethyl)benzamide (3k)

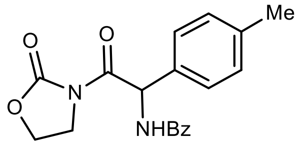

Prepared according to General Procedure 3 in 64% yield as a white solid.

^{1}H NMR (600 MHz, $CDCl_3$) δ 7.77 (d, J = 7.6 Hz, 2H), 7.48 (t, J = 7.4 Hz, 1H), 7.46 – 7.36 (m, 4H), 7.18 (d, J = 7.9 Hz, 2H), 7.02 (d, J = 6.7 Hz, 1H), 6.90 (d, J = 6.9 Hz, 1H), 4.43-4.38 (m, 1H), 4.33-4.29 (m, 1H), 4.16 – 4.07 (m, 1H), 3.96 – 3.89 (m, 1H), 2.33 (s, 3H).

^{13}C NMR (150 MHz, $CDCl_3$) δ 170.8, 166.5, 152.4, 138.8, 133.5, 132.4, 131.7, 129.7 (2C), 128.5 (2C), 128.4 (2C), 127.1 (2C), 62.2, 55.5, 42.6, 21.1

IR (neat) ν: 1777, 1706, 1658, 1512, 1482, 1389, 1368, 1264, 1222, 1185, 1117, 1042, 757, 719, 711 cm^{-1}.

HRMS (ESI$^+$): exact mass calculated for $[M+Na]^+$ ($C_{19}H_{18}N_2O_4Na^+$) requires m/z 361.1159, found m/z 361.1157.

N-(1-(4-methoxyphenyl)-2-oxo-2-(2-oxooxazolidin-3-yl)ethyl)benzamide (3l)

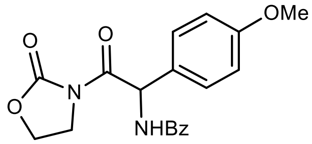

Prepared according to the General Procedure in 58% yield as a white solid.

^{1}H NMR (600 MHz, $CDCl_3$) δ 7.81 – 7.75 (m, 2H), 7.50 – 7.44 (m, 3H), 7.40 (td, J = 8.4, 1.6 Hz, 2H), 7.02 (d, J = 6.7 Hz, 1H), 6.88 (t, J = 7.3 Hz, 3H), 4.41 (td, J = 9.2, 6.5 Hz, 1H), 4.32 (td, J = 9.1, 7.0 Hz, 1H), 4.12 (ddd, J = 10.8, 9.5, 7.0 Hz, 1H), 3.93 (ddd, J = 10.9, 9.3, 6.5 Hz, 1H), 3.78 (s, 3H).

^{13}C NMR (150 MHz, $CDCl_3$) δ 170.9, 166.6, 159.9, 152.5, 133.4, 131.7, 129.9 (2C), 128.4 (2C), 127.3, 127.1 (2C), 114.3 (2C), 62.2, 55.2, 55.1, 42.5.

IR (neat) ν: 1776, 1706, 1663, 1641, 1510, 1484, 1389, 1366, 1263, 1222, 1179, 1041, 716, 579, 531 cm^{-1}.

HRMS (ESI$^+$): exact mass calculated for $[M+Na]^+$ ($C_{19}H_{18}N_2O_5Na^+$) requires m/z 377.1108, found m/z 377.1106.

***N*-(1-([1,1'-biphenyl]-4-yl)-2-oxo-2-(2-oxooxazolidin-3-yl)ethyl)benzamide (3m)**

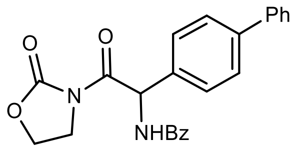

Prepared according to General Procedure 3 in 55% yield as a white solid.

^{1}H NMR (400 MHz, CDCl$_3$) δ 7.81 (dd, *J* = 5.6, 3.3 Hz, 2H), 7.66 – 7.54 (m, 5H), 7.50 (dd, *J* = 11.7, 4.5 Hz, 1H), 7.43 (q, *J* = 7.0 Hz, 5H), 7.35 (t, *J* = 7.3 Hz, 1H), 7.14 (d, *J* = 6.8 Hz, 1H), 7.00 (d, *J* = 6.9 Hz, 1H), 4.50 – 4.29 (m, 2H), 4.24 – 4.08 (m, 1H), 3.97 (ddd, *J* = 10.8, 9.4, 6.5 Hz, 1H).

^{13}C NMR (100 MHz, CDCl$_3$) δ 170.8, 166.6, 152.5, 141.8, 140.3, 134.3, 133.4, 131.8, 129.0 (2C), 128.8 (2C), 128.5 (2C), 127.7 (2C), 127.6, 127.1 (2C), 127.0 (2C), 62.3, 55.5, 42.6.

IR (neat) ν: 1779, 1707, 1662, 1519, 1485, 1389, 1366, 1223, 1183, 1119, 1041, 756, 734, 696, 560 cm^{-1}.

HRMS (ESI$^+$): exact mass calculated for $[M+Na]^+$ ($C_{24}H_{20}N_2O_4Na^+$) requires m/z 423.1315, found m/z 423.1312.

***N*-(2-oxo-2-(2-oxooxazolidin-3-yl)-1-(m-tolyl)ethyl)benzamide (3n)**

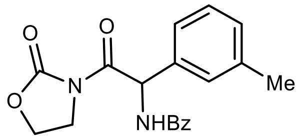

Prepared according to General Procedure 3 in 72% yield as a white solid.

^{1}H NMR (400 MHz, CDCl$_3$) δ 7.78 (dd, *J* = 5.2, 3.3 Hz, 2H), 7.53 – 7.46 (m, 1H), 7.44 – 7.38 (m, 2H), 7.34 (d, *J* = 12.2 Hz, 2H), 7.29 – 7.24 (m, 1H), 7.16 (d, *J* = 7.5 Hz, 1H), 6.99 (d, *J* = 6.8 Hz, 1H), 6.91 (d, *J* = 6.9 Hz, 1H), 4.43 (td, *J* = 9.2, 6.4 Hz, 1H), 4.34 (td, *J* = 9.1, 7.1 Hz, 1H), 4.14 (ddd, *J* = 10.8, 9.5, 7.1 Hz, 1H), 3.96 (ddd, *J* = 10.9, 9.3, 6.4 Hz, 1H), 2.36 (s, 3H).

^{13}C NMR (100 MHz, CDCl$_3$) δ 170.8, 166.6, 152.5, 138.9, 135.2, 133.5, 131.8, 129.7, 129.2, 128.9, 128.5 (2C), 127.1 (2C), 125.6, 62.2, 55.7, 42.6, 21.4.

IR (neat) ν: 1776, 1706, 1658, 1512, 1483, 1389, 1365, 1266, 1222, 1118, 1041, 715, 692 cm^{-1}.

HRMS (ESI$^+$): exact mass calculated for $[M+Na]^+$ ($C_{19}H_{18}N_2O_4Na^+$) requires m/z 361.1159, found m/z 361.1159.

N-(1-(4-fluorophenyl)-2-oxo-2-(2-oxooxazolidin-3-yl)ethyl)benzamide (3o)

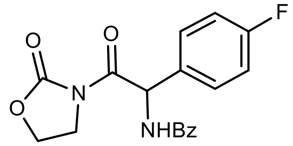

Prepared according to the General Procedure in 64% yield as a white solid.

^{1}H NMR (400 MHz, $CDCl_3$) δ 7.77 (dd, *J* = 5.2, 3.3 Hz, 2H), 7.57 – 7.46 (m, 3H), 7.45 – 7.38 (m, 2H), 7.16 – 6.99 (m, 3H), 6.92 (d, *J* = 6.9 Hz, 1H), 4.44 (td, *J* = 9.2, 6.5 Hz, 1H), 4.35 (td, *J* = 9.1, 7.0 Hz, 1H), 4.14 (ddd, *J* = 10.9, 9.5, 7.0 Hz, 1H), 3.96 (ddd, *J* = 10.9, 9.3, 6.5 Hz, 1H).

^{13}C NMR (100 MHz, $CDCl_3$) δ 170.7, 166.5, 162.9 (d, J^1_{C-F} = 247.0 Hz), 152.4, 133.4, 131.9, 131.4 (J^4_{C-F} = 4.0 Hz), 130.5 (J^3_{C-F} = 8.0 Hz, 2C), 128.4 (2C), 127.1 (2C), 116.0 (J^2_{C-F} = 21.0 Hz, 2C), 62.3, 55.0, 42.6.

IR (neat) ν: 1776, 1706, 1640, 1528, 1483, 1389, 1265, 1222, 1041, 833, 800, 758, 711, 573, 478 cm^{-1}.

HRMS (ESI$^+$): exact mass calculated for $[M+Na]^+$ ($C_{18}H_{15}N_2O_4FNa^+$) requires m/z 365.0908, found m/z 365.0907.

N-(1-(4-chlorophenyl)-2-oxo-2-(2-oxooxazolidin-3-yl)ethyl)benzamide (3p)

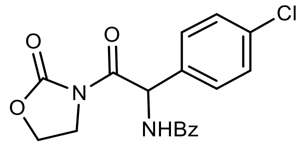

Prepared according to the General Procedure in 67% yield as a white solid.

^{1}H NMR (400 MHz, $CDCl_3$) δ 7.81 – 7.73 (m, 2H), 7.53 – 7.46 (m, 3H), 7.41 (dd, *J* = 10.3, 4.7 Hz, 2H), 7.38 – 7.31 (m, 2H), 7.13 (d, *J* = 6.8 Hz, 1H), 6.91 (d, *J* = 7.0 Hz, 1H), 4.48-4.33 (m, 2H), 4.14 (ddd, *J* = 10.8, 9.5, 7.1 Hz, 1H), 3.96 (ddd, *J* = 10.9, 9.3, 6.5 Hz, 1H).

^{13}C NMR (100 MHz, $CDCl_3$) δ 170.5, 166.5, 152.4, 134.9, 134.1, 133.3, 131.9, 130.0 (2C), 129.2 (2C), 128.6 (2C), 127.1 (2C), 62.3, 55.1, 42.6.

IR (neat) ν: 1773, 1703, 1639, 1512, 1485, 1388, 1360, 1221, 1119, 1091, 1014, 711, 565 cm^{-1}.

HRMS (ESI$^+$): exact mass calculated for $[M+Na]^+$ ($C_{18}H_{15}N_2O_4{}^{35}ClNa^+$) requires m/z 381.0613, found m/z 381.0615.

***N*-(2-oxo-2-(2-oxooxazolidin-3-yl)-1-(4-(trifluoromethyl)phenyl)ethyl)benzamide (3q)**

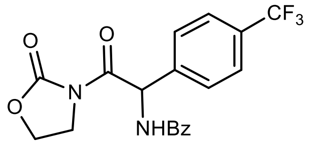

Prepared according to General Procedure 3 in 66% yield as a white solid.

^{1}H NMR (400 MHz, CDCl$_3$) δ 7.83 – 7.77 (m, 2H), 7.70 (d, *J* = 8.3 Hz, 2H), 7.64 (d, *J* = 8.4 Hz, 2H), 7.58 – 7.50 (m, 1H), 7.44 (dd, *J* = 10.4, 4.6 Hz, 2H), 7.27 (d, *J* = 8.8 Hz, 1H), 7.03 (d, *J* = 7.1 Hz, 1H), 4.44 (dtd, *J* = 35.0, 9.1, 6.8 Hz, 2H), 4.23 – 4.11 (m, 1H), 4.00 (ddd, *J* = 10.9, 9.3, 6.4 Hz, 1H).

^{13}C NMR (150 MHz, CDCl$_3$) δ 170.2, 166.5, 152.4, 139.6, 133.2, 133.0, 130.9 (q, $^{2}J_{C-F}$ = 32.6 Hz, 2C), 129.0, 128.6 (2C), 127.1 (2C), 125.9 (q, $^{3}J_{C-F}$ = 3.7 Hz, 2C), 123.8 (q, $^{1}J_{C-F}$ = 270 Hz), 62.4, 55.4, 42.6.

^{19}F NMR (400 MHz, CDCl$_3$) δ -62.8.

IR (neat) ν: 1777, 1704, 1662, 1642, 1511, 1481, 1389, 1324, 1267, 1223, 1112, 1067, 954, 756, 669 cm^{-1}.

HRMS (ESI$^+$): exact mass calculated for [M+Na]$^+$ ($C_{19}H_{15}N_2O_4F_3Na^+$) requires m/z 415.0876, found m/z 415.0883.

***N*-(1-(3-chlorophenyl)-2-oxo-2-(2-oxooxazolidin-3-yl)ethyl)benzamide (3r)**

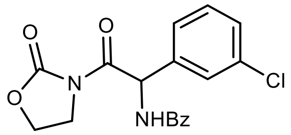

Prepared according to General Procedure 3 in 66% yield as a white solid.

^{1}H NMR (600 MHz, CDCl$_3$) δ 7.83 – 7.73 (m, 2H), 7.54 – 7.39 (m, 5H), 7.35 – 7.27 (m, 2H), 7.15 (d, *J* = 7.0 Hz, 1H), 6.93 (d, *J* = 7.1 Hz, 1H), 4.48-4.34 (m, 2H), 4.14 (ddd, *J* = 10.9, 9.5, 7.1 Hz, 1H), 3.98 (ddd, *J* = 10.9, 9.3, 6.5 Hz, 1H).

^{13}C NMR (150 MHz, CDCl$_3$) δ 170.3, 166.5, 152.4, 137.4, 134.7, 133.2, 131.9, 130.3, 129.1, 128.6 (2C), 128.3, 127.2, 127.1 (2C), 62.4, 55.2, 42.6.

IR (neat) ν: 1774, 1703, 1662, 1511, 1496, 1388, 1367, 1265, 1188, 1118, 1041, 787, 714, 668, 545 cm^{-1}.

HRMS (ESI$^+$): exact mass calculated for [M+Na]$^+$ ($C_{18}H_{15}N_2O_4{}^{35}ClNa^+$) requires m/z 381.0613, found m/z 381.0614.

***N*-(1-(2-chlorophenyl)-2-oxo-2-(2-oxooxazolidin-3-yl)ethyl)benzamide (3s)**

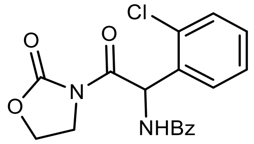

Prepared according to General Procedure 3 in 81% yield as a white solid.

^{1}H NMR (400 MHz, CDCl$_3$) δ 7.74 – 7.67 (m, 3H), 7.48 – 7.13 (m, 6H), 7.00 (d, *J* = 7.6 Hz, 1H), 6.78 (d, *J* = 7.6 Hz, 1H), 4.36-4.23 (m, 2H), 4.10 – 3.99 (m, 1H), 3.92 (ddd, *J* = 10.9, 9.2, 6.7 Hz, 1H).

^{13}C NMR (100 MHz, CDCl$_3$) δ 170.1, 169.4, 167.0, 152.4, 135.1, 133.3, 133.0, 131.9, 130.5, 130.1, 129.4, 128.5 (2C), 127.2, 127.1, 62.5, 54.8, 42.7.

IR (neat) v: 1784, 1666, 1577, 1389, 1296, 1185, 1039, 758, 716, 624, 589, 555 cm^{-1}.

HRMS (ESI$^+$): exact mass calculated for [M+Na]$^+$ ($C_{18}H_{15}N_2O_4{}^{35}ClNa^+$) requires m/z 381.0613, found m/z 381.0613.

***N*-(1-(naphthalen-1-yl)-2-oxo-2-(2-oxooxazolidin-3-yl)ethyl)benzamide (3t)**

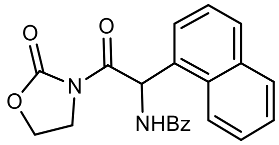

Prepared according to General Procedure 3 in 71% yield as a white solid.

^{1}H NMR (600 MHz, CDCl$_3$) δ 8.38 (d, *J* = 8.5 Hz, 1H), 7.88 (d, *J* = 8.0 Hz, 2H), 7.84 – 7.69 (m, 3H), 7.61 (ddd, *J* = 8.4, 6.9, 1.3 Hz, 1H), 7.58 – 7.50 (m, 1H), 7.50 – 7.33 (m, 5H), 6.77 (d, *J* = 8.0 Hz, 1H), 4.47 – 4.25 (m, 2H), 4.20-4.13 (m, 1H), 4.09 – 4.01 (m, 1H).

^{13}C NMR (150 MHz, CDCl$_3$) δ 171.2, 167.0, 152.5, 134.2, 133.4, 131.9, 131.8, 131.5, 129.9, 128.6, 128.5 (2C), 127.3, 127.2 (2C), 126.5, 125.0, 124.8, 123.9, 62.3, 53.0, 42.6.

IR (neat) v: 1775, 1709, 1643, 1527, 1480, 1388, 1284, 1119, 1042, 801, 778, 755, 714 cm^{-1}.

HRMS (ESI$^+$): exact mass calculated for [M+Na]$^+$ ($C_{22}H_{18}N_2O_4Na^+$) requires m/z 397.1159, found m/z 397.1157.

3-benzamido-4-oxo-4-(2-oxooxazolidin-3-yl)butyl-2-(3-cyano-4-isobutoxyphenyl)-4-methylthiazole-5-carboxylate (3u)

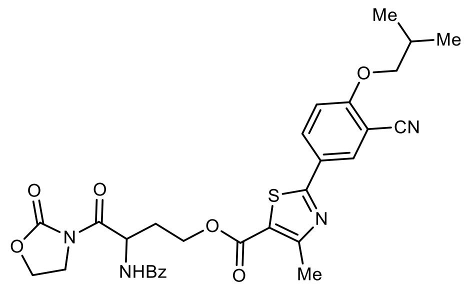

Prepared according to General Procedure 3 in 51% yield as a white solid.

^{1}H NMR (400 MHz, $CDCl_3$) δ 8.03 – 7.91 (m, 2H), 7.75 (dd, *J* = 5.2, 3.3 Hz, 2H), 7.52 – 7.41 (m, 1H), 7.35 (dd, *J* = 10.4, 4.7 Hz, 2H), 7.00 (d, *J* = 8.1 Hz, 1H), 6.92 (d, *J* = 8.9 Hz, 1H), 5.89 (td, *J* = 8.5, 3.7 Hz, 1H), 4.50 (dt, *J* = 11.6, 5.8 Hz, 1H), 4.43-4.32 (m, 3H), 4.04 (ddd, *J* = 10.9, 8.8, 7.2 Hz, 1H), 3.99 – 3.89 (m, 1H), 3.83 (d, *J* = 6.5 Hz, 2H), 2.67 (s, 3H), 2.51 – 2.38 (m, 1H), 2.21 – 2.08 (m, 2H), 1.02 (d, *J* = 6.7 Hz, 6H).

^{13}C NMR (100 MHz, $CDCl_3$) δ 172.0, 167.3, 167.2, 162.5, 161.8, 152.8, 133.6, 132.6, 132.0, 131.9, 128.6 (2C), 127.2 (2C), 125.8, 121.1, 116.1, 115.3, 112.5, 102.9, 75.7, 62.6, 61.8, 50.4, 42.6, 31.3, 28.1, 19.0 (2C), 17.5.

IR (neat) ν: 1780, 1711, 1661, 1605, 1525, 1431, 1371, 1221, 1096, 1012, 758, 715 cm^{-1}.

HRMS (ESI$^+$): exact mass calculated for $[M+Na]^+$ ($C_{30}H_{30}N_4O_7SNa^+$) requires m/z 613.1727, found m/z 613.1736.

4.4 Characterizations of the α-amino thioesters

S-Methyl 2-benzamido-2-cyclopentylethanethioate (5a)

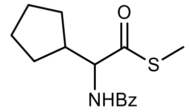

Prepared according to General Procedure 4 in 76% yield as a yellow solid.

^{1}H-NMR (700 MHz, $CDCl_3$) δ 7.80 (d, *J* = 7.4 Hz, 2H), 7.52 (t, *J* = 7.2 Hz, 1H), 7.45 (t, *J* = 7.3 Hz, 2H), 6.60 (d, *J* = 7.9 Hz, 1H), 4.89 (t, *J* = 7.9 Hz, 1H), 2.46 – 2.36 (m, 1H), 2.32 (s, 3H), 1.84 – 1.32 (m, 8H).

^{13}C-NMR (176 MHz, $CDCl_3$) δ 200.7, 167.4, 134.1, 132.0, 128.8 (2C), 127.2 (2C), 62.5, 42.9, 29.5, 28.5, 25.4, 25.2, 11.7.

IR (neat) ν: 3306, 2954, 2867, 1686, 1639, 1521, 1487, 692 cm^{-1}.

HRMS (ESI$^+$): exact mass calculated for $[M+Na]^+$ ($C_{15}H_{19}NO_2SNa^+$) requires m/z 300.1029, found 300.1024 m/z.

S-Methyl 2-benzamido-2-cyclohexylethanethioate (5b)

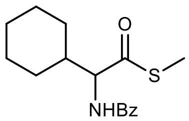

Prepared according to General Procedure 4 in 65% yield as a yellow solid.

^{1}H NMR (600 MHz, CDCl$_3$) δ 7.82 (dt, *J* = 8.4, 1.7 Hz, 2H), 7.57 – 7.50 (m, 1H), 7.50 – 7.42 (m, 2H), 6.58 (d, *J* = 8.9 Hz, 1H), 4.88 (dd, *J* = 9.1, 5.5 Hz, 1H), 2.33 (s, 3H), 2.05 – 1.97 (m, 1H), 1.77 (tdd, *J* = 4.9, 3.3, 1.7 Hz, 3H), 1.71 – 1.65 (m, 2H), 1.33 – 1.06 (m, 5H).

^{13}C NMR (151 MHz, CDCl$_3$) δ 200.6, 167.4, 134.2, 132.0, 128.8 (2C), 127.2 (2C), 63.9, 41.4, 30.1, 27.9, 26.1, 26.1 (2C), 11.7.

IR (neat) ν: 3301, 2927, 2853, 1686, 1643, 1524, 1487, 745 cm^{-1}.

HRMS (ESI$^+$): exact mass calculated for [M+Na]$^+$ ($C_{16}H_{21}NO_2SNa^+$) requires m/z 314.1186, found 314.1176 m/z.

S-Methyl 2-benzamido-3-methylbutanethioate (5c)

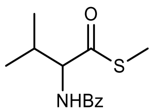

Prepared according to General Procedure 4 in 62% yield as a yellow solid.

^{1}H NMR (700 MHz, CDCl$_3$) δ 7.83 (dt, *J* = 8.4, 1.6 Hz, 2H), 7.54 (ddd, *J* = 6.9, 4.0, 1.2 Hz, 1H), 7.49 – 7.46 (m, 2H), 6.55 (d, *J* = 8.7 Hz, 1H), 4.91 (dd, *J* = 9.2, 4.9 Hz, 1H), 2.41 – 2.36 (m, 1H), 2.33 (s, 3H), 1.05 (d, *J* = 6.8 Hz, 3H), 0.97 (d, *J* = 6.9 Hz, 3H).

^{13}C NMR (176 MHz, CDCl$_3$) δ 200.7, 167.6, 134.2, 132.0, 128.9 (2C), 127.2 (2C), 64.2, 31.7, 19.7, 17.4, 11.7.

IR (neat) ν: 3304, 2963, 2928, 1684, 1642, 1520, 1487, 712, 693 cm^{-1}.

HRMS (ESI$^+$): exact mass calculated for [M+Na]$^+$ ($C_{15}H_{19}NO_2SNa^+$) requires m/z 274.0872, found 274.0873 m/z.

S-Methyl 2-benzamidohexanethioate (5d)

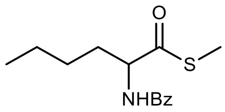

Prepared according to General Procedure 5 in 65% yield as a yellow foam.

^{1}H NMR (700 MHz, CDCl$_3$) δ 7.83 – 7.81 (m, 2H, H_{11}), 7.56 – 7.52 (m, 1H, H_{13}), 7.48 – 7.45 (m, 2H, H_{12}), 6.54 (d, *J* = 7.9 Hz, 1H, H_8), 4.94 (td, *J* = 8.3, 5.0 Hz, 1H, H_3), 2.33 (s, 3H, H_1), 2.06 – 1.97 (m, 1H, H_4), 1.79 – 1.71 (m, 1H, $H_{4'}$), 1.43 – 1.32 (m, 4H, $H_{5\text{-}6}$), 0.90 (dt, *J* = 11.5, 4.2 Hz, 3H, H_7).

^{13}C NMR (176 MHz, CDCl$_3$) δ 201.1, 167.3, 134.0, 132.0, 128.9 (2C), 127.2 (2C), 59.6, 33.0, 27.6, 22.5, 14.0, 11.6.

IR (neat) ν: 3308, 2956, 2859, 1687, 1642, 1527, 1489, 739 cm^{-1}.

HRMS (ESI$^+$): exact mass calculated for [M+Na]$^+$ ($C_{14}H_{19}NO_2SNa^+$) requires m/z 288.1029, found 288.1024 m/z.

***S*-Methyl 2-benzamidohexanethioate (5e)**

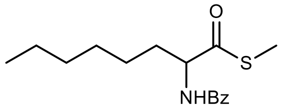

Prepared according to General Procedure 5 in 70% yield as a yellow foam.

^{1}H NMR (600 MHz, CDCl$_3$) δ 7.82 (dt, *J* = 8.5, 1.7 Hz, 2H), 7.55 – 7.51 (m, 1H), 7.48 – 7.44 (m, 2H), 6.58 (d, *J* = 8.2 Hz, 1H), 4.93 (td, *J* = 8.3, 5.0 Hz, 1H), 2.32 (s, 3H), 2.03 – 1.97 (m, 1H), 1.78 – 1.70 (m, 1H), 1.43 – 1.24 (m, 8H), 0.87 (t, *J* = 7.0 Hz, 3H).

^{13}C NMR (151 MHz, CDCl$_3$) δ 201.1, 167.3, 134.1, 132.0, 128.8 (2C), 127.2 (2C), 59.6, 33.3, 31.7, 29.0, 25.4, 22.7, 14.2, 11.6.

IR (neat) ν: 3306, 2952, 2858, 1684, 1642, 1527, 1489, 738, 695 cm^{-1}.

HRMS (ESI$^+$): exact mass calculated for [M+Na]$^+$ ($C_{16}H_{23}NO_2SNa^+$) requires m/z 316.1342, found 316.1344 m/z.

***S*-Methyl 2-benzamido-5-methylhexanethioate (5f)**

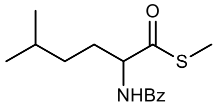

Prepared according to General Procedure 5 in 59% yield as a yellow foam.

^{1}H NMR (400 MHz, CDCl$_3$) δ 7.85 – 7.79 (m, 2H), 7.57 – 7.49 (m, 1H), 7.48 – 7.42 (m, 2H), 6.61 (d, *J* = 8.2 Hz, 1H), 4.92 (td, *J* = 8.3, 5.0 Hz, 1H), 2.32 (s, 3H), 2.08 – 1.96 (m, 1H), 1.80 – 1.70 (m, 1H), 1.57 (dp, *J* = 13.3, 6.7 Hz, 1H), 1.34 – 1.22 (m, 2H), 0.88 (d, *J* = 6.6 Hz, 6H).

^{13}C NMR (100 MHz, CDCl$_3$) δ 201.0, 167.2, 133.9, 131.8, 128.6 (2C), 127.1 (2C), 59.6, 34.2, 31.0, 27.8, 22.5, 22.2, 11.5.

IR (neat) v: 1713, 1643, 1527, 1489, 1467, 1366, 1331, 1310, 1287, 713, 694, 531 cm^{-1}.

HRMS (ESI$^+$): exact mass calculated for $[M+Na]^+$ ($C_{15}H_{21}NO_2SNa^+$) requires m/z 302.1185, found 302.1187 m/z.

***S*-Methyl 2-benzamido-3-cyclohexylpropanethioate (5g)**

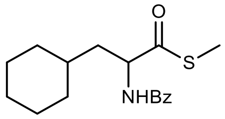

Prepared according to General Procedure 5 in 62% yield as a yellow foam.

^{1}H NMR (400 MHz, CDCl$_3$) δ 7.87 – 7.78 (m, 2H), 7.52 (dd, *J* = 5.0, 3.7 Hz, 1H), 7.50 – 7.43 (m, 2H), 6.46 (d, *J* = 8.4 Hz, 1H), 4.99 (ddd, *J* = 9.8, 8.7, 4.7 Hz, 1H), 2.30 (s, 3H), 1.85 (ddd, *J* = 13.7, 8.8, 4.7 Hz, 2H), 1.74 – 1.55 (m, 5H), 1.49 – 1.35 (m, 1H), 1.28 – 1.11 (m, 3H), 1.06 – 0.88 (m, 2H).

^{13}C NMR (100 MHz, CDCl$_3$) δ 201.6, 167.2, 133.8, 131.9, 128.7 (2C), 127.1 (2C), 57.4, 40.6, 34.2, 33.7, 32.3, 26.3, 26.1, 25.9, 11.5.

IR (neat) v: 1789, 1739, 1718, 1436, 1420, 1367, 1353, 1229, 1219, 1202, 530 cm^{-1}.

HRMS (ESI$^+$): exact mass calculated for $[M+Na]^+$ ($C_{17}H_{23}NOSNa^+$) requires m/z 328.1342, found 328.1340 m/z.

***S*-Methyl 2-benzamido-5-chloropentanethioate (5h)**

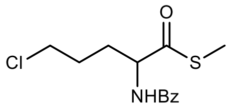

Prepared according to General Procedure 5 in 68% yield as a yellow foam.

^{1}H NMR (700 MHz, CDCl$_3$) δ 7.82 (dd, *J* = 5.2, 3.2 Hz, 2H), 7.57 – 7.52 (m, 1H), 7.51 – 7.44 (m, 2H), 6.64 (d, *J* = 8.2 Hz, 1H), 4.98 (td, *J* = 8.1, 4.8 Hz, 1H), 3.61 – 3.56 (m, 2H), 2.35 (s, 3H), 2.25 – 2.18 (m, 1H), 1.98 – 1.85 (m, 3H).

^{13}C NMR (176 MHz, CDCl$_3$) δ 200.5, 167.3, 133.7, 132.2, 128.9 (2C), 127.3 (2C), 58.8, 44.4, 30.7, 28.6, 11.7.

IR (neat) *v*: 3304, 2928, 1684, 1642, 1580, 1526, 1488, 1289, 694, 651 cm^{-1}.

HRMS (ESI$^+$): exact mass calculated for $[M+Na]^+$ ($C_{13}H_{16}NO_2SClNa^+$) requires m/z 308.0483, found 308.0482 m/z.

4-Benzamido-5-(methylthio)-5-oxopentyl acetate (5i)

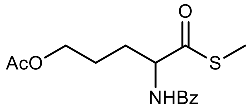

Prepared according to General Procedure 5 in 65% yield as a yellow foam.

^{1}H NMR (700 MHz, $CDCl_3$) δ 7.82 (dd, *J* = 8.2, 1.1 Hz, 2H), 7.56 – 7.52 (m, 1H), 7.46 (dd, *J* = 10.6, 4.8 Hz, 2H), 6.73 (d, *J* = 8.2 Hz, 1H), 4.98 (td, *J* = 8.1, 5.0 Hz, 1H), 4.11 – 4.07 (m, 2H), 2.33 (s, 3H), 2.14 – 2.07 (m, 1H), 2.04 (s, 3H), 1.84 – 1.72 (m, 3H).

^{13}C NMR (176 MHz, $CDCl_3$) δ 200.7, 171.2, 167.4, 133.8, 132.1, 128.9 (2C), 127.3 (2C), 63.8, 59.2, 29.8, 24.9, 21.1, 11.9.

IR (neat) ν: 3318, 2929, 1735, 1683, 1642, 1523, 1488, 1233, 1037, 712, 693 cm^{-1}.

HRMS (ESI$^+$): exact mass calculated for $[M+Na]^+$ ($C_{15}H_{19}NO_2SNa^+$) requires m/z 332.0927, found 332.0925 m/z.

***S*-Methyl 2-benzamido-5-(1,3-dioxoisoindolin-2-yl)pentanethioate (5j)**

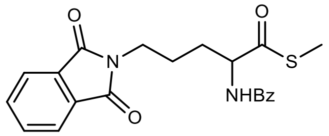

Prepared according to General Procedure 5 in 66% yield as a yellow foam.

^{1}H NMR (600 MHz, $CDCl_3$) δ 7.86 – 7.82 (m, 2H), 7.81 (dt, *J* = 7.2, 3.4 Hz, 2H), 7.69 (dt, *J* = 7.3, 3.2 Hz, 2H), 7.51 (t, *J* = 7.1 Hz, 1H), 7.43 (t, *J* = 7.6 Hz, 2H), 6.97 (d, *J* = 8.3 Hz, 1H), 4.98 (td, *J* = 8.4, 4.8 Hz, 1H), 3.72 (t, *J* = 6.8 Hz, 2H), 2.29 (s, 3H), 2.10 – 2.04 (m, 1H), 1.88 – 1.75 (m, 3H).

^{13}C NMR (151 MHz, $CDCl_3$) δ 200.8, 168.6 (2C), 167.4, 134.2 (2C), 133.7, 132.1 (2C), 132.0, 128.8 (2C), 127.4 (2C), 123.4 (2C), 59.4, 37.5, 29.9, 25.2, 11.7.

IR (neat) ν: 3325, 2929, 1771, 1704, 1643, 1522, 1436, 714, 692, 529 cm^{-1}.

HRMS (ESI$^+$): exact mass calculated for $[M+Na]^+$ ($C_{21}H_{20}N2O_4SNa^+$) requires m/z 419.1036, found 419.1035 m/z.

S-Methyl 2-benzamido-3,3-dimethylbutanethioate (5k)

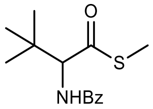

Prepared according to General Procedure 5 in 55% yield as a yellow foam.

^{1}H NMR (700 MHz, CDCl$_3$) δ 7.83 – 7.78 (m, 2H), 7.55 – 7.50 (m, 1H), 7.49 – 7.44 (m, 2H), 6.64 (d, *J* = 9.3 Hz, 1H), 4.79 (d, *J* = 9.5 Hz, 1H), 2.32 (s, 3H), 1.09 (s, 9H).

^{13}C NMR (176 MHz, CDCl$_3$) δ 199.7, 167.1, 134.2, 132.0, 128.9 (2C), 127.2 (2C), 66.5, 35.2, 27.0 (3C), 12.0.

IR (neat) ν: 3327, 2962, 2928, 1689, 1638, 1529, 1490, 1328, 1271, 1075, 690, 645 cm^{-1}.

HRMS (ESI$^+$): exact mass calculated for [M+Na]$^+$ ($C_{14}H_{19}NO_2SNa^+$) requires m/z 288.1029, found 288.1025 m/z.

S-Methyl 2-benzamido-2-phenylethanethioate (5l)

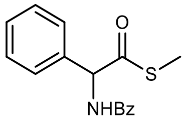

Prepared according to General Procedure 5 in 63% yield as a white foam.

^{1}H NMR (400 MHz, CDCl$_3$) δ 7.87 – 7.79 (m, 2H), 7.51 (ddt, *J* = 5.5, 4.2, 2.1 Hz, 1H), 7.47 – 7.32 (m, 7H), 7.20 (d, *J* = 6.7 Hz, 1H), 5.92 (d, *J* = 7.1 Hz, 1H), 2.34 (s, 3H).

^{13}C NMR (100 MHz, CDCl$_3$) δ 198.5, 166.6, 136.5, 133.5, 131.9, 129.1 (2C), 128.8, 128.6 (2C), 127.7 (2C), 127.2 (2C), 63.6, 11.7.

IR (neat) ν: 1640, 1580, 1519, 1484, 1313, 1235, 1149, 1029, 714, 593, 531 cm^{-1}.

HRMS (ESI$^+$): exact mass calculated for [M+Na]$^+$ ($C_{16}H_{15}NO_2SNa^+$) requires m/z 308.0716, found 308.0717 m/z.

S-Methyl 2-benzamido-2-(4-(trifluoromethyl)phenyl)ethanethioate (5m)

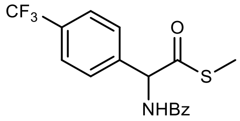

Prepared according to General Procedure 5 in 54% yield as a white foam.

^{1}**H NMR (400 MHz, $CDCl_3$)** δ 7.86 – 7.78 (m, 2H), 7.65 (d, *J* = 8.3 Hz, 2H), 7.62 – 7.51 (m, 3H), 7.45 (t, *J* = 7.5 Hz, 2H), 7.33 (d, *J* = 6.6 Hz, 1H), 5.97 (d, *J* = 6.9 Hz, 1H), 2.37 (s, 3H).

^{13}**C NMR (100 MHz, $CDCl_3$)** δ 197.7, 166.5, 140.6, 133.2, 132.2, 131.0 (q, $^2J_{C\text{-}F}$ = 32.8 Hz), 128.7 (2C), 128.1 (2C), 127.2 (2C), 126.1 (q, $^3J_{C\text{-}F}$ = 3.7 Hz, 2C), 123.8 (q, $^1J_{C\text{-}F}$ = 272.3 Hz), 63.2, 11.8.

^{19}**F NMR (377 MHz, $CDCl_3$)** δ -62.7.

IR (neat) ν: 1690, 1525, 1512, 1486, 1419, 1325, 1167, 1128, 1114, 1068, 1019, 713, 692 cm^{-1}.

HRMS (ESI⁺): exact mass calculated for $[M+Na]^+$ ($C_{17}H_{14}NO_2SF_3Na^+$) requires m/z 376.0590, found 376.0591 m/z.

***S*-(*p*-Tolyl) 2-benzamidohexanethioate (5n)**

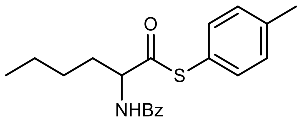

Prepared according to General Procedure 5 in 45% yield as a white solid.

^{1}**H NMR (600 MHz, $CDCl_3$)** δ 7.84 (dt, *J* = 8.5, 1.7 Hz, 2H), 7.57 – 7.51 (m, 1H), 7.47 (tt, *J* = 6.8, 1.2 Hz, 2H), 7.32 – 7.28 (m, 2H), 7.25 – 7.21 (m, 2H), 6.58 (d, *J* = 8.4 Hz, 1H), 5.06 (td, *J* = 8.3, 4.9 Hz, 1H), 2.38 (s, 3H), 2.08 (tdd, *J* = 9.4, 7.5, 5.0 Hz, 1H), 1.85 – 1.78 (m, 1H), 1.46 – 1.35 (m, 4H), 0.92 (t, *J* = 7 Hz, 3H).

^{13}**C NMR (151 MHz, $CDCl_3$) δ** 199.4, 167.3, 140.1, 134.7, 134.0 (2C), 132.1, 130.3 (2C), 128.9 (2C), 127.3 (2C), 123.3, 59.4, 33.0, 27.6, 22.5, 21.5, 14.0.

IR (neat) ν: 3296, 2955, 2926, 2860, 1701, 1640, 1523, 1488, 806, 792 cm^{-1}.

HRMS (ESI⁺): exact mass calculated for $[M+Na]^+$ ($C_{20}H_{23}NO_2SNa^+$) requires m/z 364.1342, found 364.1340 m/z.

4-Benzamido-5-(methylthio)-5-oxopentyl 2-(1-(4-chlorobenzoyl)-5-methoxy-2-methyl-1H-indol-3-yl)acetate (5o)

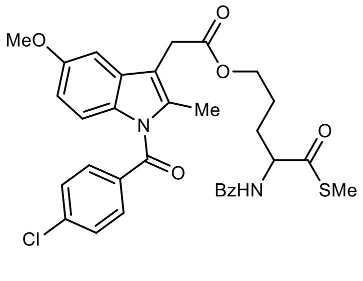

Prepared according to General Procedure 5 in 41% yield as a white foam.

^{1}**H NMR (600 MHz, $CDCl_3$)** δ 7.86 – 7.78 (m, 2H), 7.65 (d, *J* = 8.5 Hz, 2H), 7.54 (t, *J* = 7.4 Hz, 1H), 7.50 – 7.42 (m, 4H), 6.97 (d, *J* = 2.4 Hz, 1H), 6.86 (d, *J* = 9.0 Hz, 1H), 6.70 – 6.61 (m, 2H), 4.93 (td, *J* = 8.4, 5.0 Hz, 1H),

4.21 – 4.09 (m, 2H), 3.79 (s, 3H), 3.66 (s, 2H), 2.38 (s, 3H), 2.30 (s, 3H), 2.04-1.98 (m, 1H), 1.77-1.74 (m, 2H), 1.71 – 1.65 (m, 1H).

^{13}C NMR (150 MHz, CDCl$_3$) δ 200.3, 170.8, 168.3, 167.2, 155.9, 139.3, 136.0, 133.8, 133.6, 132.0, 131.2 (2C), 130.9, 130.6, 129.1 (2C), 128.7 (2C), 127.1 (2C), 114.9, 112.5, 111.5, 101.6, 64.2, 58.9, 55.8, 30.4, 29.7, 24.9, 13.4, 11.5.

IR (neat) v: 2959, 2923, 2852, 1738, 1717, 1687, 1660, 1479, 1457, 1360, 1325, 1222, 531 cm^{-1}.

HRMS (ESI$^+$): exact mass calculated for [M+Na]$^+$ ($C_{32}H_{31}N_2O_6S^{35}ClNa^+$) requires m/z 629.1484, found 629.1489 m/z.

5. Chirality transfer study

5.1 Conditions optimization

Supplementary Table 4 | Optimizations of enantioselective hydrative amination[a]

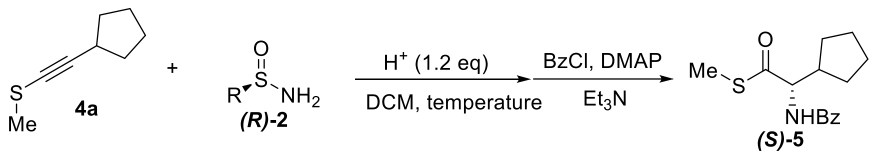

Entry	R	Acid	temperature	Yield	e.r.
1	^{t}Bu	HOTf	0 °C to rt	76%	86:14
2	^{t}Bu	HOTf	0 °C	52%	88:12
3	***^{t}Bu***	***$HNTf_2$***	***0 °C to rt***	***65%***	***88:12***
4	^{t}Bu	$HNTf_2$	0 °C	40%	89:11
5	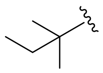	$HNTf_2$	0 °C	42%	89:11
6	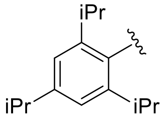	$HNTf_2$	0 °C	trace	

[a]To a flame-dried Schlenk tube, the CH_2Cl_2 (1.0 mL) solution of thialkyne **4a** (0.20 mmol) was added under Ar atmosphere. Then the solution was cooled down to 0 °C before a solution of Tf_2NH (68.0 mg, 0.24 mmol, 1.20 equiv.) in CH_2Cl_2 (1.0 mL) was added in dropwise. The reaction mixture was then stirred for 15 min at the same temperature. *(R)*-sulfinamide (0.40 mmol, 2.00 equiv.) was then added to the reaction in one portion before it's allowed to stirred at room temperature for 16 h. Triethylamine (84 µL, 0.60 mmol, 3.00 equiv.), 4-dimethylaminopyridine (DMAP, 5.0 mg, 0.04 mmol, 0.20 equiv.) and benzoyl chloride (70 µL, 0.60 mmol, 3.00 equiv.) were then added in sequence. The reaction was stirred at room temperature for another 3 h before the addition of a saturated aqueous solution of sodium bicarbonate. The combined organic layers were dried over anhydrous magnesium sulfate, the dried solution was filtered and the filtrate was concentrated under reduced pressure to afford the crude product. The crude product was then purified by column to give the desired enantioenriched aminated amide. Enantiomeric ratio (e.r.) was determined by chiral HPLC analysis.

5.2 Hydrative amination of ynamides with enantioenriched sulfinamide

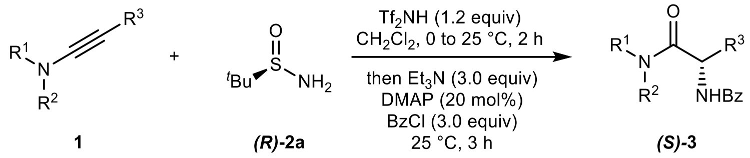

General procedure 6: To a flame-dried Schlenk tube, the CH_2Cl_2 (1.0 mL) solution of ynamide (0.20 mmol) was added under Ar atmosphere. Then the solution was cooled down to 0 °C before a solution of Tf_2NH (68.0 mg, 0.24 mmol, 1.20 equiv.) in CH_2Cl_2 (1.0 mL) was added in dropwise. The reaction mixture was then stirred for 15 min at the same temperature. *(R)*-2-methylpropane-2-sulphinamide (48.5 mg, 0.40 mmol, 2.00 equiv.) was then added to the reaction in one portion before it's allowed to stirred at room temperature for 2 h. Triethylamine (84 µL, 0.60 mmol, 3.00 equiv.), 4-dimethylaminopyridine (DMAP, 5.0 mg, 0.04 mmol, 0.20 equiv.) and benzoyl chloride (70 µL, 0.60 mmol, 3.00 equiv.) were then added in sequence. The reaction was stirred at room temperature for another 3 h before the addition of a saturated aqueous solution of sodium bicarbonate. The combined organic layers were dried over anhydrous magnesium sulfate, the dried solution was filtered and the filtrate was concentrated under reduced pressure to afford the crude product. The crude product was then purified by column to give the desired enantioenriched aminated amide.

***(S)-N*-(3,3-dimethyl-1-oxo-1-(2-oxooxazolidin-3-yl)butan-2-yl)benzamide (*(S)*-3h)**

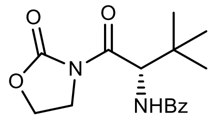

Prepared according to General Procedure 6 in 50% yield as a white solid.

Enantiomeric ratio = 99:1 was determined by chiral HPLC analysis: Lux-Cellulose 1, *n*-heptane+0.1%IPA/IPA 80:20, 1 mL/min, 25 °C, detection at 230 nm, retention time (min): 21.8 (major) and 19.2 (minor).

$[\alpha]_D^{20}$ = 0.65 (c = 1.0, $CHCl_3$).

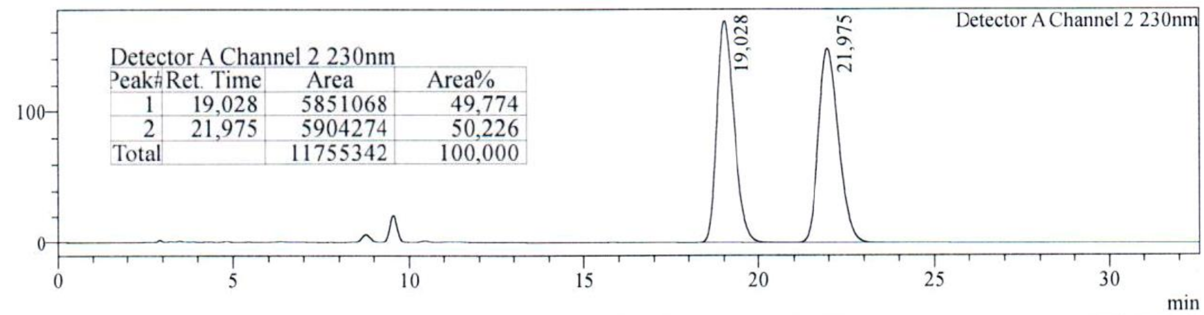

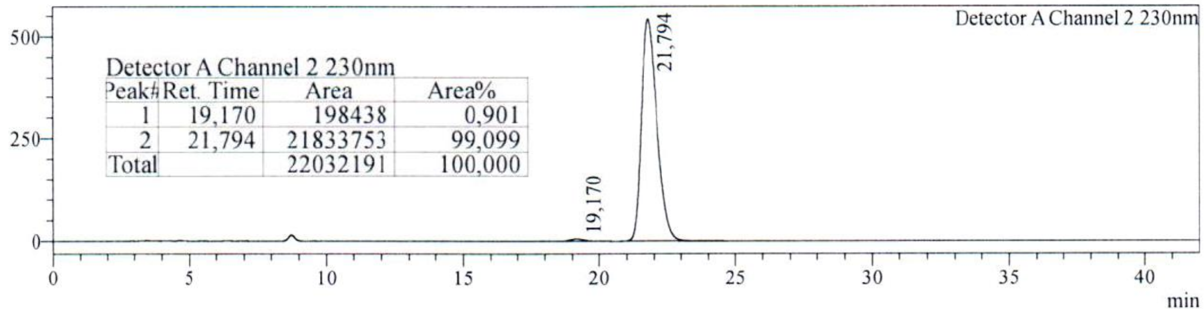

***(S)*-*N*-(1-cyclopentyl-2-oxo-2-(2-oxooxazolidin-3-yl)ethyl)benzamide (*(S)*-3g)**

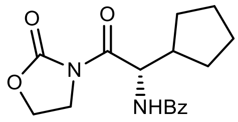

Prepared according to General Procedure 63 in 50% yield as a white solid.

Enantiomeric ratio = 62:38 was determined by chiral HPLC analysis: Lux-Cellulose 1, *n*-heptane+0.1%IPA/IPA 80:20, 1 mL/min, 25 °C, detection at 230 nm, retention time (min): 21.8 (major) and 19.2 (minor).

$[\alpha]_D^{20}$ = 0.15 (c = 1.0, $CHCl_3$).

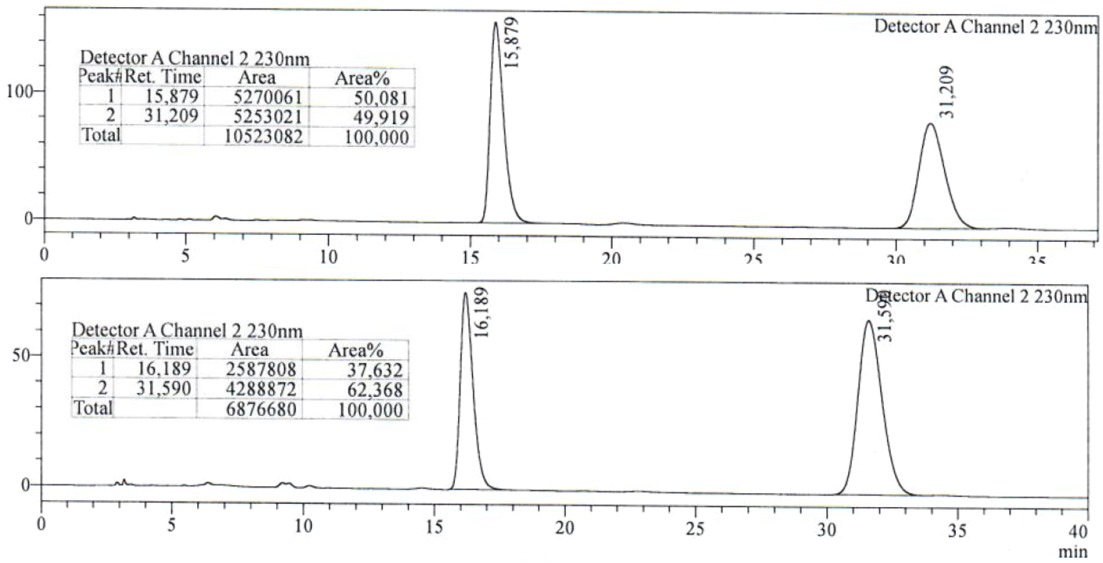

***(S)*-*N*-(1-oxo-1-(2-oxooxazolidin-3-yl)hexan-2-yl)benzamide ((*S*)-3a)**

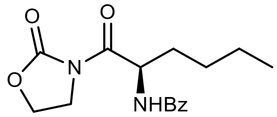

Prepared according to General Procedure 6 in 65% yield as a white solid.

Enantiomeric ratio = 68:32 was determined by chiral HPLC analysis: Lux-Cellulose 1, *n*-heptane+0.1%IPA/IPA 80:20, 1 mL/min, 25 °C, detection at 230 nm, retention time (min): 21.8 (major) and 19.2 (minor).

$[\alpha]_D^{20}$ = -0.65 (c = 1.0, $CHCl_3$).

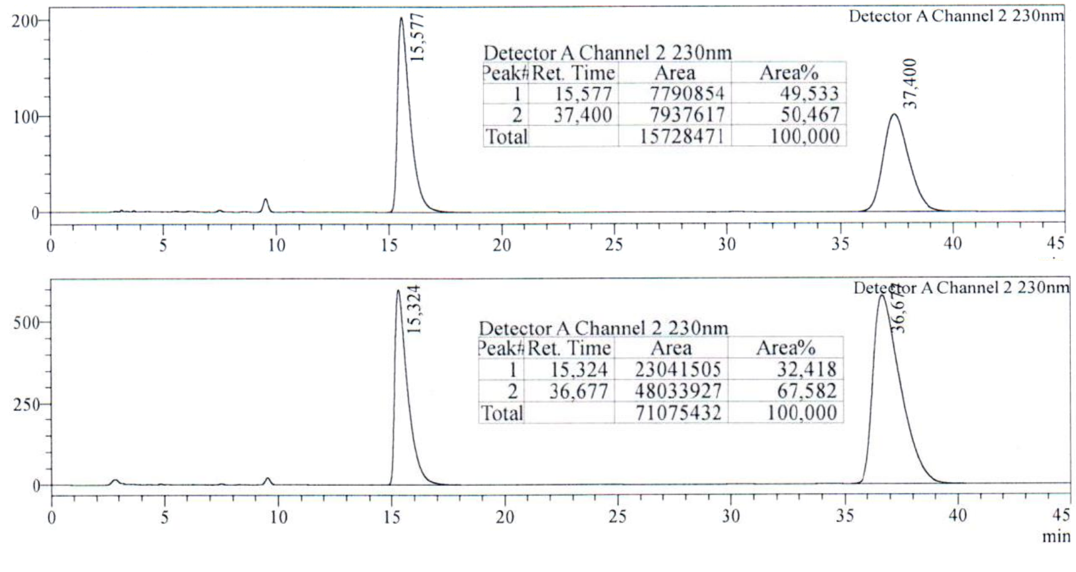

***(S)*-*N*-(2-oxo-2-(2-oxooxazolidin-3-yl)-1-(p-tolyl)ethyl)benzamide (*(S)*-3k)**

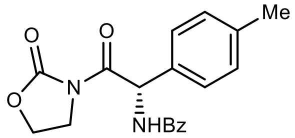

Prepared according to General Procedure 6 in 73% yield as a white solid.

Enantiomeric ratio = 79:21 was determined by chiral HPLC analysis: Lux-Cellulose 1, *n*-heptane+0.1%IPA/IPA 80:20, 1 mL/min, 25 °C, detection at 230 nm, retention time (min): 33.5 (major) and 23.6 (minor).

$[\alpha]_D^{20}$ = 0.65 (c = 1.0, $CHCl_3$).

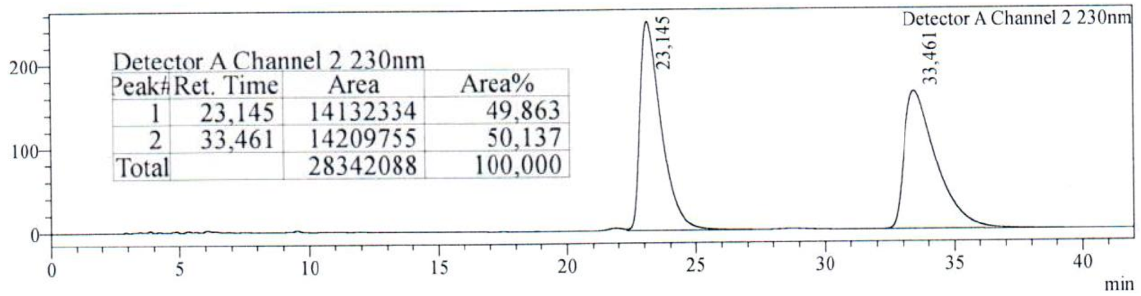

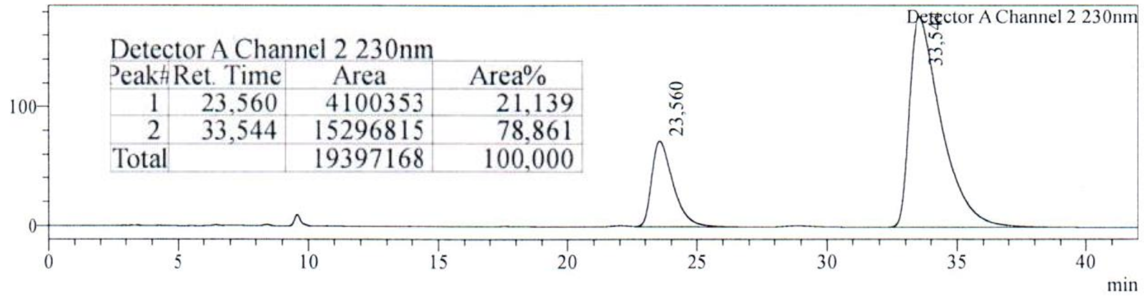

5.3 Hydrative amination of thioalkynes with enantioenriched sulfinamide

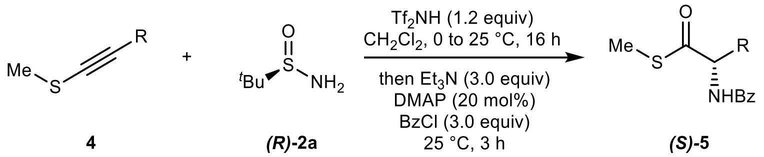

General procedure 7: To a flame-dried Schlenk tube, the CH_2Cl_2 (1.0 mL) solution of thialkyne **4** (0.20 mmol) was added under Ar atmosphere. Then the solution was cooled down to 0 °C before a solution of Tf_2NH (68.0 mg, 0.24 mmol, 1.20 equiv.) in CH_2Cl_2 (1.0 mL) was added in dropwise. The reaction mixture

was then stirred for 15 min at the same temperature. *(R)*- 2-methylpropane-2-sulphinamide (48.5 mg, 0.40 mmol, 2.00 equiv.) was then added to the reaction in one portion before it's allowed to stirred at room temperature for 16 h. Triethylamine (84 µL, 0.60 mmol, 3.00 equiv.), 4-dimethylaminopyridine (DMAP, 5.0 mg, 0.04 mmol, 0.20 equiv.) and benzoyl chloride (70 µL, 0.60 mmol, 3.00 equiv.) were then added in sequence. The reaction was stirred at room temperature for another 3 h before the addition of a saturated aqueous solution of sodium bicarbonate. The combined organic layers were dried over anhydrous magnesium sulfate, the dried solution was filtered and the filtrate was concentrated under reduced pressure to afford the crude product. The crude product was then purified by column to give the desired enantioenriched aminated amide.

***S*-Methyl *(S)*-2-benzamido-3,3-dimethylbutanethioate (*(S)*-5k)**

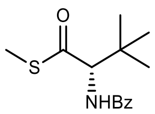

Prepared according to General Procedure 7 in 54% yield as a yellow foam.

Enantiomeric ratio = 96:4 was determined by chiral HPLC analysis: Chiralpak IC, *n*-heptane+0.1%IPA/IPA 70:30, 1 mL/min, 25 °C, detection at 254 nm, retention time (min): 6.0 (major) and 7.3 (minor).

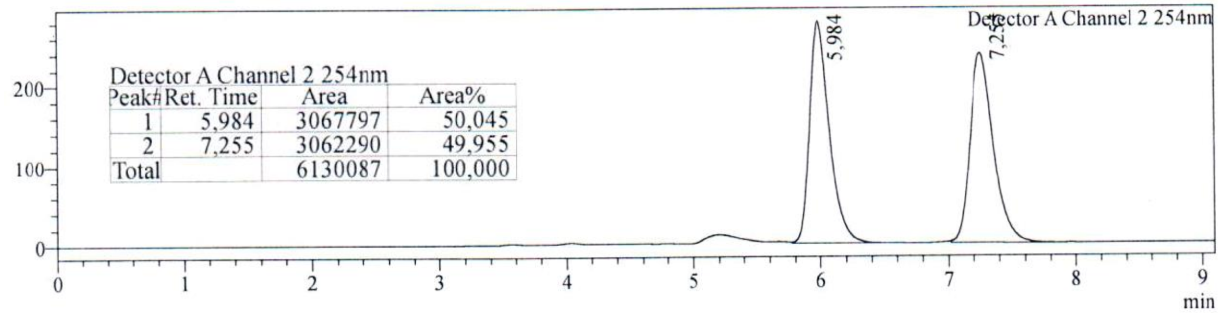

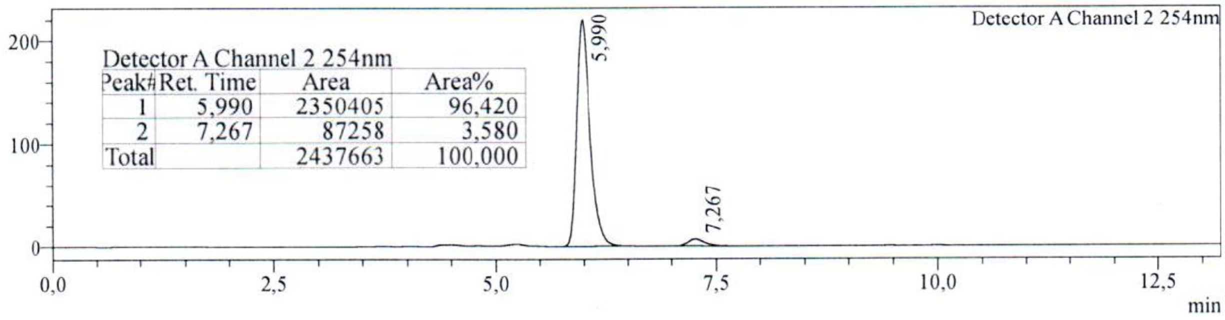

***S*-methyl *(S)*-2-benzamido-2-cyclopentylethanethioate (*(S)*-5a)**

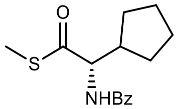

Prepared according to General Procedure 7 in 65% yield as a white solide.

Enantiomeric ratio = 96:4 was determined by chiral HPLC analysis: Chiralpak IC, *n*-heptane+0.1%IPA/IPA 70:30, 1 mL/min, 25 °C, detection at 254 nm, retention time (min): 6.0 (major) and 7.3 (minor).

$[\alpha]_D^{20}$ = 0.09 (c = 1.0, $CHCl_3$).

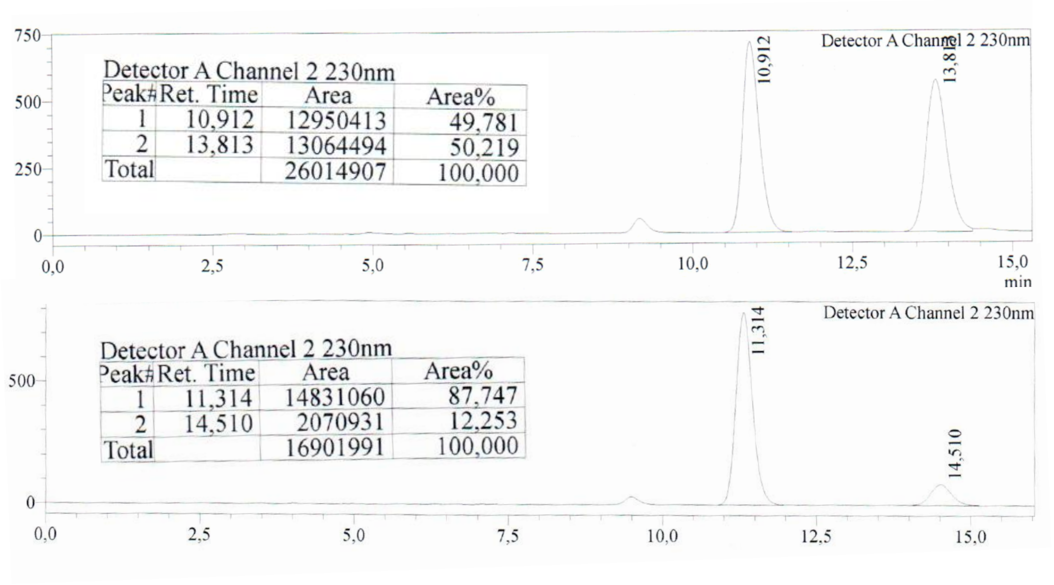

S-**methyl** ***(S)*-2-benzamidohexanethioate (*(S)*-5d)**

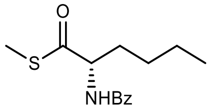

Prepared according to General Procedure 7 in 64% yield as a yellow foam.

Enantiomeric ratio = 83:17 was determined by chiral HPLC analysis: Chiralpak IC, *n*-heptane+0.1%IPA/IPA 70:30, 1 mL/min, 25 °C, detection at 254 nm, retention time (min): 6.6 (major) and 7.4 (minor).

$[\alpha]_D^{20}$ = 0.17 (c = 1.0, $CHCl_3$).

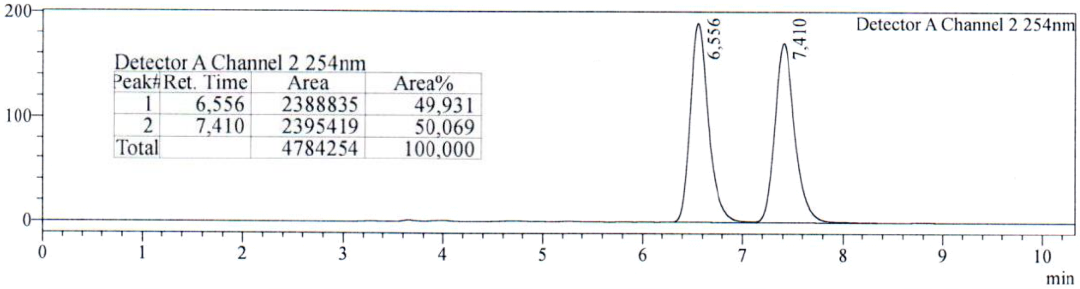

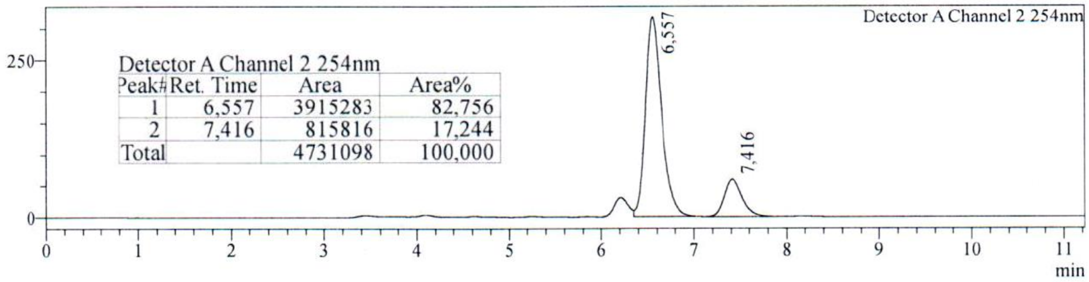

***S*-methyl *(S)*-2-benzamido-2-phenylethanethioate (*(S)*-5l)**

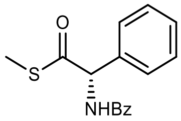

Prepared according to General Procedure 7 in 73% yield as a yellow foam.

Enantiomeric ratio = 86:14 was determined by chiral HPLC analysis: Chiralpak IC, *n*-heptane+0.1%IPA/IPA 70:30, 1 mL/min, 25 °C, detection at 254 nm, retention time (min): 9.0 (major) and 12.5 (minor).

[α]$_D^{20}$ = 1.08 (c = 1.0, $CHCl_3$).

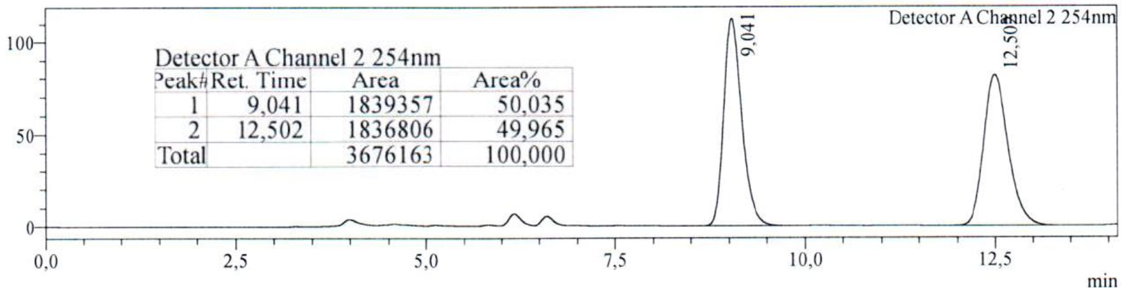

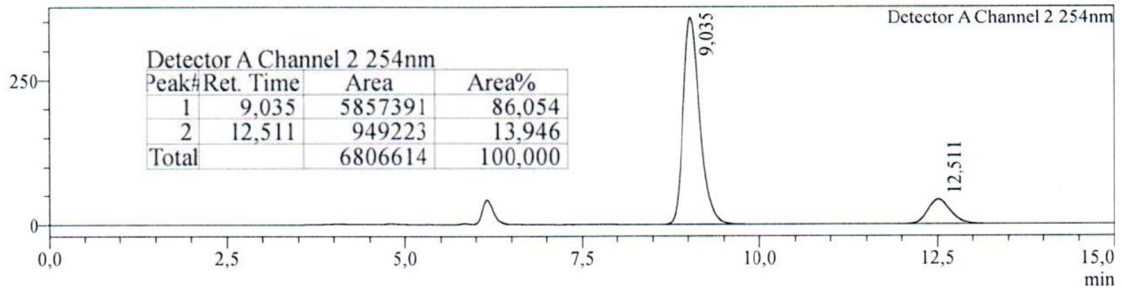

6. Post-functionalizations of α-amino amides and α-amino thioesters

2-Benzamido-3-cyclohexylpropanoic acid (6)

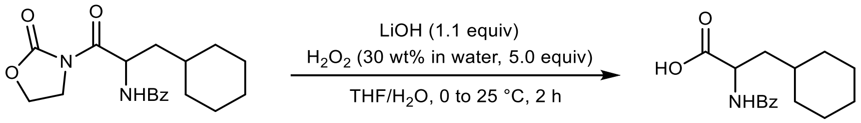

To a solution of α-amino amide **3d** (48 mg, 0.14 mmol, 1.00 equiv.) in 4.0 mL of THF/H_2O (3:1) at 0 °C, LiOH (3.8 mg, 0.16 mmol, 1.10 equiv.) and a solution of aqueous H_2O_2 30% in water (70 µL, 0.7 mmol, 5.00 equiv.) were added. The mixture was stirred at 25 °C for 2 h before 1.0 mL of aqueous $Na_2S_2O_3$ (1.5 M) was added. After 10 min, an aqueous solution of HCl 1.0 M was added dropwise until the pH reached 2.0. The crude product was extracted with ethyl acetate for three times, the organic phase was combined and dried by $MgSO_4$. Then, the solvent was evaporated and the residue was purified by column chromatography on silica gel (dichloromethane/methanol/formic acid) to give the desired product **6** with a yield of 95%.

^{1}H NMR (600 MHz, MeOD) δ 7.85 (d, *J* = 7.3 Hz, 2H), 7.54 (t, *J* = 7.4 Hz, 1H), 7.46 (t, *J* = 7.7 Hz, 2H), 4.68 (t, *J* = 7.6 Hz, 1H), 1.87 (d, *J* = 12.8 Hz, 1H), 1.81 – 1.69 (m, 5H), 1.69 – 1.60 (m, 1H), 1.51 – 1.40 (m, 1H), 1.34 – 1.13 (m, 3H), 1.08 – 0.88 (m, 2H). (The 2 exchangable protons are not observed)

^{13}C NMR (151 MHz, MeOD) δ 176.5, 170.4, 135.4, 132.8, 129.2 (2C), 128.5 (2C), 52.1, 40.0, 35.8, 34.9, 33.3, 27.6, 27.4, 27.2.

IR (neat) ν: 2921, 2850, 1716, 1630, 1576, 711 cm^{-1}.

HRMS (ESI$^+$): exact mass calculated for $[M+Na]^+$ ($C_{17}H_{22}N_2O_4Na^+$) requires m/z 298.1414, found 298.1415 m/z.

***N*-(1-Cyclohexyl-3-hydroxypropan-2-yl)benzamide (7)**

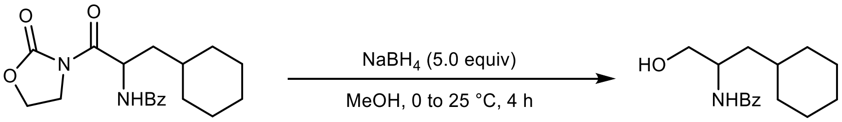

To a solution of α-amino amide **3d** (48.2 mg, 0.14 mmol, 1.00 equiv.) in 2.0 mL of MeOH at 0 °C, $NaBH_4$ (15.8 mg, 0.42 mmol, 3.00 equiv.) was added. After 2 h, another portion of $NaBH_4$ (5.3 mg, 0.14 mmol, 1.0 equiv.) was added. After an additional hour and another one portion of $NaBH_4$ (5.3 mg, 0.14 mmol, 1.0 equiv.) was added. The mixture was stirred for 1 h at 0 °C and then 1 h at 25 °C. Then, the reaction was quenched with acetone (2.0 mL). After the volatiles were evaporated, EtOAc and saturated aqueous NH_4Cl solution were introduced. The organic layer was separated, washed with brine, dried over anhydrous $MgSO_4$, and concentrated under vacuum. The crude product was purified by chromatography on silica gel (heptane/ethyl acetate) to give the final product **7** with a yield of 85%.

^{1}H NMR (600 MHz, $CDCl_3$) δ 7.80 – 7.73 (m, 2H), 7.50 (t, *J* = 7.4 Hz, 1H), 7.43 (t, *J* = 7.6 Hz, 2H), 6.28 (d, *J* = 7.5 Hz, 1H), 4.43 – 4.14 (m, 1H), 3.77 (dd, *J* = 11.0, 3.2Hz, 1H), 3.64 (dd, *J* = 11.0, 5.5 Hz, 1H), 2.90 (s, 1H), 1.83 (d, *J* = 12.9 Hz, 1H), 1.73 1.68 (m, 3H), 1.64 (d, *J* = 11.2 Hz, 1H), 1.51 – 1.46 (m, 2H), 1.41 – 1.31 (m, 1H), 1.27 – 1.11 (m, 3H), 1.03 – 0.85 (m, 2H).

^{13}C NMR (151 MHz, CDCl3) δ 168.4, 134.5, 131.8, 128.7 (2C), 127.1 (2C), 66.5, 50.0, 39.1, 34.6, 33.9, 33.1, 26.6, 26.4, 26.3.

IR (neat) ν: 3316, 2921, 2850, 1708, 1578, 711 cm^{-1}.

HRMS (ESI$^+$): exact mass calculated for $[M+Na]^+$ ($C_{17}H_{22}N_2O_4Na^+$) requires m/z 284.1621, found 284.1621 m/z.

Benzyl 2-benzamidohexanoate (8)

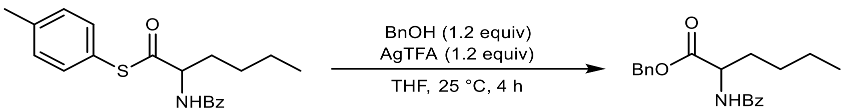

To a solution of α-amino thioester **5n** (34 mg, 0.10 mmol, 1.0 equiv.) and benzyl alcohol (12 µL, 0.12 mmol, 1.2 equiv.) in THF (1.0 mL), silver trifluoroacetate (27 mg, 0.12 mmol, 1.2 equiv.) was added. The mixture was stirred at 25 °C for 4 h. Then EtOAc (10 mL) was added and the mixture was passed through a pad of celite. The filtrate was concentrated to give the crude product. Purification by flash chromatography over silica gel gave the desired α-amino ester in 88% yield.

^{1}H NMR (600 MHz, $CDCl_3$) δ 7.83 – 7.76 (m, 2H), 7.54 – 7.50 (m, 1H), 7.44 (dd, *J* = 10.5, 4.7 Hz, 2H), 7.40 – 7.29 (m, 5H), 6.65 (d, *J* = 7.5 Hz, 1H), 5.22 (dd, *J* = 38.7, 12.2 Hz, 2H), 4.88 (td, *J* = 7.3, 5.4 Hz, 1H), 1.98 (ddt, *J* = 13.8, 10.6, 5.2 Hz, 1H), 1.80 (dddd, *J* = 13.7, 10.7, 7.0, 4.9 Hz, 1H), 1.41 – 1.26 (m, 4H), 0.85 (t, *J* = 7.2 Hz, 3H).

^{13}C NMR (150 MHz, $CDCl_3$) δ 172.6, 167.0, 135.3, 134.04 131.7, 128.7 (2C), 128.6 (2C), 128.5, 128.3 (2C), 127.0 (2C), 67.2, 52.6, 32.4, 27.2, 22.3, 13.8.

IR (neat) ν: 2958, 2930, 1741, 1666, 1537, 1488, 1454, 1379, 1365, 1352, 1230, 1216, 1189, 755 cm^{-1}.

HRMS (ESI$^+$): exact mass calculated for $[M+Na]^+$ ($C_{20}H_{23}NO_3Na^+$) requires m/z 348.1570, found 348.1573 m/z.

Methyl (2-benzamidohexanoyl)glycinate (9)

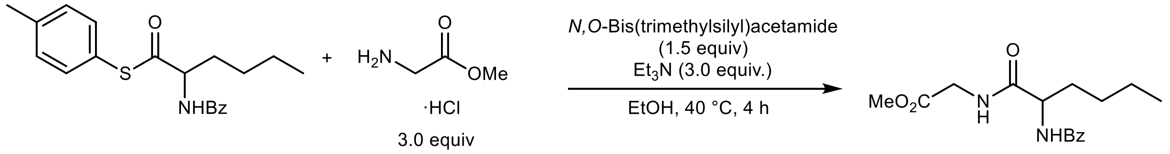

To a solution of α-amino thioester **5n** (34 mg, 0.10 mmol, 1.0 equiv.) and glycine methyl ester hydrochloride (38 mg, 0.30 mmol, 3.0 equiv.) in EtOH (1.0 mL), *N,O*-Bis(trimethylsilyl)acetamide (37 µL, 0.15 mmol, 1.5 equiv.) and triethylamine (42 µL, 0.30 mmol, 3.0 equiv.) were added. The mixture was stirred at 40 °C for 4 h. The solvent was evaporated under reduced pressure, and the residue dissolved in ethyl acetate (5.0 mL) then washed successively with 1.0 M aqueous HCl (1.0 mL), 2.0 M aqueous NaOH (1.0 mL), brine (1.0 mL). The combined organic layer was dried with sodium sulfate, filtered, and concentrated to dryness. The crude product was purified by flash chromatography on silica gel to give the title compound in 95% yield.

^{1}H NMR (700 MHz, $CDCl_3$) δ 7.84 – 7.76 (m, 2H), 7.49 (t, *J* = 7.4 Hz, 1H), 7.41 (t, *J* = 7.7 Hz, 2H), 7.15 (s, 1H), 7.04 (s, 1H), 4.74 (dd, *J* = 14.2, 7.4 Hz, 1H), 4.04 (ddd, *J* = 60.8, 18.1, 5.5 Hz, 2H), 3.72 (s, 3H), 2.01 – 1.92 (m, 1H), 1.75 (ddd, *J* = 15.4, 14.0, 7.6 Hz, 1H), 1.39 – 1.27 (m, 4H), 0.87 (t, *J* = 7.2 Hz, 3H).

^{13}C NMR (175 MHz, $CDCl_3$) δ 172.3, 170.0, 167.4, 133.7, 131.8, 128.5 (2C), 127.1 (2C), 53.4, 52.3, 41.2, 32.2, 27.6, 22.4, 13.9.

IR (neat) ν: 1758, 1636, 1536, 1489, 1367, 1214, 1204, 1180, 713, 693 cm^{-1}.

HRMS (ESI$^+$): exact mass calculated for $[M+Na]^+$ ($C_{16}H_{22}N_2O_4Na^+$) requires m/z 329.1472, found 329.1475 m/z.

***N*-(1-(4-Methoxyphenyl)-1-oxohexan-2-yl)benzamide (11)**

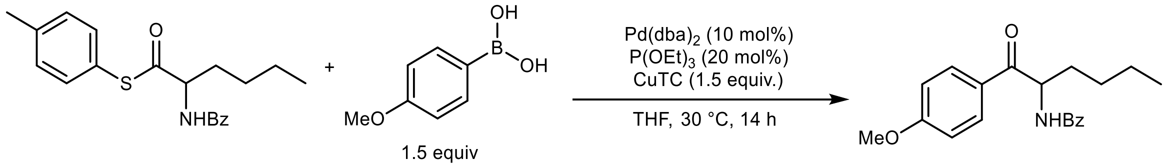

α-Amino thioester **5n** (34 mg, 0.10 mmol, 1.0 equiv.), 4-methoxyphenylboronic acid (23 mg, 0.15 mmol, 1.5 equiv.), CuTC (29 mg, 0.15 mmol, 1.5 equiv.), and $Pd(dba)_2$ (5.8 mg, 0.01 mmol, 10 mol%) were placed under an argon atmosphere. THF (1.0 mL) and $P(OEt)_3$ (3.5 µL, 0.02 mmol, 20 mol%) were added, and the mixture was stirred at 30 °C for 14 h before workup. The reaction mixture was diluted with dichloromethane (5.0 mL), washed with saturated aqueous $NaHCO_3$ (2.0 mL) and brine (2.0 mL), and

followed by drying over sodium sulfate. The filtrate was concentrated to give the crude product. Purification by flash chromatography over silica gel gave the tile compound in 81% yield.

^{1}H NMR (600 MHz, $CDCl_3$) δ 8.06 – 8.00 (m, 2H), 7.90 – 7.82 (m, 2H), 7.55 – 7.48 (m, 1H), 7.45 (dd, *J* = 10.4, 4.6 Hz, 2H), 7.27 – 7.22 (m, 1H), 7.03 – 6.95 (m, 2H), 5.78 (td, *J* = 7.3, 4.7 Hz, 1H), 3.89 (s, 3H), 2.09-2.04 (m, 1H), 1.72 (dddd, *J* = 13.9, 11.2, 7.1, 4.4 Hz, 1H), 1.43 – 1.36 (m, 1H), 1.35 – 1.23 (m, 3H), 0.83 (t, *J* = 7.1 Hz, 3H).

^{13}C NMR (150 MHz, $CDCl_3$) δ 197.5, 166.8, 164.2, 134.3, 131.6, 131.1 (2C), 128.6 (2C), 127.3, 127.1 (2C), 114.1 (2C), 55.5, 53.8, 33.6, 27.0, 22.5, 13.8.

IR (neat) ν: 1752, 1718, 1438, 1421, 1366, 1230, 1202, 761, 531.

HRMS (ESI$^+$): exact mass calculated for $[M+Na]^+$ ($C_{20}H_{23}NO_3Na^+$) requires m/z 348.1570, found 348.1570 m/z.

7. Computational details

7.1 Computational method

The conformational space of all molecules has been initially searched using meta-dynamics simulations based on tight-binding quantum chemical calculations as implemented in the software package Conformer-Rotamer Ensemble Sampling Tool (CREST) [18,19].

The structures located with CREST have then been subjected to a B3LYP-D3BJ/def2-SVP[20–29] geometry optimization. The nature of all stationary points (minima and transition states) was verified through the computation of harmonic vibrational frequencies. The thermal corrections to the Gibbs free energies were combined with the single point energies calculated at the B3LYP-D3BJ/def2-TZVP level of theory to yield Gibbs free energies ("G_{298}") at 298.15 K.

The DFT calculations have been performed with the Gaussian 16[30] program package. The SMD[31,32] model with parameters of dichloromethane was applied to consider implicit solvation effects in both, the geometries and energies. All energies are reported in kcal/mol. The energy profiles were constructed using the most stable conformation (the global minimum) of each intermediate and transition state.

Free energies in solution have been corrected to a reference state of 1 mol/L at 298.15 K through the addition of RTln(24.46) = +7.925 kJ/mol to the gas phase (1 atm) free energies.

7.2 Additional computational results

Figure S1 presents the proposed [2,3]-sigmatropic rearrangement from structure ***E*-B** and alternative pathways in comparison. Our calculations deny the possibility of S–O bond dissociations, either with the formation of an oxirenium ion (***E*-E**) or with a concerted attack of TfO^- to form a TfO adduct (***E*-E'**). Both steps would require an extremely high activation barrier of approx. 30 kcal/mol, which is in no way competitive to the barrier of 9.4 kcal/mol computed for the [2,3]-sigmatropic rearrangement. Furthermore, the attempts to obtain a pathway for the protonation of the oxazolidinone by the sulfinamine show that this pathway is computed to be kinetically and thermodynamically forbidden. One of the obtained energy scans probing the viability of this transformation is shown in Figure S2, with the decrease (in increments of 0.1 Å) of the distance between the proton from the sulfinamide moiety and the oxygen from the oxazolidinone group. Through the scan, there is no indication of a transition state for the proton transfer, and the energy increases by over 16 kcal/mol from the structure of intermediate ***E*-B**, which is already significantly higher than the barrier obtained for the proposed [2,3]-rearrangement.

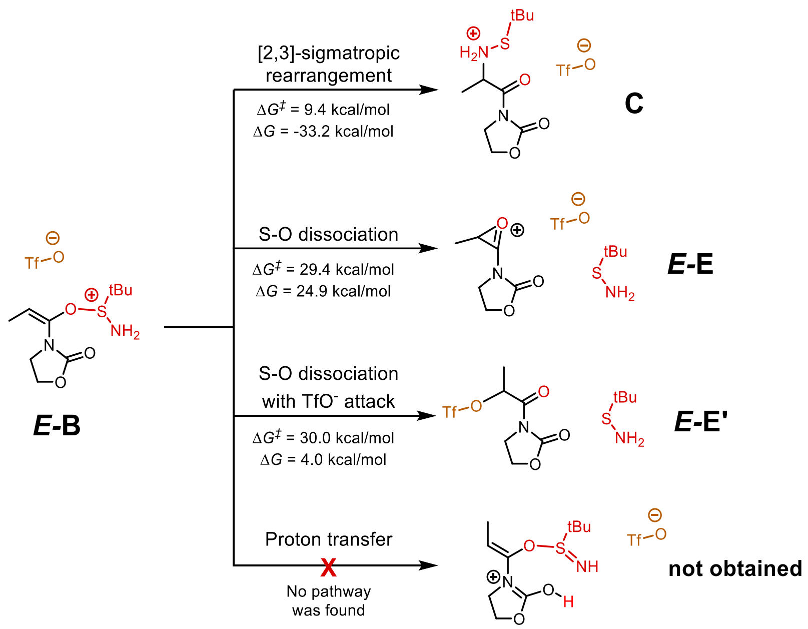

Figure S1 – Computationally studied alternative reaction pathways occurring from the intermediate ***E-B****.*

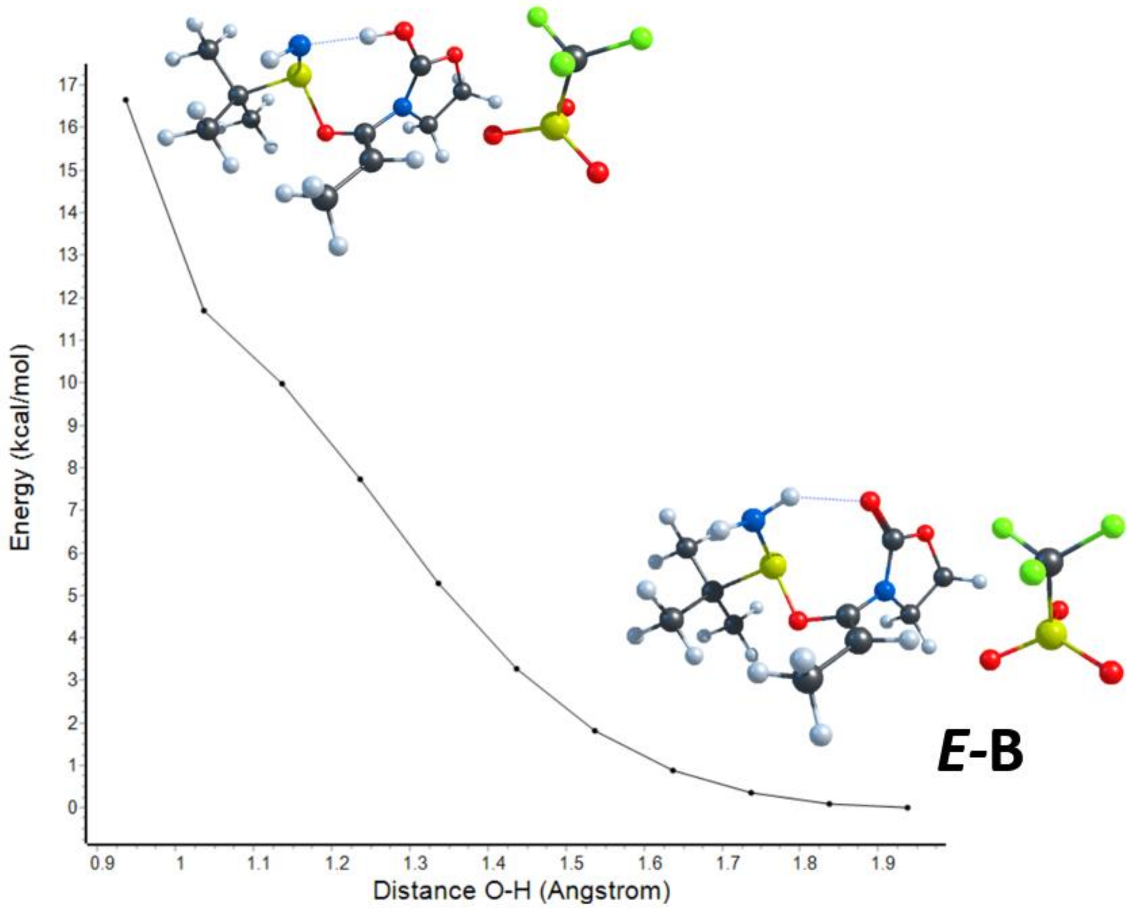

Figure S2 – Relaxed energy scan resulting from the attempted intramolecular protonation of the oxazolidine.

Considering that the most favorable profile leading to product formation involves intermediates with the *E*-configured carbon-carbon double bond, the possibility of obtaining selectivity for the product by using an enantiopure sulfinamide was briefly evaluated computationally. Applying (*R*)-sulfinamide as a reactant (a part of the reactant complex **A** as shown in Figure S3), the sigmatropic rearrangement, step **B-C**, can lead to an (*S*)-product via **TS**$_{E\text{-}\mathbf{BC(S)}}$ or an (*R*)-product via **TS**$_{E\text{-}\mathbf{BC(R)}}$. The resulting products **C** are enantiomers and, therefore, are energetically degenerate. The relatively small free energy gap between **TS**$_{E\text{-}\mathbf{BC(S)}}$ and **TS**$_{E\text{-}\mathbf{BC(R)}}$ ($\Delta\Delta G^{\ddagger}$ = 1.2 kcal/mol) strongly indicates that this reaction would present poor enantio-selectivity if an enantiopure sulfinamide is used.

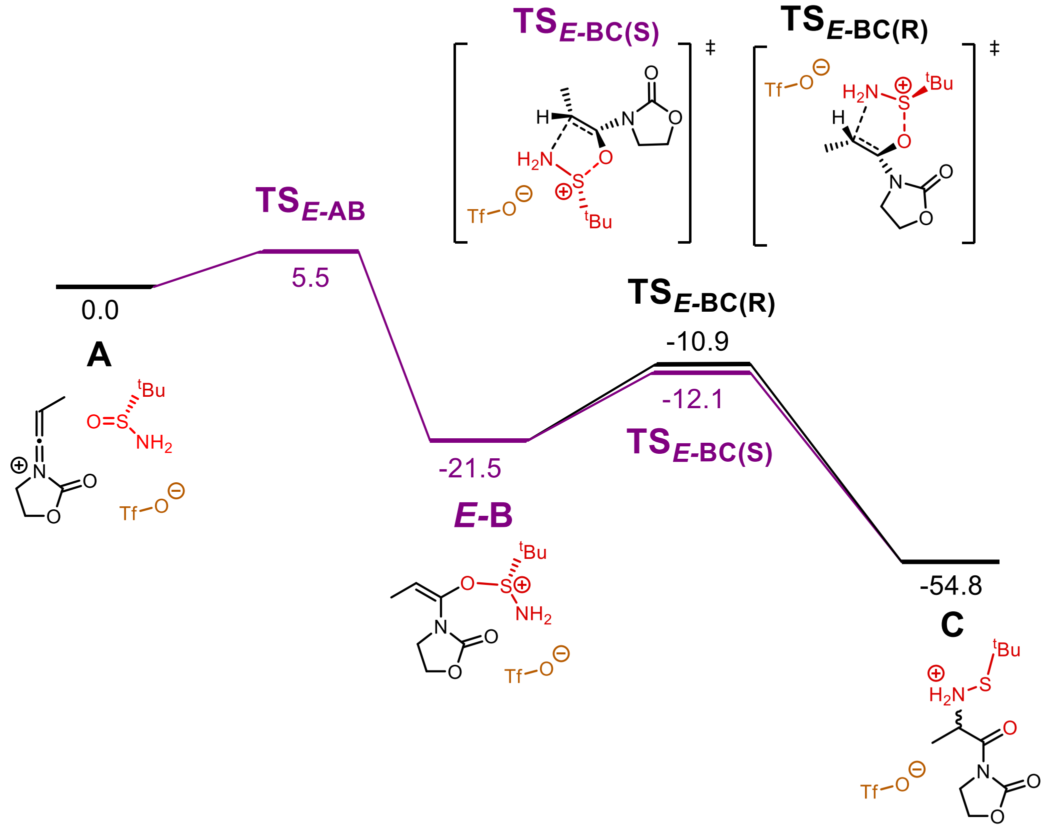

*Figure S3 – Computed Gibbs free energy profile for the interconversion of the reactant complex **A** (taken as a reference, 0.0 kcal/mol) into the enantiomeric products **C** considering intermediates with E-configured carbon-carbon double bond. The diastereomeric pathways are also shown.*

Finally, the possibility of sulfur extrusion by a base was considered. Given that triethylamine is added during the reaction workup, we have computed the pathway for S–N bond cleavage from structure **C** by a trimethylamine (for computational expediency), as shown in Figure S4. The single transition state obtained for this profile, structure **TS**$_{\mathbf{C'F}}$ shows that this is a S_N2-type step, that presents a very low activation barrier ($\Delta G^{\ddagger}$(**C'**→**F**) = 7.1 kcal/mol) and even though it is slightly endergonic (ΔG(**C'**→**F**) = 4.1 kcal/mol) it allows the amine protection step to form the observed product. Any attempts to obtain an equivalent step promoted by the triflate anion did not provide a viable pathway.

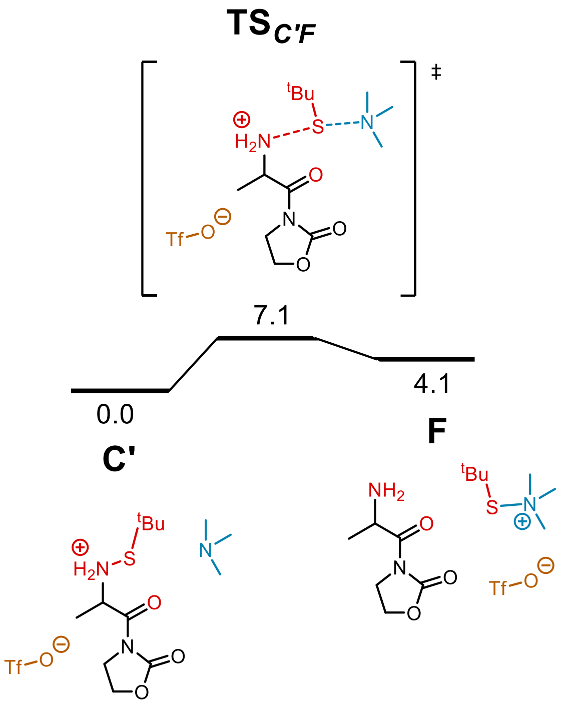

*Figure S4 – Computed Gibbs free energy profile for the proposed sulfur extrusion step. The reactant complex **C'** is taken as reference (0.0 kcal/mol).*

7.3 Cartesian coordinates (XYZ)

Cartesian coordinates for the most stable conformations ($\Delta G_{298,DCM}$) as computed at the B3LYP-D3BJ/def2-TZVP,SMD(DCM)//B3LYP-D3BJ/def2-SVP,SMD(DCM) level of theory

```
43
A
O       -2.082799000     0.470656000    -2.488077000
C       -0.531824000     2.805230000     1.783883000
C        0.196726000     2.764996000     2.854269000
H        0.470211000     1.744060000     3.192460000
C       -0.903662000     2.944010000    -0.652692000
C       -2.213695000     3.392993000    -1.310119000
H       -0.624205000     1.932667000    -0.974512000
H       -0.074611000     3.646585000    -0.795461000
H       -2.468600000     2.751614000    -2.161496000
H       -2.217351000     4.456098000    -1.586055000
N       -1.287104000     2.890431000     0.775445000
O       -3.240173000     3.203585000    -0.300792000
C       -2.769225000     2.925376000     0.891330000
O       -3.362088000     2.769770000     1.909303000
S       -2.921577000    -0.441064000    -1.602075000
N       -2.615284000     0.038613000    -0.001053000
H       -1.676017000    -0.165988000     0.375412000
H       -3.355525000    -0.274767000     0.629674000
C       -2.091225000    -2.148063000    -1.753965000
C        0.680783000     3.951843000     3.638162000
H        0.345899000     4.905979000     3.211504000
H        0.316470000     3.863000000     4.674196000
H        1.781776000     3.928990000     3.674348000
C       -2.629400000    -3.026738000    -0.624849000
H       -2.314503000    -4.070125000    -0.794888000
H       -2.237165000    -2.716840000     0.355209000
H       -3.732236000    -3.019355000    -0.587130000
C       -2.544016000    -2.651944000    -3.129644000
H       -3.638180000    -2.778544000    -3.178235000
H       -2.239043000    -1.957360000    -3.927803000
H       -2.082088000    -3.631645000    -3.335513000
C       -0.578003000    -1.973461000    -1.693101000
H       -0.234004000    -1.268225000    -2.464073000
H       -0.244001000    -1.610664000    -0.711402000
H       -0.090434000    -2.946548000    -1.873176000
S        0.567137000    -0.852687000     2.197695000
O        0.740735000    -0.229519000     3.532092000
O        0.029852000     0.088870000     1.167574000
O       -0.024675000    -2.200295000     2.183099000
C        2.326230000    -1.115940000     1.618441000
F        2.969053000    -1.951313000     2.439016000
F        2.351142000    -1.636906000     0.386648000
F        2.991505000     0.044215000     1.594636000

43
E-AB
O        2.712878000    -0.188783000    -1.267112000
C        1.786912000     2.586171000    -1.717159000
C        1.736736000     2.026673000    -2.882519000
H        2.093751000     0.977196000    -2.802179000
C        2.941855000     3.824252000     0.083060000
C        2.532665000     3.760691000     1.556588000
H        2.946666000     4.849664000    -0.311142000
H        3.895649000     3.326741000    -0.127271000
H        2.681483000     4.708922000     2.085525000
H        3.024696000     2.941630000     2.098475000
N        1.826334000     3.086071000    -0.556526000
O        1.107304000     3.481636000     1.539861000
C        0.688335000     2.997706000     0.391147000
O       -0.384179000     2.569477000     0.112386000
S        1.818219000    -1.378892000    -0.908033000
N        0.231119000    -0.832919000    -1.021924000
H       -0.459954000    -1.596834000    -1.128795000
H       -0.052283000    -0.169582000    -0.296955000
C        2.108632000    -1.619809000     0.954854000
C        1.278820000     2.608831000    -4.184026000
H        0.444237000     1.999743000    -4.566869000
H        0.958564000     3.653991000    -4.087764000
H        2.099500000     2.536914000    -4.915443000
```

C	1.201875000	-2.766241000	1.399200000
H	0.133794000	-2.544127000	1.263746000
H	1.425573000	-3.694889000	0.848337000
H	1.369491000	-2.968895000	2.470537000
C	3.590263000	-1.992580000	1.070004000
H	4.237124000	-1.179560000	0.708370000
H	3.838788000	-2.189982000	2.125982000
H	3.825030000	-2.904735000	0.496326000
C	1.806648000	-0.311340000	1.680129000
H	2.115650000	-0.395009000	2.735365000
H	2.363201000	0.522835000	1.225546000
H	0.731413000	-0.072019000	1.675216000
S	-2.771303000	-3.160153000	-0.150218000
O	-2.103845000	-3.713247000	1.046911000
O	-4.006012000	-3.840914000	-0.577869000
O	-1.855758000	-2.784054000	-1.264475000
C	-3.384054000	-1.493285000	0.449742000
F	-2.358944000	-0.720306000	0.856796000
F	-4.028968000	-0.832273000	-0.519294000
F	-4.221991000	-1.632263000	1.483671000

43
Z-AB

O	2.406344000	0.650928000	1.019585000
C	2.270425000	-2.094732000	0.815964000
C	1.656639000	-2.611285000	1.832296000
H	1.559715000	-3.707717000	1.775761000
C	4.320437000	-1.328412000	-0.282885000
C	4.460849000	-0.986318000	-1.766328000
H	4.952054000	-2.165091000	0.036442000
H	4.456549000	-0.455266000	0.367867000
H	4.856712000	-1.820283000	-2.361845000
H	5.052227000	-0.081012000	-1.941903000
N	2.890827000	-1.697740000	-0.207688000
O	3.108630000	-0.726389000	-2.221405000
C	2.189691000	-1.204297000	-1.408902000
O	1.014053000	-1.251852000	-1.569452000
S	1.096854000	1.373924000	1.348685000
N	-0.131228000	0.319114000	0.899703000
H	-0.238858000	0.193919000	-0.111822000
H	-1.047775000	0.600874000	1.277795000
C	0.992259000	2.772251000	0.084976000
C	1.079409000	-1.889521000	3.011641000
H	1.488384000	-0.877342000	3.092495000
H	-0.012895000	-1.827925000	2.884172000
H	1.293458000	-2.463472000	3.925002000
C	2.141391000	3.711962000	0.452563000
H	2.038835000	4.094183000	1.481480000
H	3.112334000	3.202110000	0.365378000
H	2.141583000	4.577541000	-0.229556000
C	1.161035000	2.204271000	-1.317808000
H	0.329114000	1.541780000	-1.597844000
H	1.174785000	3.035113000	-2.042233000
H	2.111986000	1.661144000	-1.411636000
C	-0.370991000	3.430427000	0.287167000
H	-0.444940000	4.316401000	-0.364397000
H	-1.192782000	2.748916000	0.030193000
H	-0.506515000	3.768433000	1.327777000
S	-3.241690000	0.497327000	-0.376612000
O	-2.940217000	0.930754000	1.016852000
O	-2.120806000	0.678010000	-1.331506000
O	-4.577230000	0.866027000	-0.871698000
C	-3.335553000	-1.362116000	-0.203973000
F	-3.589293000	-1.941620000	-1.382804000
F	-2.178184000	-1.860425000	0.259296000
F	-4.304026000	-1.716304000	0.649323000

43
E-B

O	2.334668000	-0.819842000	-0.564956000
C	1.640809000	-1.907530000	-1.087943000
C	1.045939000	-1.797623000	-2.284764000
H	1.087781000	-0.812547000	-2.759380000
C	2.782565000	-3.900458000	0.015087000
C	2.107908000	-5.019721000	0.816178000
H	3.528145000	-3.346694000	0.610324000
H	3.256978000	-4.268392000	-0.904849000
H	2.700094000	-5.367224000	1.672062000
H	1.836854000	-5.878282000	0.182060000
N	1.617558000	-3.061842000	-0.281461000
O	0.889828000	-4.422825000	1.315322000
C	0.592230000	-3.319199000	0.607447000
O	-0.419456000	-2.671154000	0.773828000
S	1.552989000	-0.073697000	0.792357000
N	0.042114000	0.284693000	0.387980000
H	-0.591682000	-0.505133000	0.558843000
H	-0.216239000	0.930614000	-0.406300000
C	2.508958000	1.558822000	0.730444000
C	0.349522000	-2.892205000	-3.020040000
H	0.773121000	-3.004596000	-4.032814000
H	0.414108000	-3.856918000	-2.497659000
H	-0.716265000	-2.637830000	-3.156454000
C	2.505146000	2.133195000	-0.682581000
H	3.029595000	1.479529000	-1.392306000
H	1.489017000	2.332210000	-1.048668000
H	3.041157000	3.095652000	-0.642727000
C	1.787802000	2.462618000	1.735525000
H	2.411229000	3.360855000	1.872451000
H	0.803928000	2.782548000	1.363608000
H	1.675957000	1.984113000	2.721803000
C	3.915435000	1.174961000	1.203359000
H	4.399359000	0.460337000	0.521823000
H	4.524367000	2.092440000	1.220090000
H	3.908710000	0.756650000	2.222008000
S	-1.515058000	3.143698000	-1.261886000
O	-1.922779000	3.976566000	-2.401474000
O	-0.886017000	3.835041000	-0.118448000
O	-0.831313000	1.863081000	-1.651568000
C	-3.130550000	2.513746000	-0.556638000
F	-3.850089000	1.885648000	-1.491625000
F	-2.900859000	1.648805000	0.442244000
F	-3.861768000	3.522345000	-0.073246000

43
Z-B

O	-2.327131000	0.841515000	-0.748174000
C	-1.723130000	1.995086000	-1.246887000
C	-1.119522000	1.992890000	-2.443899000
H	-0.746740000	2.963276000	-2.785827000
C	-3.018402000	3.905830000	-0.161623000
C	-2.433245000	5.062039000	0.657659000
H	-3.751302000	3.312358000	0.410760000
H	-3.482937000	4.246294000	-1.097164000
H	-3.068073000	5.372643000	1.496980000
H	-2.196763000	5.936025000	0.030786000
N	-1.799697000	3.133981000	-0.420296000
O	-1.195823000	4.538439000	1.190013000
C	-0.813624000	3.455312000	0.490424000
O	0.232340000	2.871662000	0.680901000
S	-1.585674000	0.223401000	0.683419000
N	-0.048859000	-0.119646000	0.375182000
H	0.263178000	-0.807961000	-0.362325000
H	0.556835000	0.694383000	0.531085000
C	-2.497506000	-1.433650000	0.715625000
C	-0.926724000	0.821197000	-3.345900000
H	-1.405430000	1.009660000	-4.322718000
H	0.147564000	0.672253000	-3.549245000
H	-1.333330000	-0.107417000	-2.926366000
C	-3.928986000	-1.053648000	1.111117000
H	-4.513592000	-1.984124000	1.183592000
H	-3.967752000	-0.554170000	2.091783000
H	-4.408178000	-0.410733000	0.358538000
C	-2.430576000	-2.120015000	-0.644956000
H	-1.397628000	-2.318905000	-0.959508000
H	-2.941204000	-3.091563000	-0.542307000
H	-2.949686000	-1.543098000	-1.421727000
C	-1.789596000	-2.236903000	1.811751000
H	-0.786821000	-2.559231000	1.497302000
H	-1.722263000	-1.681348000	2.761011000
H	-2.396093000	-3.137685000	1.998366000
S	1.608965000	-3.061314000	-1.053567000
O	0.945081000	-1.796101000	-1.520279000
O	2.098090000	-3.917823000	-2.142402000
O	0.906838000	-3.729964000	0.060629000
C	3.168777000	-2.403547000	-0.254282000
F	2.866062000	-1.516374000	0.704767000
F	3.869520000	-3.395485000	0.303080000
F	3.948202000	-1.793381000	-1.152346000

```
43
E-BC
O       -2.322376000     1.595454000    -0.203590000
C       -1.165123000     1.983574000    -0.633620000
C       -0.454998000     1.267276000    -1.574564000
H       -0.967813000     0.394184000    -1.983824000
C       -1.289685000     4.363583000     0.242661000
C       -0.215794000     5.158365000     0.989661000
H       -2.193331000     4.198032000     0.852622000
H       -1.577399000     4.834068000    -0.707649000
H       -0.612557000     5.794397000     1.790966000
H        0.402071000     5.764030000     0.308129000
N       -0.570508000     3.106668000     0.023396000
O        0.624702000     4.143835000     1.583528000
C        0.434473000     2.970076000     0.955615000
O        1.070613000     1.969139000     1.227315000
S       -1.855089000     0.077245000     1.269687000
N       -0.457693000    -0.370876000     0.621593000
H        0.355727000     0.193932000     0.919763000
H       -0.315170000    -1.116064000    -0.123001000
C       -3.131052000    -1.220469000     0.764257000
C        0.879556000     1.592602000    -2.134294000
H        1.281351000     2.546760000    -1.768710000
H        1.586165000     0.781048000    -1.881104000
H        0.828390000     1.617615000    -3.237431000
C       -3.142674000    -1.435479000    -0.746329000
H       -3.951860000    -2.150603000    -0.968075000
H       -3.351294000    -0.501222000    -1.284128000
H       -2.203730000    -1.871983000    -1.113888000
C       -2.763037000    -2.493162000     1.535989000
H       -2.732819000    -2.323641000     2.623449000
H       -3.541942000    -3.246497000     1.332923000
H       -1.796757000    -2.905323000     1.210677000
C       -4.440845000    -0.601118000     1.267835000
H       -5.253369000    -1.317284000     1.068790000
H       -4.420051000    -0.411637000     2.352807000
H       -4.676135000     0.336487000     0.742205000
S        1.549475000    -2.449792000    -1.402448000
O        1.972390000    -2.978087000    -2.704714000
O        0.071513000    -2.183296000    -1.294818000
O        2.375796000    -1.385358000    -0.796059000
C        1.759215000    -3.889452000    -0.225926000
F        1.108417000    -4.967546000    -0.671735000
F        3.049907000    -4.204016000    -0.089650000
F        1.273200000    -3.579875000     0.984381000

43
Z-BC
O        2.274531000     1.587884000     0.581705000
C        1.111779000     2.036523000     0.887754000
C        0.249855000     1.440117000     1.791675000
H       -0.718891000     1.927919000     1.939406000
C        1.490713000     4.334398000    -0.127521000
C        0.470385000     5.252595000    -0.806131000
H        2.326363000     4.064984000    -0.793979000
H        1.895729000     4.766136000     0.798191000
H        0.881553000     5.825764000    -1.646563000
H       -0.012106000     5.938065000    -0.091735000
N        0.644276000     3.172596000     0.150295000
O       -0.534707000     4.348670000    -1.317429000
C       -0.445322000     3.163316000    -0.688833000
O       -1.224419000     2.251063000    -0.897053000
S        1.758566000     0.279871000    -1.159491000
N        0.311656000    -0.140202000    -0.597715000
H       -0.460842000     0.503373000    -0.845616000
H        0.085497000    -1.008374000    -0.026070000
C        2.928471000    -1.186524000    -0.922323000
C        0.584031000     0.318222000     2.701342000
H        0.510672000     0.674562000     3.745948000
H       -0.146507000    -0.502272000     2.603599000
H        1.592427000    -0.077297000     2.533513000
C        2.943675000    -1.669571000     0.523375000
H        3.663695000    -2.502243000     0.583957000
H        3.275539000    -0.877558000     1.207917000
H        1.963203000    -2.050649000     0.840604000
C        2.451628000    -2.269521000    -1.897639000
H        1.473055000    -2.684205000    -1.613445000
H        2.401907000    -1.899454000    -2.933694000
H        3.191387000    -3.087157000    -1.869609000
C        4.274037000    -0.582976000    -1.344674000
H        4.255077000    -0.212266000    -2.381855000
H        4.584866000     0.233311000    -0.675025000
H        5.033506000    -1.378456000    -1.287674000
S       -1.147835000    -3.405981000     0.252947000
O       -1.337729000    -4.547056000     1.156460000
O       -0.711733000    -3.695122000    -1.126144000
O       -0.424381000    -2.244272000     0.885434000
C       -2.864650000    -2.695585000     0.031072000
F       -2.815527000    -1.578435000    -0.707487000
F       -3.660408000    -3.571367000    -0.588156000
F       -3.412285000    -2.391049000     1.211074000

43
C
O        0.696046000     1.956426000    -2.044014000
C       -0.317926000     1.668077000    -1.447324000
C       -1.271455000     0.566889000    -1.982962000
H       -2.288005000     0.984928000    -2.035966000
C        0.237810000     3.430602000     0.199827000
C       -0.639598000     4.081884000     1.271235000
H        0.520242000     4.123591000    -0.602038000
H        1.144001000     2.966519000     0.613627000
H       -1.130061000     5.001395000     0.919281000
H       -0.108749000     4.280546000     2.210144000
N       -0.660780000     2.385104000    -0.305381000
O       -1.673415000     3.102737000     1.537668000
C       -1.709718000     2.178693000     0.586624000
O       -2.550477000     1.302558000     0.544430000
S       -2.246079000    -2.020580000    -1.538358000
N       -1.333678000    -0.557109000    -0.983913000
H       -1.823229000    -0.154505000    -0.155129000
H       -0.350525000    -0.846562000    -0.688652000
C       -2.851200000    -2.736758000     0.092957000
C       -0.808777000     0.093987000    -3.351287000
H       -0.757888000     0.952469000    -4.034402000
H        0.188261000    -0.366349000    -3.299644000
H       -1.523743000    -0.627177000    -3.772433000
C       -3.996368000    -1.890525000     0.656171000
H       -4.830841000    -1.818555000    -0.057808000
H       -4.372633000    -2.364906000     1.578301000
H       -3.673159000    -0.872532000     0.919067000
C       -3.359766000    -4.114858000    -0.361408000
H       -2.541282000    -4.753632000    -0.727142000
H       -3.824833000    -4.615700000     0.502971000
H       -4.128075000    -4.034013000    -1.148069000
C       -1.703338000    -2.878961000     1.095521000
H       -0.844575000    -3.415882000     0.667736000
H       -1.356663000    -1.906489000     1.474545000
H       -2.067930000    -3.449866000     1.965848000
S        1.410027000    -0.734043000     1.258097000
O        1.141445000    -1.175330000    -0.154221000
O        1.678125000    -1.827651000     2.201064000
O        0.484970000     0.328702000     1.715378000
C        3.058371000     0.132243000     1.089941000
F        3.455888000     0.601032000     2.275752000
F        3.988091000    -0.709587000     0.634519000
F        2.972071000     1.162209000     0.239474000

43
E-AD
O       -1.573778000    -1.769292000     1.486272000
C       -0.165367000     1.510910000     1.114467000
C       -0.003145000     0.715613000     2.123717000
H       -0.383159000    -0.315858000     2.009344000
C        0.783726000     2.816717000    -0.767221000
C        0.271477000     4.190263000    -1.188780000
H        0.739519000     2.065157000    -1.566520000
H        1.789840000     2.810919000    -0.338192000
H        0.339827000     4.365476000    -2.268243000
H        0.755632000     5.010794000    -0.640705000
N       -0.213926000     2.459867000     0.268672000
O       -1.135786000     4.188945000    -0.826596000
C       -1.418013000     3.274828000     0.081891000
O       -2.452654000     3.097447000     0.643591000
S       -1.563422000    -1.801822000    -0.034145000
N       -1.061354000    -0.248345000    -0.562782000
H       -1.760951000     0.284167000    -1.080135000
H       -0.157723000    -0.300962000    -1.061803000
```

```
C      -3.386001000     -1.709142000     -0.524881000
C       0.666995000      1.131736000      3.401559000
H       0.932747000      2.196735000      3.415779000
H       0.007114000      0.900691000      4.252014000
H       1.582537000      0.527981000      3.512294000
C      -3.964137000     -3.031771000     -0.014951000
H      -3.825990000     -3.130261000      1.071533000
H      -5.043585000     -3.063763000     -0.231935000
H      -3.492472000     -3.896668000     -0.508752000
C      -4.021009000     -0.522316000      0.191775000
H      -5.113421000     -0.554690000      0.051010000
H      -3.807472000     -0.567436000      1.269808000
H      -3.666324000      0.446946000     -0.189768000
C      -3.466423000     -1.618390000     -2.046106000
H      -3.076162000     -0.664903000     -2.432953000
H      -2.915823000     -2.441169000     -2.530142000
H      -4.521398000     -1.693808000     -2.355610000
S       2.766451000     -0.217503000     -0.755382000
O       2.748857000      0.784116000      0.335456000
O       4.037521000     -0.365040000     -1.479885000
O       1.555279000     -0.209753000     -1.631381000
C       2.588027000     -1.832392000      0.164698000
F       3.607511000     -2.009565000      1.009894000
F       1.451089000     -1.848336000      0.879371000
F       2.557342000     -2.866379000     -0.680654000

43
Z-AD
O      -1.339893000     -2.045003000      0.707029000
C       0.300469000      1.759528000      1.271483000
C       0.649786000      1.133998000      2.354956000
H       1.183957000      1.817946000      3.036174000
C       1.390390000      2.777315000     -0.694378000
C       1.076589000      4.177586000     -1.209825000
H       1.246181000      1.993412000     -1.447348000
H       2.381908000      2.671888000     -0.241873000
H       1.201272000      4.277467000     -2.293681000
H       1.642158000      4.962667000     -0.688363000
N       0.333929000      2.623539000      0.331970000
O      -0.331231000      4.369883000     -0.903219000
C      -0.749136000      3.553113000      0.046294000
O      -1.817799000      3.546197000      0.577212000
S      -1.828895000     -1.017630000     -0.288417000
N      -1.843574000      0.484777000      0.571045000
H      -2.435183000      0.436212000      1.406959000
H      -2.189180000      1.235007000     -0.031584000
C      -3.684049000     -1.384558000     -0.454568000
C       0.483714000     -0.285598000      2.765193000
H      -0.146457000     -0.848188000      2.067425000
H       1.488729000     -0.736383000      2.770909000
H       0.085252000     -0.335000000      3.790582000
C      -3.716450000     -2.723597000     -1.195908000
H      -3.209879000     -3.508340000     -0.614938000
H      -4.761928000     -3.031970000     -1.358216000
H      -3.229134000     -2.651353000     -2.182201000
C      -4.298987000     -1.504722000      0.932348000
H      -5.326265000     -1.895804000      0.851179000
H      -3.712229000     -2.197664000      1.553907000
H      -4.359483000     -0.531836000      1.445912000
C      -4.301561000     -0.267353000     -1.290715000
H      -4.315899000      0.692528000     -0.751753000
H      -3.762252000     -0.127722000     -2.242472000
H      -5.345012000     -0.524675000     -1.535815000
S       2.411081000     -0.437294000     -0.447834000
O       3.129552000      0.109554000      0.726338000
O       3.082578000     -0.250325000     -1.748889000
O       0.950013000     -0.159369000     -0.465953000
C       2.480708000     -2.285214000     -0.184702000
F       3.743663000     -2.721850000     -0.282757000
F       2.022783000     -2.627195000      1.024957000
F       1.749264000     -2.922967000     -1.104103000

43
E-D
O      -2.239854000     -1.563011000      1.939923000
C      -0.440691000      0.493167000      0.587463000
C      -0.133669000      0.581567000      1.895761000
H       0.025031000     -0.358068000      2.428188000
C      -0.027054000      1.859446000     -1.503540000
C      -0.399765000      3.331012000     -1.703952000
H      -0.402116000      1.223179000     -2.322433000
H       1.050899000      1.693773000     -1.399085000
H      -0.648671000      3.580428000     -2.742739000
H       0.388307000      4.009089000     -1.341028000
N      -0.732850000      1.574113000     -0.249621000
O      -1.574836000      3.525684000     -0.891496000
C      -1.672184000      2.549995000      0.034885000
O      -2.474945000      2.562937000      0.935415000
S      -1.987387000     -2.008737000      0.539356000
N      -0.480018000     -0.798309000     -0.026083000
H      -0.423888000     -0.766017000     -1.051482000
H       0.377790000     -1.356437000      0.278155000
C      -3.414191000     -1.364015000     -0.534065000
C       0.021529000      1.850747000      2.655347000
H       0.267007000      2.695704000      1.995318000
H      -0.913749000      2.105279000      3.183280000
H       0.813644000      1.745310000      3.412113000
C      -4.265912000     -2.633491000     -0.686909000
H      -4.577068000     -3.037188000      0.289597000
H      -5.178154000     -2.372551000     -1.246598000
H      -3.736624000     -3.420649000     -1.246358000
C      -4.147114000     -0.278282000      0.236794000
H      -5.000127000      0.056230000     -0.375907000
H      -4.540232000     -0.659441000      1.189286000
H      -3.516014000      0.594275000      0.450647000
C      -2.879214000     -0.894211000     -1.883132000
H      -2.376706000      0.079613000     -1.806328000
H      -2.196623000     -1.626693000     -2.345265000
H      -3.731750000     -0.774048000     -2.569906000
S       2.720114000     -1.326729000     -0.562664000
O       1.910144000     -0.683819000     -1.625016000
O       3.807272000     -2.210381000     -0.992903000
O       1.873792000     -1.867263000      0.555971000
C       3.601739000      0.108634000      0.246720000
F       4.444945000      0.678037000     -0.618019000
F       2.728062000      1.043008000      0.650002000
F       4.295000000     -0.300552000      1.309794000

43
Z-D
O       1.782187000     -0.975093000      1.425509000
C       0.897819000      0.490323000     -0.939585000
C      -0.176605000     -0.308650000     -1.080180000
H      -1.150622000      0.152434000     -0.903136000
C      -0.172600000      2.247787000      0.541575000
C       0.168924000      3.738889000      0.621883000
H      -0.037965000      1.713135000      1.494574000
H      -1.192358000      2.069218000      0.178954000
H       0.182755000      4.129819000      1.646392000
H      -0.499020000      4.353452000      0.000380000
N       0.838022000      1.794090000     -0.420406000
O       1.507311000      3.848542000      0.076911000
C       1.816166000      2.742064000     -0.602180000
O       2.840146000      2.616180000     -1.255526000
S       2.969887000     -0.354195000      0.777434000
N       2.236437000      0.040408000     -1.116141000
H       2.308196000     -0.788246000     -1.716798000
H       2.830212000      0.826676000     -1.462668000
C       4.175512000     -1.709739000      0.280803000
C      -0.185147000     -1.752875000     -1.436781000
H       0.810056000     -2.196503000     -1.572911000
H      -0.702606000     -2.309142000     -0.638412000
H      -0.771425000     -1.913699000     -2.356963000
C       4.881425000     -2.006778000      1.612940000
H       4.177595000     -2.391356000      2.366087000
H       5.646100000     -2.778510000      1.431562000
H       5.390293000     -1.117703000      2.017975000
C       3.415351000     -2.923196000     -0.231180000
H       4.115345000     -3.770118000     -0.306589000
H       2.607040000     -3.206861000      0.458210000
H       2.990346000     -2.758249000     -1.232158000
C       5.134990000     -1.094079000     -0.736203000
H       4.669564000     -0.954885000     -1.723276000
H       5.538175000     -0.127742000     -0.391369000
H       5.988148000     -1.777993000     -0.867499000
S      -3.463159000      0.215555000      0.623175000
O      -3.073633000      1.133718000     -0.484988000
O      -4.553468000      0.703826000      1.485336000
O      -2.326115000     -0.432911000      1.313260000
```

C -4.250925000 -1.199537000 -0.309578000
F -5.313406000 -0.785632000 -1.010375000
F -3.381423000 -1.751298000 -1.172998000
F -4.657345000 -2.160438000 0.528997000

43
E-BC(S)
O 2.322376000 1.595454000 -0.203590000
C 1.165123000 1.983574000 -0.633620000
C 0.454998000 1.267276000 -1.574564000
H 0.967813000 0.394184000 -1.983824000
C 1.289685000 4.363583000 0.242661000
C 0.215794000 5.158365000 0.989661000
H 2.193331000 4.198032000 0.852622000
H 1.577399000 4.834068000 -0.707649000
H 0.612557000 5.794397000 1.790966000
H -0.402071000 5.764030000 0.308129000
N 0.570508000 3.106668000 0.023396000
O -0.624702000 4.143835000 1.583528000
C -0.434473000 2.970076000 0.955615000
O -1.070613000 1.969139000 1.227315000
S 1.855089000 0.077245000 1.269687000
N 0.457693000 -0.370876000 0.621593000
H -0.355727000 0.193932000 0.919763000
H 0.315170000 -1.116064000 -0.123001000
C 3.131052000 -1.220469000 0.764257000
C -0.879556000 1.592602000 -2.134294000
H -1.281351000 2.546760000 -1.768710000
H -1.586165000 0.781048000 -1.881104000
H -0.828390000 1.617615000 -3.237431000
C 3.142674000 -1.435479000 -0.746329000
H 3.951860000 -2.150603000 -0.968075000
H 3.351294000 -0.501222000 -1.284128000
H 2.203730000 -1.871983000 -1.113888000
C 2.763037000 -2.493162000 1.535989000
H 2.732819000 -2.323641000 2.623449000
H 3.541942000 -3.246497000 1.332923000
H 1.796757000 -2.905323000 1.210677000
C 4.440845000 -0.601118000 1.267835000
H 5.253369000 -1.317284000 1.068790000
H 4.420051000 -0.411637000 2.352807000
H 4.676135000 0.336487000 0.742205000
S -1.549475000 -2.449792000 -1.402448000
O -1.972390000 -2.978087000 -2.704714000
O -0.071513000 -2.183296000 -1.294818000
O -2.375796000 -1.385358000 -0.796059000
C -1.759215000 -3.889452000 -0.225926000
F -1.108417000 -4.967546000 -0.671735000
F -3.049907000 -4.204016000 -0.089650000
F -1.273200000 -3.579875000 0.984381000

43
E-BC(R)
O -1.821876000 -0.129136000 -1.229500000
C -1.441122000 1.098137000 -0.992324000
C -0.120942000 1.455937000 -1.050419000
H 0.549574000 0.654128000 -1.364877000
C -3.665635000 2.292861000 -1.265009000
C -4.229144000 3.439388000 -0.419109000
H -3.459786000 2.594058000 -2.300778000
H -4.324444000 1.409046000 -1.269936000
H -3.965770000 4.426581000 -0.828620000
H -5.312959000 3.380171000 -0.262518000
N -2.427569000 2.018432000 -0.537577000
O -3.575890000 3.294433000 0.863338000
C -2.486699000 2.520645000 0.734564000
O -1.717493000 2.308395000 1.657025000
S -1.106766000 -1.301584000 0.374332000
N -0.399886000 -0.130997000 1.195255000
H 0.651179000 -0.029978000 1.151141000
H -0.928587000 0.670927000 1.598579000
C -2.736143000 -1.692419000 1.217666000
C 0.478714000 2.779309000 -0.766283000
H -0.266299000 3.565240000 -0.587014000
H 1.134089000 3.077097000 -1.602888000
H 1.139875000 2.694098000 0.113895000
C -3.543978000 -0.452073000 1.560245000
H -3.827658000 0.103056000 0.658143000
H -3.019947000 0.211795000 2.260454000
H -4.469390000 -0.785845000 2.055418000
C -2.336786000 -2.478942000 2.470802000
H -1.720858000 -3.358939000 2.229681000
H -3.260078000 -2.834480000 2.956167000
H -1.791809000 -1.844863000 3.185322000
C -3.446203000 -2.571681000 0.185410000
H -3.679635000 -2.004581000 -0.727026000
H -4.390451000 -2.924728000 0.627563000
H -2.848707000 -3.457394000 -0.082792000
S 2.789453000 -0.301776000 -0.176684000
O 1.822987000 -1.222525000 -0.820639000
O 3.524878000 0.612171000 -1.060795000
O 2.237708000 0.337005000 1.064906000
C 4.093492000 -1.443170000 0.509915000
F 3.549456000 -2.313062000 1.365561000
F 5.034591000 -0.749450000 1.154579000
F 4.676050000 -2.129627000 -0.476543000

38
E-SMe(S)
O 2.230779000 -1.862572000 0.217326000
C 1.120141000 -2.505852000 0.439059000
C 0.177277000 -2.019174000 1.314821000
H 0.450477000 -1.106287000 1.848435000
S 1.756068000 -0.400042000 -1.179724000
N 0.241310000 -0.149022000 -0.736518000
H -0.509209000 -0.588066000 -1.272836000
H -0.069047000 0.417647000 0.098355000
C 2.734543000 0.968745000 -0.344100000
C -1.138797000 -2.625816000 1.632245000
H -1.258578000 -3.634296000 1.211321000
H -1.937775000 -1.978995000 1.226087000
H -1.294459000 -2.666980000 2.723595000
C 2.410078000 1.059024000 1.141807000
H 3.053505000 1.845985000 1.566522000
H 2.626319000 0.115859000 1.658431000
H 1.367322000 1.350177000 1.326333000
C 2.355846000 2.248041000 -1.094735000
H 2.562530000 2.171538000 -2.173160000
H 2.967465000 3.069724000 -0.688770000
H 1.296371000 2.503483000 -0.948669000
C 4.186566000 0.555155000 -0.593753000
H 4.409471000 0.460324000 -1.668087000
H 4.427645000 -0.391209000 -0.088540000
H 4.838725000 1.340920000 -0.182810000
S -2.372428000 1.049490000 0.875218000
O -3.347885000 1.213415000 1.957132000
O -0.934879000 1.137606000 1.305019000
O -2.616833000 -0.039797000 -0.098895000
C -2.543634000 2.590643000 -0.163805000
F -2.358160000 3.686370000 0.574954000
F -3.754659000 2.655094000 -0.720377000
F -1.629709000 2.587553000 -1.145828000
S 0.726554000 -3.891237000 -0.635174000
C 2.399454000 -4.365262000 -1.175430000
H 2.890183000 -3.541744000 -1.709365000
H 2.265725000 -5.215905000 -1.858996000
H 3.014927000 -4.678266000 -0.319826000

38
E-SMe(R)
O -1.682001000 -0.107469000 1.327383000
C -1.733399000 -1.355550000 0.972833000
C -0.584048000 -2.066600000 0.703975000
H 0.351011000 -1.532746000 0.886949000
S -0.942626000 1.039442000 -0.277890000
N -0.542666000 -0.164736000 -1.250890000
H 0.481713000 -0.441743000 -1.302370000
H -1.223944000 -0.591516000 -1.882100000
C -2.492545000 1.813255000 -1.007072000
C -0.481434000 -3.479662000 0.261885000
H 0.123081000 -4.057714000 0.983855000
H 0.059413000 -3.537649000 -0.699223000
H -1.457833000 -3.969934000 0.147118000
C -3.139065000 2.514826000 0.187391000
H -2.444175000 3.220178000 0.670423000
H -3.488518000 1.791116000 0.935408000
H -4.001887000 3.091821000 -0.179006000
C -3.403965000 0.777414000 -1.645160000

H	-2.955824000	0.329133000	-2.544972000
H	-4.321335000	1.294733000	-1.967456000
H	-3.683088000	-0.022372000	-0.945862000
C	-1.953948000	2.813335000	-2.037429000
H	-1.282607000	3.555715000	-1.580453000
H	-2.814758000	3.350157000	-2.468384000
H	-1.420399000	2.304410000	-2.853638000
S	2.822722000	0.488171000	-0.895213000
O	4.212770000	0.437372000	-1.362701000
O	2.052655000	1.716514000	-1.171209000
O	2.036996000	-0.770060000	-1.146153000
C	2.967551000	0.478624000	0.966782000
F	3.646560000	-0.588739000	1.393284000
F	1.746308000	0.445030000	1.529015000
F	3.590239000	1.576975000	1.399571000
S	-3.341246000	-2.111498000	0.663955000
C	-4.395954000	-1.079827000	1.730280000
H	-4.515146000	-0.067576000	1.322176000
H	-3.989376000	-1.029712000	2.749990000
H	-5.376831000	-1.576011000	1.753554000

43

E-BE

O	2.267569000	-1.642565000	-2.275951000
C	1.243457000	-2.026591000	-1.516147000
H	4.270111000	-1.304868000	-1.883030000
C	3.294018000	-0.984039000	-1.501413000
C	3.032555000	-1.432115000	-0.062769000
H	3.176752000	-0.612227000	0.650618000
N	1.615199000	-1.811269000	-0.154322000
H	3.634655000	-2.306970000	0.222856000
H	3.167658000	0.099940000	-1.613378000
C	0.812490000	-1.916738000	0.911655000
C	1.190915000	-1.831376000	2.306441000
H	0.713934000	-1.010121000	2.848876000
C	1.972520000	-2.844245000	3.003603000
H	2.311471000	-3.669134000	2.364437000
H	2.858081000	-2.305258000	3.409830000
H	1.440094000	-3.213291000	3.897942000
O	-0.372629000	-2.251545000	1.017547000
S	-2.362587000	-1.317091000	-0.767494000
N	-1.889536000	-0.162291000	-1.948259000
H	-1.003524000	0.284915000	-1.677170000
C	-3.572789000	-0.323420000	0.242966000
C	-4.832492000	-0.077890000	-0.589811000
H	-5.328746000	-1.024031000	-0.856774000
H	-5.549217000	0.539444000	-0.020315000
H	-4.583038000	0.458192000	-1.518304000
C	-2.928221000	0.996205000	0.661710000
H	-3.616114000	1.556465000	1.318290000
H	-1.980204000	0.834114000	1.193714000
H	-2.716708000	1.620942000	-0.216749000
C	-3.867028000	-1.208335000	1.457115000
H	-4.616385000	-0.717595000	2.099680000
H	-4.273513000	-2.188441000	1.157225000
H	-2.958736000	-1.378816000	2.055247000
S	1.151395000	1.492435000	0.169215000
O	0.497492000	0.868024000	1.344261000
O	2.613328000	1.677271000	0.296612000
O	0.712114000	0.959423000	-1.147797000
C	0.507725000	3.244200000	0.171037000
F	-0.818808000	3.275709000	0.011694000
F	0.805013000	3.844360000	1.326991000
F	1.064234000	3.941481000	-0.822486000
H	-1.749380000	-0.646079000	-2.835845000
O	0.223300000	-2.502565000	-1.917015000

43

E-E

O	2.550255000	-1.012876000	-2.432906000
C	1.748796000	-1.648512000	-1.599947000
H	4.451347000	-0.216878000	-2.263786000
C	3.487704000	-0.145312000	-1.747990000
C	3.533463000	-0.673371000	-0.311945000
H	3.545552000	0.137513000	0.424715000
N	2.253986000	-1.401493000	-0.256072000
H	4.353533000	-1.385234000	-0.145774000
H	3.087067000	0.874255000	-1.791597000
C	1.626702000	-1.747661000	0.823712000
C	1.538589000	-1.785702000	2.248888000
H	1.039606000	-0.918200000	2.692644000
C	2.267069000	-2.767744000	3.090532000
H	2.677628000	-3.600439000	2.503178000
H	3.097005000	-2.228818000	3.578850000
H	1.604880000	-3.149347000	3.881882000
O	0.579067000	-2.399015000	1.115099000
S	-2.216745000	-1.569437000	-0.622877000
N	-1.791599000	-0.407402000	-1.826200000
H	-0.929744000	0.080406000	-1.562358000
C	-3.766963000	-0.807593000	0.076342000
C	-4.854310000	-0.763526000	-0.997092000
H	-5.121972000	-1.776913000	-1.335805000
H	-5.765981000	-0.276771000	-0.607743000
H	-4.507894000	-0.188060000	-1.869463000
C	-3.450682000	0.598385000	0.586017000
H	-4.344152000	1.047340000	1.054704000
H	-2.631281000	0.582435000	1.319826000
H	-3.139221000	1.245887000	-0.245215000
C	-4.161455000	-1.736003000	1.227952000
H	-5.087921000	-1.372805000	1.703047000
H	-4.345806000	-2.764000000	0.874491000
H	-3.376036000	-1.772437000	2.000114000
S	0.857041000	1.535664000	0.333986000
O	0.144117000	0.735220000	1.355343000
O	2.229018000	1.948599000	0.703314000
O	0.729512000	1.016544000	-1.056741000
C	-0.072895000	3.150882000	0.276958000
F	-1.327812000	2.969424000	-0.142210000
F	-0.109742000	3.707461000	1.490299000
F	0.529445000	3.999401000	-0.559829000
H	-1.647846000	-0.873503000	-2.721451000
O	0.812617000	-2.334903000	-1.862485000

43

E-BE'

O	-0.738486000	-3.169291000	-1.854756000
C	-1.462490000	-2.598005000	-0.876294000
H	1.104056000	-4.116601000	-1.876506000
C	0.662823000	-3.180166000	-1.515519000
C	0.667431000	-3.051402000	0.001474000
H	1.527398000	-2.475723000	0.352730000
N	-0.609740000	-2.361219000	0.220669000
H	0.654203000	-4.034858000	0.496277000
H	1.146396000	-2.317096000	-1.994553000
C	-0.925225000	-1.532918000	1.288632000
C	0.002725000	-1.299114000	2.366111000
H	-0.225089000	-0.376415000	2.900007000
C	0.923940000	-2.237280000	3.015271000
H	0.991018000	-3.226288000	2.551491000
H	1.925116000	-1.766813000	3.042009000
H	0.628768000	-2.341567000	4.076161000
O	-2.024712000	-0.964706000	1.403549000
S	-2.758095000	0.662527000	-0.423063000
N	-1.789382000	0.643927000	-1.813761000
H	-0.788865000	0.507532000	-1.606308000
C	-2.432029000	2.373136000	0.242506000
C	-3.091165000	3.379934000	-0.703817000
H	-4.179831000	3.227638000	-0.762209000
H	-2.905245000	4.402623000	-0.333659000
H	-2.669686000	3.299562000	-1.716830000
C	-0.930262000	2.618709000	0.353286000
H	-0.759931000	3.607733000	0.810016000
H	-0.431275000	1.860943000	0.971963000
H	-0.450605000	2.623643000	-0.634390000
C	-3.102809000	2.367375000	1.618530000
H	-2.996693000	3.364410000	2.074978000
H	-4.179541000	2.143471000	1.546813000
H	-2.636749000	1.627958000	2.286526000
S	2.001961000	0.128699000	-0.108436000
O	1.444962000	0.243892000	1.274695000
O	3.060522000	-0.885402000	-0.264901000
O	0.980591000	0.141024000	-1.183509000
C	2.920071000	1.744467000	-0.318656000
F	2.105118000	2.791335000	-0.171831000
F	3.892527000	1.836234000	0.589084000
F	3.463479000	1.799253000	-1.535220000
H	-2.124200000	-0.081634000	-2.449671000
O	-2.633556000	-2.355129000	-0.980909000

43
E-E'

O	2.012290000	2.904111000	-0.104229000
C	1.177001000	2.474799000	0.857085000
H	1.717106000	3.608346000	-2.024825000
C	1.407655000	2.762160000	-1.400032000
C	-0.093765000	2.741198000	-1.115534000
H	-0.617200000	2.045473000	-1.776743000
N	-0.100104000	2.264967000	0.274685000
H	-0.537493000	3.743660000	-1.193308000
H	1.754708000	1.817851000	-1.843150000
C	-1.061455000	1.499751000	0.910456000
C	-2.555916000	1.671686000	0.554350000
H	-2.984844000	2.036084000	1.499493000
C	-3.047259000	2.549410000	-0.575269000
H	-2.752402000	2.183256000	-1.566548000
H	-4.145266000	2.585309000	-0.533175000
H	-2.670824000	3.573495000	-0.445866000
O	-0.792526000	0.737331000	1.815249000
S	2.441906000	-1.873958000	0.222058000
N	1.423616000	-0.487835000	0.082195000
H	0.682424000	-0.645125000	-0.600139000
C	4.100353000	-1.165048000	-0.256956000
C	4.465427000	-0.019668000	0.687312000
H	4.499407000	-0.361232000	1.733525000
H	5.457698000	0.386247000	0.423873000
H	3.735887000	0.800001000	0.615162000
C	4.050896000	-0.679854000	-1.705570000
H	5.021546000	-0.242927000	-1.997868000
H	3.821657000	-1.506398000	-2.396098000
H	3.281245000	0.096987000	-1.828172000
C	5.071827000	-2.338591000	-0.102572000
H	6.088998000	-2.018104000	-0.381851000
H	5.105222000	-2.700878000	0.937501000
H	4.794231000	-3.182822000	-0.754598000
S	-2.681784000	-0.674120000	-0.701882000
O	-3.151337000	0.334971000	0.463209000
O	-3.817556000	-1.018186000	-1.534826000
O	-1.417440000	-0.221786000	-1.283887000
C	-2.317246000	-2.177961000	0.366588000
F	-1.194214000	-1.998628000	1.036097000
F	-3.321364000	-2.379574000	1.202178000
F	-2.192891000	-3.214841000	-0.449871000
H	0.988413000	-0.267793000	0.979488000
O	1.482076000	2.364560000	2.009177000

56
C'

O	-0.927960000	1.517063000	2.479929000
C	-0.695403000	2.032081000	1.404851000
C	-1.878731000	2.442500000	0.516064000
H	-2.719377000	1.873882000	0.924511000
C	1.690028000	1.975772000	1.997829000
C	2.934957000	2.311021000	1.173420000
H	1.640699000	0.908768000	2.242539000
H	1.595665000	2.581612000	2.910628000
H	3.410557000	1.411636000	0.764843000
H	3.668055000	2.924924000	1.710257000
N	0.615517000	2.323566000	1.059809000
O	2.441741000	3.083248000	0.053265000
C	1.133898000	2.953236000	-0.069071000
O	0.513743000	3.383932000	-1.025662000
S	-1.405963000	0.255084000	-1.183152000
N	-1.720250000	2.018535000	-0.910857000
H	-0.883831000	2.546161000	-1.280603000
H	-2.536000000	2.328112000	-1.458507000
C	-3.122777000	-0.387464000	-1.537884000
C	-2.171806000	3.938179000	0.598237000
H	-2.323647000	4.214872000	1.651378000
H	-1.351054000	4.539191000	0.184485000
H	-3.097065000	4.168699000	0.047807000
C	-2.840448000	-1.891328000	-1.684489000
H	-2.486985000	-2.329025000	-0.736380000
H	-3.779336000	-2.390994000	-1.973405000
H	-2.095323000	-2.091088000	-2.470585000
C	-4.088434000	-0.127870000	-0.382047000
H	-5.033796000	-0.658251000	-0.584061000
H	-3.686059000	-0.496792000	0.571497000
H	-4.336115000	0.940289000	-0.279093000
C	-3.642664000	0.208527000	-2.846083000
H	-3.793197000	1.300100000	-2.782858000
H	-2.959514000	-0.002050000	-3.682316000
H	-4.625365000	-0.234340000	-3.079187000
S	1.678332000	-0.964125000	-0.364146000
O	1.680579000	0.413916000	-0.915027000
O	0.752141000	-1.906043000	-1.028395000
O	1.729850000	-1.047371000	1.115906000
C	3.360562000	-1.605896000	-0.858291000
F	3.507866000	-1.591908000	-2.187013000
F	4.334546000	-0.851285000	-0.326248000
F	3.533907000	-2.861824000	-0.432079000
N	-1.792468000	-3.063771000	1.501966000
C	-2.915725000	-3.732674000	2.123866000
H	-3.219355000	-4.608618000	1.527989000
H	-3.778116000	-3.049272000	2.188189000
C	-0.655807000	-3.948037000	1.316496000
H	-0.956173000	-4.828929000	0.725384000
H	-0.237150000	-4.317607000	2.282298000
C	-1.422150000	-1.849145000	2.205017000
H	-0.562956000	-1.378640000	1.712403000
H	-1.140347000	-2.037110000	3.267554000
H	-2.259921000	-1.135568000	2.206505000
H	-2.692540000	-4.088201000	3.157380000
H	0.140127000	-3.421619000	0.772125000

56
C’F

O	0.100670000	-1.276457000	1.920027000
C	-0.062426000	-1.049064000	0.737682000
C	0.036727000	-2.167867000	-0.305099000
H	0.199731000	-3.049410000	0.331260000
C	-0.716337000	1.227274000	1.408728000
C	-1.245997000	2.393924000	0.577898000
H	0.185770000	1.476689000	1.989394000
H	-1.481584000	0.811269000	2.073247000
H	-0.885142000	3.375158000	0.910158000
H	-2.343136000	2.379748000	0.536418000
N	-0.392422000	0.257736000	0.361070000
O	-0.731069000	2.151020000	-0.751883000
C	-0.351921000	0.870966000	-0.885027000
O	0.005838000	0.411061000	-1.945507000
S	3.138875000	-0.071763000	-0.142386000
N	1.239188000	-2.006893000	-1.125853000
H	1.070881000	-1.281470000	-1.824655000
H	1.425361000	-2.878881000	-1.623226000
C	4.535755000	-1.276703000	0.300287000
C	-1.272243000	-2.366567000	-1.078458000
H	-2.125489000	-2.439687000	-0.389431000
H	-1.459501000	-1.535993000	-1.771641000
H	-1.205297000	-3.296125000	-1.666848000
C	5.528945000	-0.738562000	1.328839000
H	5.028069000	-0.397838000	2.245651000
H	6.199159000	-1.569154000	1.603438000
H	6.165158000	0.065554000	0.935409000
C	3.769526000	-2.454535000	0.927055000
H	4.506070000	-3.231699000	1.189011000
H	3.242500000	-2.151056000	1.843278000
H	3.037995000	-2.876525000	0.229475000
C	5.236810000	-1.715808000	-0.986196000
H	4.513089000	-2.114161000	-1.712226000
H	5.788325000	-0.892097000	-1.460817000
H	5.961006000	-2.513281000	-0.749656000
S	-4.294668000	-0.082451000	0.987310000
O	-5.584773000	-0.724191000	1.300972000
O	-3.090039000	-0.896608000	1.273292000
O	-4.185180000	1.346966000	1.367225000
C	-4.319272000	-0.003181000	-0.882933000
F	-4.388393000	-1.227632000	-1.423553000
F	-5.371772000	0.698445000	-1.321436000
F	-3.210259000	0.590845000	-1.363023000
N	3.811182000	1.695735000	-0.148742000
C	4.934770000	1.865986000	-1.112826000
H	4.616035000	1.501132000	-2.095494000
H	5.807412000	1.307652000	-0.762973000
C	2.620118000	2.462714000	-0.637819000
H	2.288133000	2.045298000	-1.594763000
H	2.918170000	3.511732000	-0.761396000
C	4.179859000	2.213946000	1.201634000
H	3.369976000	1.981319000	1.901909000

H	4.309657000	3.302239000	1.119315000
H	5.114702000	1.759273000	1.532937000
H	5.179872000	2.936127000	-1.166260000
H	1.824480000	2.391161000	0.110881000

56
F

O	2.634822000	0.025655000	1.978690000
C	2.891415000	-0.438927000	0.877191000
C	4.093548000	0.084694000	0.078785000
H	4.508387000	0.833181000	0.769127000
C	1.173417000	-2.147881000	1.339431000
C	0.807310000	-3.414570000	0.560555000
H	0.304816000	-1.496606000	1.495598000
H	1.643939000	-2.362066000	2.307326000
H	-0.272924000	-3.593160000	0.511227000
H	1.316240000	-4.311460000	0.944597000
N	2.139553000	-1.518893000	0.433380000
O	1.283115000	-3.175483000	-0.778829000
C	2.067085000	-2.098600000	-0.837414000
O	2.603159000	-1.732640000	-1.856670000
S	-0.120698000	1.274683000	0.039683000
N	3.684589000	0.796650000	-1.129040000
H	3.411013000	0.113117000	-1.836677000
H	4.488791000	1.306509000	-1.496874000
C	-0.106012000	2.188934000	-1.595255000
C	5.154779000	-1.009081000	-0.116862000
H	5.420514000	-1.484241000	0.841878000
H	4.815252000	-1.785790000	-0.815970000
H	6.067415000	-0.552507000	-0.532485000
C	1.014575000	3.218454000	-1.711519000
H	1.982298000	2.781245000	-1.425727000
H	1.077131000	3.545961000	-2.762841000
H	0.814046000	4.114200000	-1.105417000
C	0.172795000	0.990283000	-2.524766000
H	0.111772000	1.351110000	-3.564246000
H	1.175657000	0.576220000	-2.356484000
H	-0.566424000	0.187798000	-2.389801000
C	-1.475717000	2.786510000	-1.923495000
H	-2.271410000	2.034520000	-1.836139000
H	-1.728584000	3.639995000	-1.280987000
H	-1.460125000	3.151609000	-2.964028000
S	-2.264461000	-1.591743000	0.268950000
O	-1.202112000	-1.331088000	-0.732946000
O	-1.981332000	-1.103139000	1.640636000
O	-2.856448000	-2.943365000	0.195490000
C	-3.673451000	-0.482985000	-0.278376000
F	-3.454351000	0.798859000	0.052139000
F	-3.836931000	-0.538565000	-1.606401000
F	-4.820595000	-0.858084000	0.298670000
N	-0.037161000	2.471916000	1.462038000
C	-1.080628000	3.531473000	1.375892000
H	-2.053871000	3.056881000	1.209697000
H	-0.835772000	4.213379000	0.555563000
C	-0.324423000	1.570853000	2.630394000
H	-1.271477000	1.048938000	2.452660000
H	-0.367228000	2.193878000	3.533149000
C	1.317635000	3.076509000	1.637832000
H	2.054560000	2.269054000	1.699813000
H	1.308417000	3.655445000	2.572056000
H	1.536138000	3.737511000	0.796171000
H	-1.082952000	4.084843000	2.325156000
H	0.491333000	0.842276000	2.705899000

8. X-Ray Crystallographic data for compound 3a and (*S*)-5k

The X-ray intensity data was measured on Bruker D8 Venture diffractometer equipped with multilayer monochromator, Mo K/α INCOATEC micro focus sealed tube and Oxford cooling system. The structure was solved by *Direct Methods*. Non-hydrogen atoms were refined with *anisotropic displacement parameters*. Hydrogen atoms were inserted at calculated positions and refined with riding model. The following software was used: *Bruker SAINT software package*[33] using a narrow-frame algorithm for frame integration, *SADABS*[34] for absorption correction, *OLEX2*[35] for structure solution, refinement, molecular diagrams and graphical user-interface, *Shelxle*[36] for refinement and graphical user-interface *SHELXS-2015*[37] for structure solution, *SHELXL-2015*[37] for refinement, *Platon*[38] for symmetry check. Experimental data and CCDC-Codes Experimental data (Available online: http://www.ccdc.cam.ac.uk/conts/retrieving.html) can be found in Supplementary Table 4. Crystal data, data collection parameters, and structure refinement details are given in Supplementary Table 5. Asymmetric Unit visualized in Figure S5, H-Bonds in Figure S6.

Supplementary Table 4. Experimental parameter and CCDC-Code of compound **3a**.

**Sample**	**Machine**	**Source**	**Temp.**	**Detector Distance**	**Time/ Frame**	**#Frames**	**Frame width**	**CCDC**
	Bruker		[K]	[mm]	[s]		[°]	
**3a**	D8	Mo	100	40	10	623	0.360	2171644

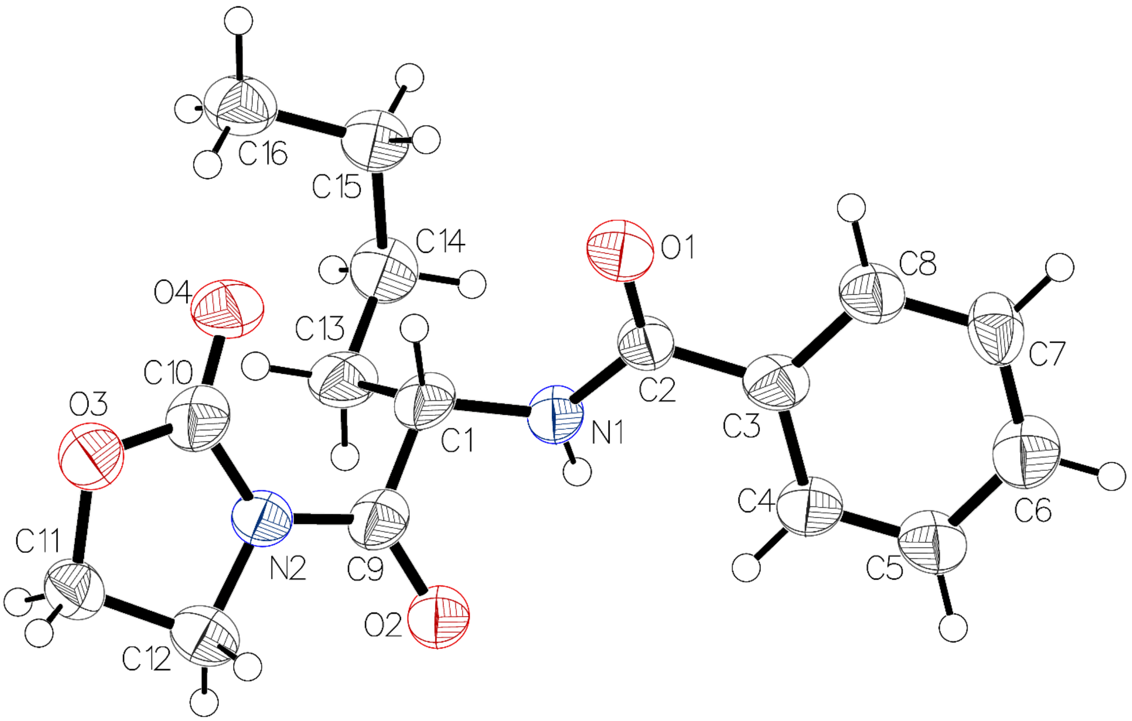

Figure S5 Asymmetric Unit is drawn with 50% displacement ellipsoid. The bond precision for C-C single bonds is 0.0084 Å.

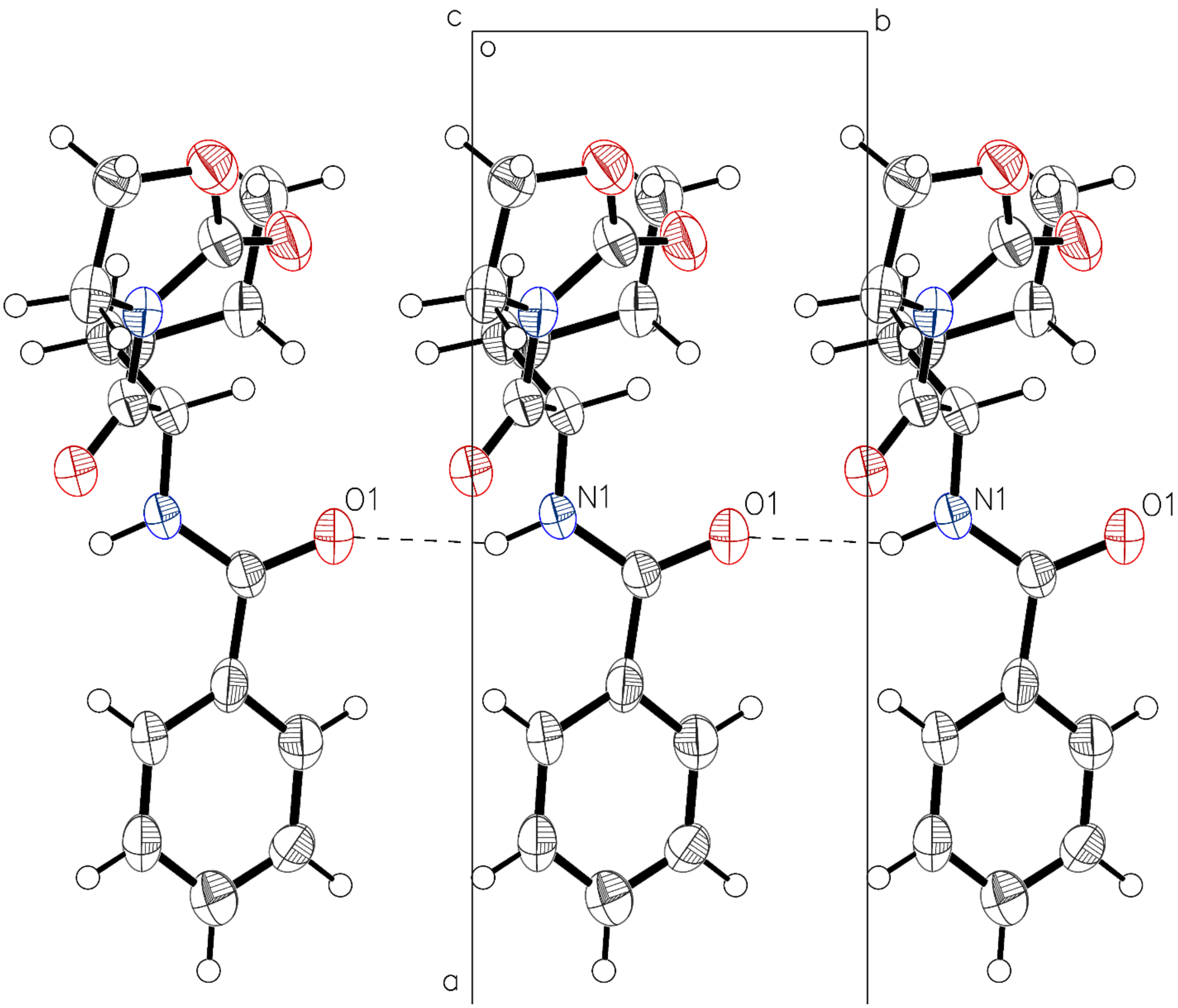

Figure S6 One intermolecular hydrogen bond of moderate character (according to Jeffrey 1997) is located at N1H to O1. This results in the one dimensional chain along axis b.

Supplementary Table 5. Sample and crystal data, Data collection and structure refinement

Identification code	MF_198_c2c
Empirical formula	$C_{16}H_{20}N_2O_4$
Formula weight	304.34
Temperature/K	100.00
Crystal system	monoclinic
Space group	C2/c

a/Å	22.674(7)
b/Å	5.1412(12)
c/Å	25.909(9)
α/°	90
β/°	97.083(13)
γ/°	90
Volume/Å^3	2997.2(15)
Z	8
ρ_{calc}g/cm^3	1.349
μ/mm^{-1}	0.098
F(000)	1296.0
Crystal size/mm^3	0.1 × 0.03 × 0.01
Radiation	MoKα (λ = 0.71073)
2Θ range for data collection/°	5.098 to 60.03
Index ranges	-31 ≤ h ≤ 27, -6 ≤ k ≤ 6, -34 ≤ l ≤ 33
Reflections collected	10543
Independent reflections	4036 [R_{int} = 0.1695, R_{sigma} = 0.3050]
Data/restraints/parameters	4036/0/200
Goodness-of-fit on F^2	0.997
Final R indexes [I>=2σ (I)]	R_1 = 0.1281, wR_2 = 0.3117
Final R indexes [all data]	R_1 = 0.2866, wR_2 = 0.3941
Largest diff. peak/hole / e Å^{-3}	0.38/-0.40

Supplementary Table 6. Experimental parameter and CCDC-Code of compound **(*S*)-5k**.

Sample	Machine	Source	Temp.	Detector Distance	Time/ Frame	#Frames	Frame width	CCDC
			[K]	[mm]	[s]		[°]	
**(*S*)-5k**	Stoe Stadivari	Mo	100	50	10	5354	0.4	2208631

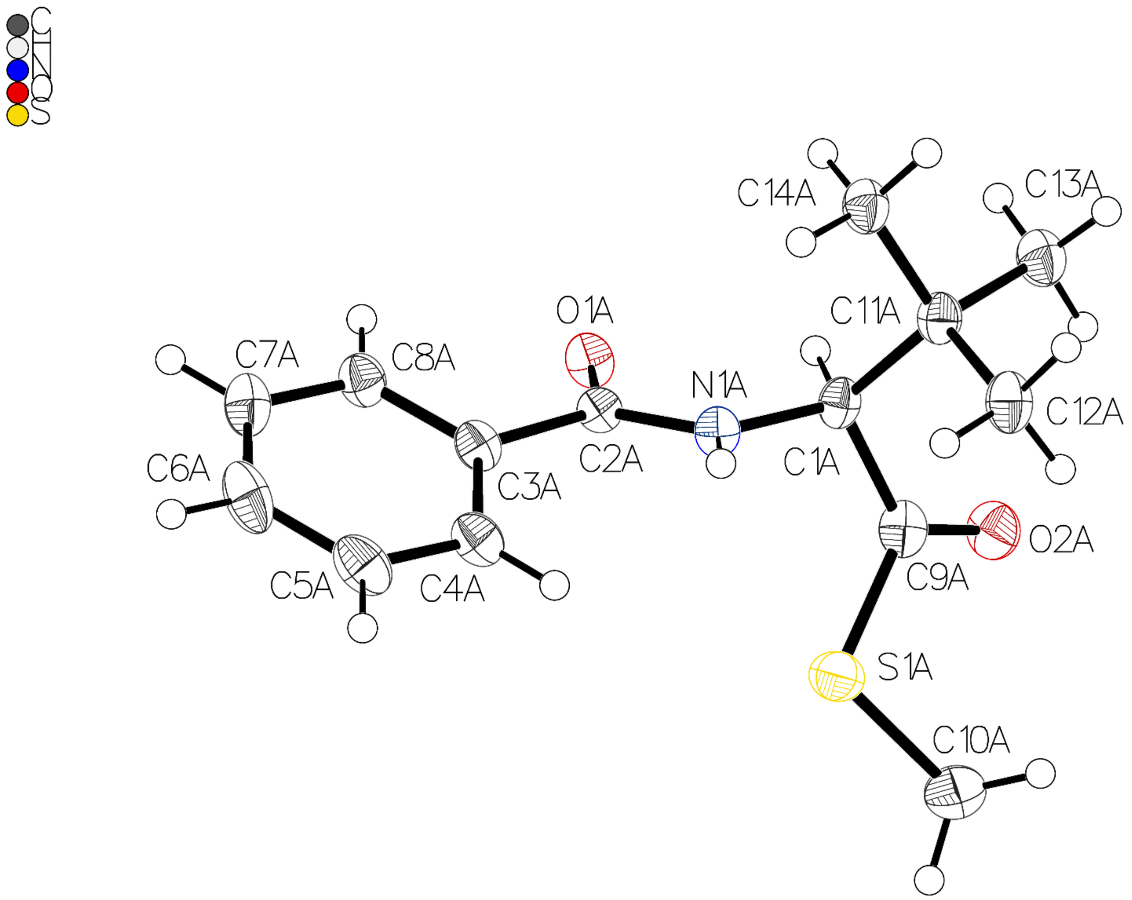

Figure S7 Asymmetric Unit drawn with 50% displacement ellipsoid. The bond precision for C-C single bonds is 0.0057Å. The chirality at C1A is proofed as S by Flack (-0.024 – classical fit, 0.031(43) – Parsons method) and Hooft (0.018(16)) parameter. One additional independent molecule flagged as “B” in the numbering omitted for clarity. The chiral proof for molecules and “A” and “B” are both S. Two Hydrogen bonds (not visualized) of moderate character, N1A – H --- O1B and N1B – H --- O1A were detected in the crystal.

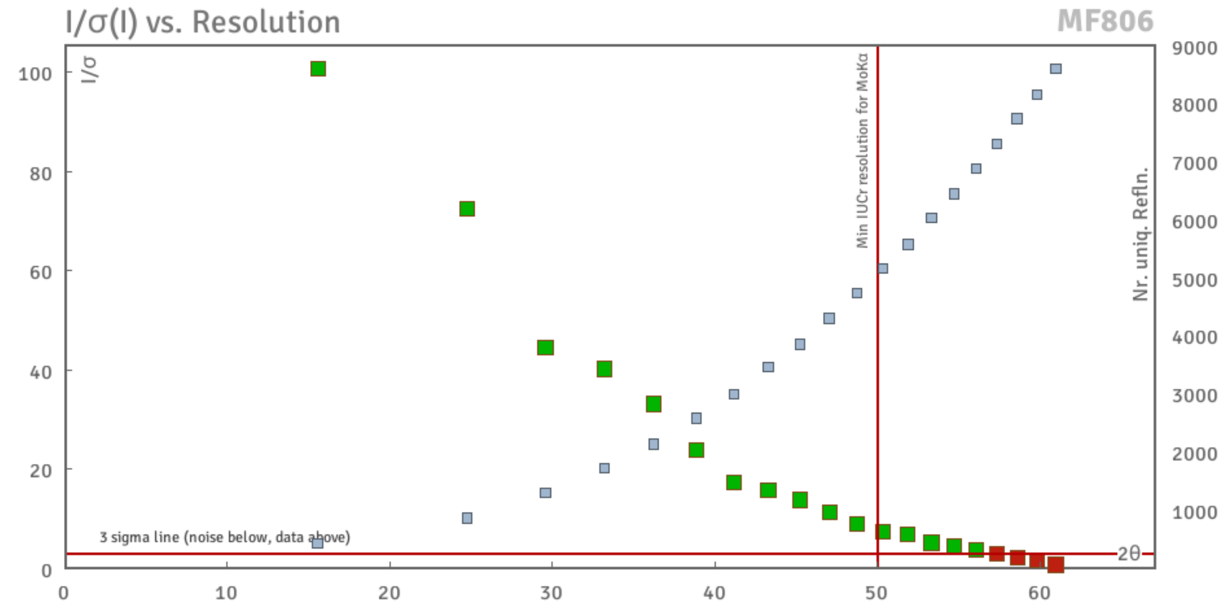

Figure S8 Data Quality – I/Sigma (I) level view: All data are above the three sigma line along the min IUCR definition

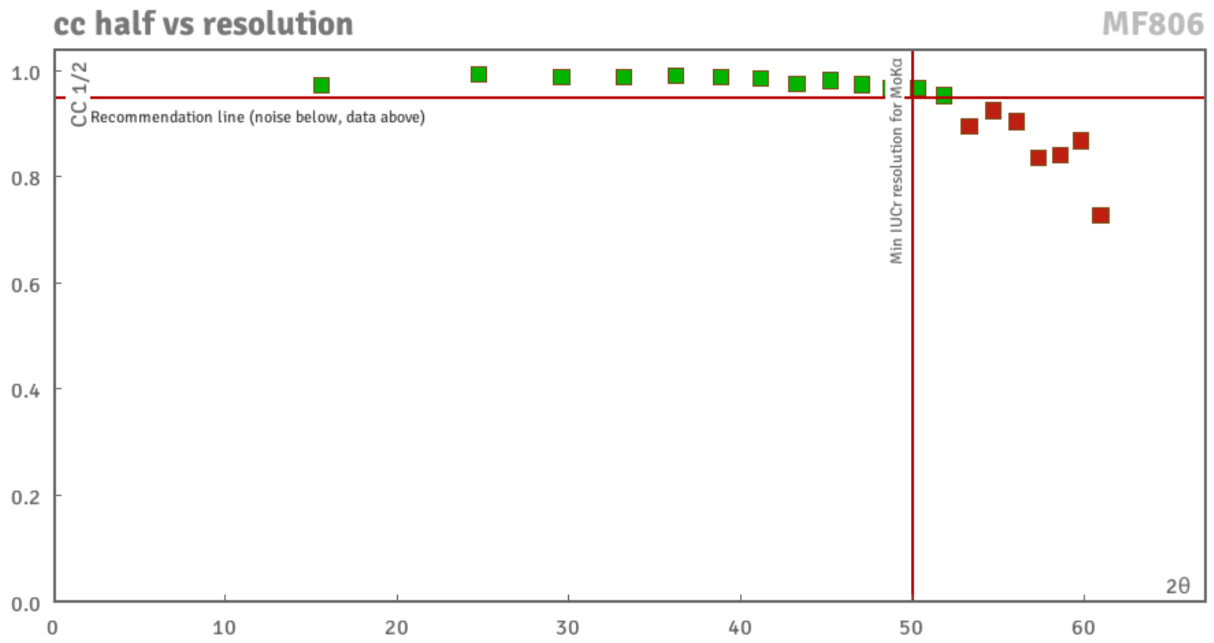

Figure S9 Data Quality – CC 1/2 level view: Data for the cc1/2 plot are located on top of the recommendation line along the min IUCR definition.

Supplementary Table S7 Sample and crystal data, data collection and structure refinement.

Identification code	MF806
Empirical formula	$C_{14}H_{19}NO_2S$
Formula weight	265.36
Temperature/K	100
Crystal system	monoclinic
Space group	$P2_1$
a/Å	9.8950(3)
b/Å	13.6424(4)
c/Å	10.8666(4)
α/°	90
β/°	99.994(3)
γ/°	90
Volume/Å^3	1444.64(8)
Z	4
ρ_{calc}g/cm^3	1.220
μ/mm^{-1}	0.219
F(000)	568.0
Crystal size/mm^3	0.3 × 0.207 × 0.12
Radiation	Mo Kα (λ = 0.71073)
2Θ range for data collection/°	3.806 to 61.536
Index ranges	-12 ≤ h ≤ 13, -19 ≤ k ≤ 17, -15 ≤ l ≤ 13
Reflections collected	32088
Independent reflections	7771 [R_{int} = 0.0384, R_{sigma} = 0.0417]
Data/restraints/parameters	7771/1/334
Goodness-of-fit on F^2	1.178
Final R indexes [I>=2σ (I)]	R_1 = 0.0423, wR_2 = 0.0978
Final R indexes [all data]	R_1 = 0.0736, wR_2 = 0.1332
Largest diff. peak/hole / e Å^{-3}	0.50/-0.46

Flack parameter	0.03(4)

9. NMR Spectra

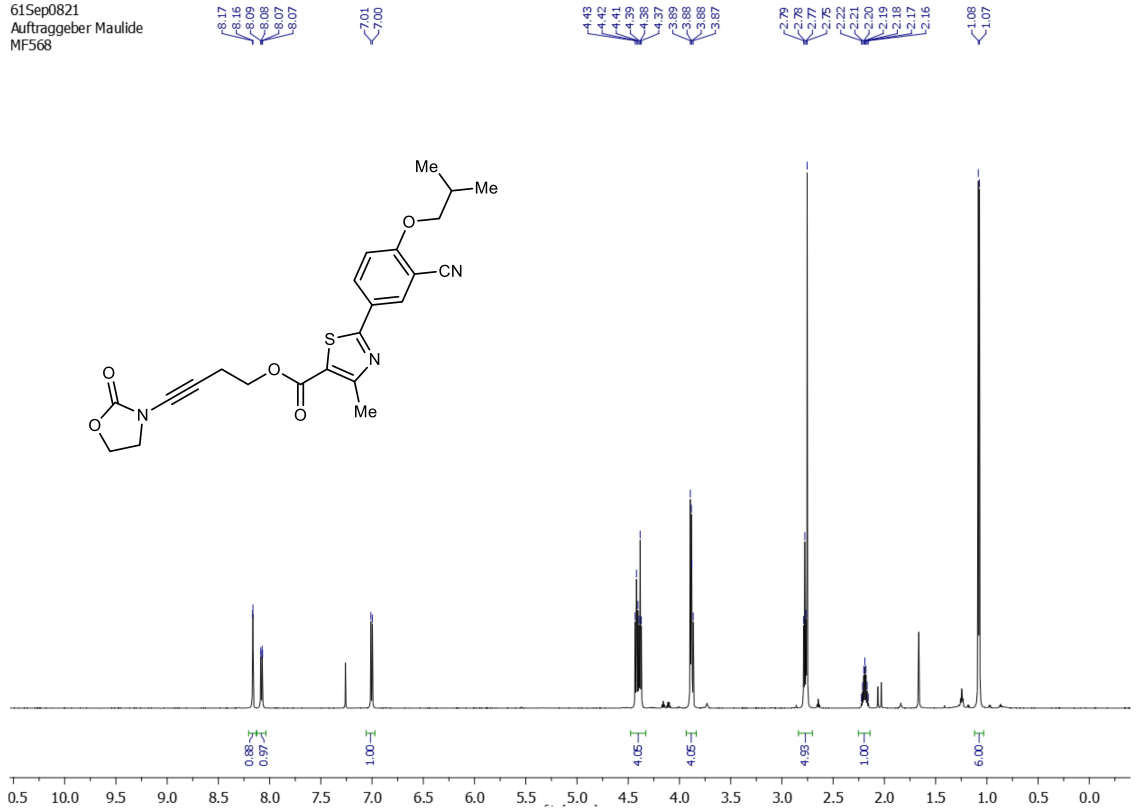

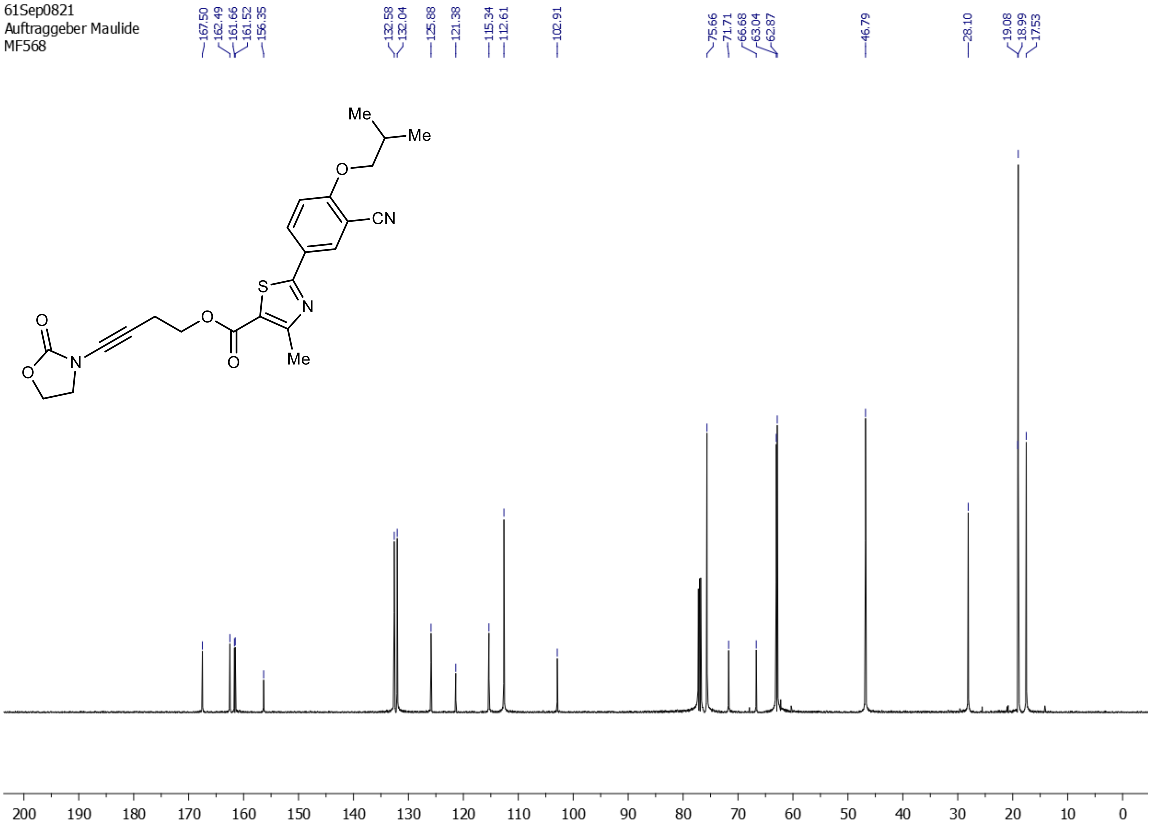

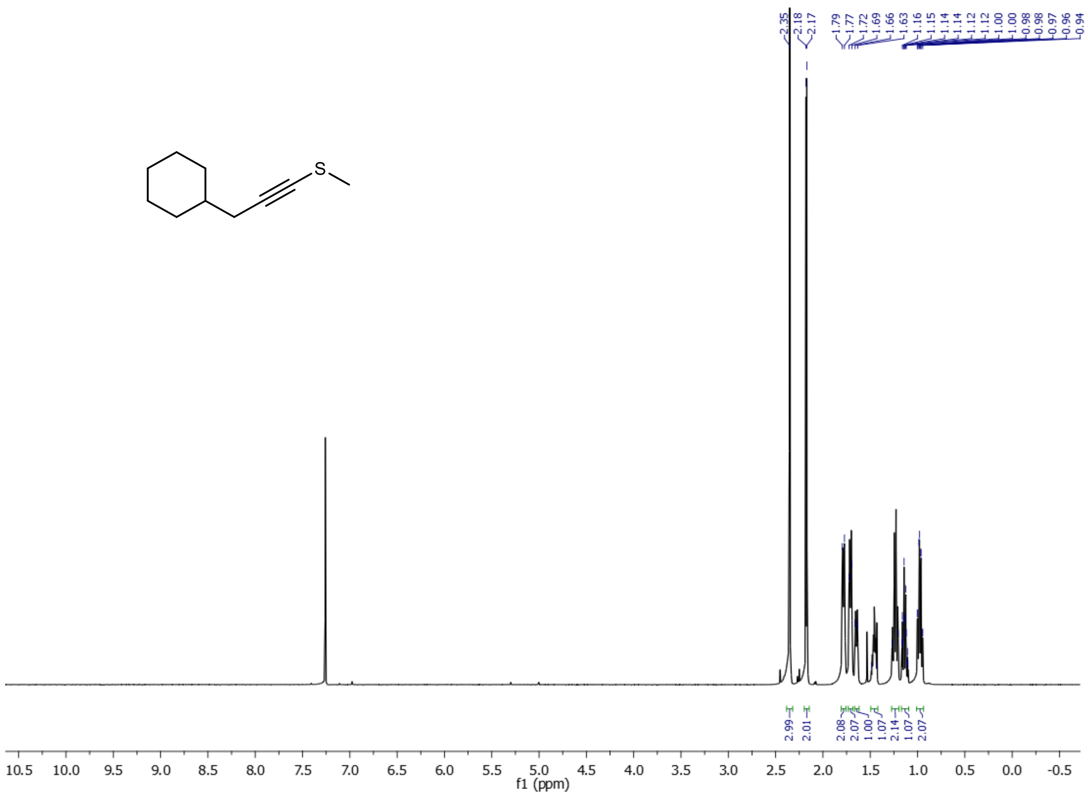
S
2.35
2.18
2.17
1.79
1.77
1.72
1.69
1.66
1.63
1.16
1.15
1.14
1.14
1.12
1.12
1.00
1.00
0.98
0.98
0.97
0.96
0.94
2.99
2.01
2.08
2.07
1.00
1.07
2.14
1.07
2.07
10.5 10.0 9.5 9.0 8.5 8.0 7.5 7.0 6.5 6.0 5.5 5.0 4.5 4.0 3.5 3.0 2.5 2.0 1.5 1.0 0.5 0.0 -0.5
f1 (ppm)

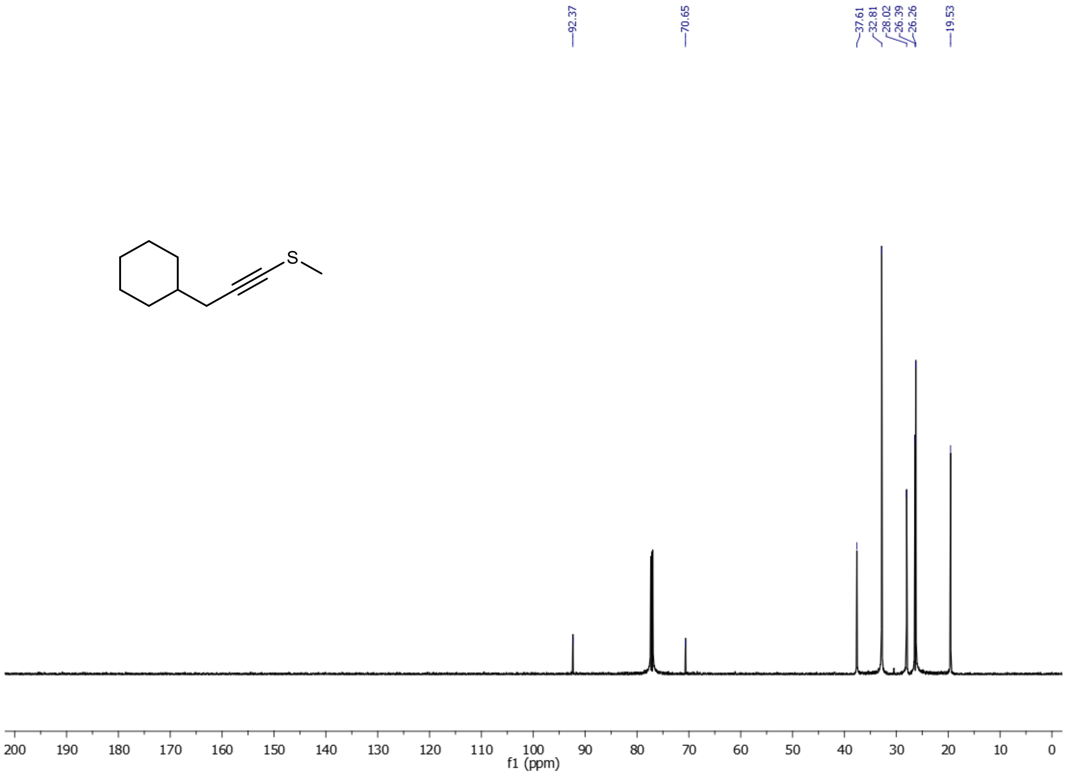
S
92.37
70.65
37.61
32.81
28.02
26.39
26.26
19.53
200 190 180 170 160 150 140 130 120 110 100 90 80 70 60 50 40 30 20 10 0
f1 (ppm)

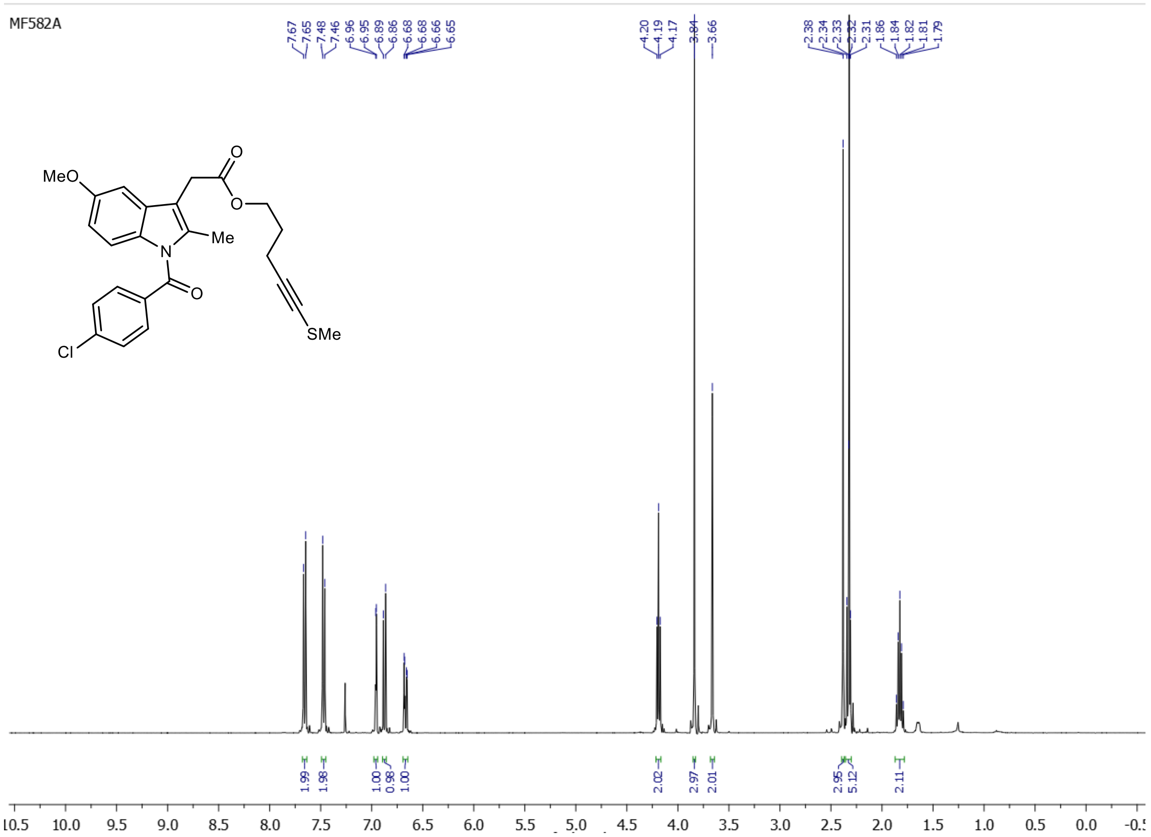
MF582A
7.67
7.65
7.48
7.46
6.96
6.95
6.89
6.86
6.68
6.68
6.66
6.65
4.20
4.19
4.17
3.84
3.66
2.38
2.34
2.33
2.32
2.31
1.86
1.84
1.82
1.81
1.79
MeO
O
O
Me
N
O
Cl
SMe
1.99
1.98
1.00
0.98
1.00
2.02
2.97
2.01
2.95
5.12
2.11

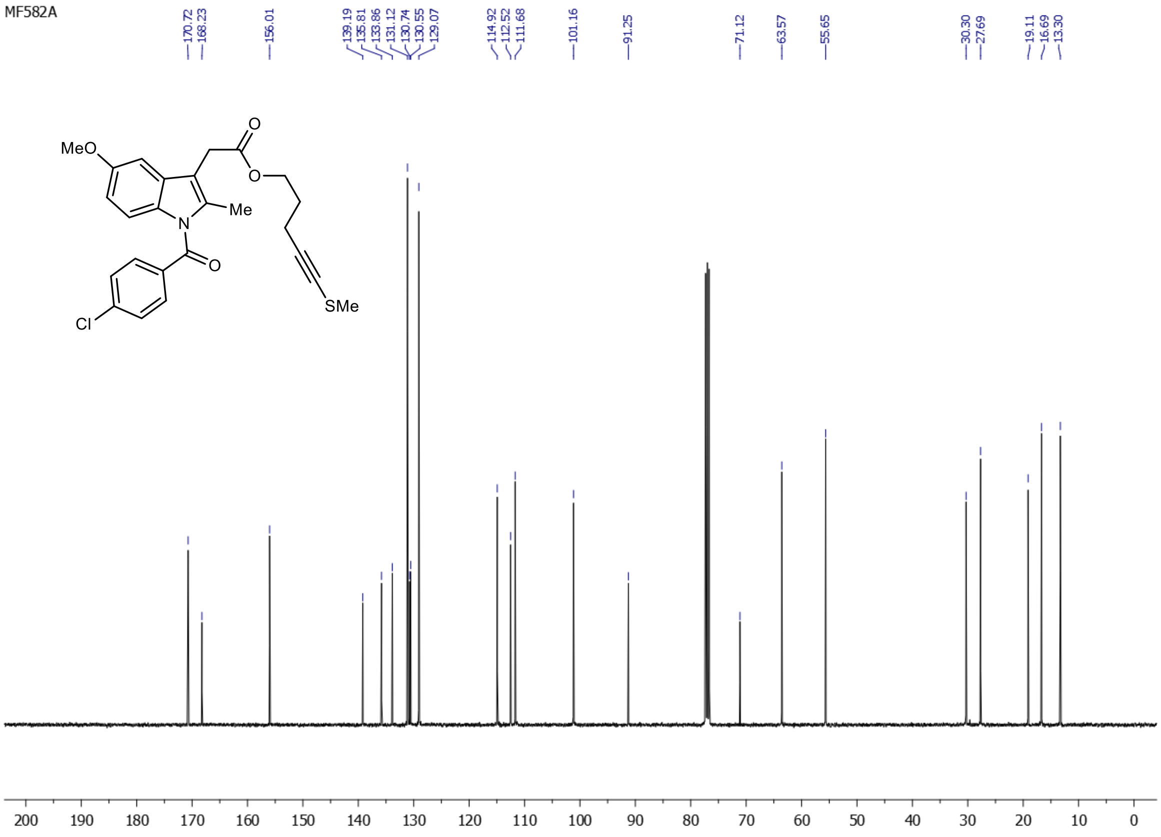
MF582A
170.72
168.23
156.01
139.19
135.81
133.86
131.12
130.74
130.55
129.07
114.92
112.52
111.68
101.16
91.25
71.12
63.57
55.65
30.30
27.69
19.11
16.69
13.30
MeO
O
O
Me
N
O
Cl
SMe

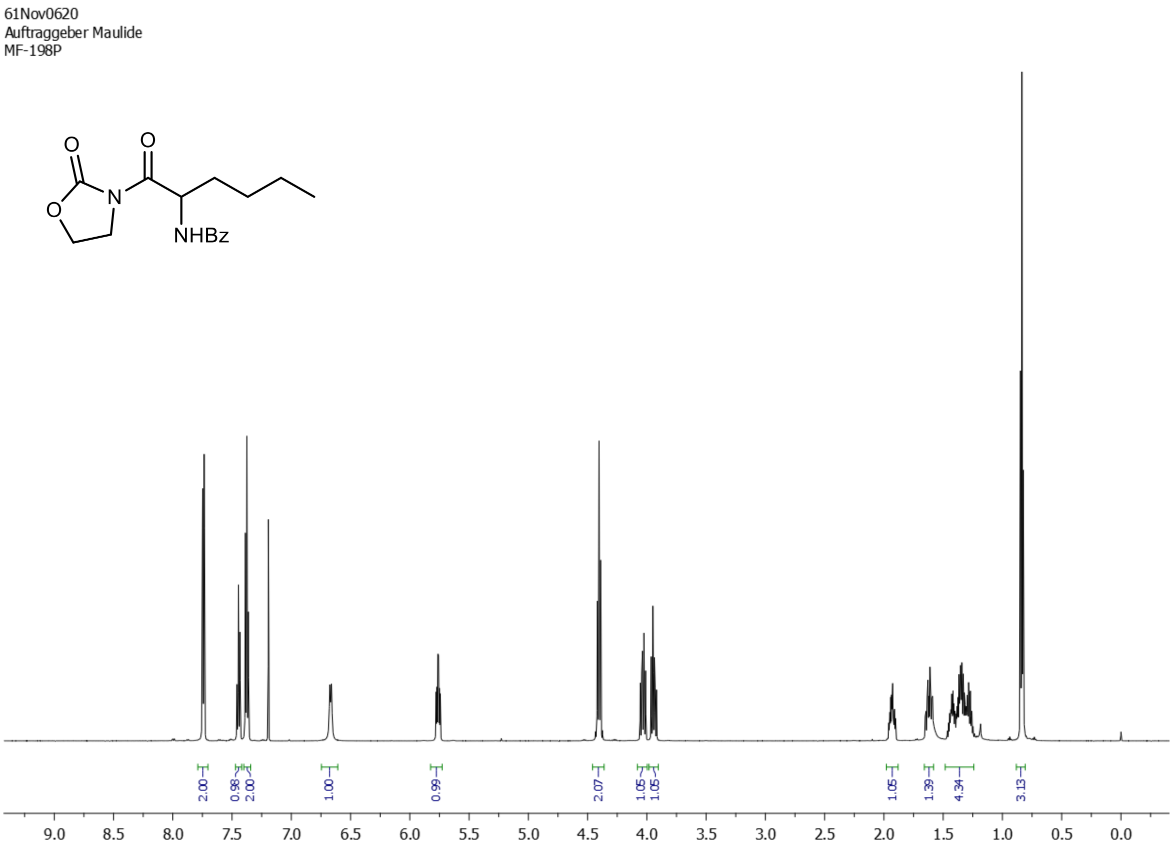
61Nov0620
Auftraggeber Maulide
MF-198P
NHBz
2.00
0.98
2.00
1.00
0.99
2.07
1.05
1.05
1.05
1.39
4.34
3.13
9.0 8.5 8.0 7.5 7.0 6.5 6.0 5.5 5.0 4.5 4.0 3.5 3.0 2.5 2.0 1.5 1.0 0.5 0.0

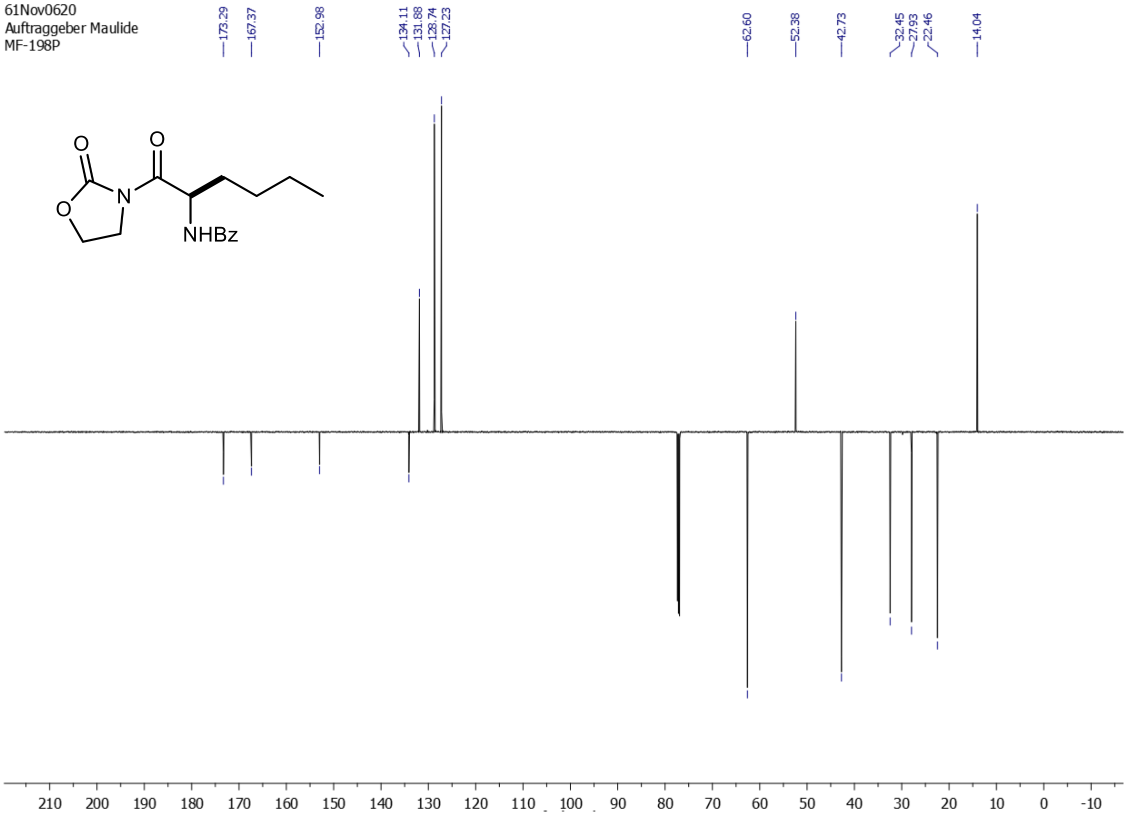
61Nov0620
Auftraggeber Maulide
MF-198P
173.29
167.37
152.98
134.11
131.88
128.74
127.23
62.60
52.38
42.73
32.45
27.93
22.46
14.04
NHBz
210 200 190 180 170 160 150 140 130 120 110 100 90 80 70 60 50 40 30 20 10 0 -10

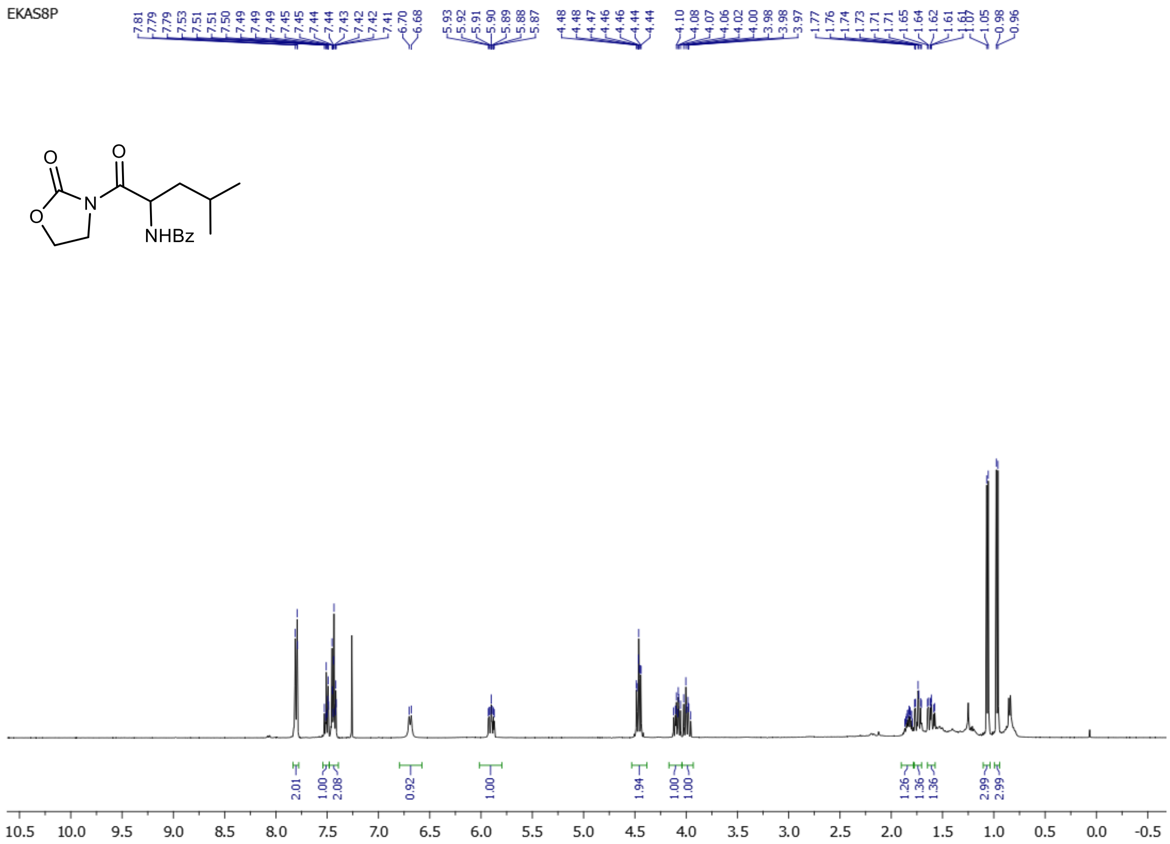
EKAS8P
7.81 7.79 7.53 7.51 7.50 7.49 7.49 7.49 7.45 7.45 7.44 7.44 7.43 7.42 7.42 7.41 6.70 6.68
5.93 5.92 5.91 5.90 5.89 5.88 5.87
4.48 4.48 4.47 4.46 4.46 4.44 4.44
4.10 4.08 4.07 4.06 4.02 4.00 3.98 3.98 3.97
1.77 1.76 1.74 1.73 1.71 1.71 1.65 1.64 1.62 1.61 1.61 1.07 1.05 0.98 0.96
NHBz
2.01 1.00 2.08 0.92 1.00 1.94 1.00 1.00 1.26 1.36 1.36 2.99 2.99

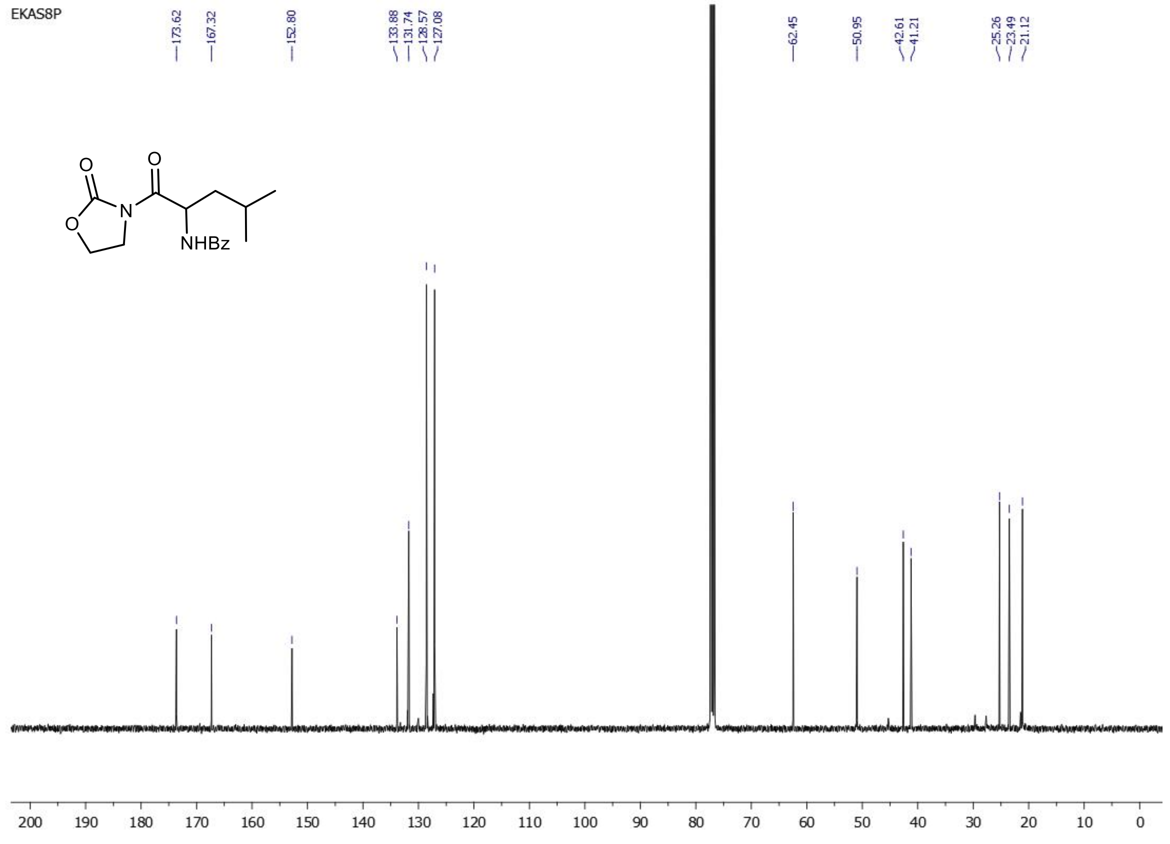
EKAS8P
173.62 167.32 152.80 133.88 131.74 128.57 127.08 62.45 50.95 42.61 41.21 25.26 23.49 21.12
NHBz

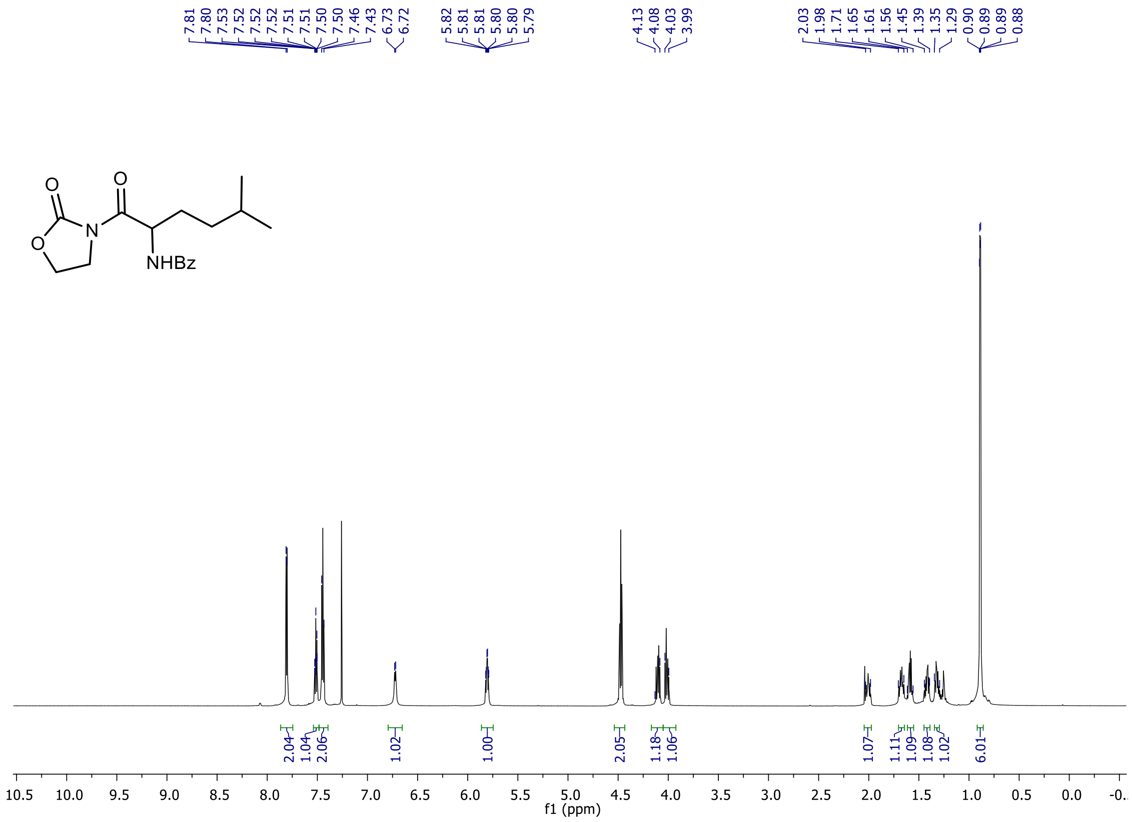
7.81
7.80
7.53
7.52
7.52
7.52
7.51
7.51
7.50
7.50
7.46
7.43
6.73
6.72
5.82
5.81
5.81
5.80
5.80
5.79
4.13
4.08
4.03
3.99
2.03
1.98
1.71
1.65
1.61
1.56
1.45
1.39
1.35
1.29
0.90
0.89
0.89
0.88
O
O
N
O
O
NHBz
2.04
1.04
2.06
1.02
1.00
2.05
1.18
1.06
1.07
1.11
1.09
1.08
1.02
6.01
f1 (ppm)

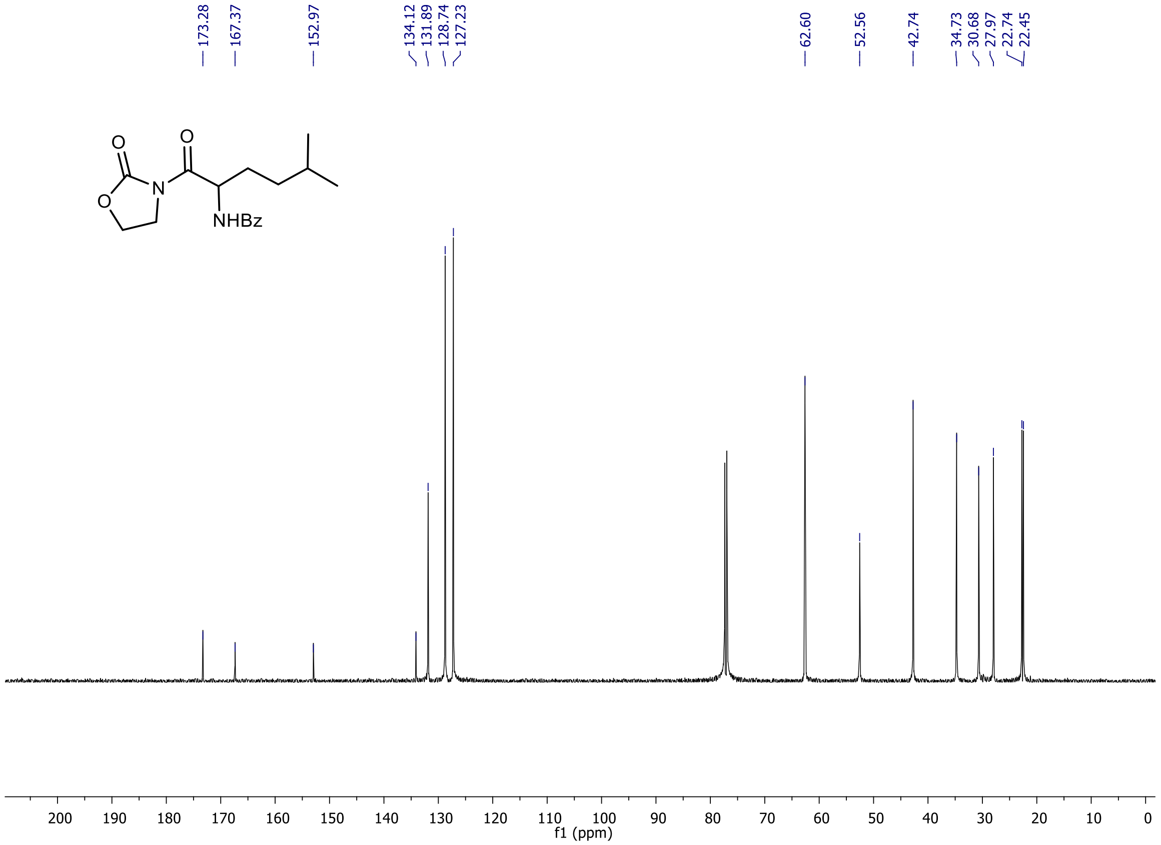
173.28
167.37
152.97
134.12
131.89
128.74
127.23
62.60
52.56
42.74
34.73
30.68
27.97
22.74
22.45
O
O
N
O
O
NHBz
f1 (ppm)

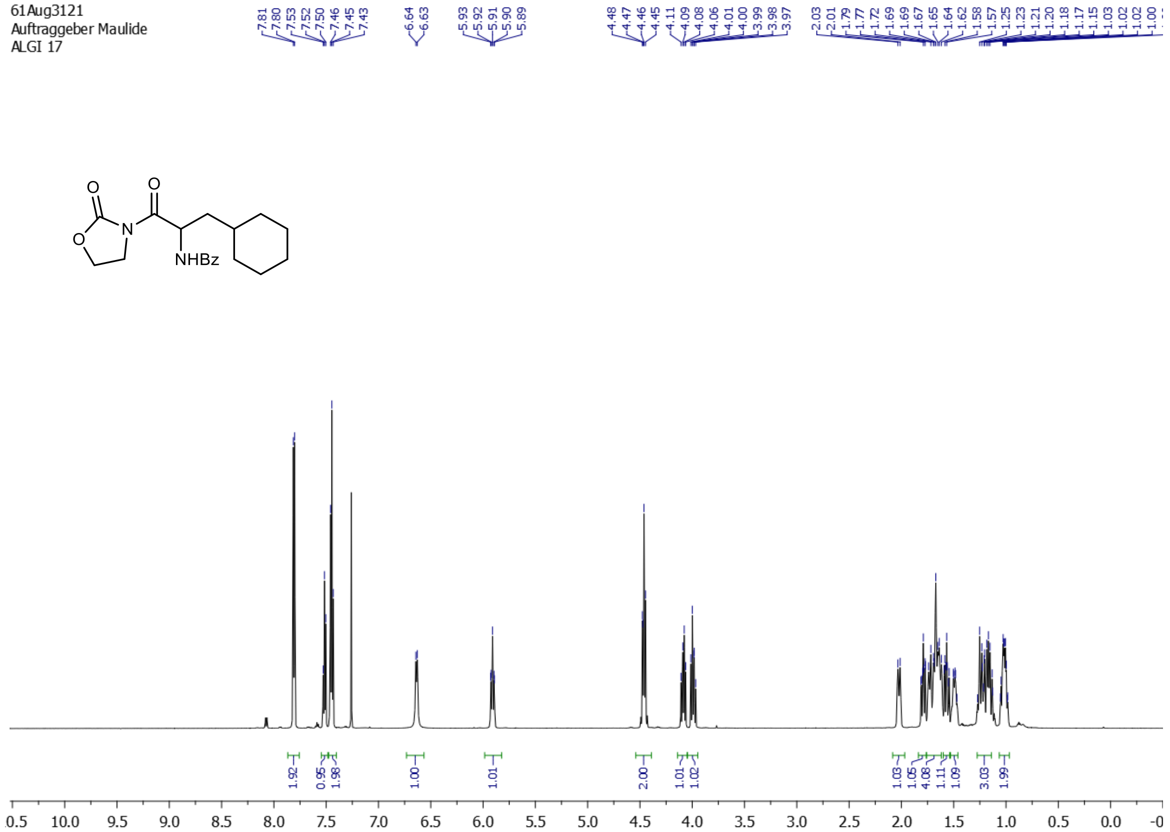
61Aug3121
Auftraggeber Maulide
ALGI 17
7.81 7.80 7.53 7.52 7.50 7.46 7.45 7.43 6.64 6.63 5.93 5.92 5.91 5.90 5.89 4.48 4.47 4.46 4.45 4.11 4.09 4.08 4.06 4.01 4.00 3.99 3.98 3.97 2.03 2.01 1.79 1.77 1.72 1.69 1.67 1.65 1.64 1.62 1.58 1.57 1.25 1.23 1.21 1.20 1.18 1.17 1.15 1.03 1.02 1.02 1.00
O
N
O
O
NHBz
1.92 0.95 1.98 1.00 1.01 2.00 1.01 1.02 1.03 1.05 4.08 1.11 1.09 3.03 1.99
0.5 10.0 9.5 9.0 8.5 8.0 7.5 7.0 6.5 6.0 5.5 5.0 4.5 4.0 3.5 3.0 2.5 2.0 1.5 1.0 0.5 0.0 -0

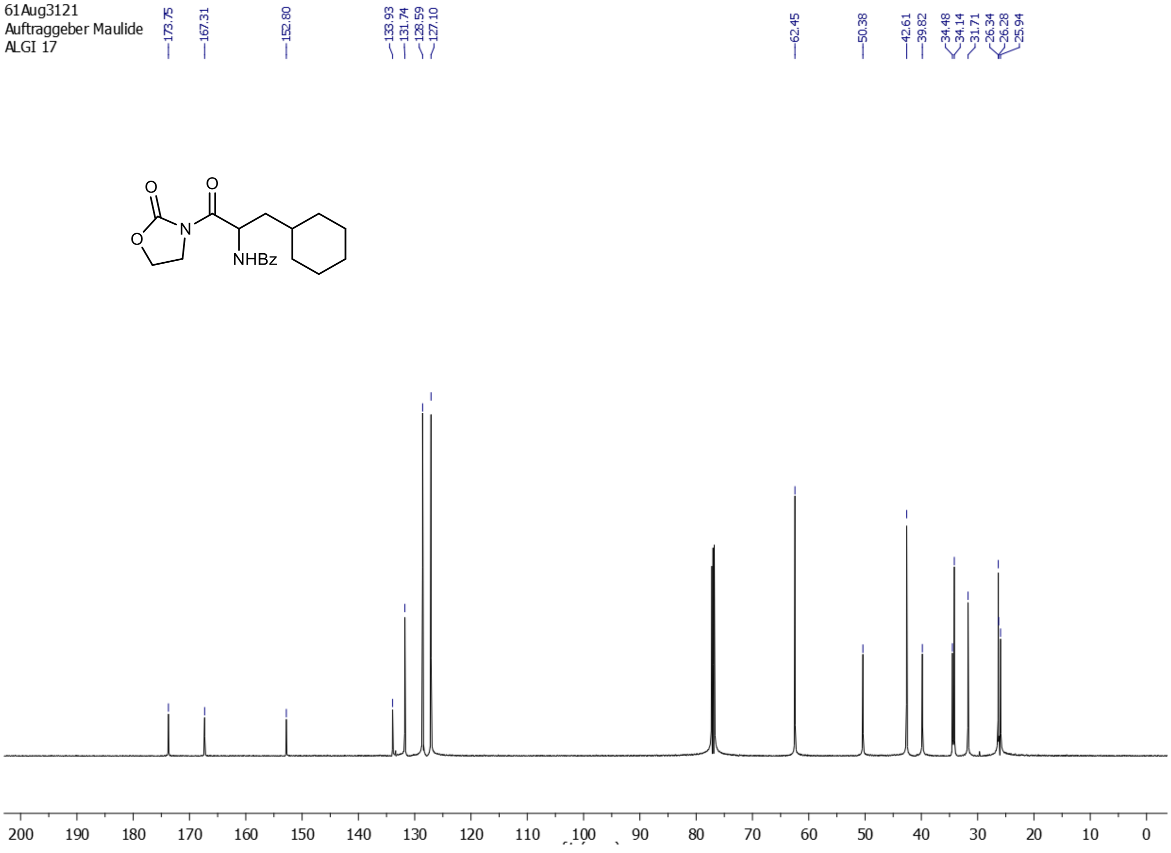
61Aug3121
Auftraggeber Maulide
ALGI 17
173.75 167.31 152.80 133.93 131.74 128.59 127.10 62.45 50.38 42.61 39.82 34.48 34.14 31.71 26.34 26.28 25.94
O
N
O
O
NHBz
200 190 180 170 160 150 140 130 120 110 100 90 80 70 60 50 40 30 20 10 0

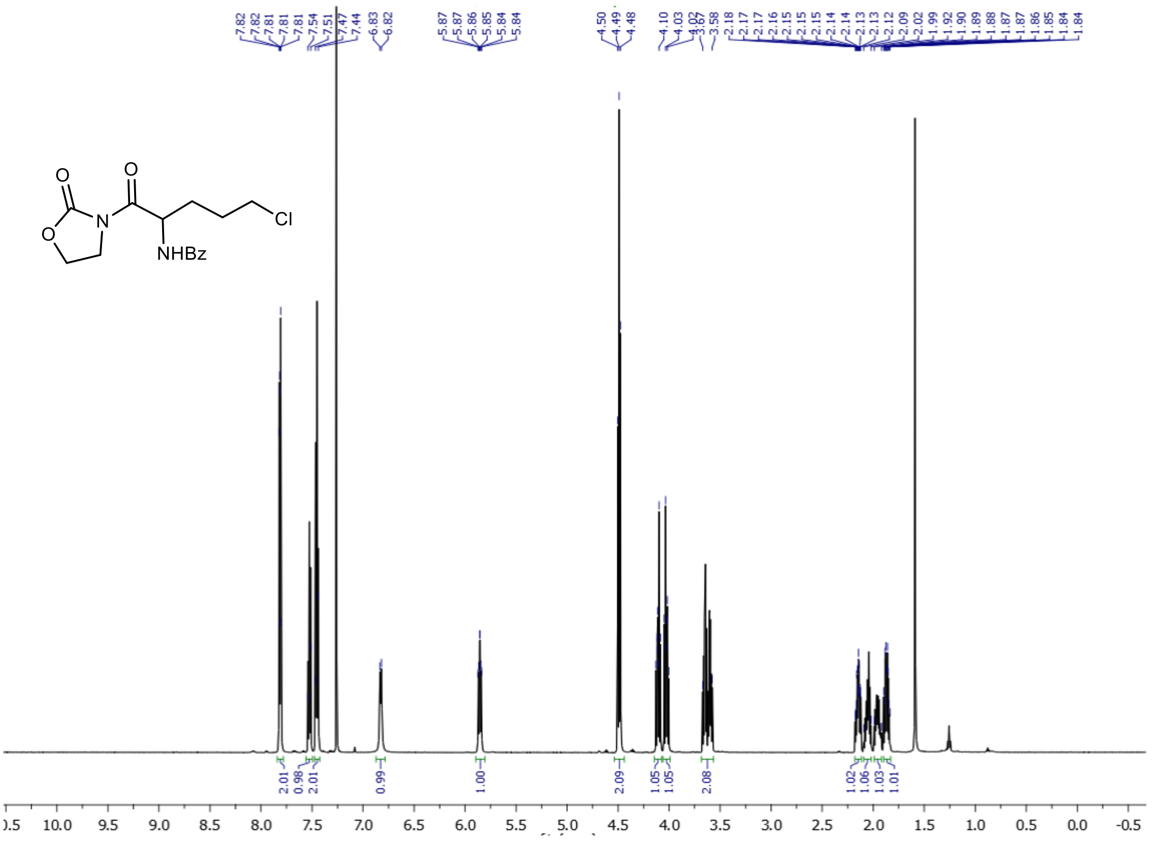

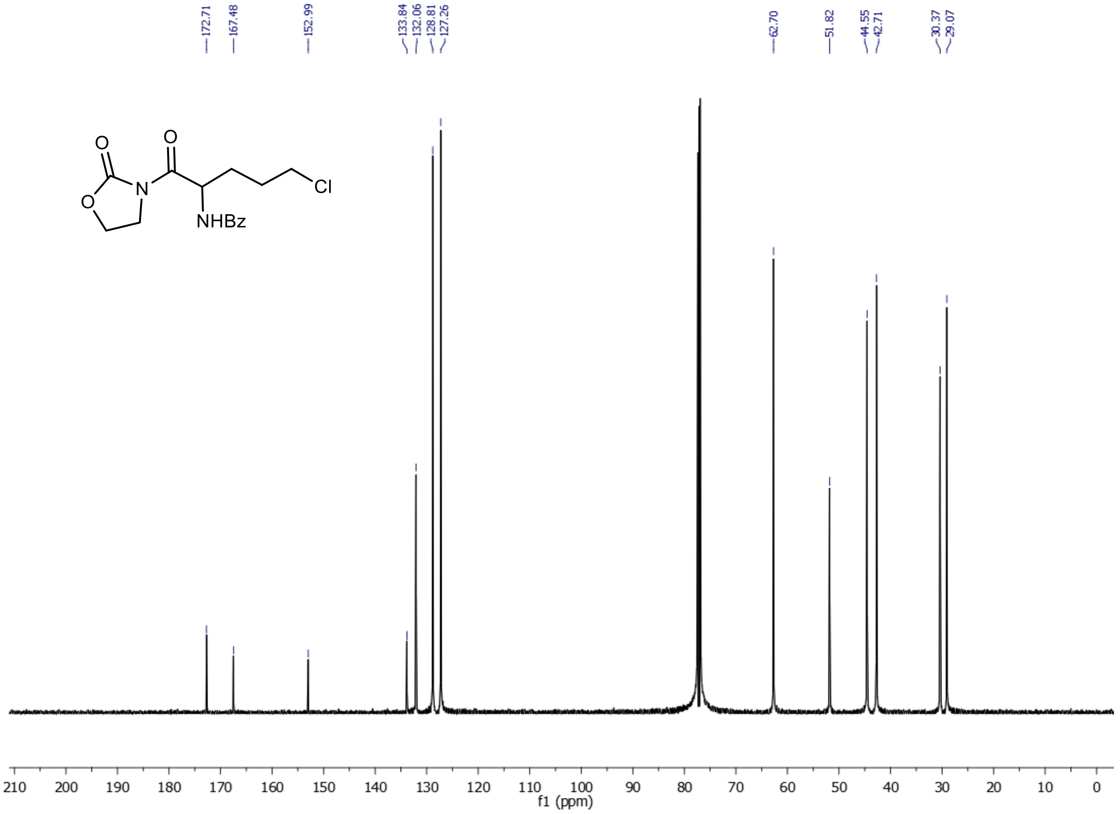

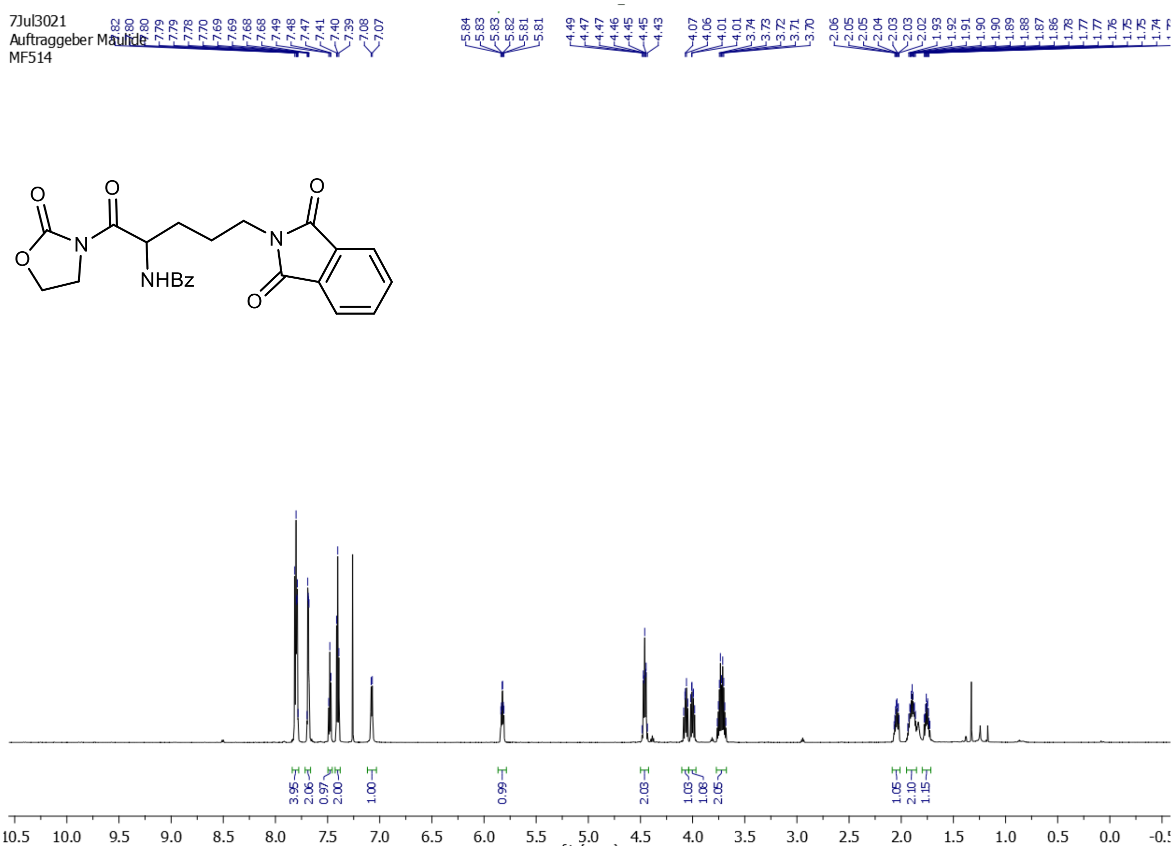

7Jul3021
Auftraggeber Maulide
MF514
NHBz

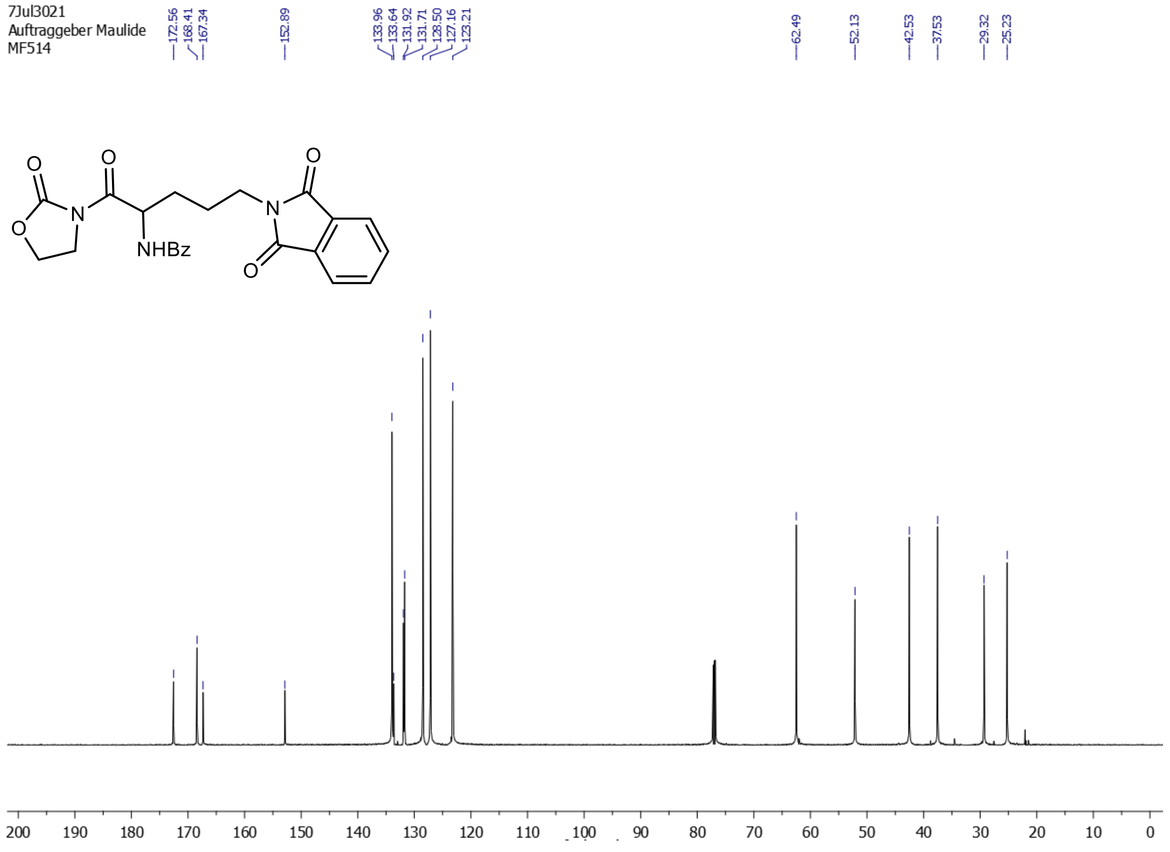

7Jul3021
Auftraggeber Maulide
MF514
172.56
168.41
167.34
152.89
133.96
133.64
131.92
131.71
128.50
127.16
123.21
62.49
52.13
42.53
37.53
29.32
25.23
NHBz

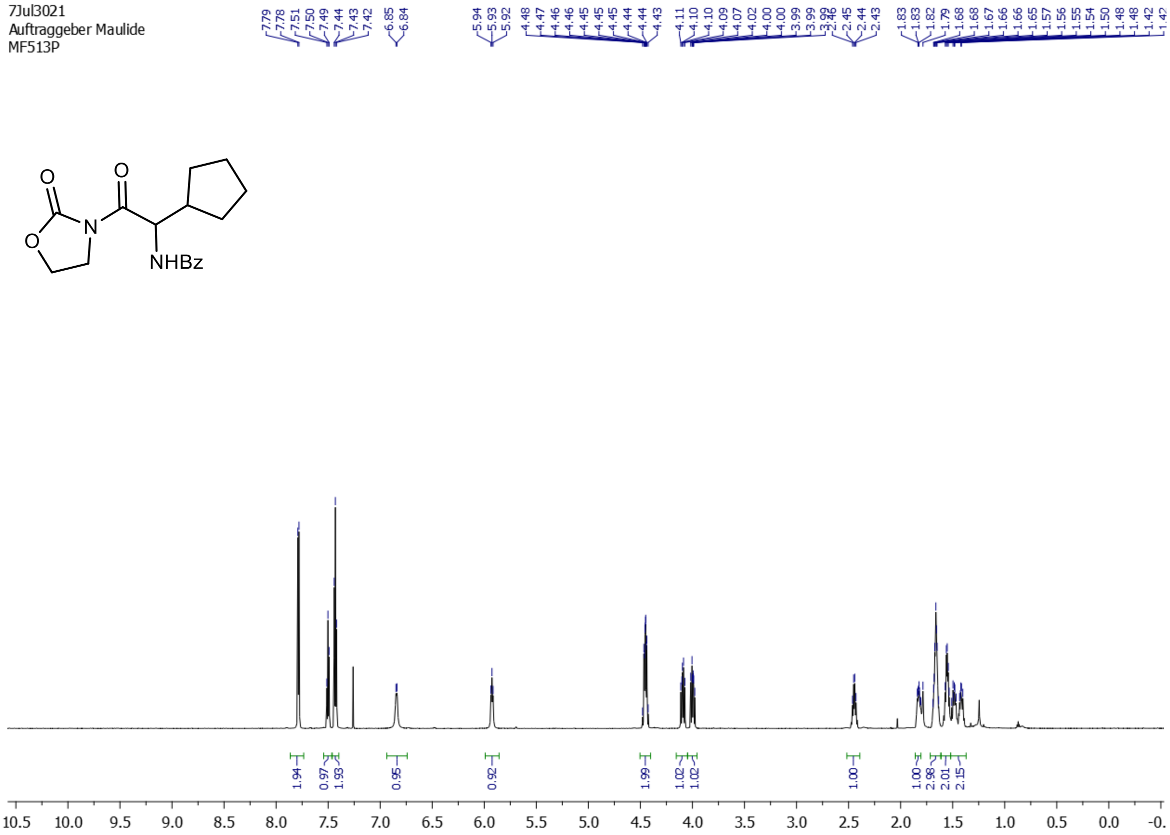
7Jul3021
Auftraggeber Maulide
MF513P
7.79
7.78
7.51
7.50
7.49
7.44
7.43
7.42
6.85
6.84
5.94
5.93
5.92
4.48
4.47
4.46
4.45
4.45
4.45
4.44
4.44
4.44
4.43
4.11
4.10
4.10
4.09
4.07
4.02
4.00
4.00
3.99
3.99
2.45
2.44
2.43
1.83
1.83
1.82
1.79
1.68
1.68
1.67
1.66
1.65
1.57
1.56
1.55
1.54
1.50
1.48
1.42
NHBz
O
N
1.94
0.97
1.93
0.95
0.92
1.99
1.02
1.02
1.00
1.00
2.98
2.01
2.15
10.5
10.0
9.5
9.0
8.5
8.0
7.5
7.0
6.5
6.0
5.5
5.0
4.5
4.0
3.5
3.0
2.5
2.0
1.5
1.0
0.5
0.0

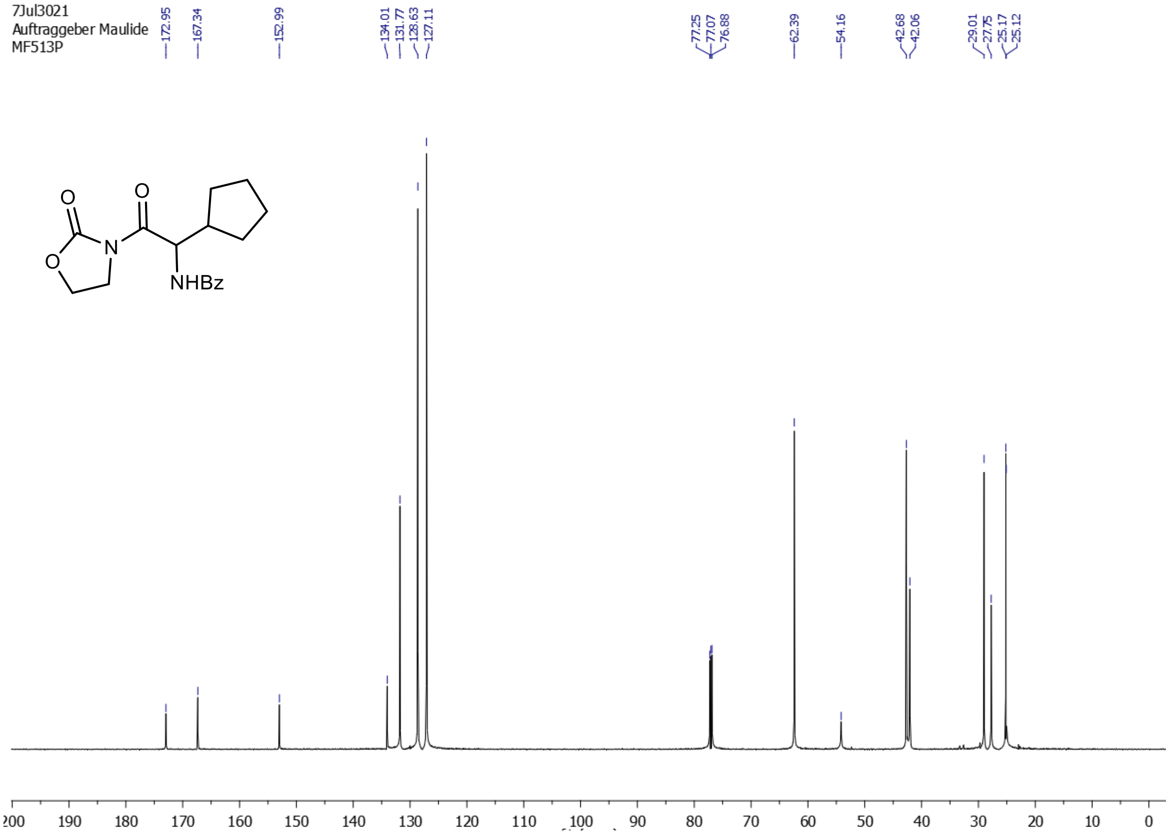
7Jul3021
Auftraggeber Maulide
MF513P
172.95
167.34
152.99
134.01
131.77
128.63
127.11
77.25
77.07
76.88
62.39
54.16
42.68
42.06
29.01
27.75
25.17
25.12
NHBz
O
N
190
180
170
160
150
140
130
120
110
100
90
80
70
60
50
40
30
20
10
0

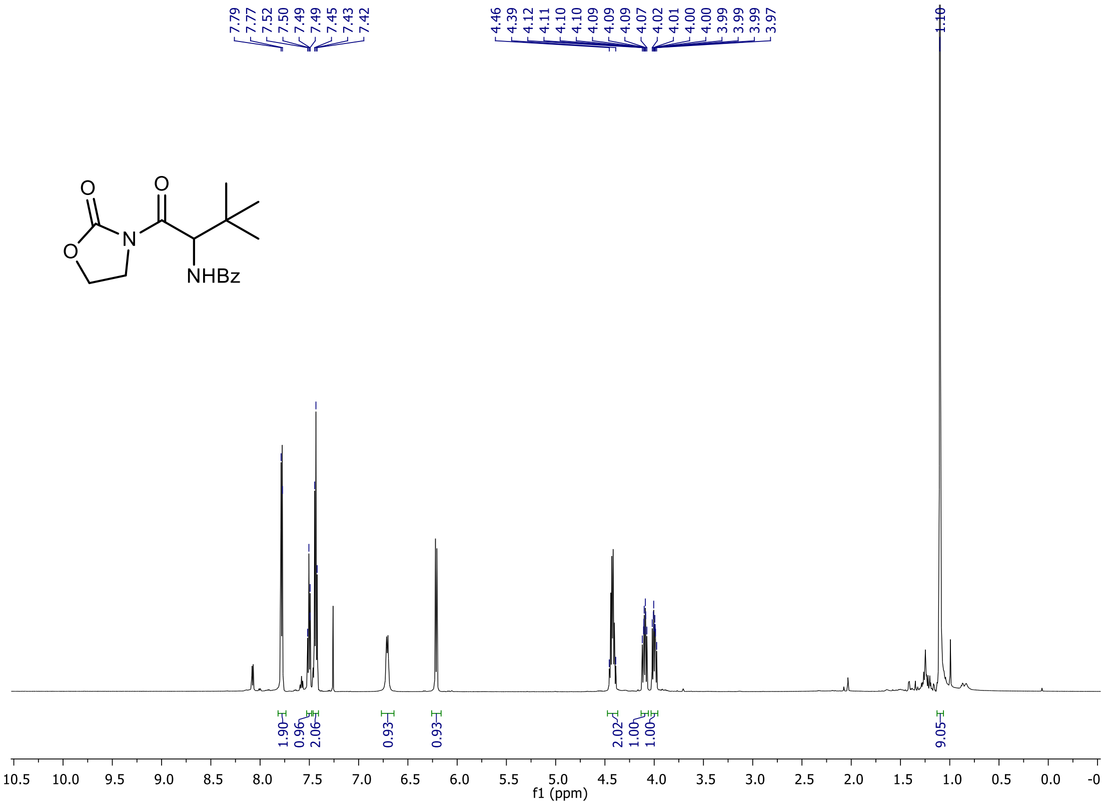

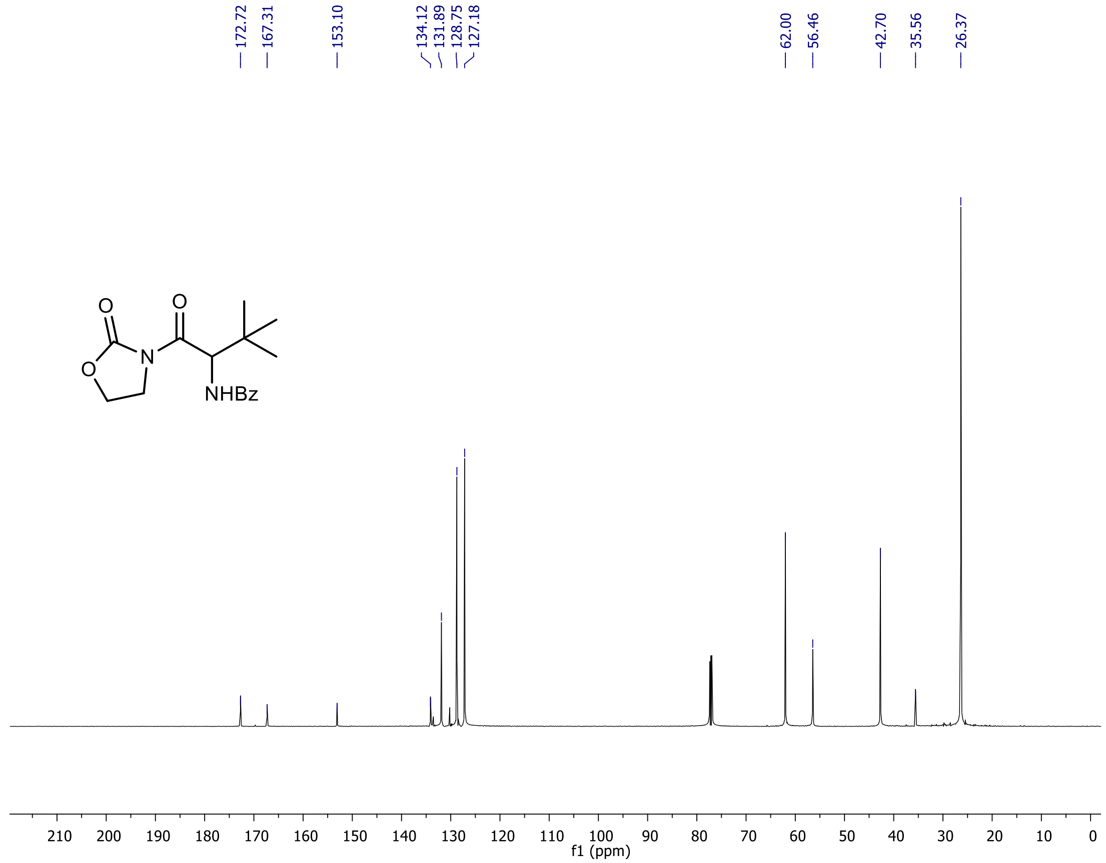

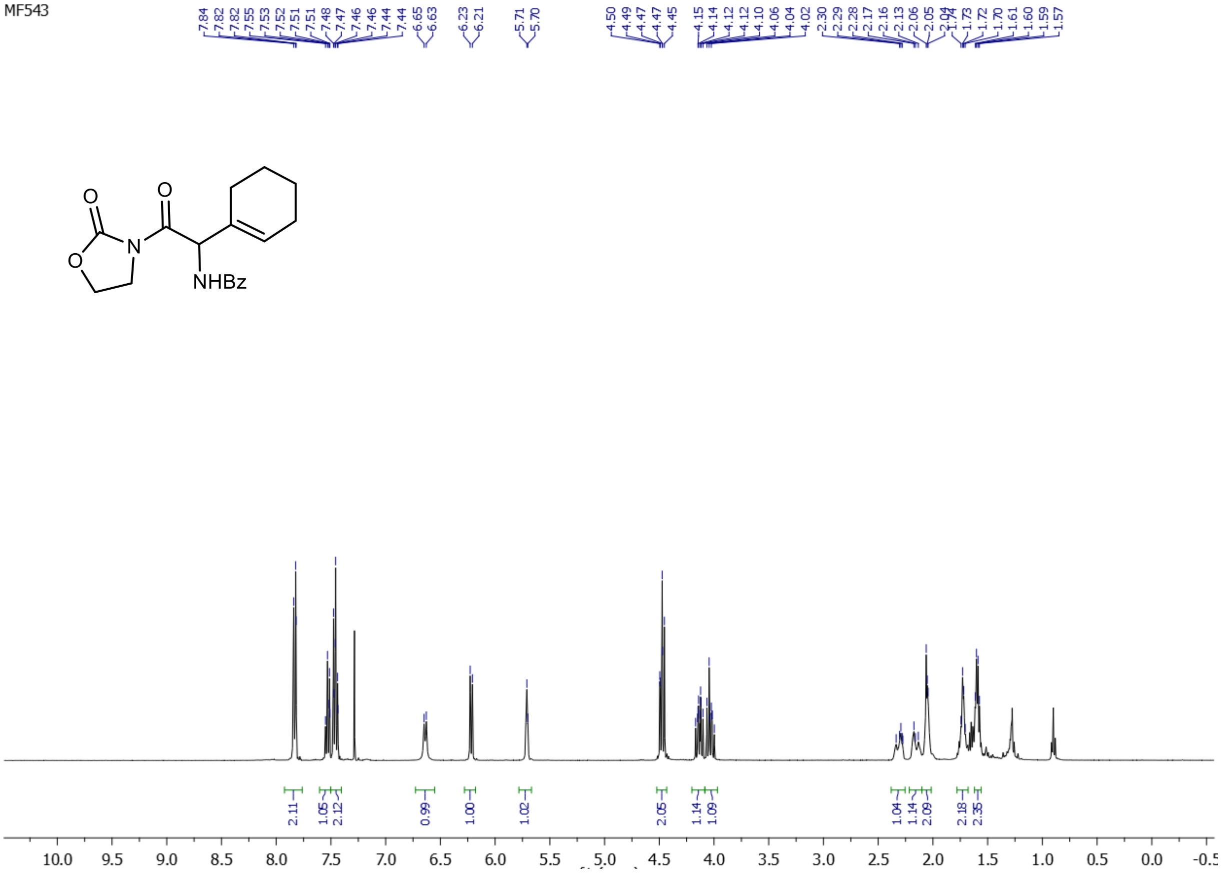
MF543
7.84 7.82 7.82 7.55 7.53 7.52 7.51 7.51 7.48 7.47 7.46 7.46 7.44 6.65 6.63 6.23 6.21 5.71 5.70 4.50 4.49 4.47 4.47 4.45 4.15 4.14 4.12 4.12 4.10 4.06 4.04 4.02 2.30 2.29 2.28 2.17 2.16 2.13 2.06 2.05 1.74 1.73 1.72 1.70 1.61 1.60 1.59 1.57
NHBz
2.11 1.05 2.12 0.99 1.00 1.02 2.05 1.14 1.09 1.04 1.14 2.09 2.18 2.35
10.0 9.5 9.0 8.5 8.0 7.5 7.0 6.5 6.0 5.5 5.0 4.5 4.0 3.5 3.0 2.5 2.0 1.5 1.0 0.5 0.0
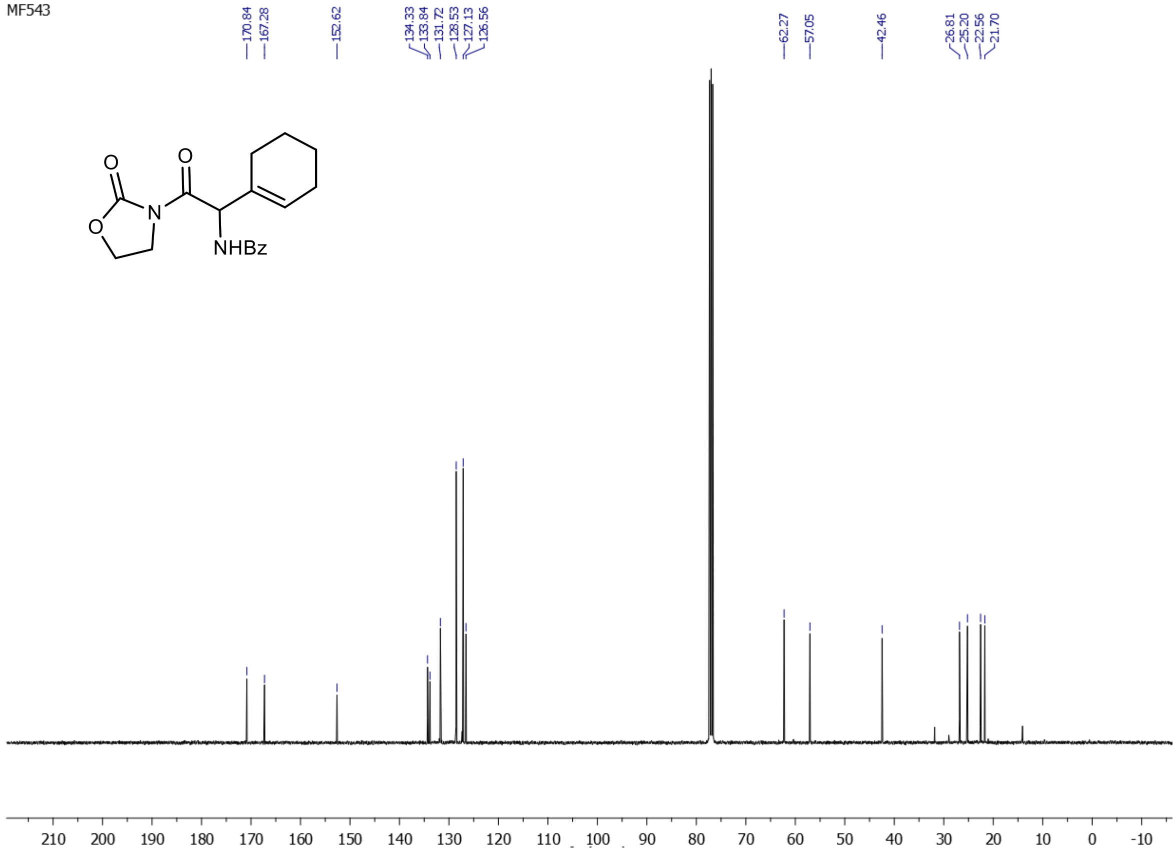
MF543
170.84 167.28 152.62 134.33 133.84 131.72 128.53 127.13 126.56 62.27 57.05 42.46 26.81 25.20 22.56 21.70
NHBz
210 200 190 180 170 160 150 140 130 120 110 100 90 80 70 60 50 40 30 20 10 0 -10

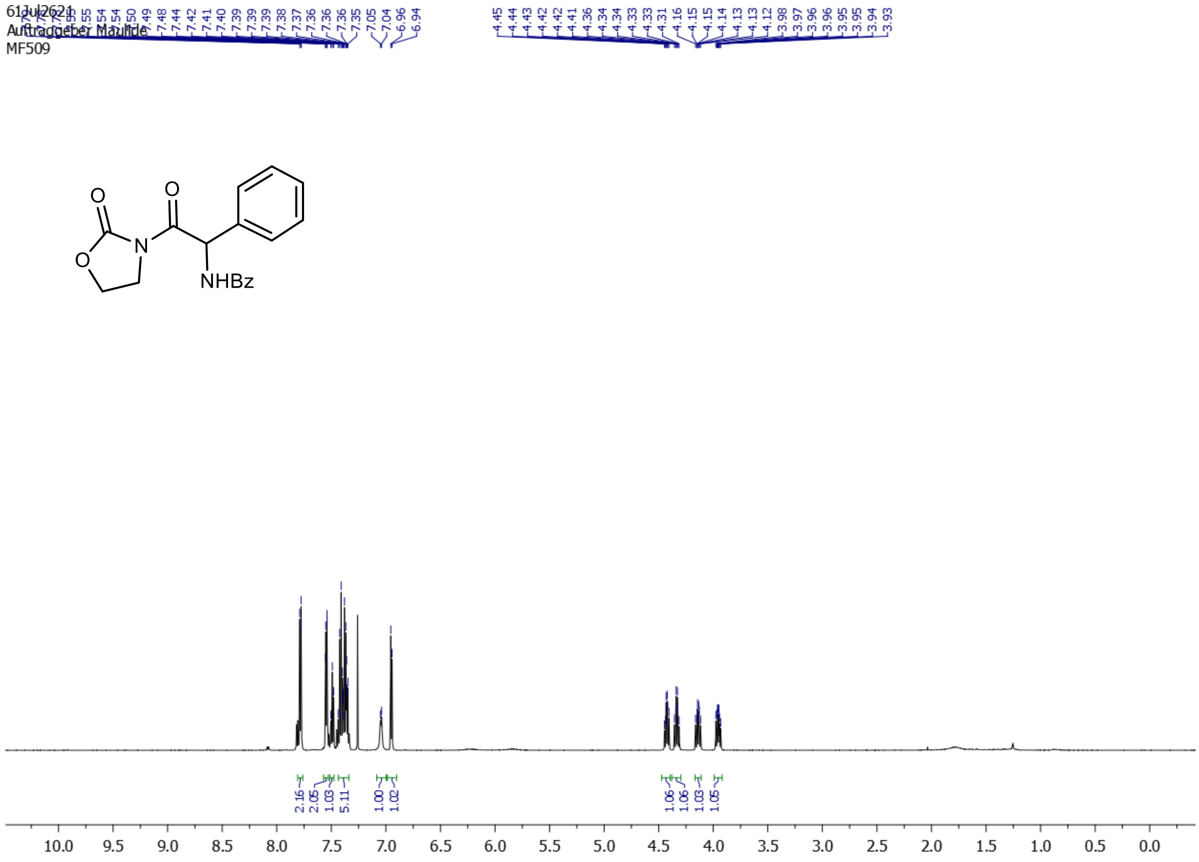
61Jul2621
Auftraggeber Maulide
MF509
7.55 7.54 7.50 7.49 7.48 7.44 7.42 7.41 7.40 7.39 7.39 7.39 7.38 7.37 7.36 7.36 7.36 7.35 7.05 7.04 6.96 6.94
4.45 4.44 4.43 4.42 4.42 4.41 4.36 4.34 4.34 4.33 4.33 4.31 4.16 4.15 4.15 4.14 4.13 4.13 4.12 3.98 3.97 3.96 3.96 3.95 3.95 3.94 3.93
O
O
N
O
NHBz
2.16 2.05 1.03 5.11 1.00 1.02 1.06 1.06 1.03 1.05
10.0 9.5 9.0 8.5 8.0 7.5 7.0 6.5 6.0 5.5 5.0 4.5 4.0 3.5 3.0 2.5 2.0 1.5 1.0 0.5 0.0

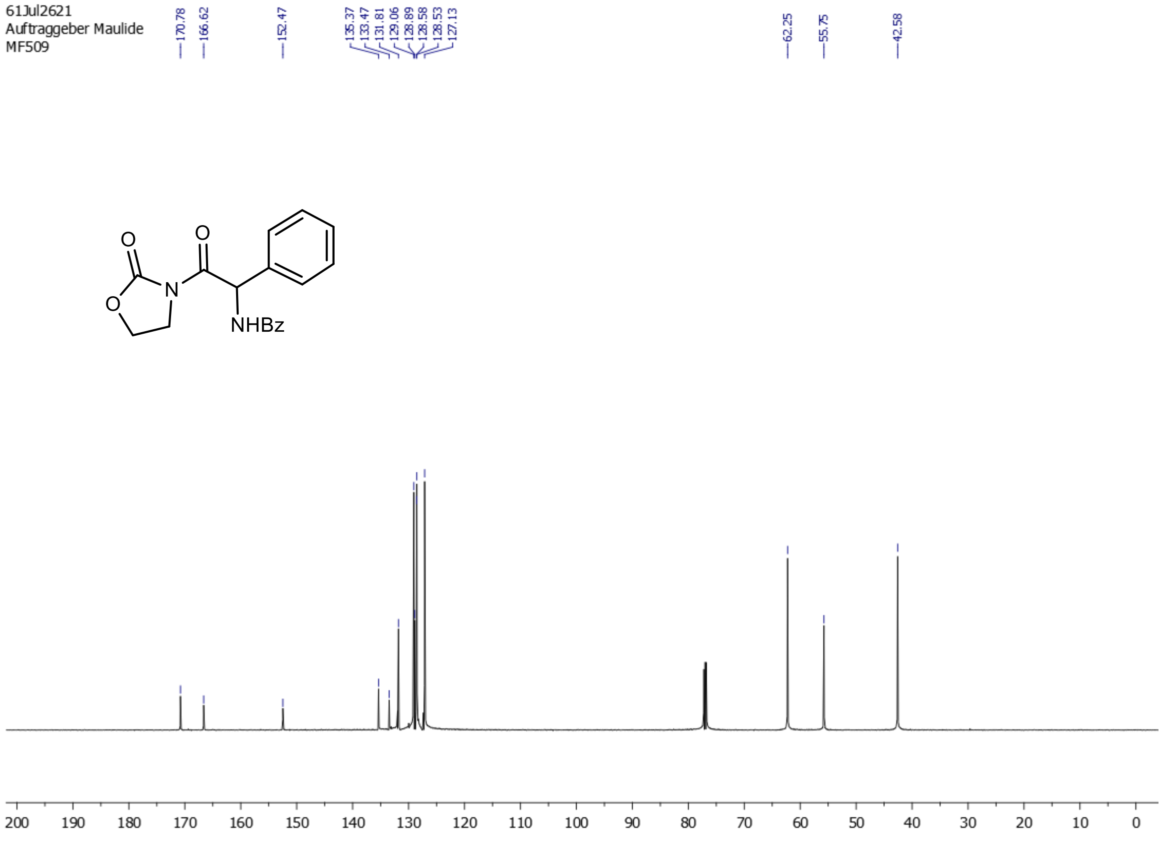
61Jul2621
Auftraggeber Maulide
MF509
170.78 166.62 152.47 135.37 133.47 131.81 129.06 128.89 128.58 128.53 127.13 62.25 55.75 42.58
O
O
N
O
NHBz
200 190 180 170 160 150 140 130 120 110 100 90 80 70 60 50 40 30 20 10 0

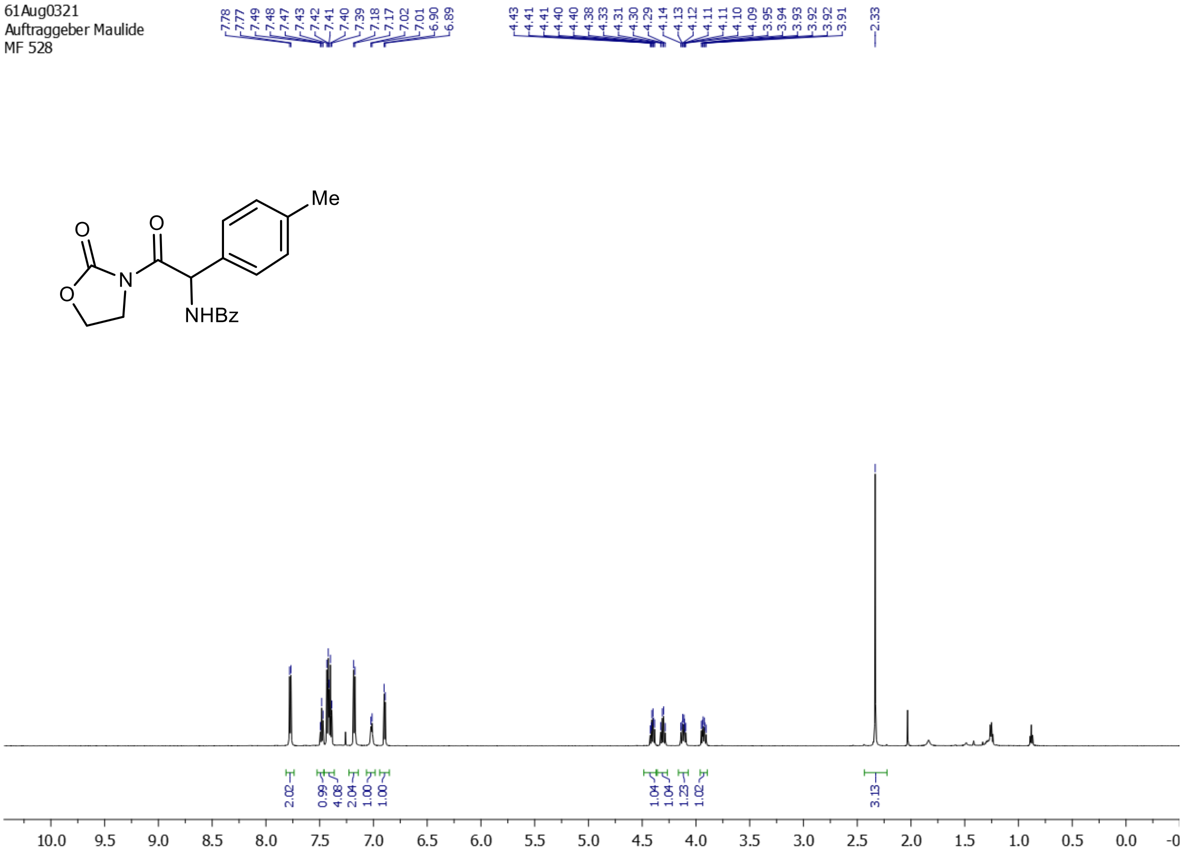
61Aug0321
Auftraggeber Maulide
MF 528
7.78 7.77 7.49 7.48 7.47 7.43 7.42 7.41 7.40 7.39 7.18 7.17 7.02 7.01 6.90 6.89
4.43 4.41 4.41 4.40 4.40 4.38 4.33 4.31 4.30 4.29 4.14 4.13 4.12 4.11 4.11 4.10 4.09 3.95 3.94 3.93 3.92 3.92 3.91
2.33
Me
O
O
N
O
NHBz
2.02 0.99 4.08 2.04 1.00 1.00
1.04 1.04 1.23 1.02
3.13
10.0 9.5 9.0 8.5 8.0 7.5 7.0 6.5 6.0 5.5 5.0 4.5 4.0 3.5 3.0 2.5 2.0 1.5 1.0 0.5 0.0 -0

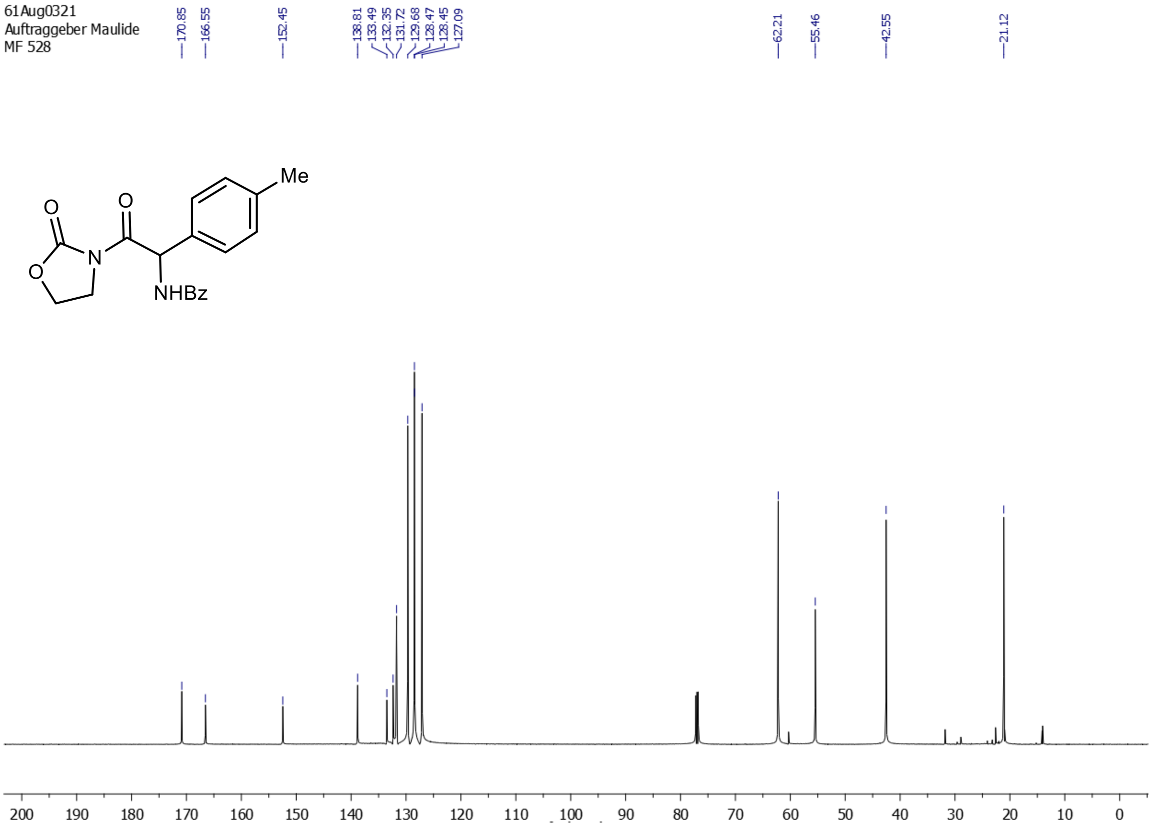
61Aug0321
Auftraggeber Maulide
MF 528
170.85 166.55
152.45
138.81 133.49 132.35 131.72 129.68 128.47 128.45 127.09
62.21 55.46
42.55
21.12
Me
O
O
N
O
NHBz
200 190 180 170 160 150 140 130 120 110 100 90 80 70 60 50 40 30 20 10 0

MF523P

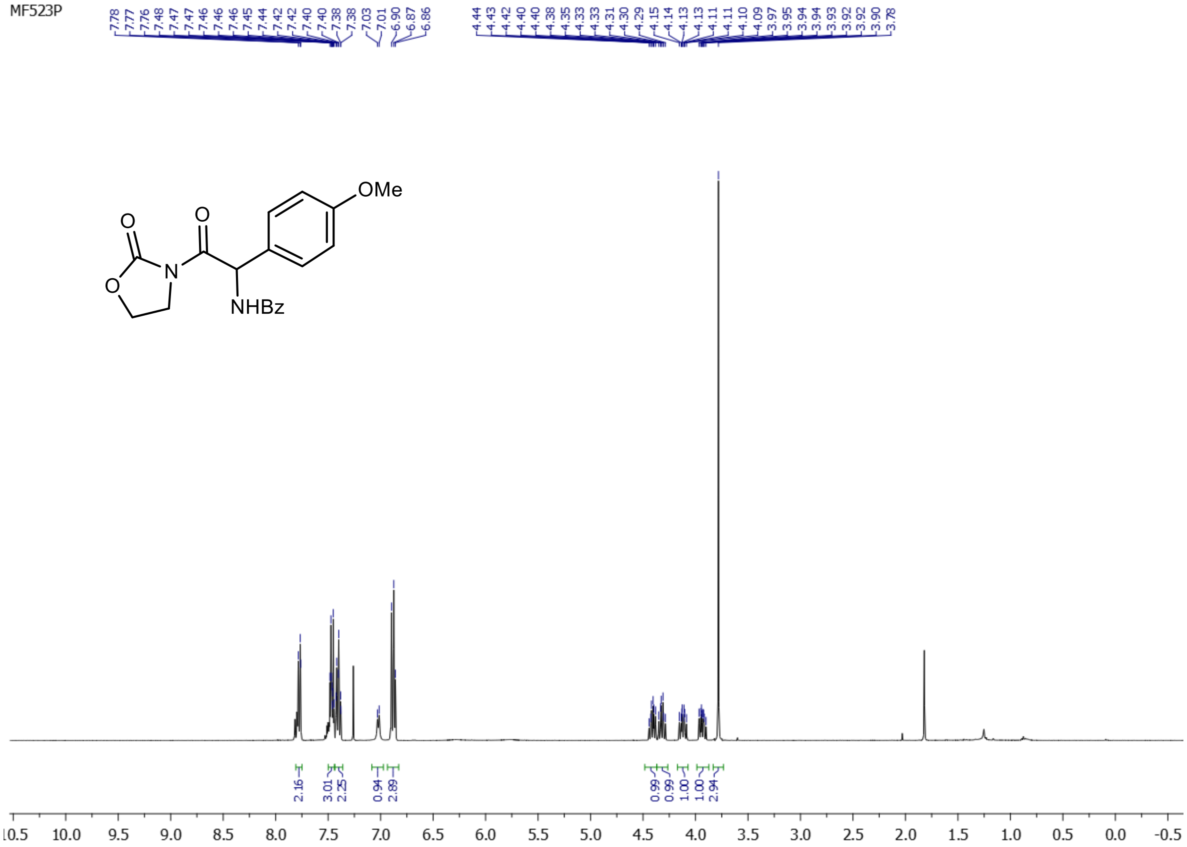

MF523P

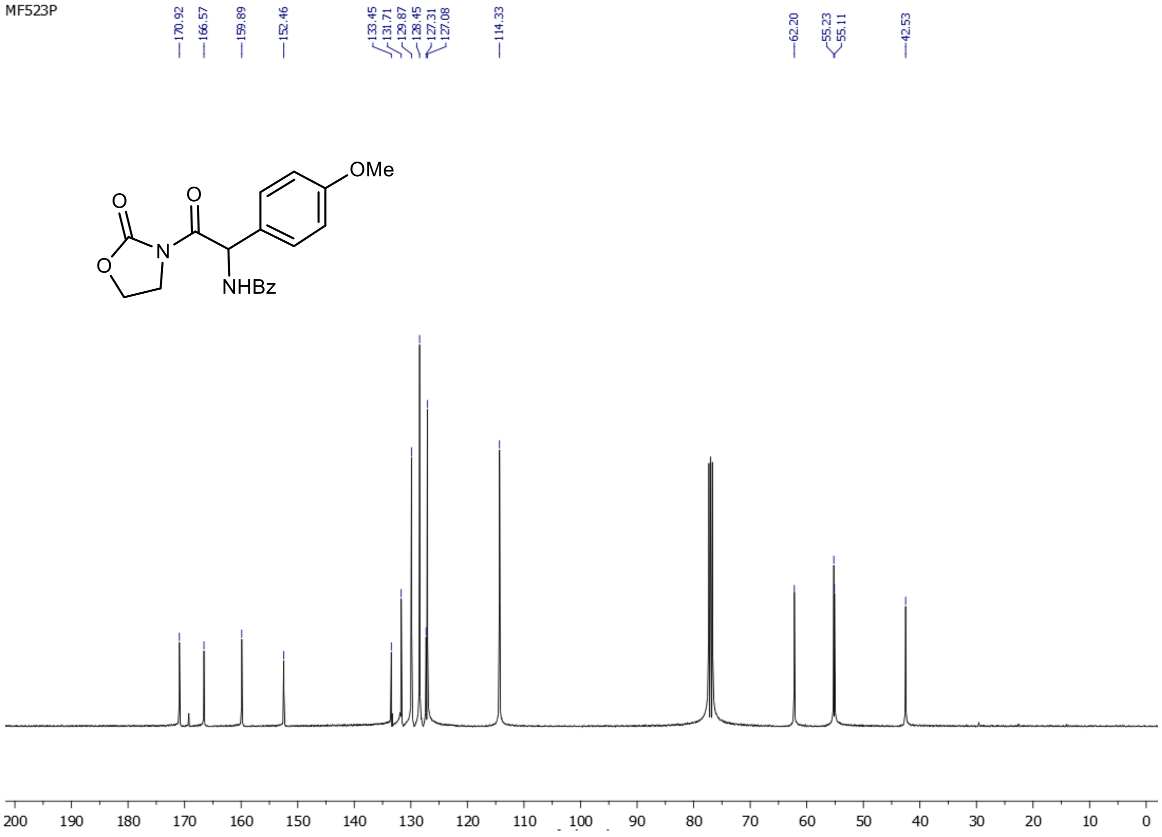

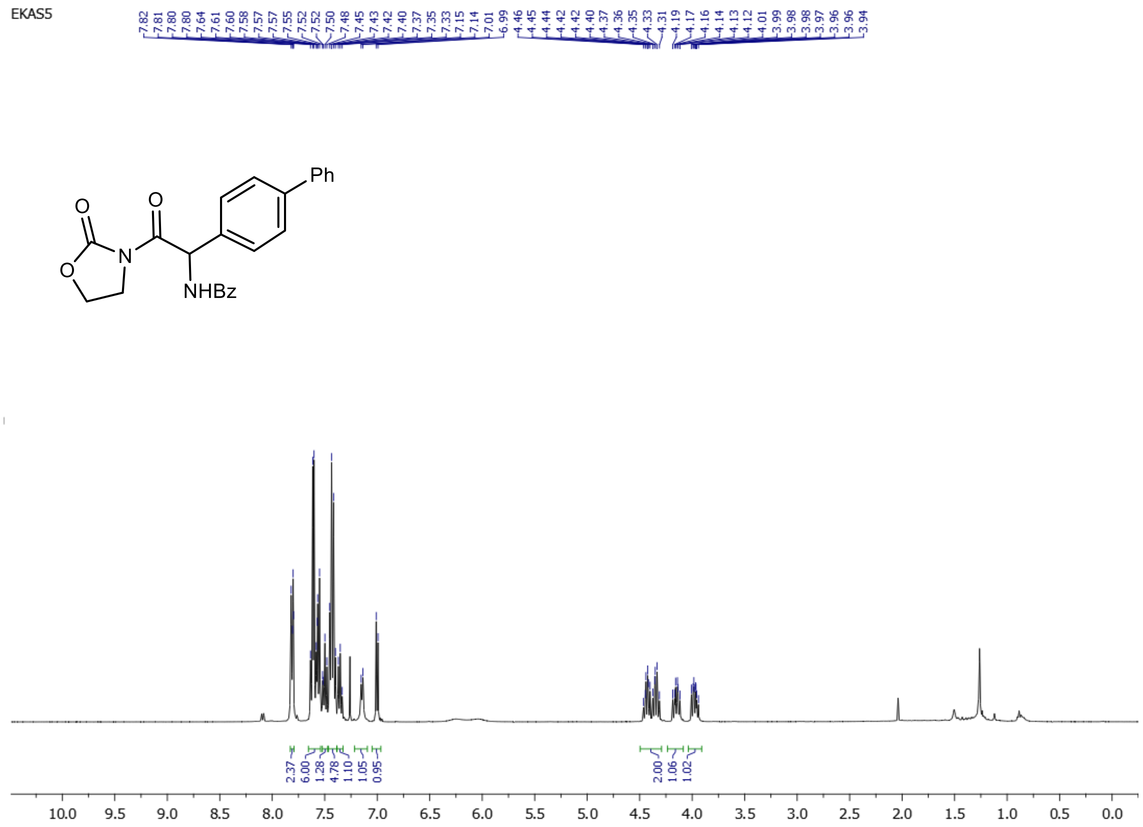
EKAS5
7.82 7.81 7.80 7.64 7.61 7.60 7.58 7.57 7.57 7.55 7.52 7.52 7.50 7.48 7.45 7.43 7.42 7.40 7.37 7.35 7.33 7.15 7.14 7.01 6.99 4.46 4.45 4.44 4.42 4.42 4.40 4.37 4.36 4.35 4.33 4.31 4.19 4.17 4.16 4.14 4.13 4.12 4.01 3.99 3.98 3.98 3.97 3.96 3.94
Ph
O
O
N
O
NHBz
2.37 6.00 1.28 4.78 1.10 1.05 0.95
2.00 1.06 1.02
10.0 9.5 9.0 8.5 8.0 7.5 7.0 6.5 6.0 5.5 5.0 4.5 4.0 3.5 3.0 2.5 2.0 1.5 1.0 0.5 0.0

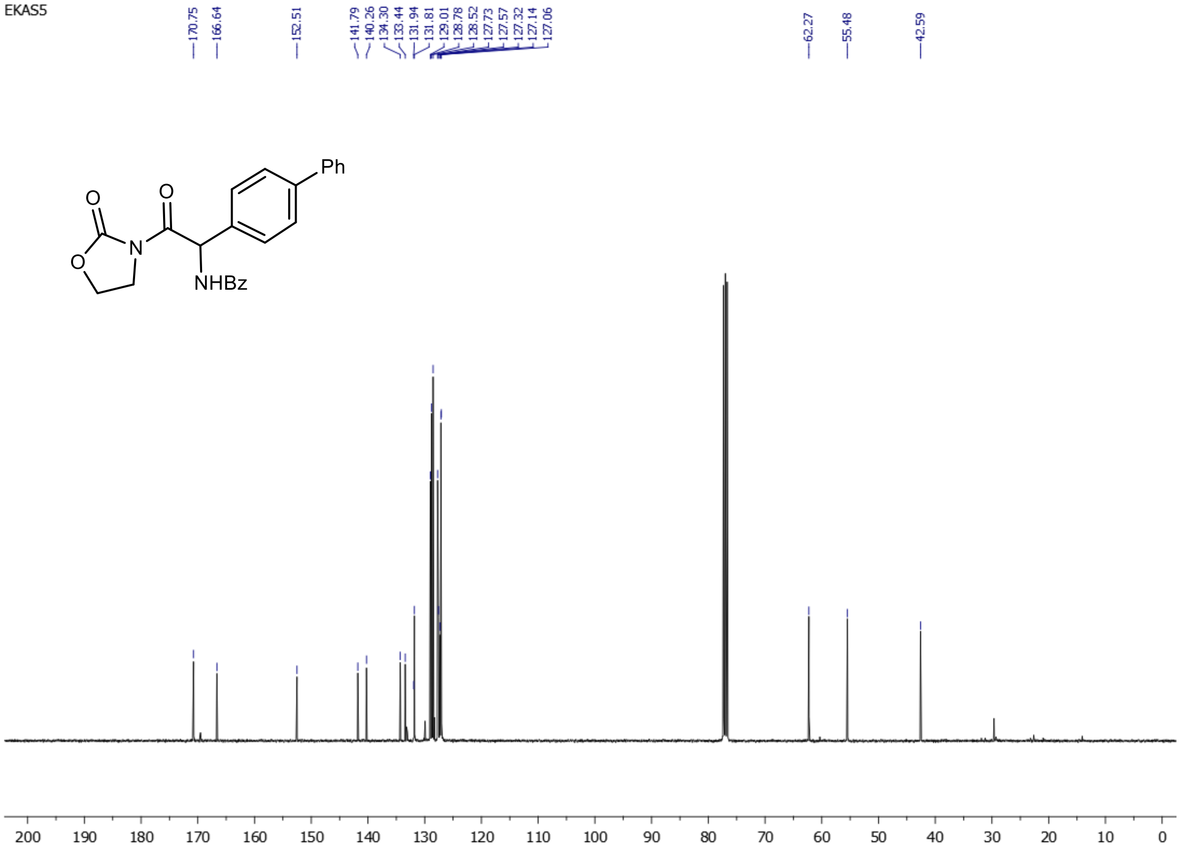
EKAS5
170.75 166.64 152.51 141.79 140.26 134.30 133.44 131.94 131.81 129.01 128.78 128.52 127.73 127.57 127.32 127.14 127.06 62.27 55.48 42.59
Ph
O
O
N
O
NHBz
200 190 180 170 160 150 140 130 120 110 100 90 80 70 60 50 40 30 20 10 0

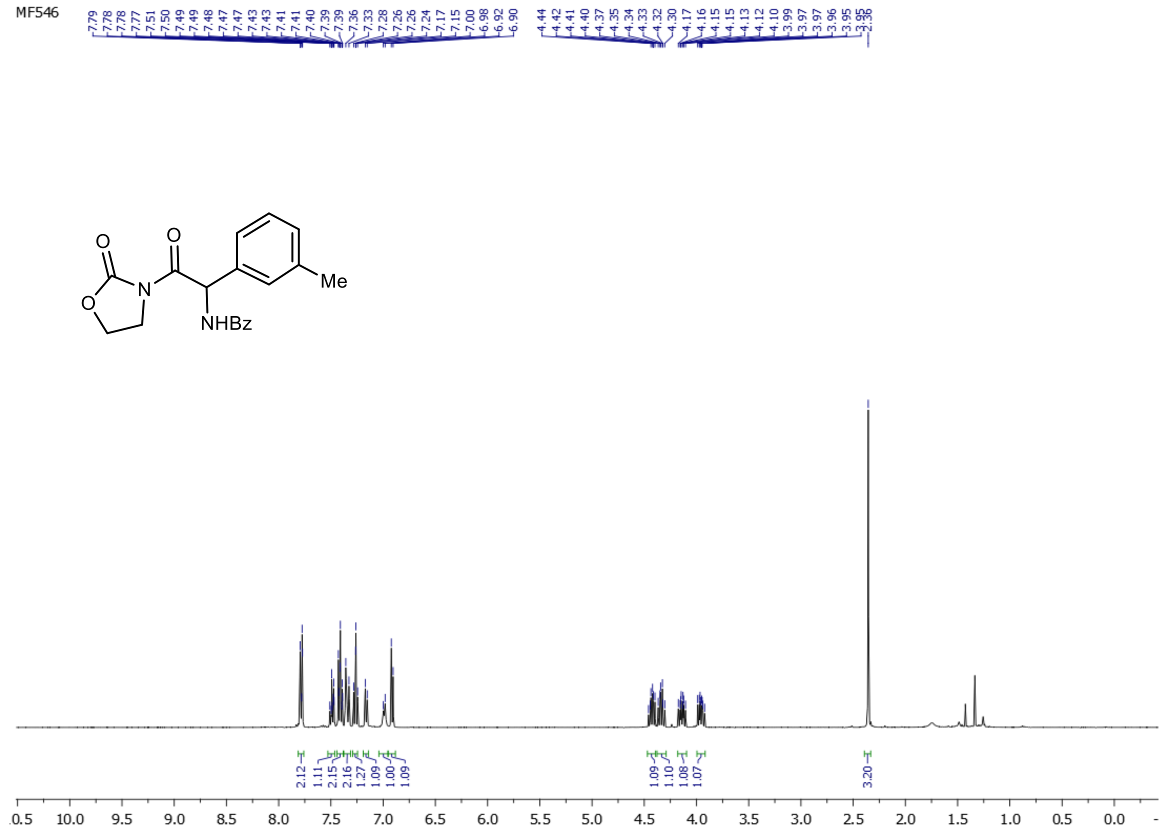
MF546
7.79 7.78 7.78 7.77 7.51 7.50 7.49 7.49 7.48 7.47 7.47 7.43 7.43 7.41 7.41 7.40 7.39 7.39 7.36 7.33 7.28 7.26 7.26 7.24 7.17 7.15 7.00 6.98 6.92 6.90
4.44 4.42 4.41 4.40 4.37 4.35 4.34 4.33 4.32 4.30 4.17 4.16 4.15 4.15 4.13 4.12 4.10 3.99 3.97 3.97 3.96 3.95 3.95 2.36
O
O
N
O
NHBz
Me
2.12 1.11 2.15 2.16 1.27 1.09 1.00 1.09
1.09 1.10 1.08 1.07
3.20
10.5 10.0 9.5 9.0 8.5 8.0 7.5 7.0 6.5 6.0 5.5 5.0 4.5 4.0 3.5 3.0 2.5 2.0 1.5 1.0 0.5 0.0

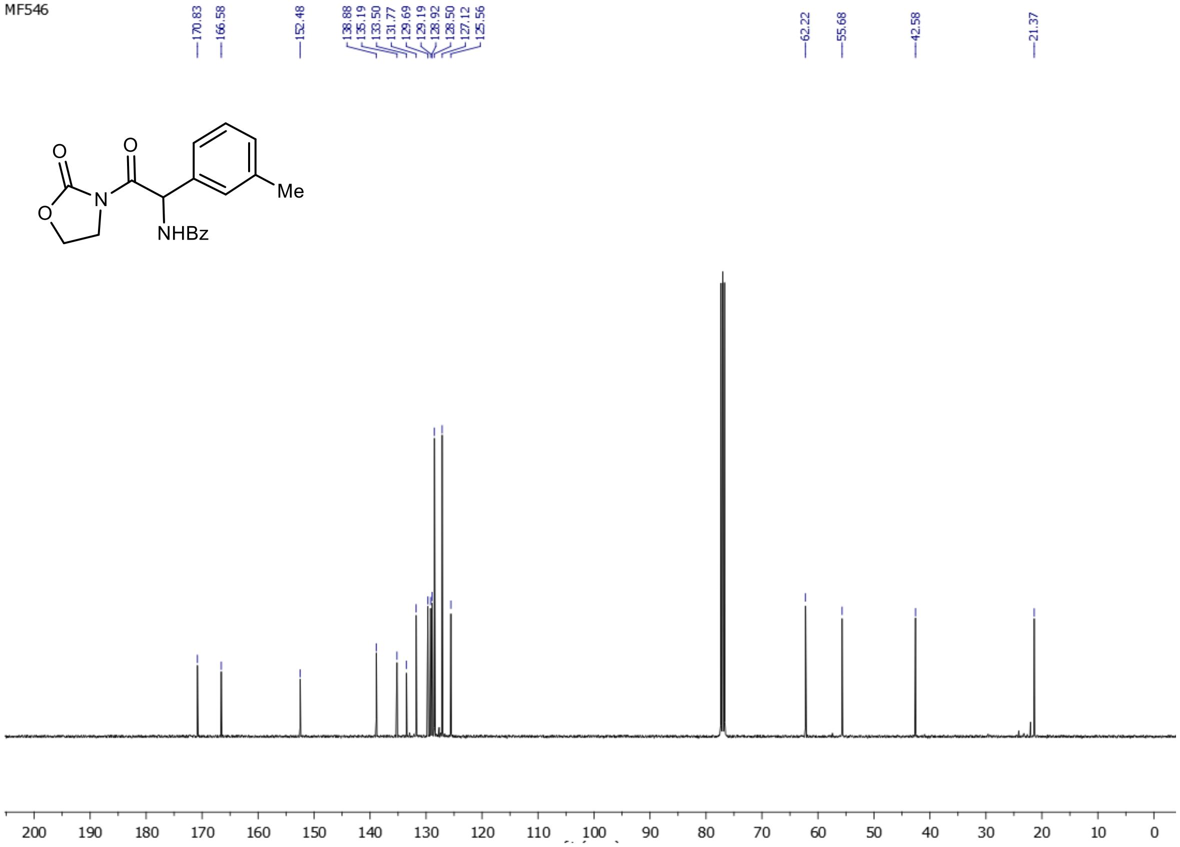
MF546
170.83 166.58 152.48 138.88 135.19 133.50 131.77 129.69 129.19 128.92 128.50 127.12 125.56 62.22 55.68 42.58 21.37
O
O
N
O
NHBz
Me
200 190 180 170 160 150 140 130 120 110 100 90 80 70 60 50 40 30 20 10 0

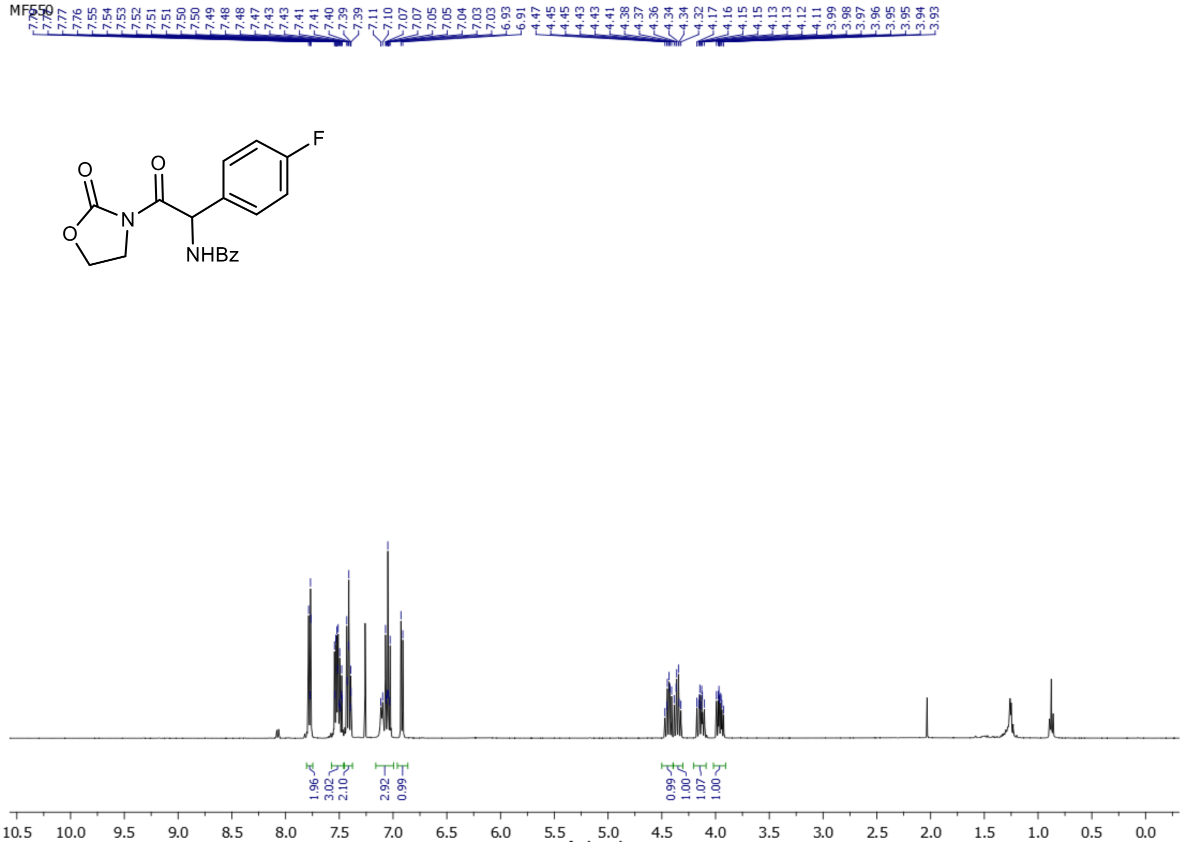
MF550
7.77 7.76 7.55 7.54 7.53 7.52 7.51 7.50 7.50 7.49 7.48 7.48 7.47 7.43 7.43 7.41 7.41 7.40 7.39 7.39 7.11 7.10 7.07 7.07 7.05 7.05 7.04 7.03 7.03 6.93 6.91 4.47 4.45 4.45 4.43 4.43 4.41 4.38 4.37 4.36 4.34 4.34 4.32 4.17 4.16 4.15 4.15 4.13 4.13 4.12 4.11 3.99 3.98 3.97 3.96 3.95 3.95 3.94 3.93
F
O
O
N
O
NHBz
1.96 3.02 2.10 2.92 0.99 0.99 1.00 1.07 1.00
10.5 10.0 9.5 9.0 8.5 8.0 7.5 7.0 6.5 6.0 5.5 5.0 4.5 4.0 3.5 3.0 2.5 2.0 1.5 1.0 0.5 0.0

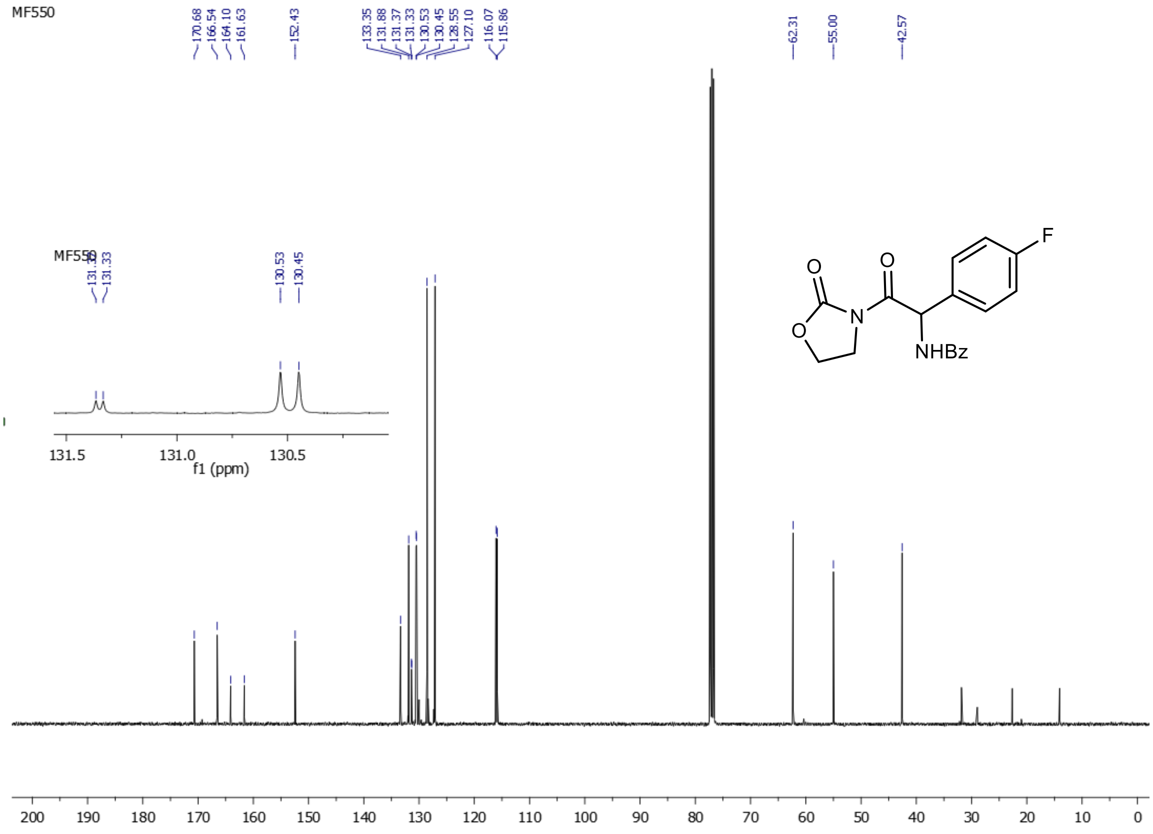
MF550
170.68 166.54 164.10 161.63 152.43 133.35 131.88 131.37 131.33 130.53 130.45 128.55 127.10 116.07 115.86 62.31 55.00 42.57
MF550
131.37 131.33 130.53 130.45
131.5 131.0 130.5
f1 (ppm)
F
O
O
N
O
NHBz
200 190 180 170 160 150 140 130 120 110 100 90 80 70 60 50 40 30 20 10 0

MF550

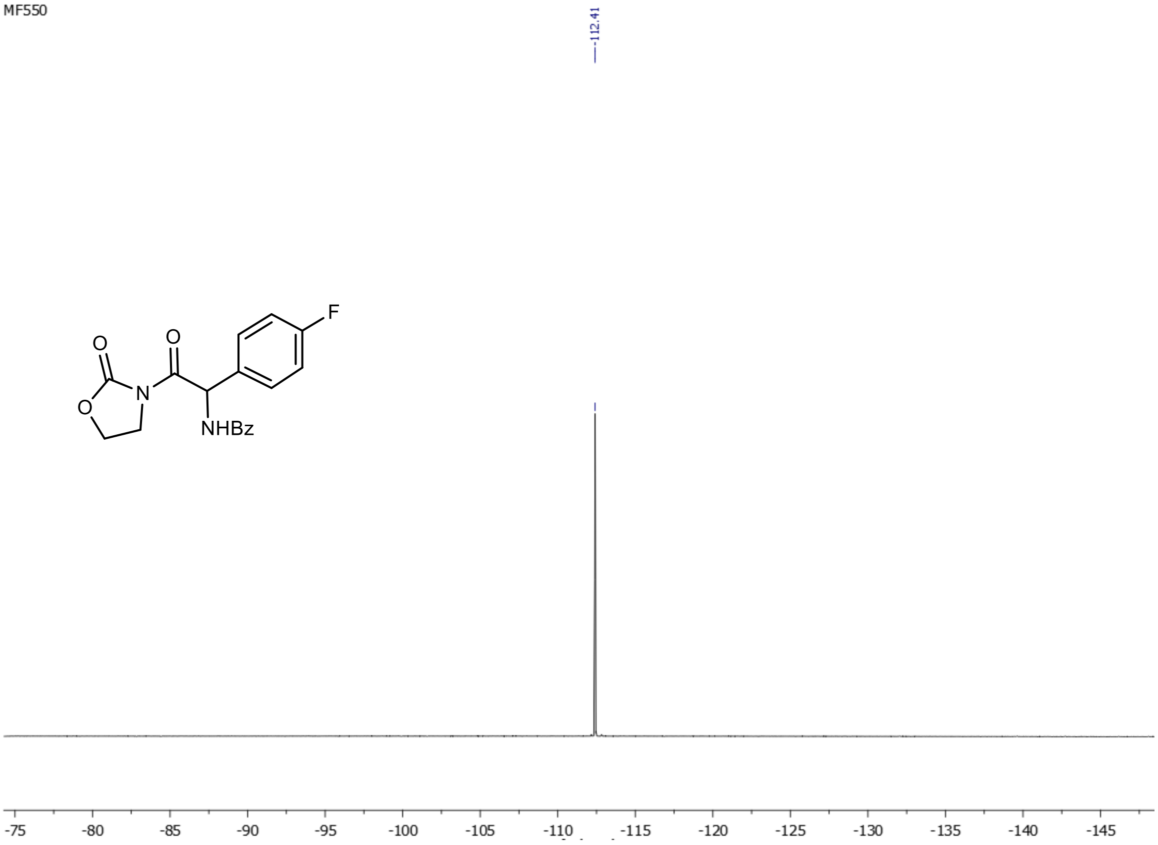
-112.41
O
O
N
O
F
NHBz
-75
-80
-85
-90
-95
-100
-105
-110
-115
-120
-125
-130
-135
-140
-145

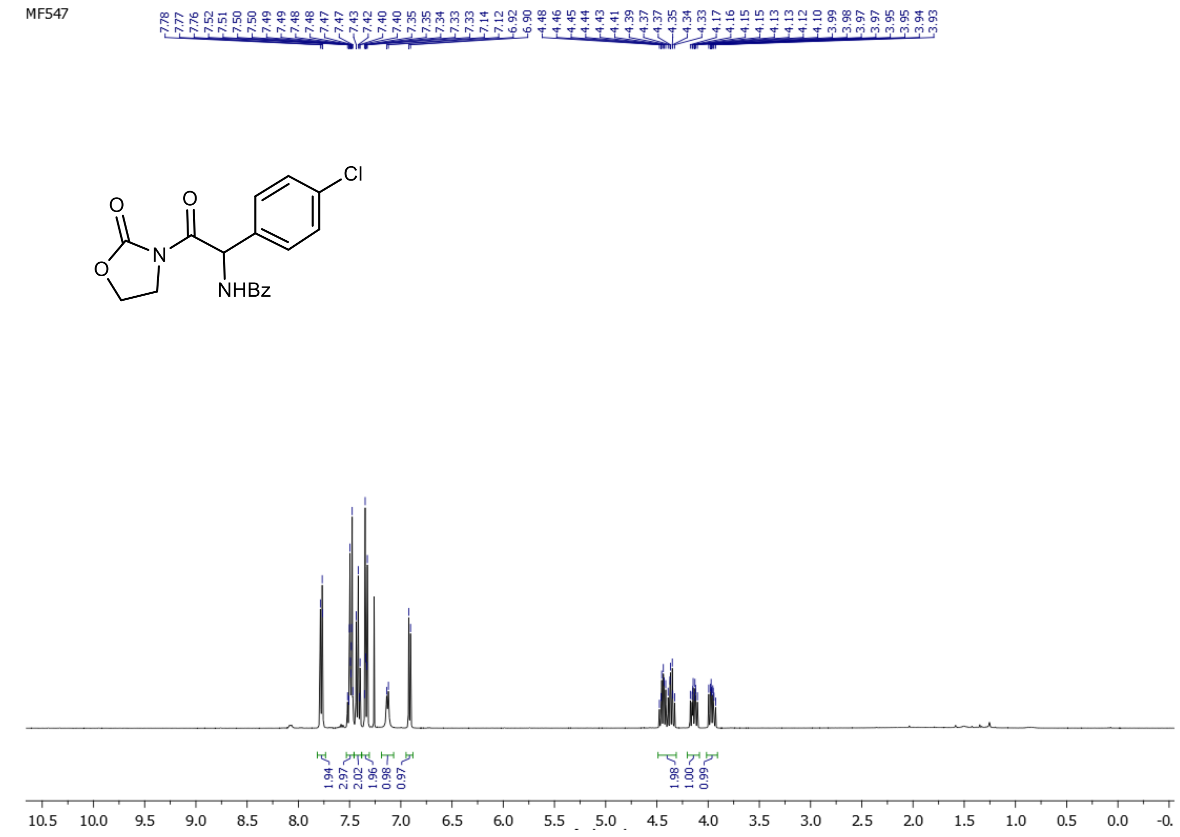

MF547
7.78 7.77 7.76 7.52 7.51 7.50 7.50 7.49 7.49 7.48 7.48 7.47 7.47 7.43 7.42 7.40 7.40 7.35 7.35 7.34 7.33 7.33 7.14 7.12 6.92 6.90 4.48 4.46 4.45 4.44 4.43 4.41 4.39 4.37 4.37 4.35 4.34 4.33 4.17 4.16 4.15 4.15 4.13 4.13 4.12 4.10 3.99 3.98 3.97 3.97 3.95 3.95 3.94 3.93
Cl
O
N
NHBz
1.94 2.97 2.02 1.96 0.98 0.97 1.98 1.00 0.99

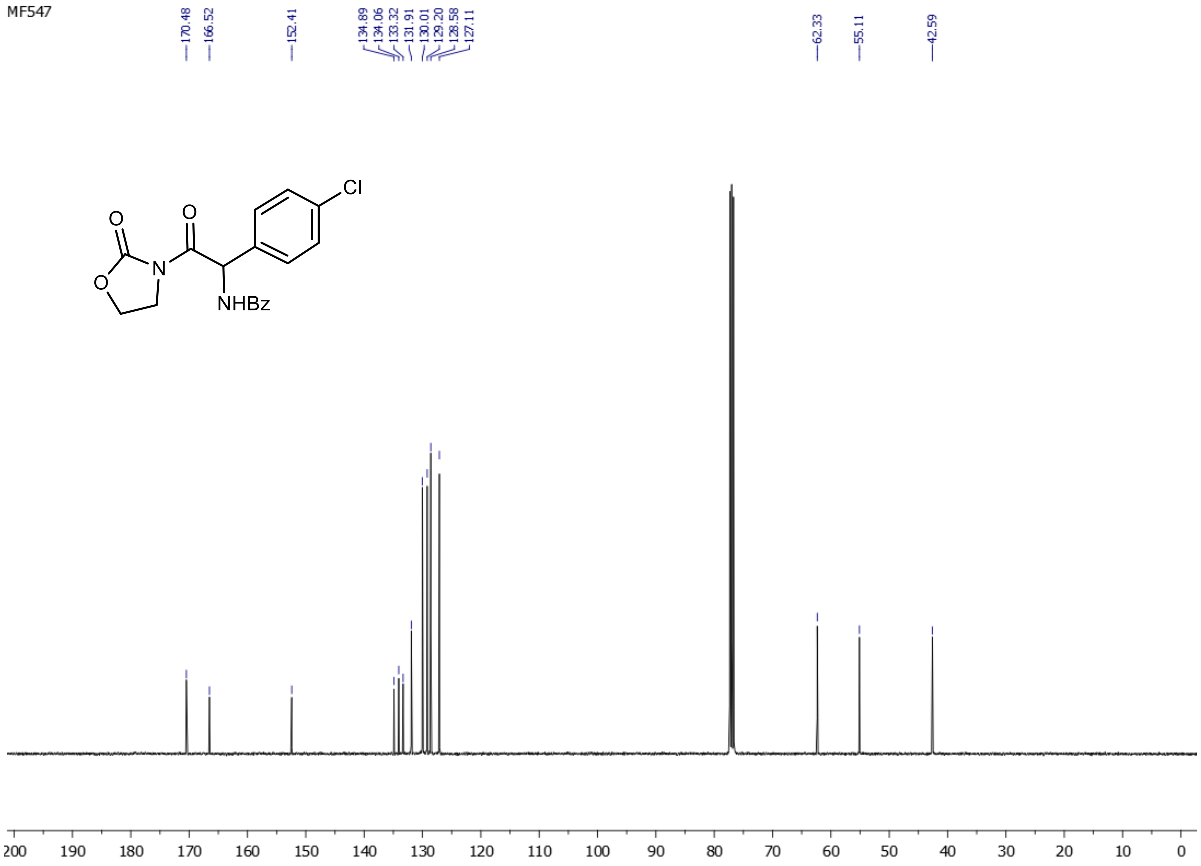

MF547
170.48 166.52 152.41 134.89 134.06 133.32 131.91 130.01 129.20 128.58 127.11 62.33 55.11 42.59
Cl
O
N
NHBz

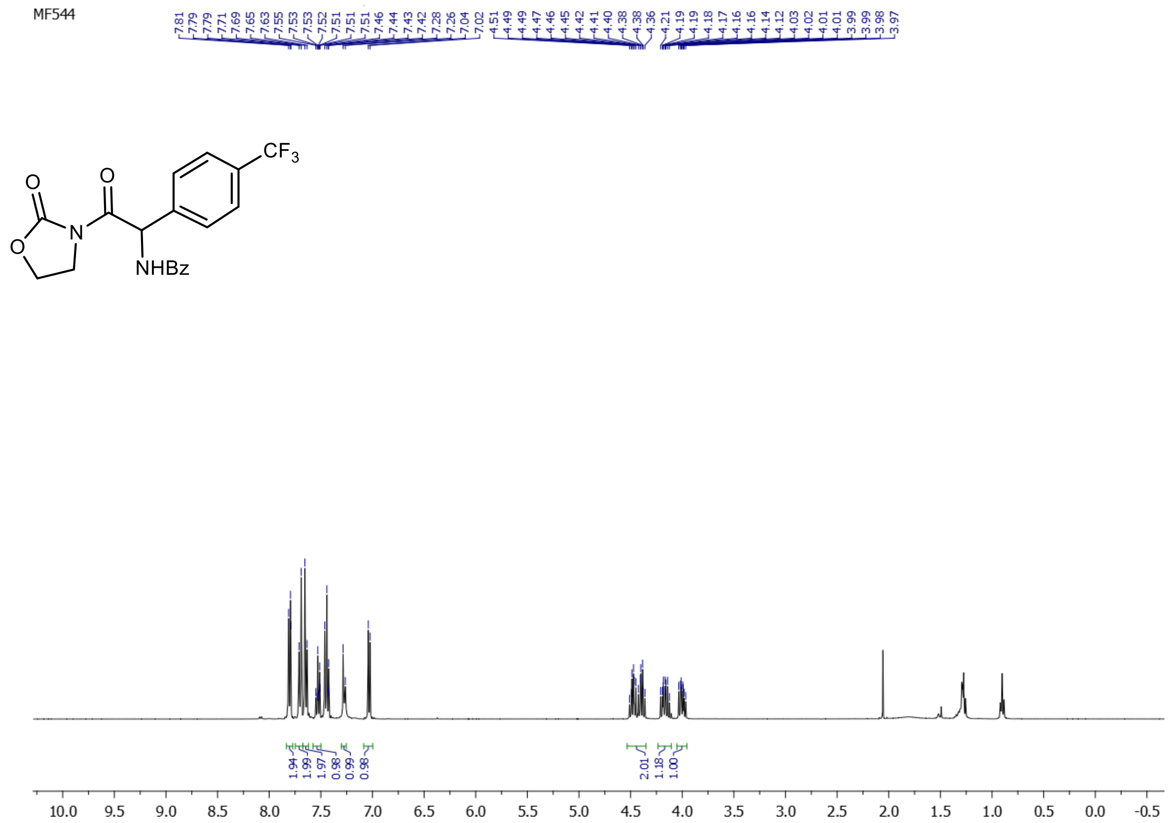
MF544
7.81 7.79 7.71 7.69 7.65 7.63 7.55 7.53 7.53 7.52 7.51 7.51 7.51 7.46 7.44 7.43 7.42 7.28 7.26 7.04 7.02 4.51 4.49 4.47 4.46 4.45 4.42 4.41 4.40 4.38 4.38 4.36 4.21 4.19 4.19 4.18 4.17 4.16 4.14 4.12 4.03 4.02 4.01 4.01 3.99 3.98 3.97
CF3
O
N
O
NHBz
1.94 1.99 1.97 0.98 0.99 0.98 2.01 1.18 1.00
10.0 9.5 9.0 8.5 8.0 7.5 7.0 6.5 6.0 5.5 5.0 4.5 4.0 3.5 3.0 2.5 2.0 1.5 1.0 0.5 0.0 -0.5

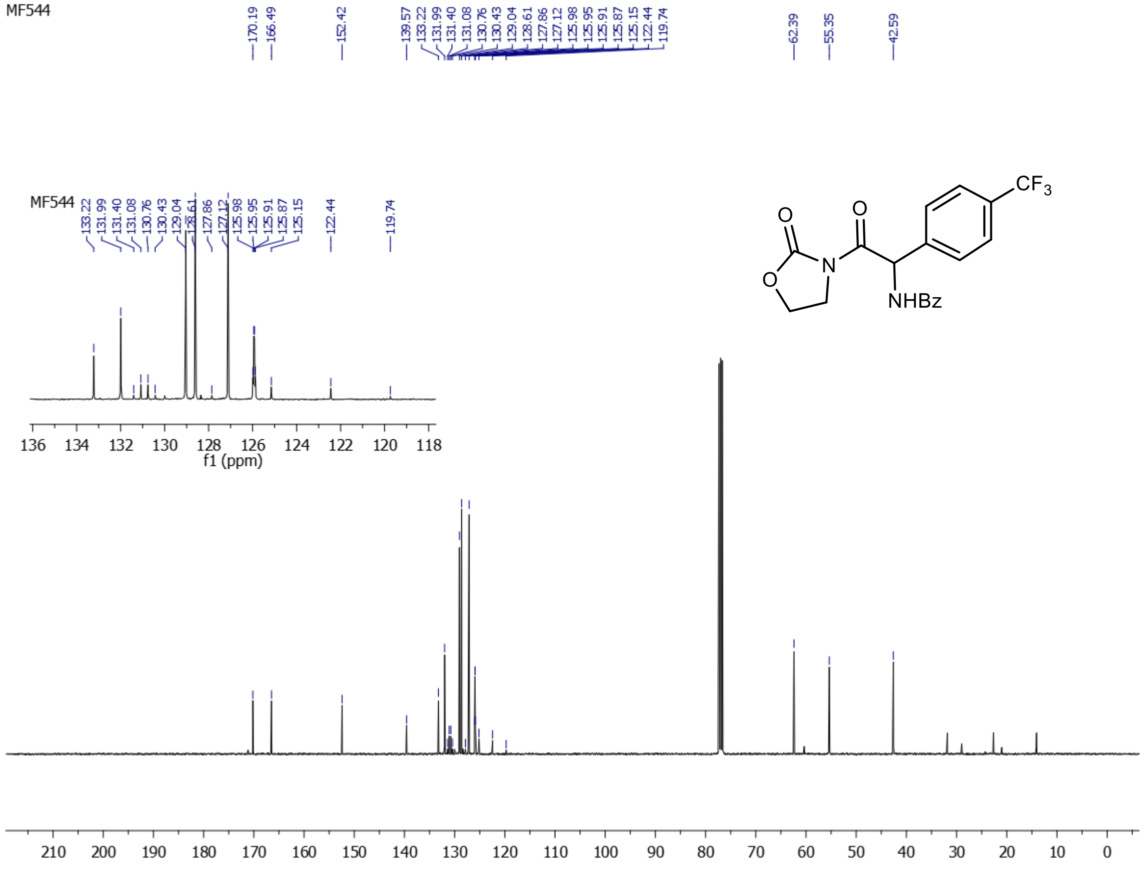
MF544
170.19 166.49 152.42 139.57 133.22 131.99 131.40 131.08 130.76 130.43 129.04 128.61 127.86 127.12 125.98 125.95 125.91 125.87 125.15 122.44 119.74 62.39 55.35 42.59
MF544
133.22 131.99 131.40 131.08 130.76 130.43 129.04 128.61 127.86 127.12 125.98 125.95 125.91 125.87 125.15 122.44 119.74
136 134 132 130 128 126 124 122 120 118
f1 (ppm)
CF3
O
N
O
NHBz
210 200 190 180 170 160 150 140 130 120 110 100 90 80 70 60 50 40 30 20 10 0

MF544

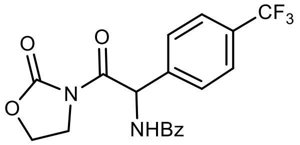

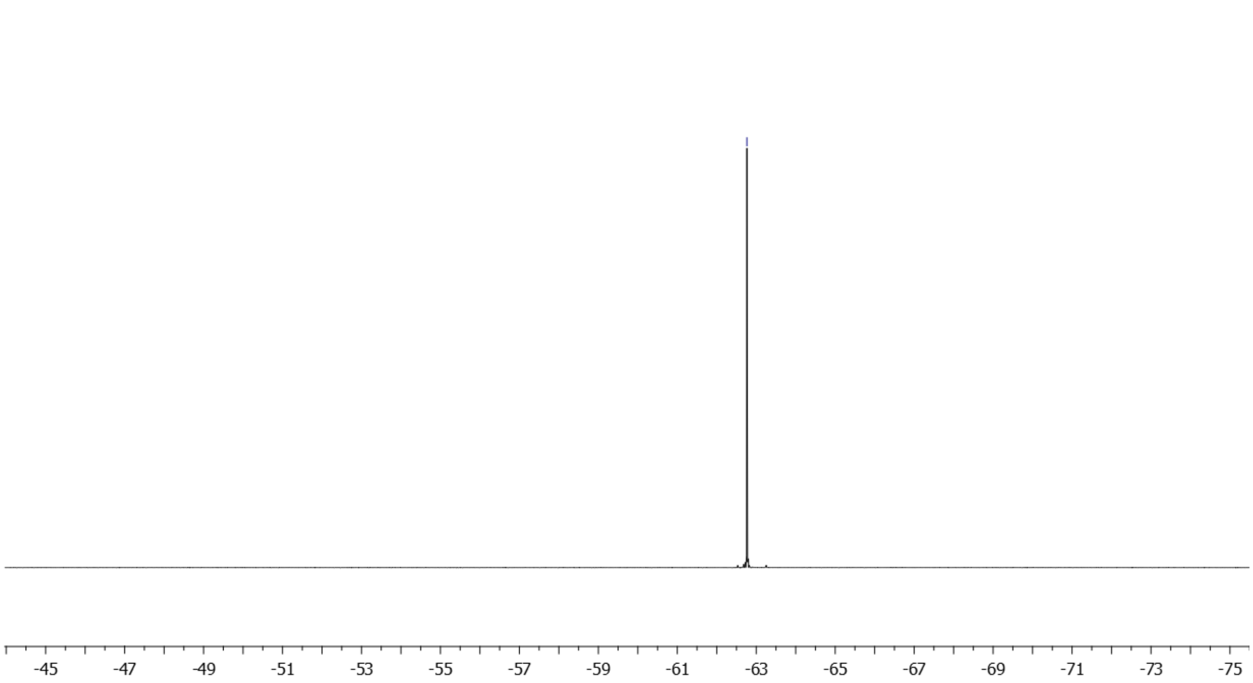

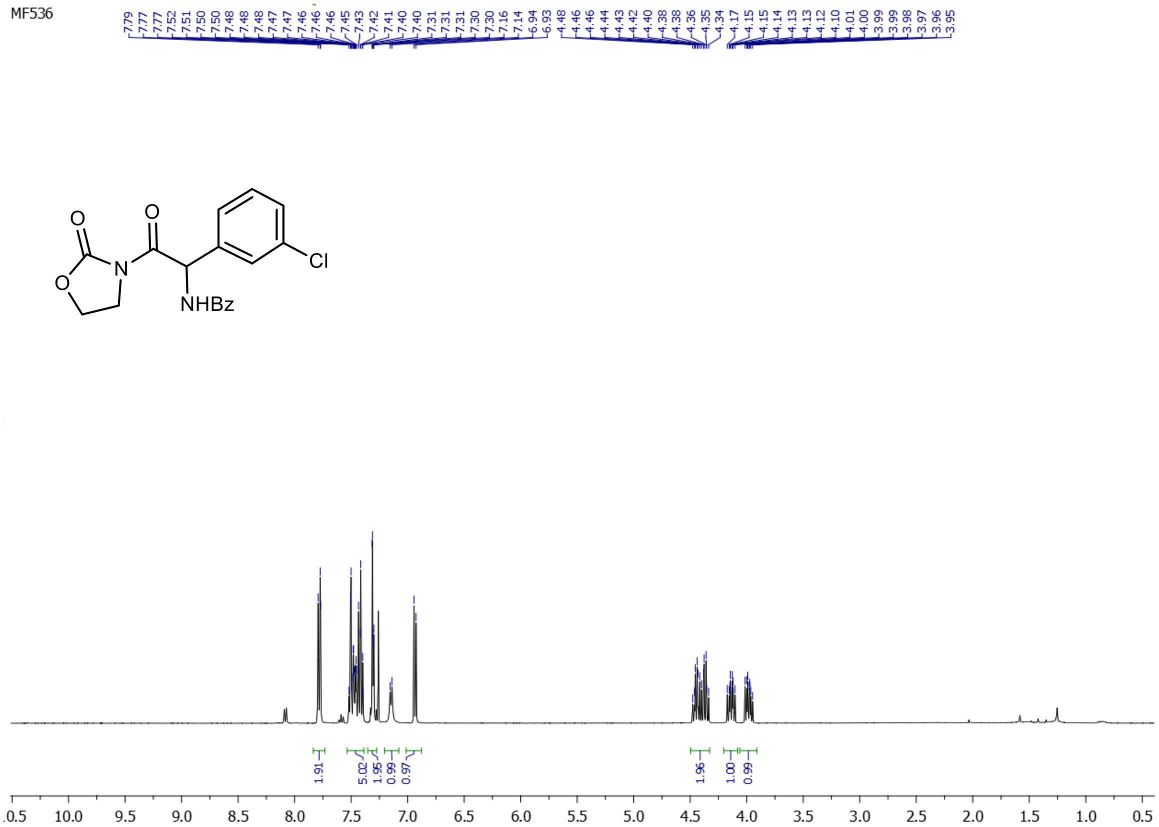
MF536
7.79
7.77
7.77
7.52
7.51
7.50
7.48
7.48
7.47
7.47
7.46
7.46
7.45
7.43
7.42
7.41
7.40
7.40
7.31
7.31
7.31
7.30
7.16
7.14
6.94
6.93
4.48
4.46
4.44
4.43
4.42
4.40
4.38
4.38
4.36
4.35
4.34
4.17
4.15
4.14
4.13
4.13
4.12
4.10
4.01
4.00
3.99
3.98
3.97
3.96
3.95
O
O
N
O
Cl
NHBz
1.91
5.02
1.95
0.99
0.97
1.96
1.00
0.99
10.5
10.0
9.5
9.0
8.5
8.0
7.5
7.0
6.5
6.0
5.5
5.0
4.5
4.0
3.5
3.0
2.5
2.0
1.5
1.0
0.5

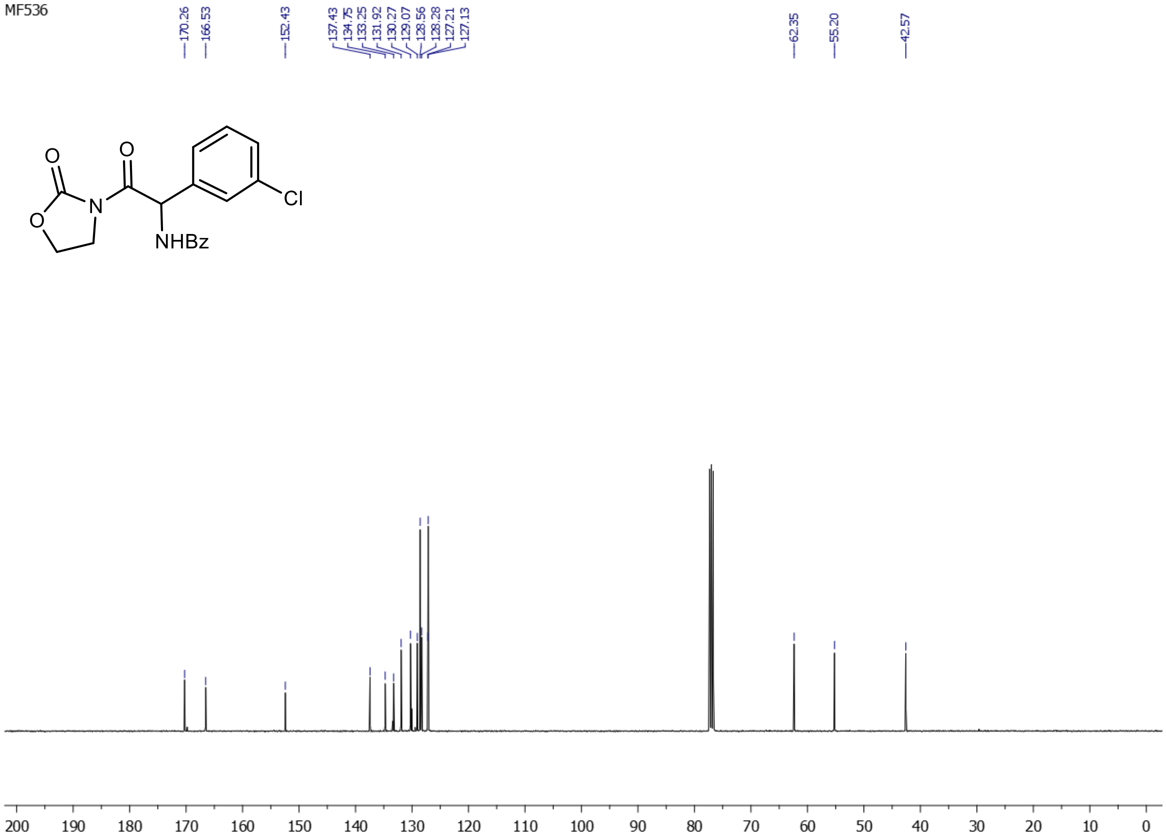
MF536
170.26
166.53
152.43
137.43
134.75
133.25
131.92
130.27
129.07
128.56
128.28
127.21
127.13
62.35
55.20
42.57
O
O
N
O
Cl
NHBz
200
190
180
170
160
150
140
130
120
110
100
90
80
70
60
50
40
30
20
10
0

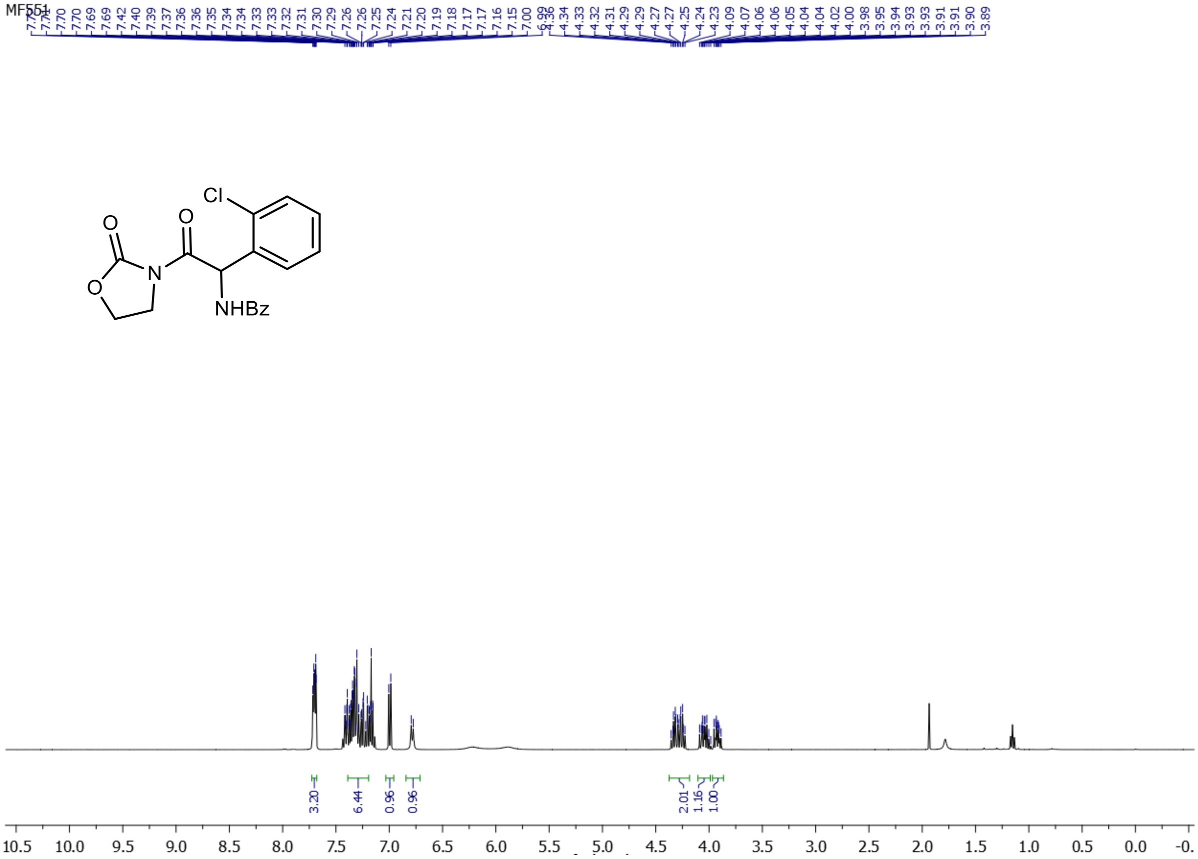
MF551
7.70
7.69
7.69
7.42
7.40
7.39
7.37
7.36
7.36
7.35
7.34
7.34
7.33
7.33
7.32
7.31
7.30
7.29
7.26
7.26
7.25
7.24
7.21
7.20
7.19
7.18
7.17
7.17
7.16
7.15
7.00
4.34
4.33
4.32
4.31
4.29
4.29
4.27
4.25
4.24
4.23
4.09
4.07
4.06
4.05
4.04
4.04
4.02
4.00
3.98
3.95
3.94
3.93
3.93
3.91
3.91
3.90
3.89
Cl
O
O
N
O
NHBz
3.20
6.44
0.96
0.96
2.01
1.16
1.00

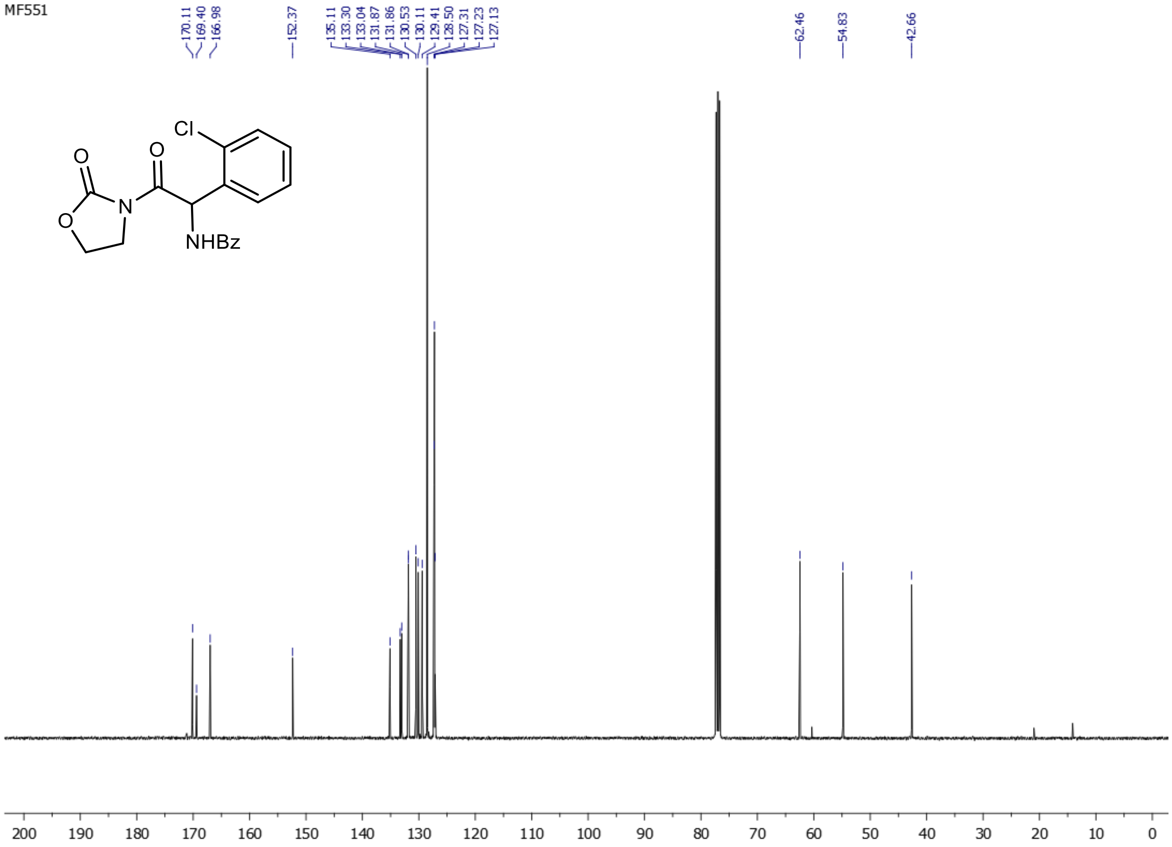
MF551
170.11
169.40
166.98
152.37
135.11
133.30
133.04
131.87
131.86
130.53
130.11
129.41
128.50
127.31
127.23
127.13
62.46
54.83
42.66
Cl
O
O
N
O
NHBz

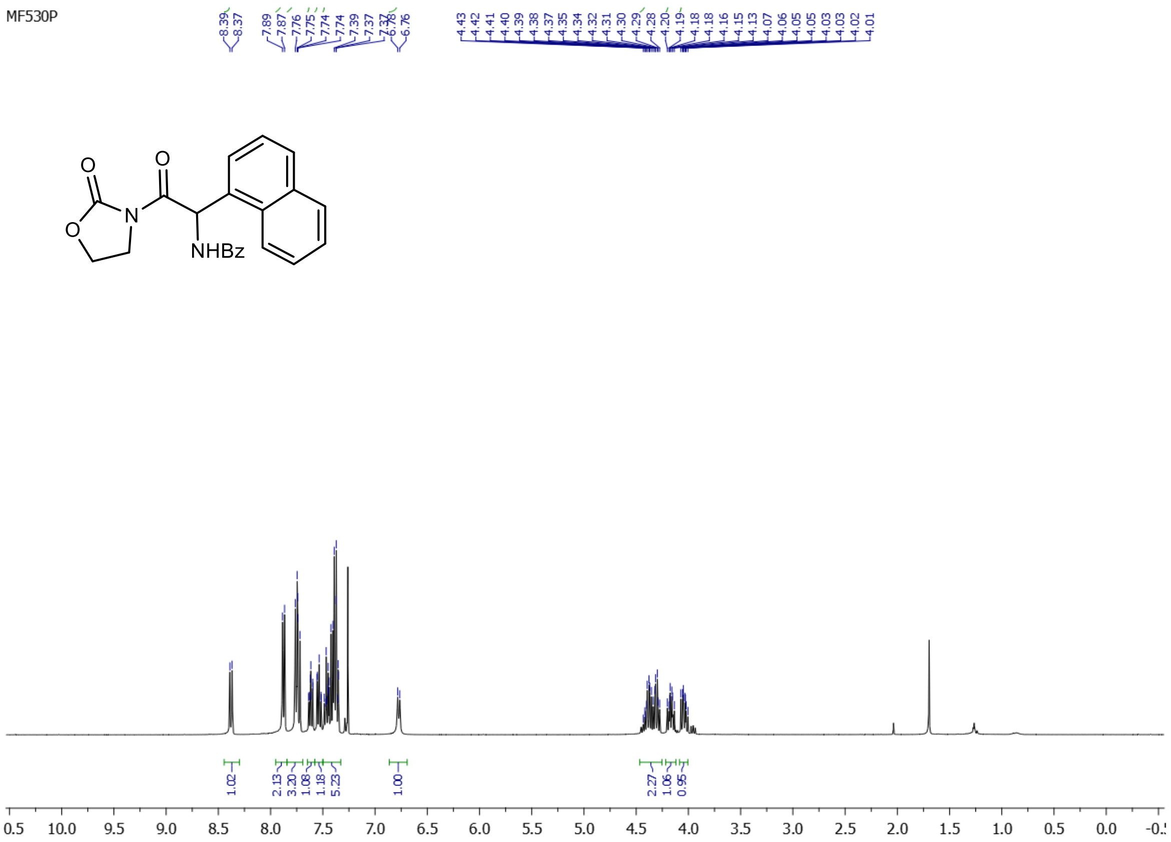
MF530P
O
O
N
O
NHBz

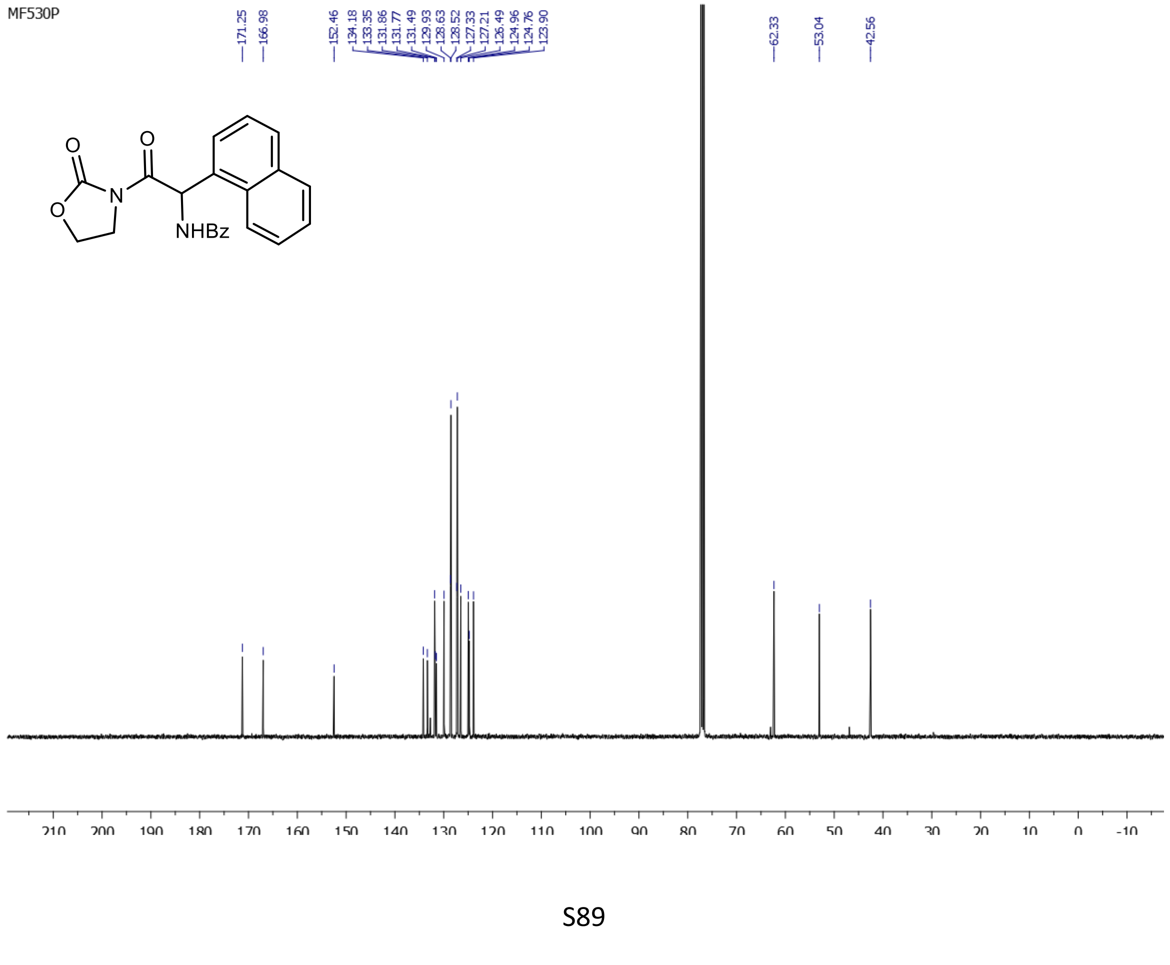
MF530P
171.25
166.98
152.46
134.18
133.35
131.86
131.77
131.49
129.93
128.63
128.52
127.33
127.21
126.49
124.96
124.76
123.90
62.33
53.04
42.56
O
O
N
O
NHBz

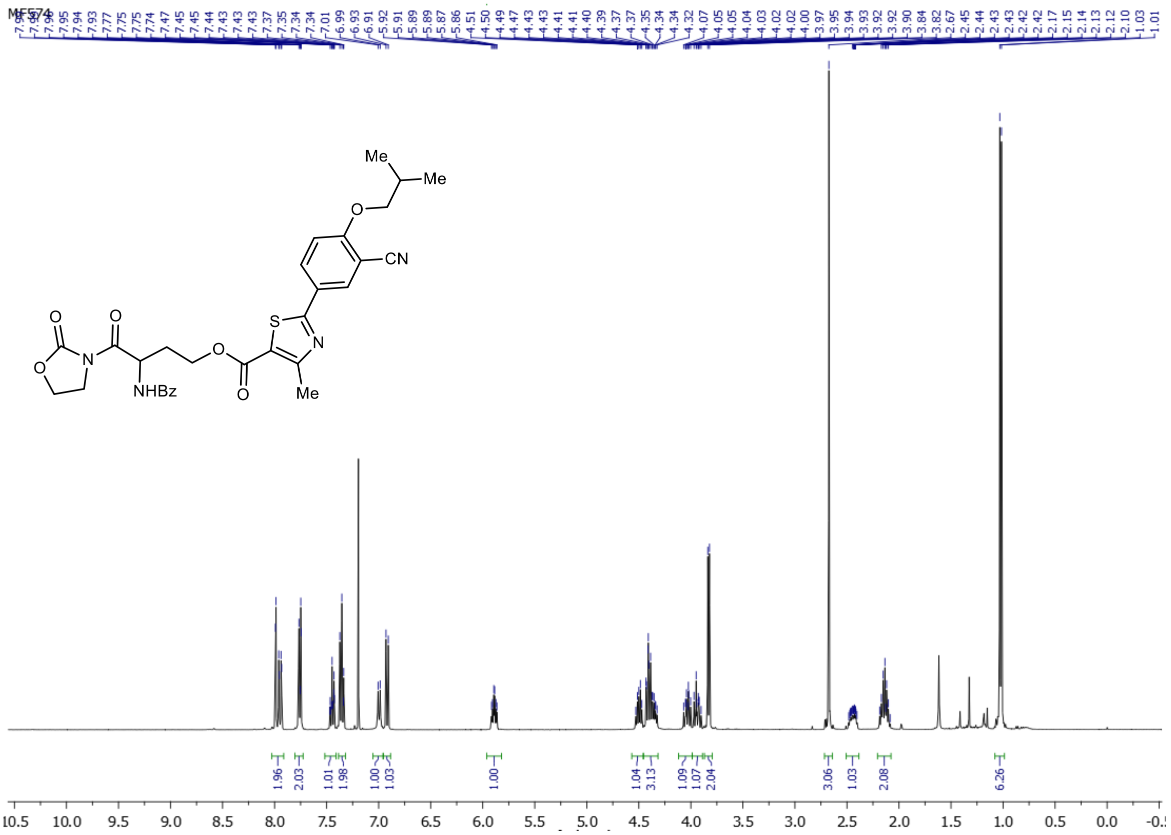
MF574
7.95
7.94
7.93
7.77
7.75
7.75
7.74
7.47
7.45
7.45
7.44
7.43
7.43
7.43
7.37
7.35
7.34
7.34
7.01
6.99
6.93
6.91
5.92
5.91
5.89
5.87
5.86
4.51
4.50
4.49
4.47
4.43
4.43
4.41
4.41
4.40
4.39
4.37
4.37
4.35
4.34
4.34
4.32
4.07
4.05
4.05
4.04
4.03
4.02
4.02
4.00
3.97
3.95
3.94
3.93
3.93
3.92
3.92
3.90
3.84
3.82
2.67
2.45
2.44
2.43
2.43
2.42
2.42
2.17
2.15
2.14
2.13
2.12
2.10
1.03
1.01
Me
Me
O
CN
S
N
O
O
O
N
O
NHBz
O
Me
1.96
2.03
1.01
1.98
1.00
1.03
1.00
1.04
3.13
1.09
1.07
2.04
3.06
1.03
2.08
6.26
10.5
10.0
9.5
9.0
8.5
8.0
7.5
7.0
6.5
6.0
5.5
5.0
4.5
4.0
3.5
3.0
2.5
2.0
1.5
1.0
0.5
0.0
-0.5

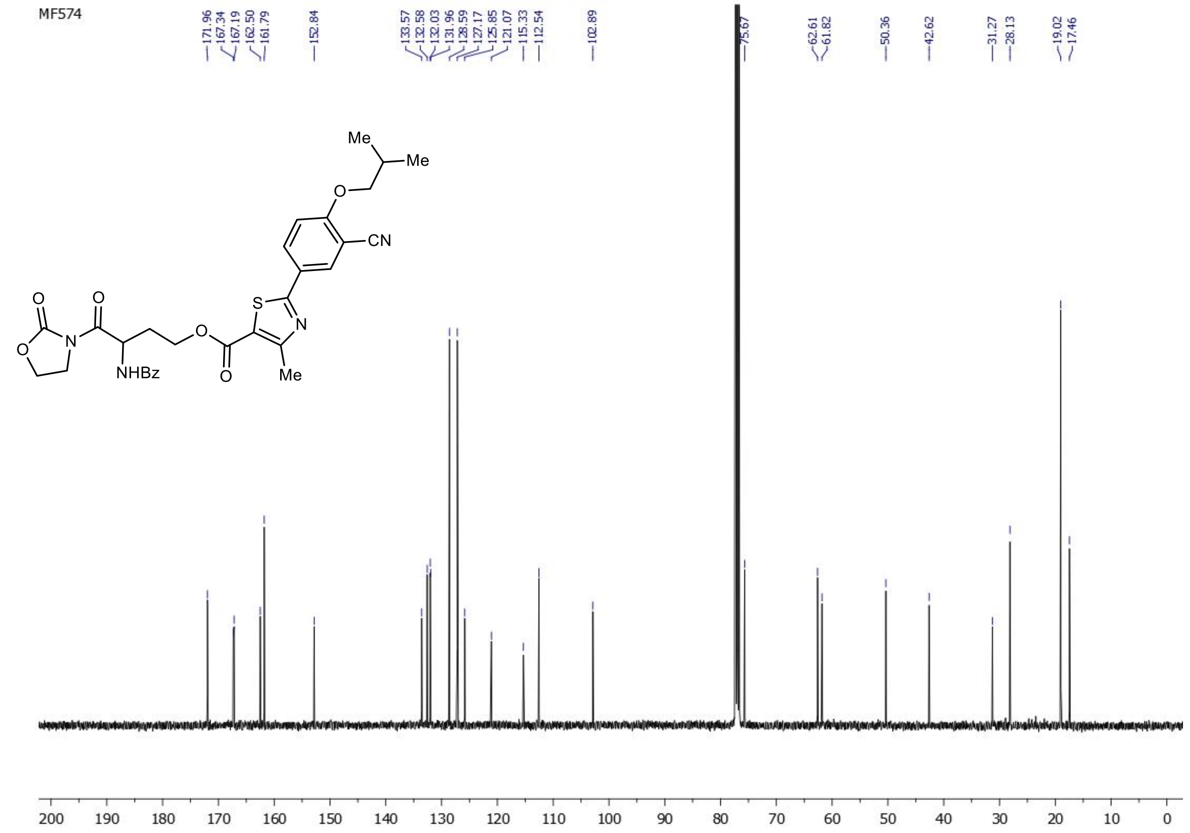
MF574
171.96
167.34
167.19
162.50
161.79
152.84
133.57
132.58
132.03
131.96
128.59
127.17
125.85
121.07
115.33
112.54
102.89
75.67
62.61
61.82
50.36
42.62
31.27
28.13
19.02
17.46
Me
Me
O
CN
S
N
O
O
O
N
O
NHBz
O
Me
200
190
180
170
160
150
140
130
120
110
100
90
80
70
60
50
40
30
20
10
0

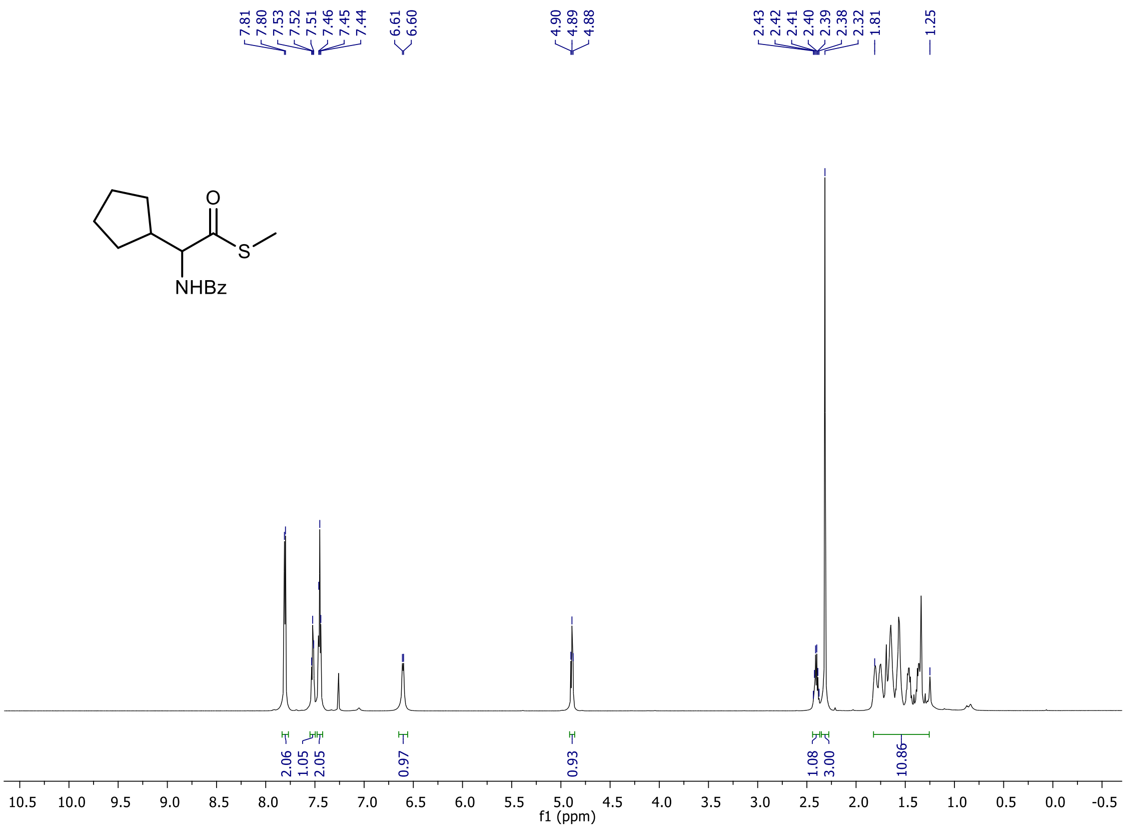

NHBz
O
S
f1 (ppm)

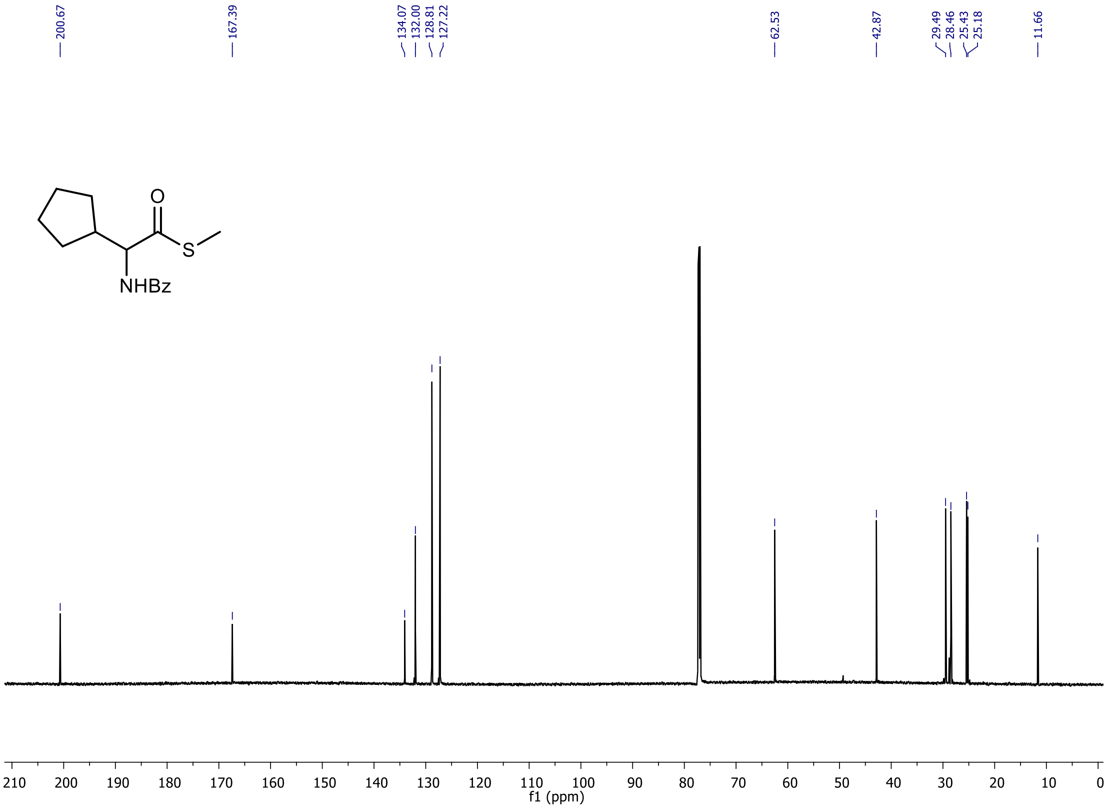

NHBz
O
S
f1 (ppm)

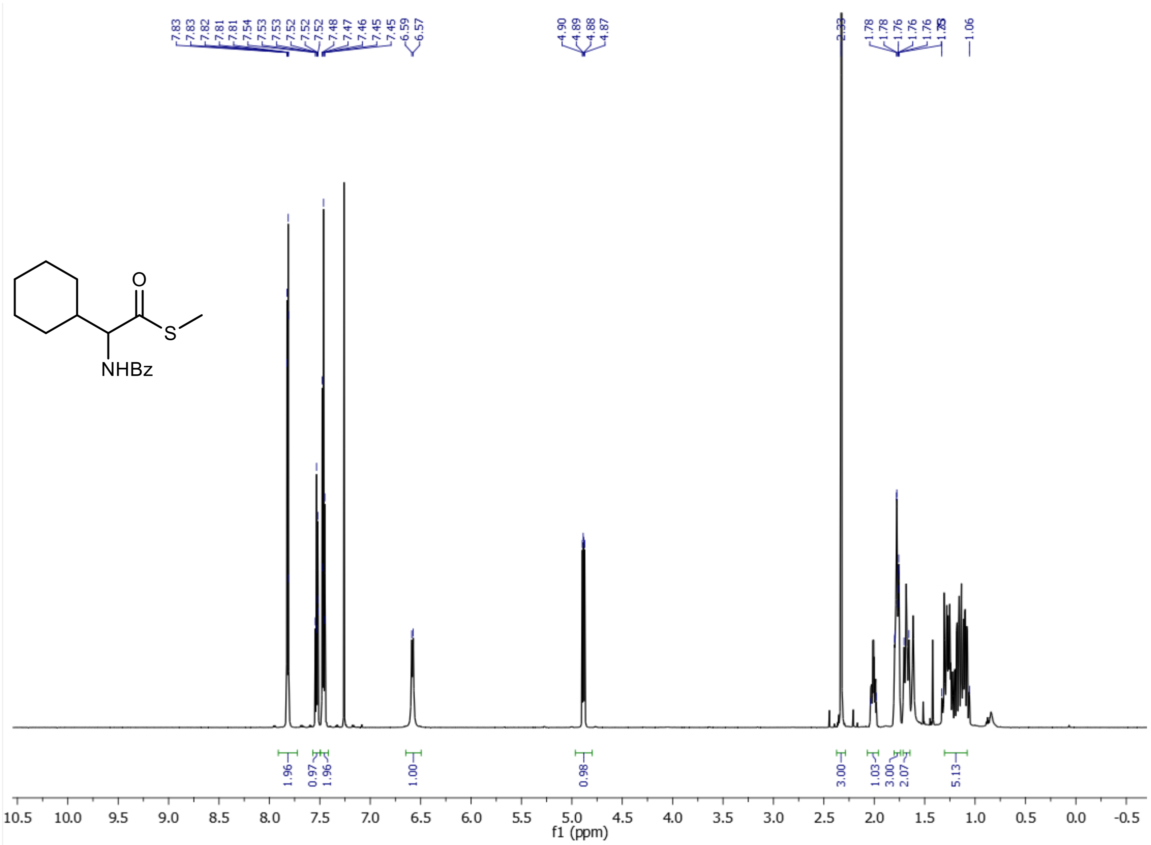

NHBz
O
S
1.96
0.97
1.96
1.00
0.98
3.00
1.03
3.00
2.07
5.13
f1 (ppm)

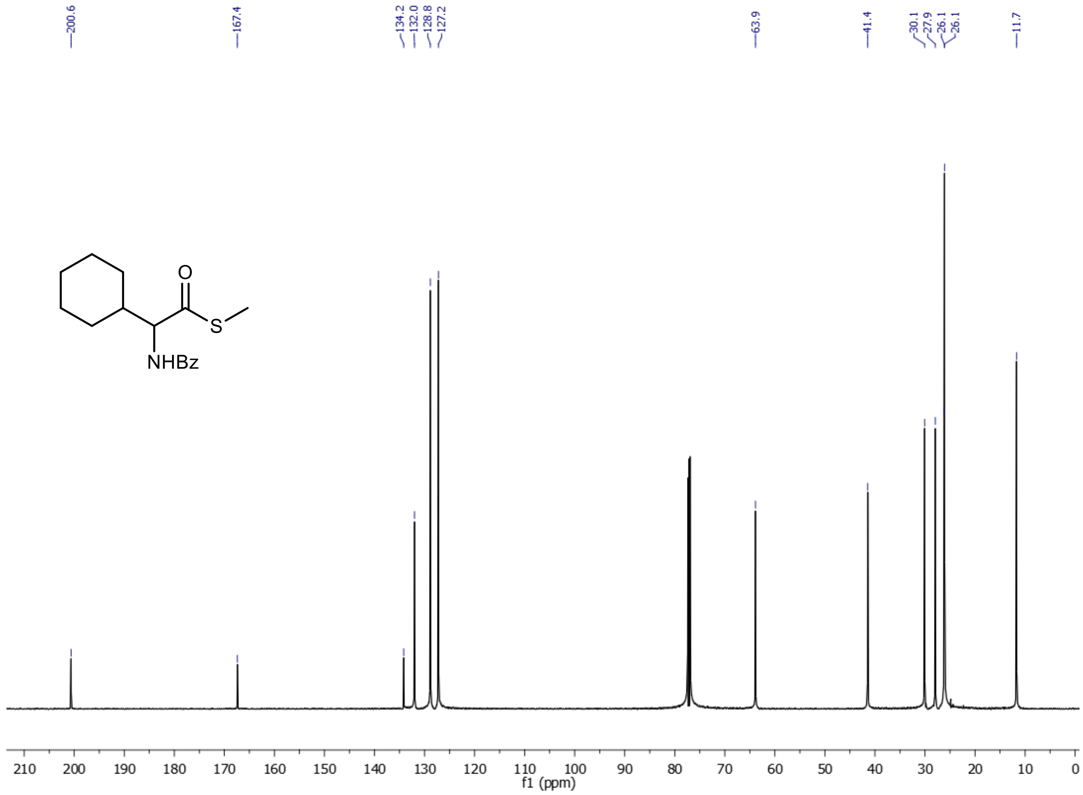

200.6
167.4
134.2
132.0
128.8
127.2
63.9
41.4
30.1
27.9
26.1
26.1
11.7
NHBz
O
S
f1 (ppm)

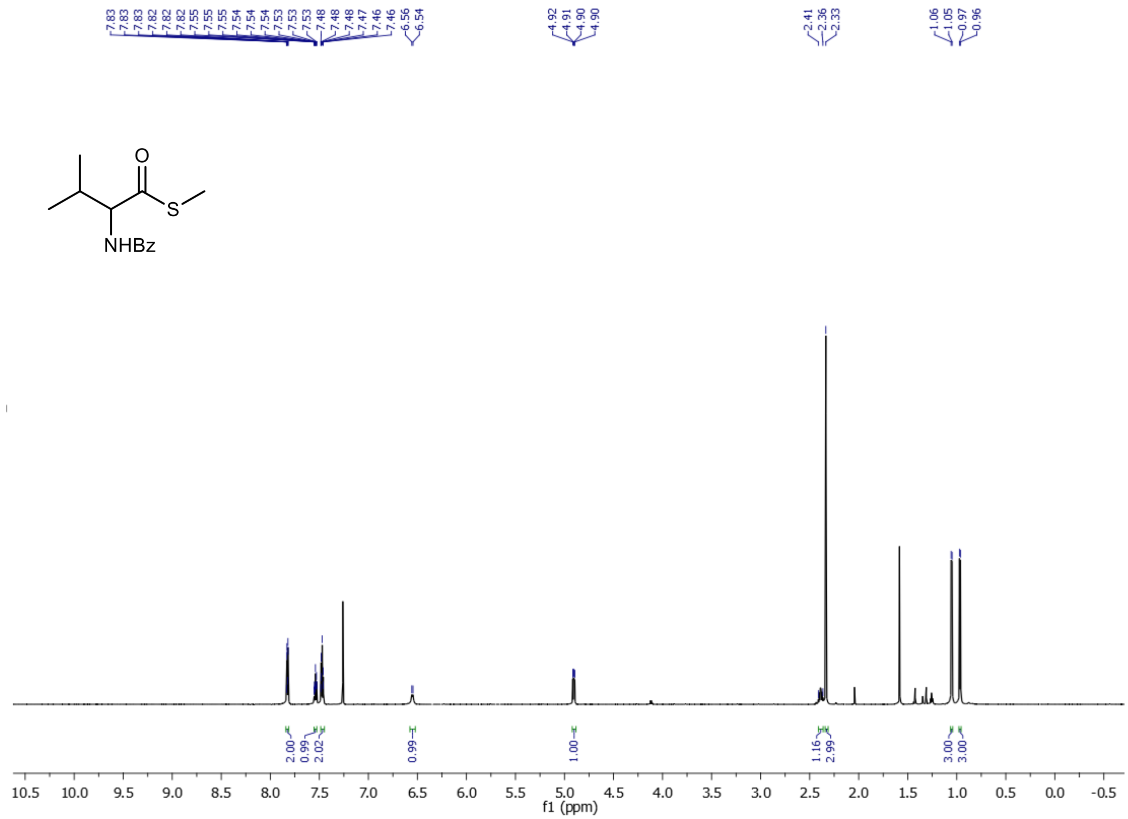

7.83
7.83
7.83
7.82
7.82
7.55
7.55
7.55
7.54
7.54
7.54
7.53
7.53
7.48
7.48
7.48
7.47
7.46
7.46
6.56
6.54
4.92
4.91
4.90
4.90
2.41
2.36
2.33
1.06
1.05
0.97
0.96
O
S
NHBz
2.00
0.99
2.02
0.99
1.00
1.16
2.99
3.00
3.00
10.5 10.0 9.5 9.0 8.5 8.0 7.5 7.0 6.5 6.0 5.5 5.0 4.5 4.0 3.5 3.0 2.5 2.0 1.5 1.0 0.5 0.0 -0.5
f1 (ppm)

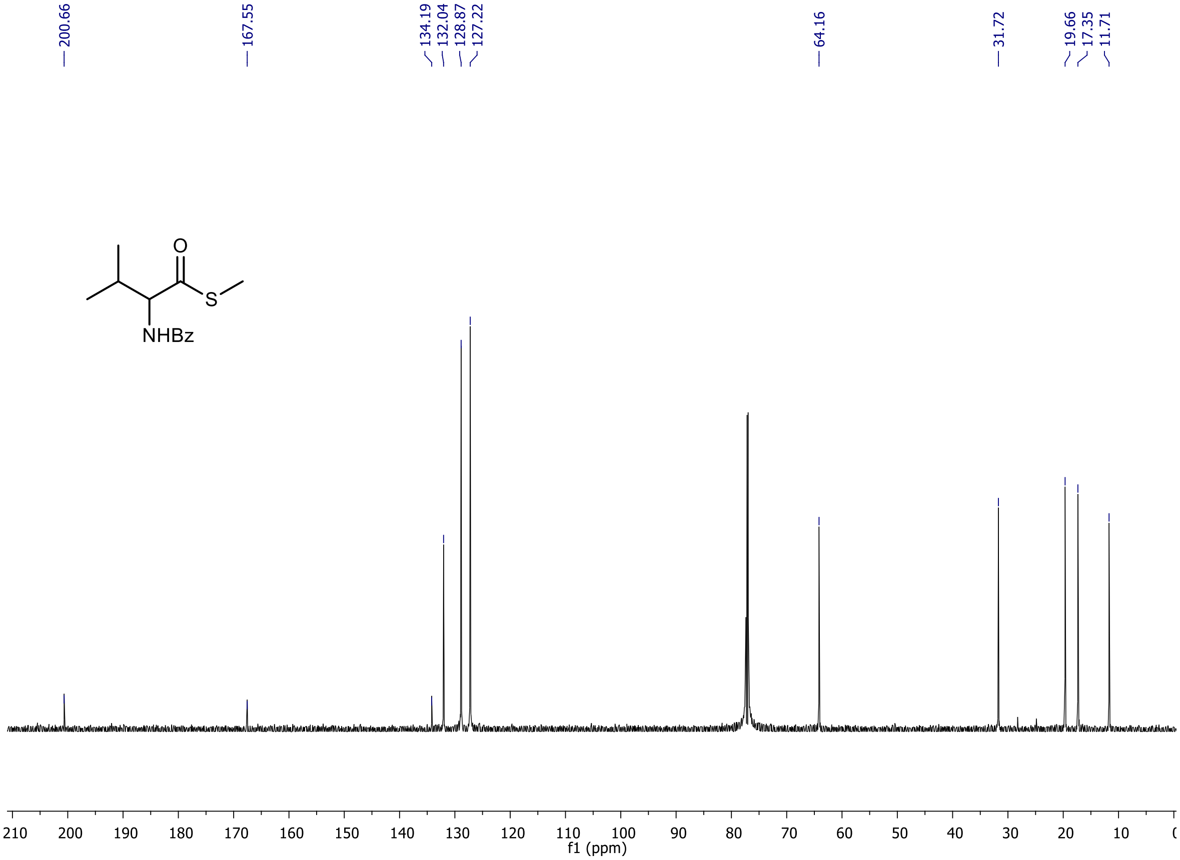

200.66
167.55
134.19
132.04
128.87
127.22
64.16
31.72
19.66
17.35
11.71
O
S
NHBz
210 200 190 180 170 160 150 140 130 120 110 100 90 80 70 60 50 40 30 20 10
f1 (ppm)

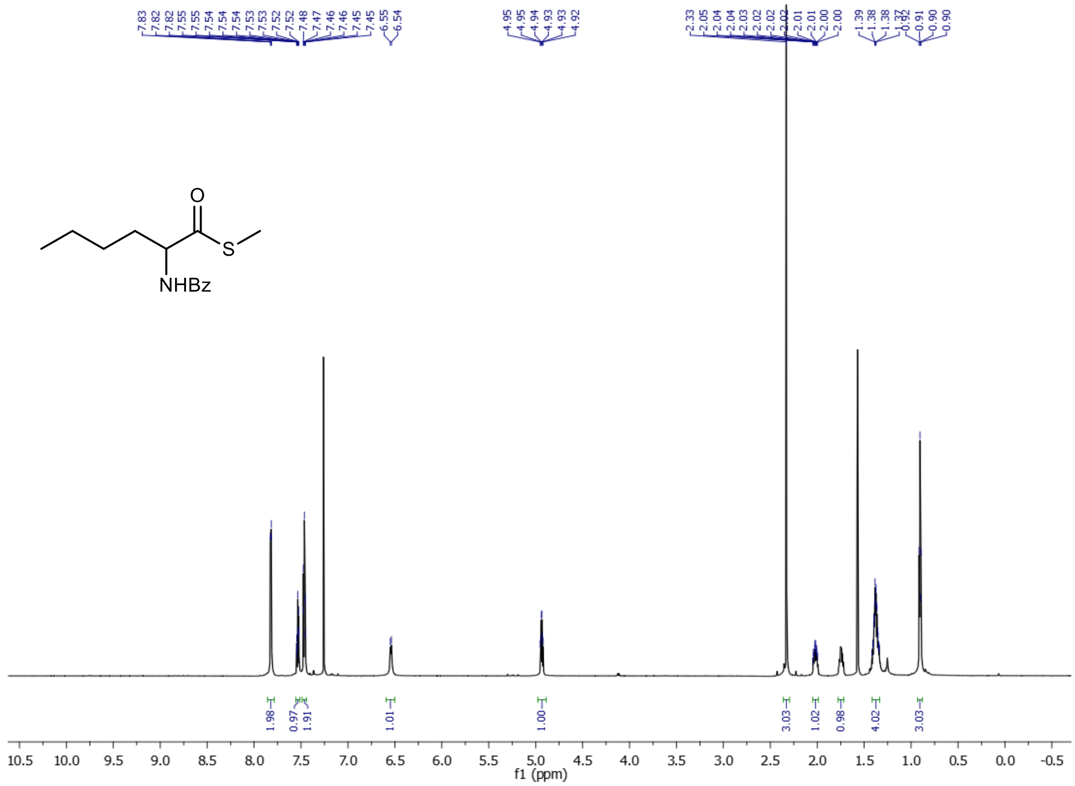
7.83
7.82
7.55
7.55
7.54
7.54
7.54
7.53
7.53
7.52
7.52
7.48
7.47
7.46
7.46
7.45
7.45
6.55
6.54
4.95
4.95
4.94
4.93
4.93
4.92
2.33
2.05
2.04
2.04
2.03
2.02
2.02
2.02
2.01
2.01
2.00
2.00
1.39
1.38
1.38
1.37
0.92
0.91
0.90
0.90
O
S
NHBz
1.98
0.97
1.91
1.01
1.00
3.03
1.02
0.98
4.02
3.03
10.5 10.0 9.5 9.0 8.5 8.0 7.5 7.0 6.5 6.0 5.5 5.0 4.5 4.0 3.5 3.0 2.5 2.0 1.5 1.0 0.5 0.0 -0.5
f1 (ppm)

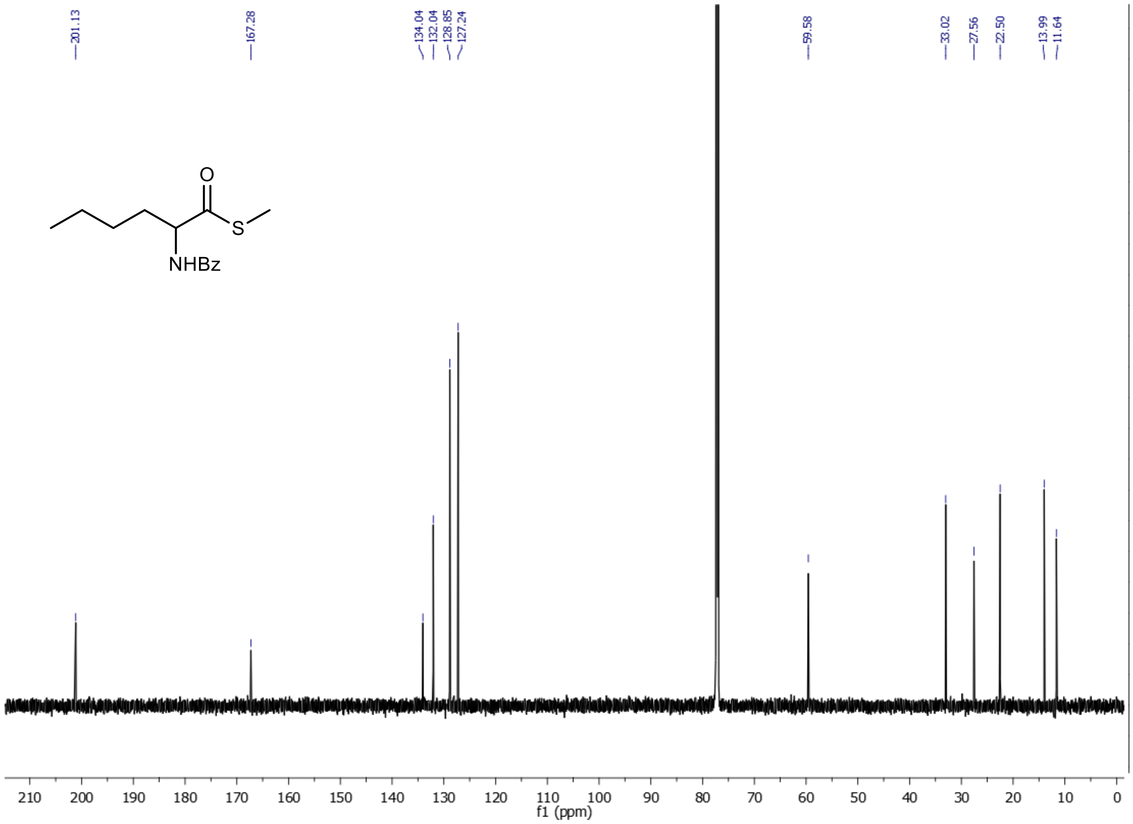
201.13
167.28
134.04
132.04
128.85
127.24
59.58
33.02
27.56
22.50
13.99
11.64
O
S
NHBz
210 200 190 180 170 160 150 140 130 120 110 100 90 80 70 60 50 40 30 20 10 0
f1 (ppm)

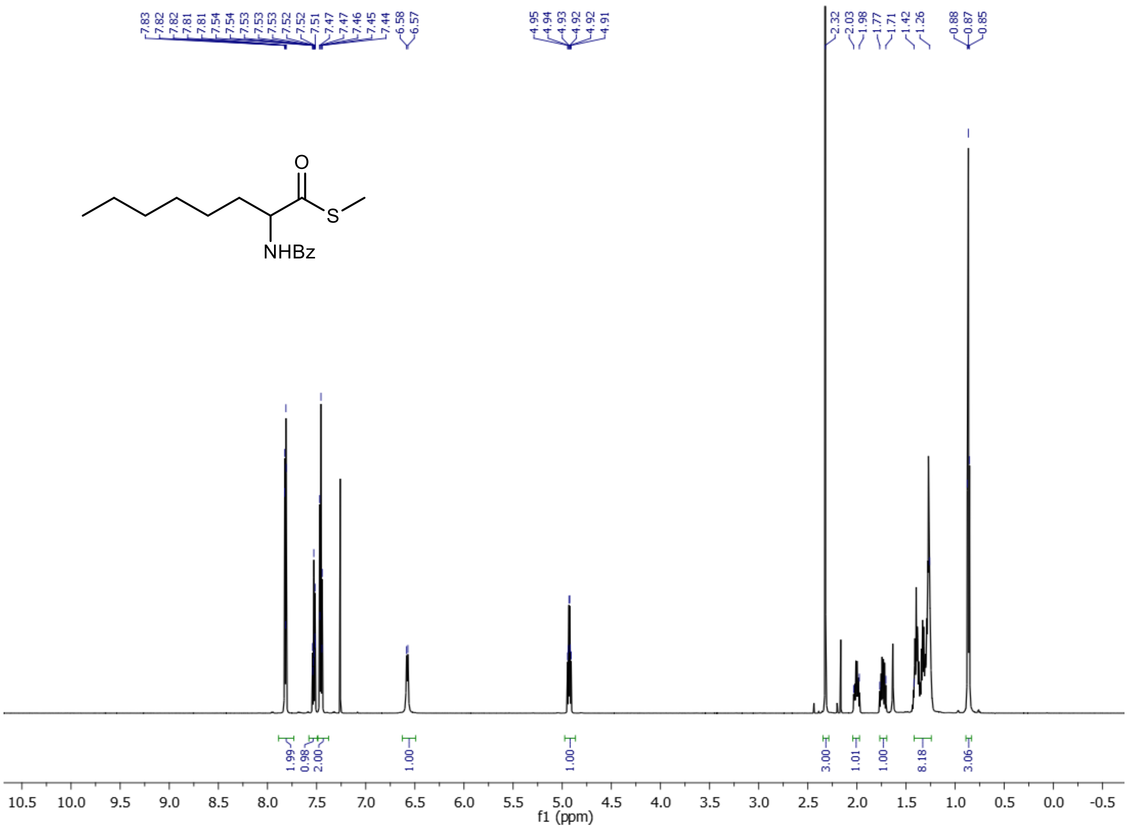
7.83
7.82
7.82
7.81
7.54
7.54
7.53
7.53
7.53
7.52
7.52
7.51
7.47
7.47
7.46
7.45
7.44
6.58
6.57
4.95
4.94
4.93
4.92
4.92
4.91
2.32
2.03
1.98
1.77
1.71
1.42
1.26
0.88
0.87
0.85
O
S
NHBz
1.99
0.98
2.00
1.00
1.00
3.00
1.01
1.00
8.18
3.06
10.5 10.0 9.5 9.0 8.5 8.0 7.5 7.0 6.5 6.0 5.5 5.0 4.5 4.0 3.5 3.0 2.5 2.0 1.5 1.0 0.5 0.0 -0.5
f1 (ppm)

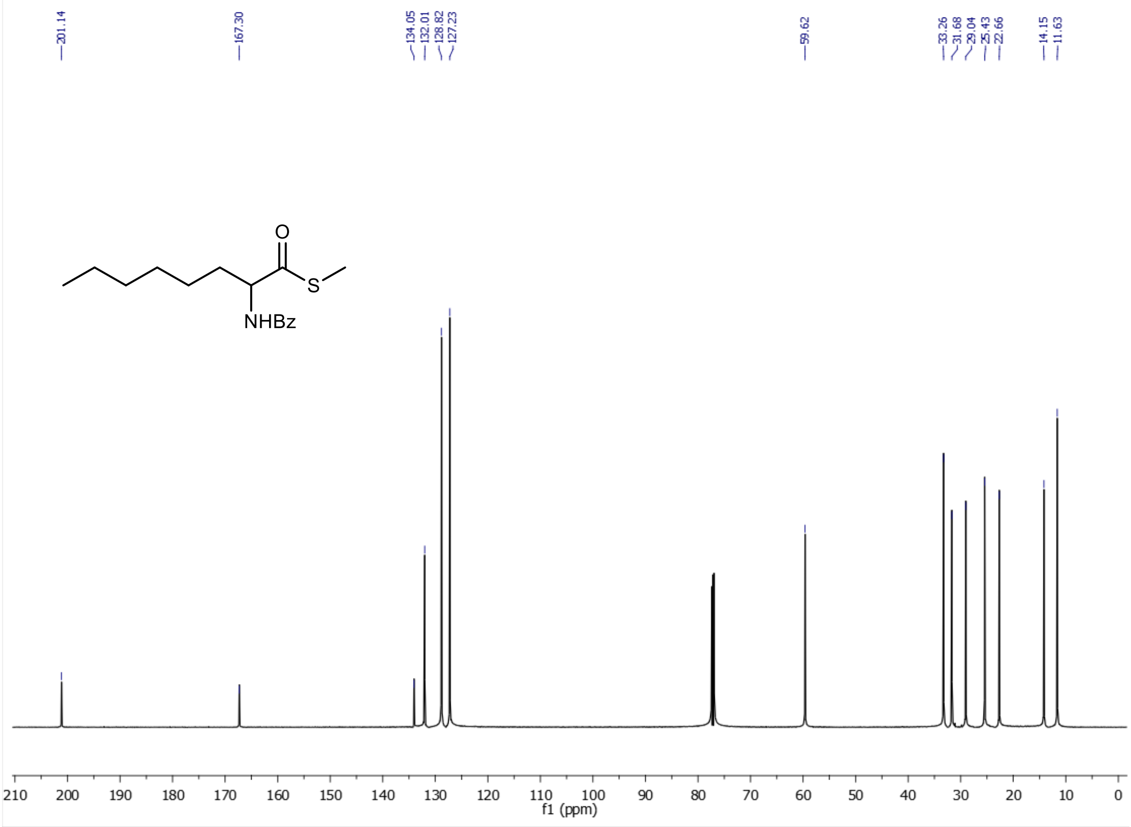
201.14
167.30
134.05
132.01
128.82
127.23
59.62
33.26
31.68
29.04
25.43
22.66
14.15
11.63
O
S
NHBz
210 200 190 180 170 160 150 140 130 120 110 100 90 80 70 60 50 40 30 20 10 0
f1 (ppm)

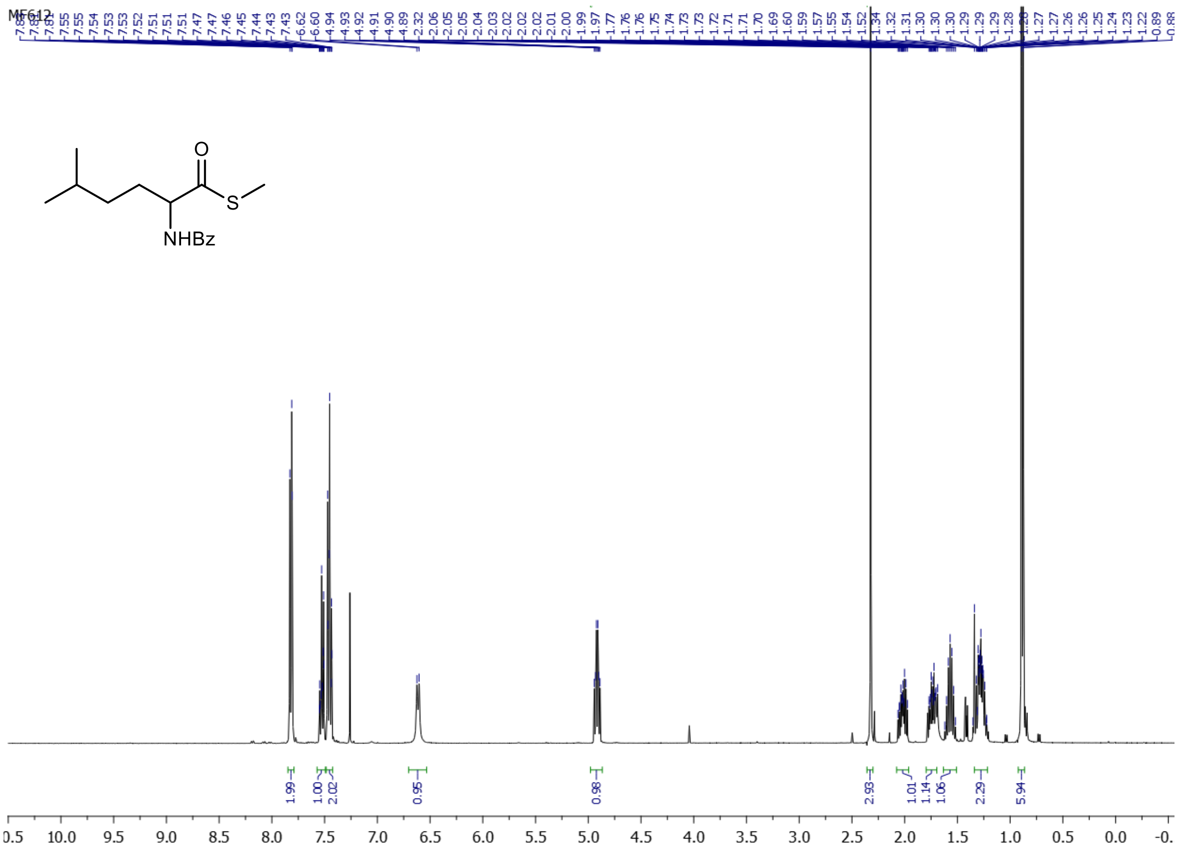
MF612
NHBz
S
O
2.93
1.01
1.14
1.06
2.29
5.94
1.99
1.00
2.02
0.95
0.98

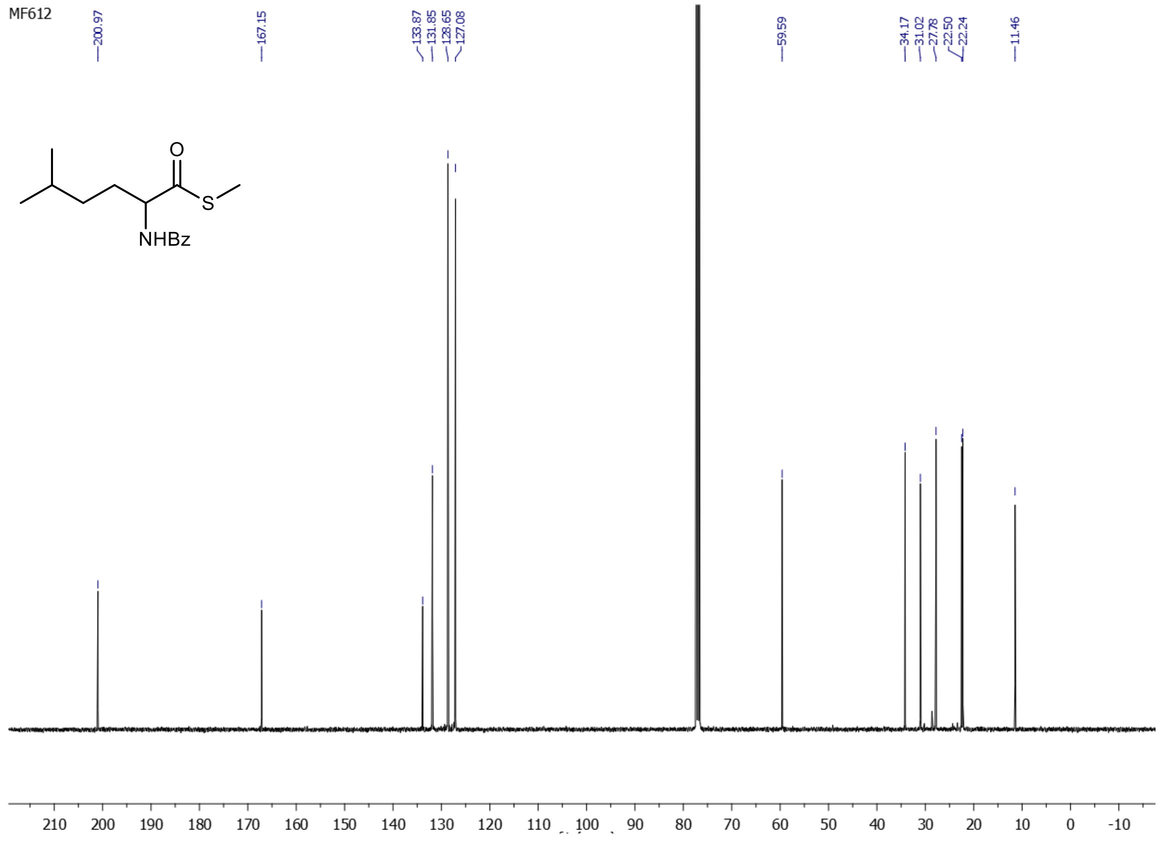
MF612
200.97
167.15
133.87
131.85
128.65
127.08
59.59
34.17
31.02
27.78
22.50
22.24
11.46
NHBz
S
O

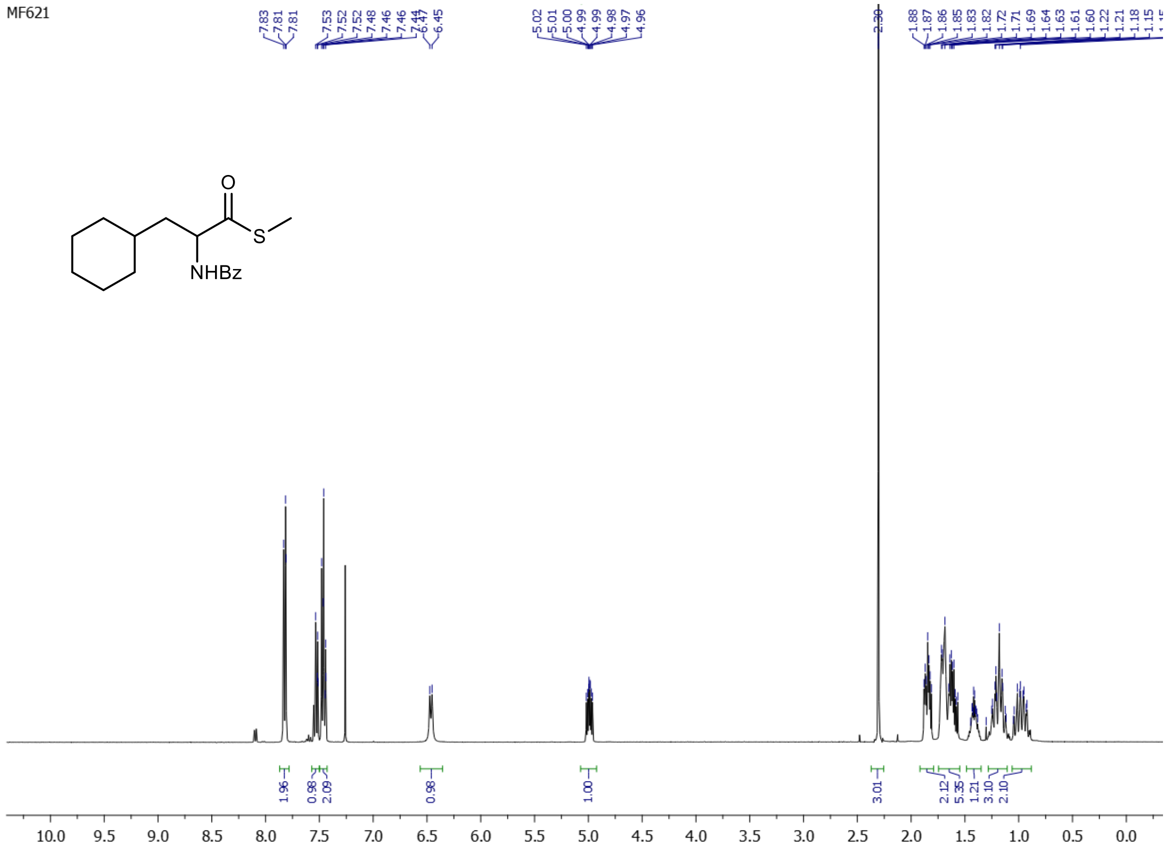
MF621
7.83 7.81 7.81 7.53 7.52 7.52 7.48 7.46 7.46 7.44 6.47 6.45
5.02 5.01 5.00 4.99 4.99 4.98 4.97 4.96
1.88 1.87 1.86 1.85 1.83 1.82 1.72 1.71 1.69 1.64 1.63 1.61 1.60 1.22 1.21 1.18 1.15
NHBz
O
S
1.96 0.98 2.09 0.98 1.00 3.01 2.12 5.35 1.21 3.10 2.10
10.0 9.5 9.0 8.5 8.0 7.5 7.0 6.5 6.0 5.5 5.0 4.5 4.0 3.5 3.0 2.5 2.0 1.5 1.0 0.5 0.0

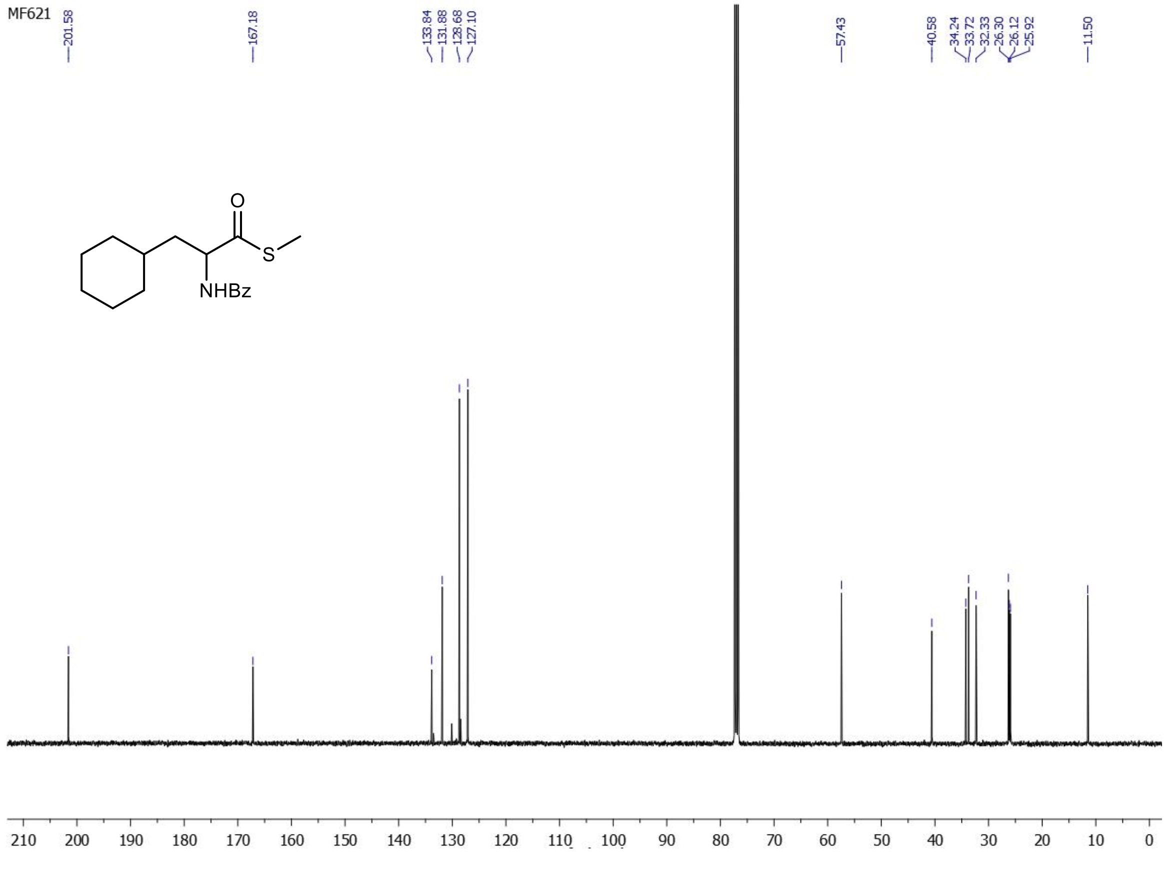
MF621
201.58
167.18
133.84 131.88 128.68 127.10
57.43
40.58 34.24 33.72 32.33 26.30 26.12 25.92
11.50
NHBz
O
S
210 200 190 180 170 160 150 140 130 120 110 100 90 80 70 60 50 40 30 20 10 0

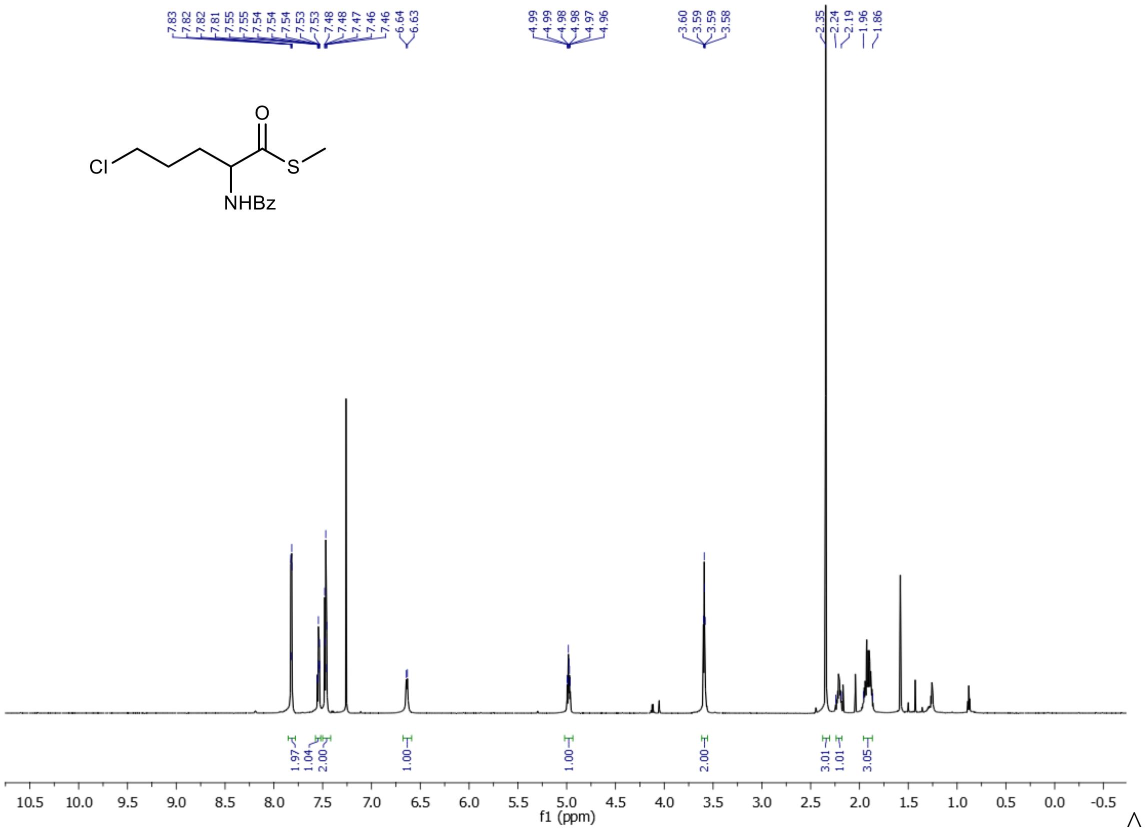

7.83
7.82
7.82
7.81
7.55
7.55
7.54
7.54
7.54
7.53
7.48
7.47
7.46
7.46
6.64
6.63
4.99
4.99
4.98
4.98
4.97
4.96
3.60
3.59
3.59
3.58
2.35
2.24
2.19
1.96
1.86
Cl
O
S
NHBz
1.97
1.04
2.00
1.00
1.00
2.00
3.01
1.01
3.05
f1 (ppm)

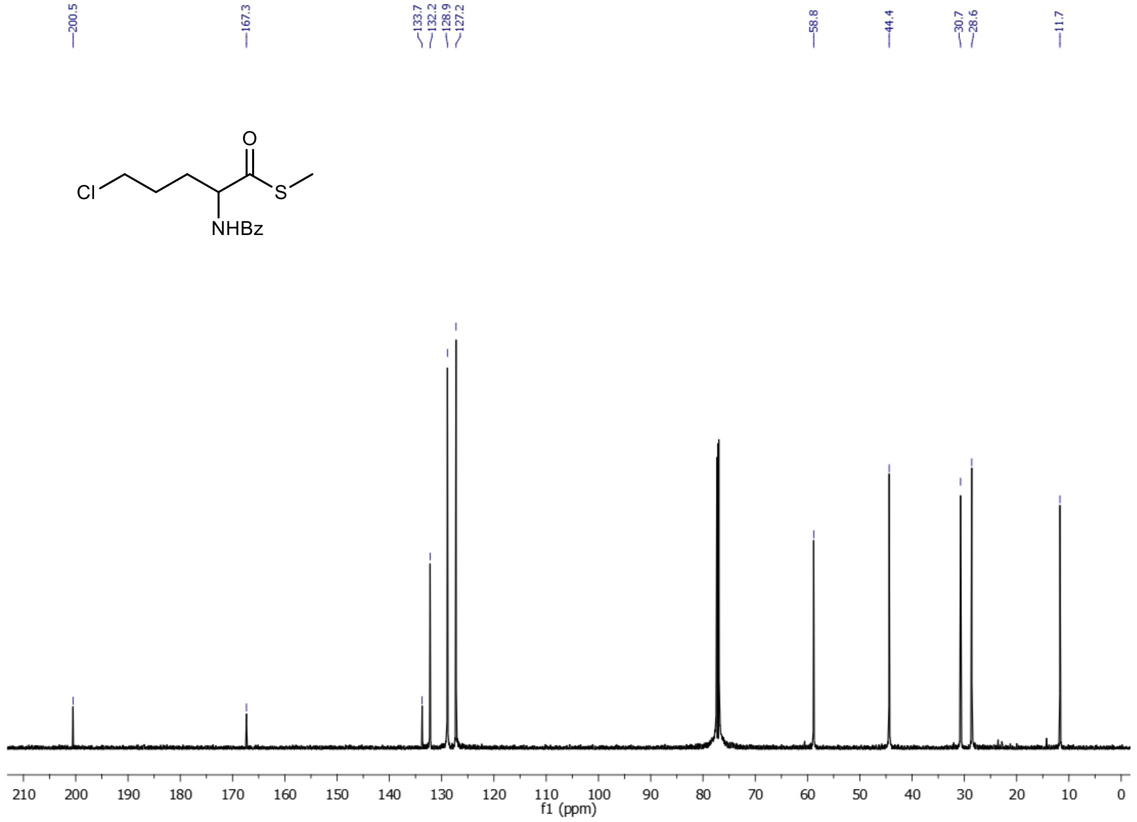

200.5
167.3
133.7
132.2
128.9
127.2
58.8
44.4
30.7
28.6
11.7
Cl
O
S
NHBz
f1 (ppm)

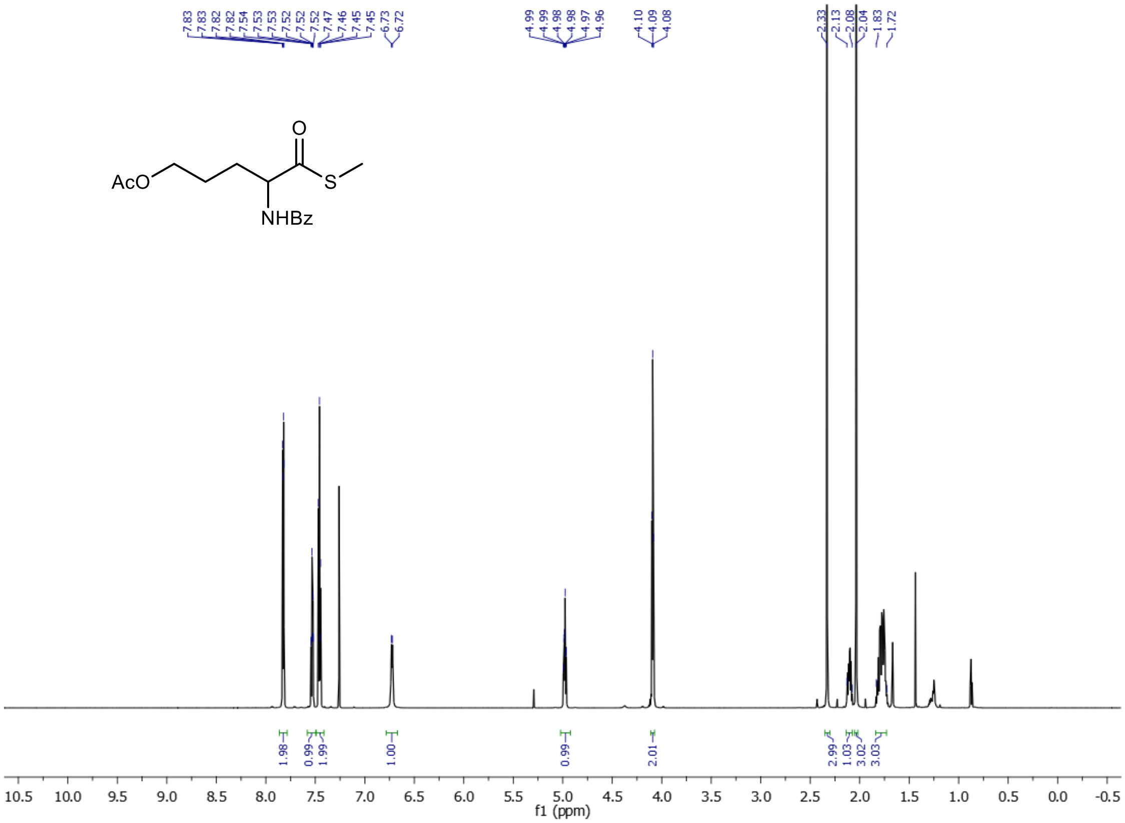
7.83 7.83 7.82 7.54 7.53 7.53 7.52 7.52 7.52 7.47 7.46 7.45 7.45 6.73 6.72
4.99 4.99 4.98 4.98 4.97 4.96
4.10 4.09 4.08
2.33 2.13 2.08 2.04 1.83 1.72
O
AcO
S
NHBz
1.98 0.99 1.99 1.00 0.99 2.01 2.99 1.03 3.02 3.03
10.5 10.0 9.5 9.0 8.5 8.0 7.5 7.0 6.5 6.0 5.5 5.0 4.5 4.0 3.5 3.0 2.5 2.0 1.5 1.0 0.5 0.0 -0.5
f1 (ppm)

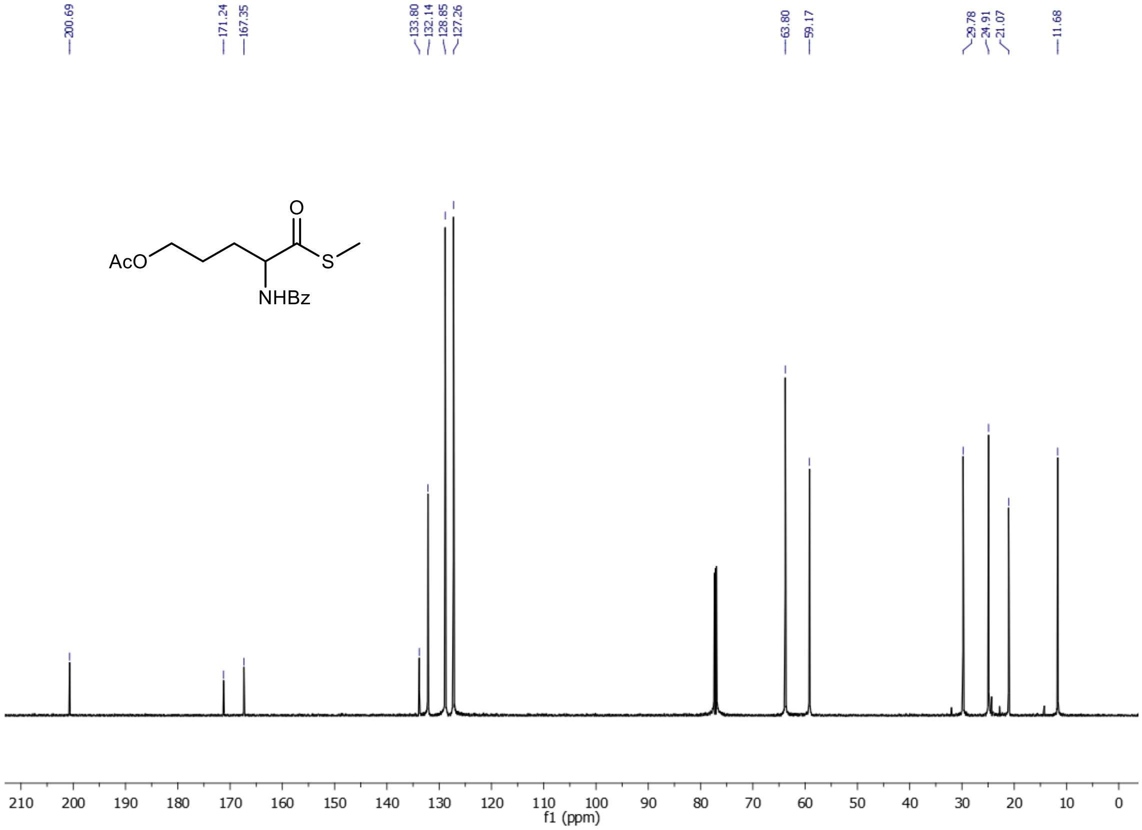
200.69
171.24 167.35
133.80 132.14 128.85 127.26
63.80 59.17
29.78 24.91 21.07
11.68
O
AcO
S
NHBz
210 200 190 180 170 160 150 140 130 120 110 100 90 80 70 60 50 40 30 20 10 0
f1 (ppm)

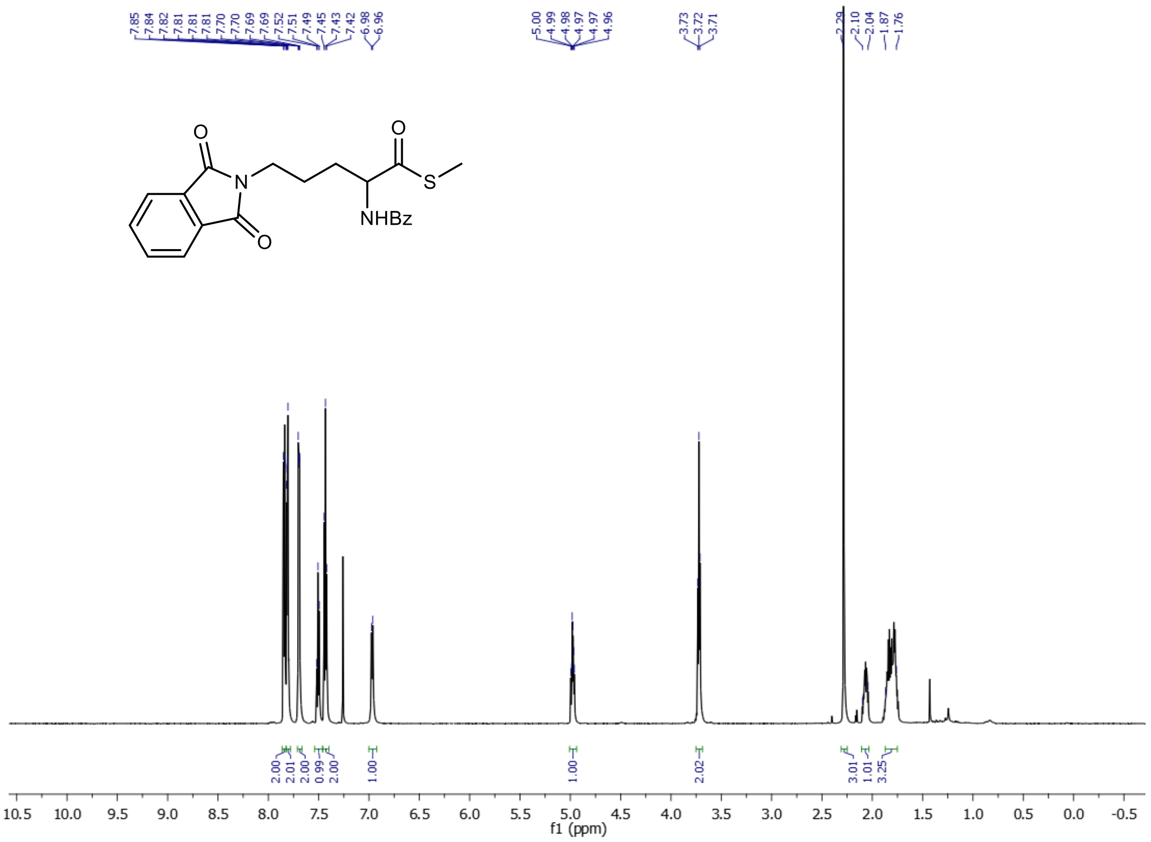
7.85
7.84
7.82
7.81
7.81
7.81
7.70
7.70
7.69
7.69
7.52
7.51
7.49
7.45
7.43
7.42
6.98
6.96
5.00
4.99
4.98
4.97
4.97
4.96
3.73
3.72
3.71
2.29
2.10
2.04
1.87
1.76
O
N
O
O
S
NHBz
2.00
2.01
2.00
0.99
2.00
1.00
1.00
2.02
3.01
1.01
3.25
10.5 10.0 9.5 9.0 8.5 8.0 7.5 7.0 6.5 6.0 5.5 5.0 4.5 4.0 3.5 3.0 2.5 2.0 1.5 1.0 0.5 0.0 -0.5
f1 (ppm)

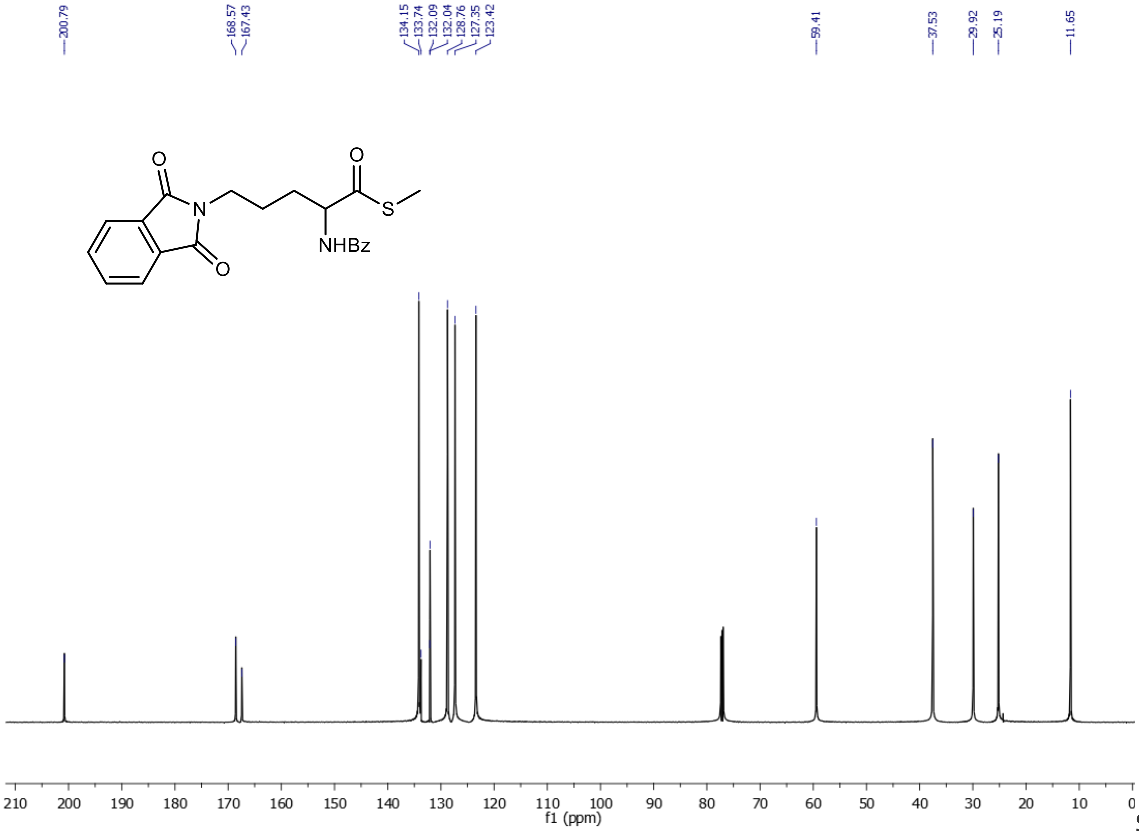
200.79
168.57
167.43
134.15
133.74
132.09
132.04
128.76
127.35
123.42
59.41
37.53
29.92
25.19
11.65
O
N
O
O
S
NHBz
210 200 190 180 170 160 150 140 130 120 110 100 90 80 70 60 50 40 30 20 10 0
f1 (ppm)

S

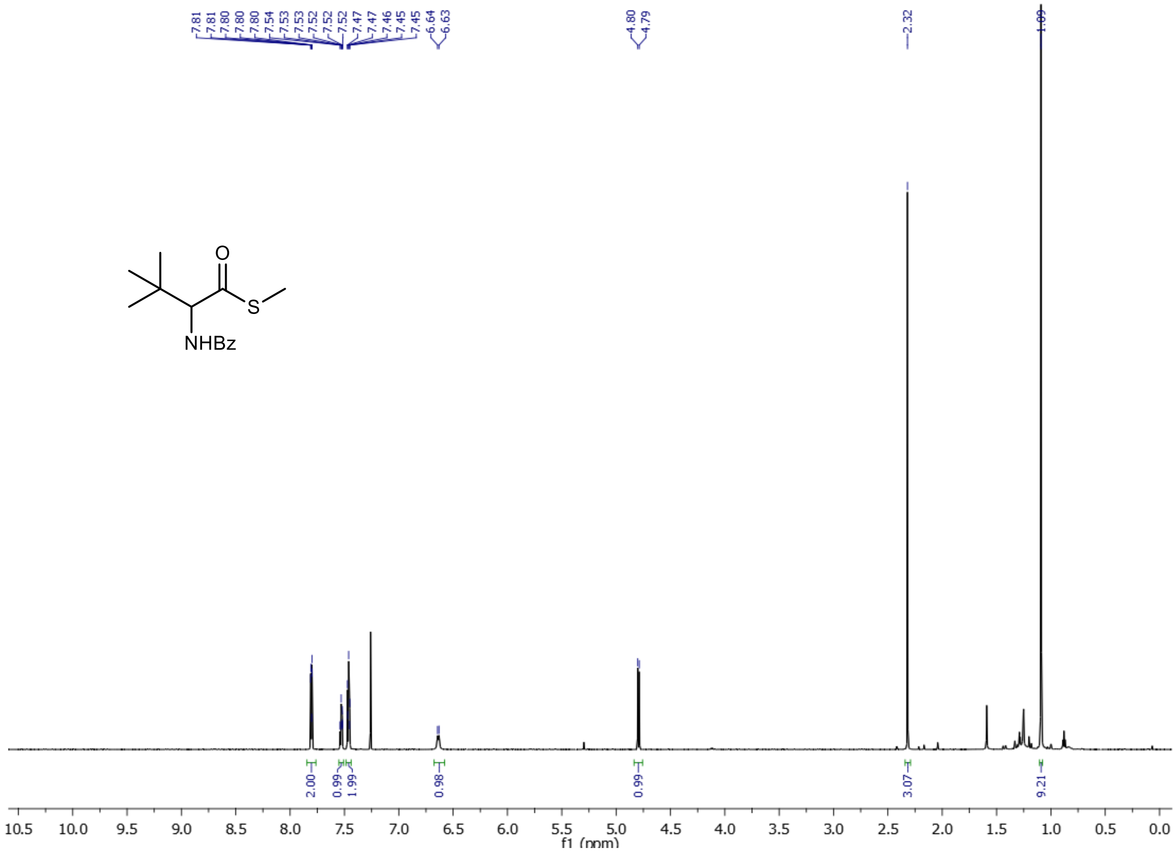
NHBz
O
S
f1 (ppm)

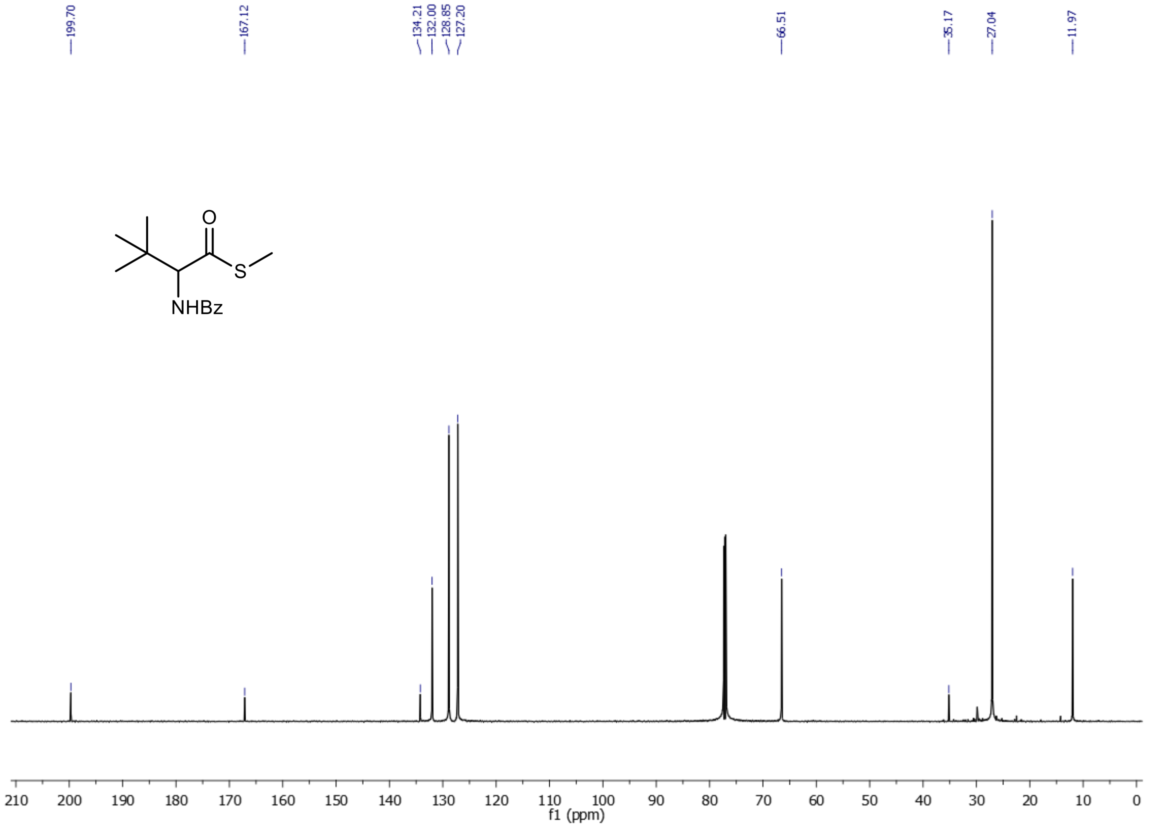
NHBz
O
S
f1 (ppm)

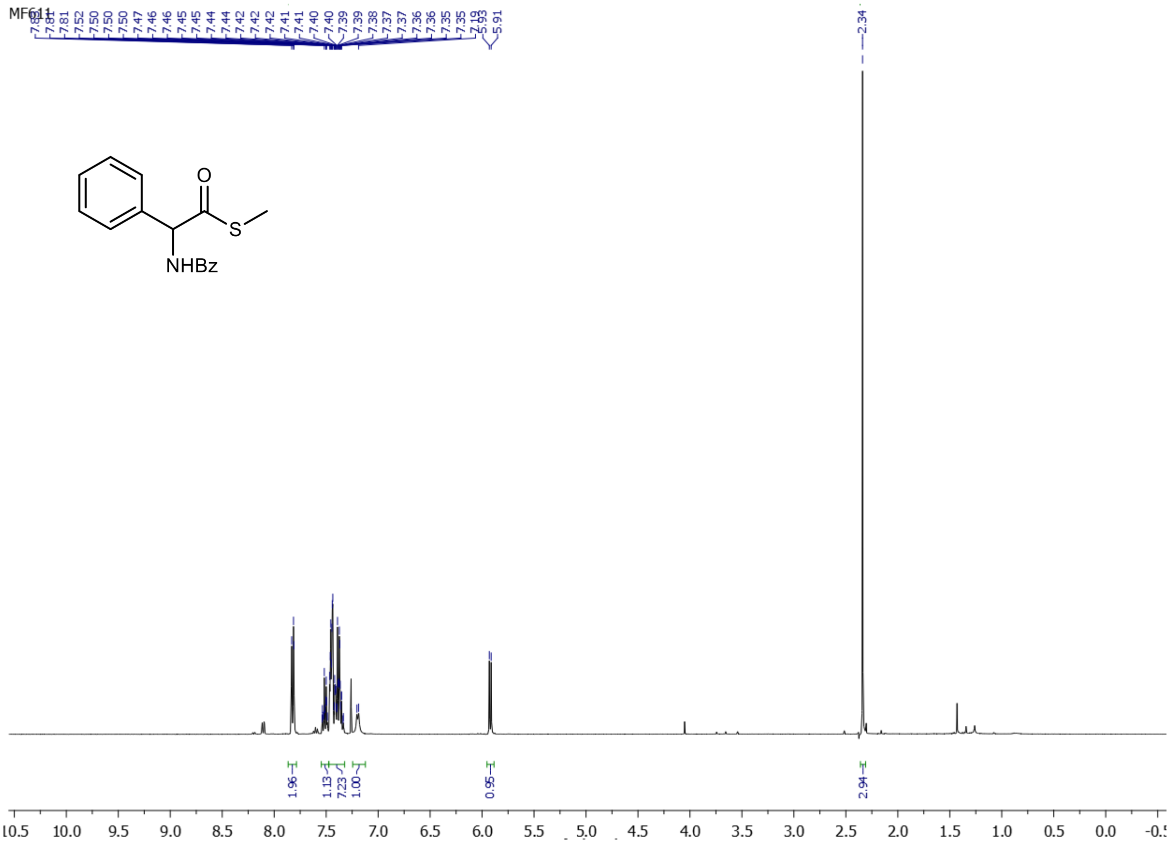
MF611
7.81
7.52
7.50
7.50
7.50
7.47
7.46
7.46
7.45
7.45
7.44
7.42
7.42
7.42
7.41
7.41
7.40
7.40
7.39
7.39
7.38
7.37
7.37
7.36
7.36
7.35
7.35
7.19
5.93
5.91
2.34
O
S
NHBz
1.96
1.13
7.23
1.00
0.95
2.94
10.5 10.0 9.5 9.0 8.5 8.0 7.5 7.0 6.5 6.0 5.5 5.0 4.5 4.0 3.5 3.0 2.5 2.0 1.5 1.0 0.5 0.0

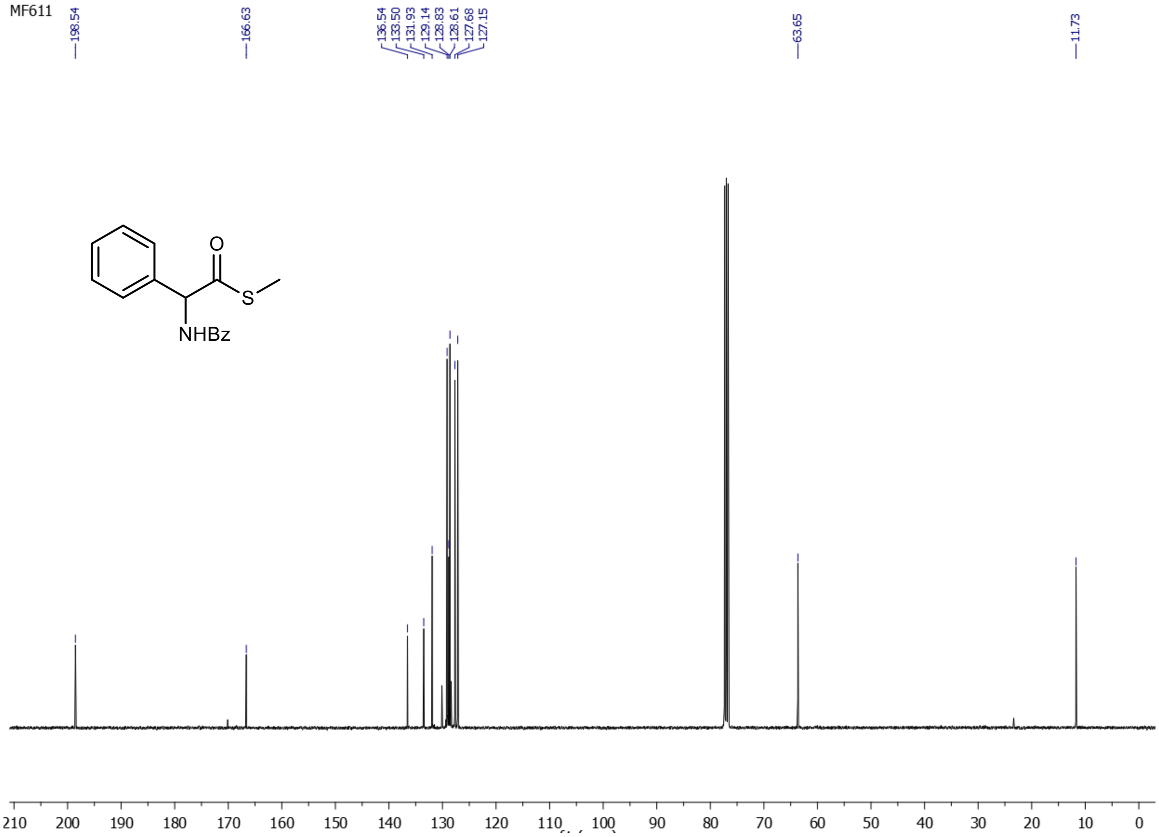
MF611
198.54
166.63
136.54
133.50
131.93
129.14
128.83
128.61
127.68
127.15
63.65
11.73
O
S
NHBz
210 200 190 180 170 160 150 140 130 120 110 100 90 80 70 60 50 40 30 20 10 0

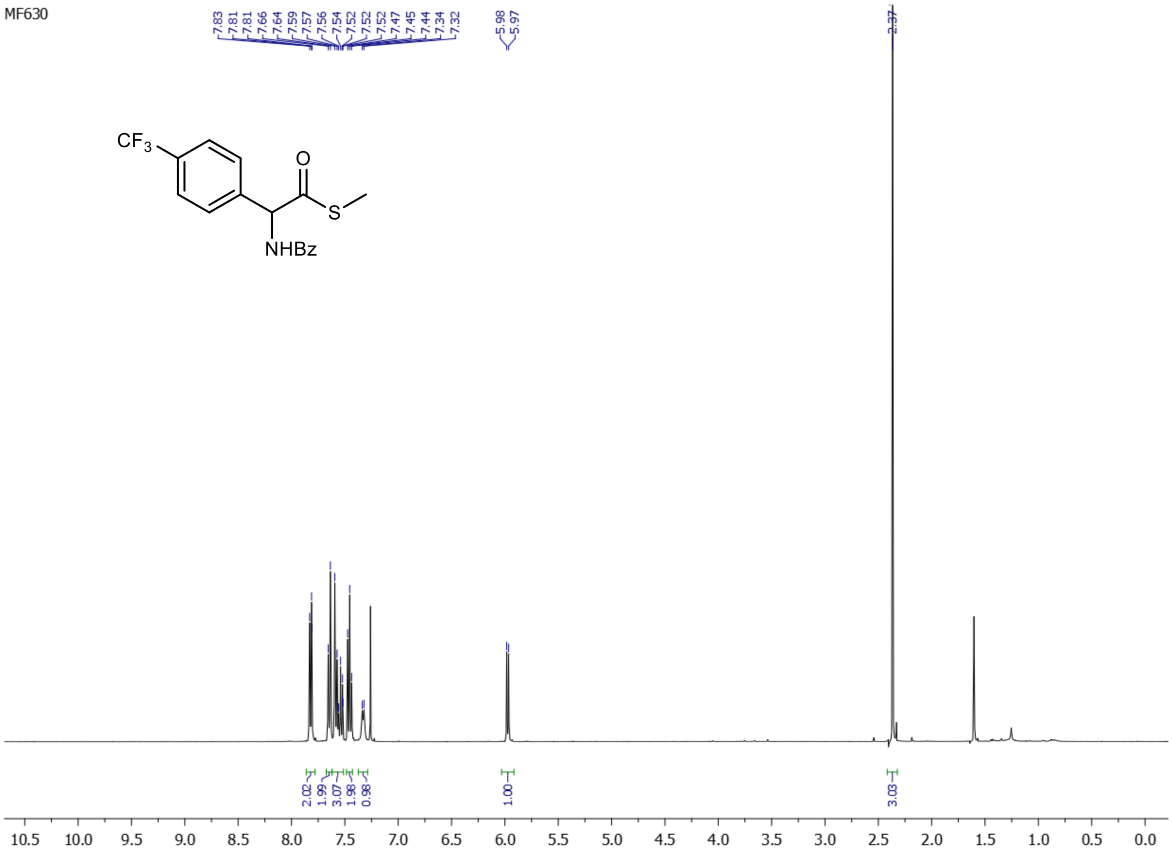
MF630
7.83
7.81
7.81
7.66
7.64
7.59
7.57
7.56
7.54
7.52
7.52
7.52
7.47
7.45
7.44
7.34
7.32
5.98
5.97
2.37
CF3
O
S
NHBz
2.02
1.99
3.07
1.98
0.98
1.00
3.03
10.5 10.0 9.5 9.0 8.5 8.0 7.5 7.0 6.5 6.0 5.5 5.0 4.5 4.0 3.5 3.0 2.5 2.0 1.5 1.0 0.5 0.0

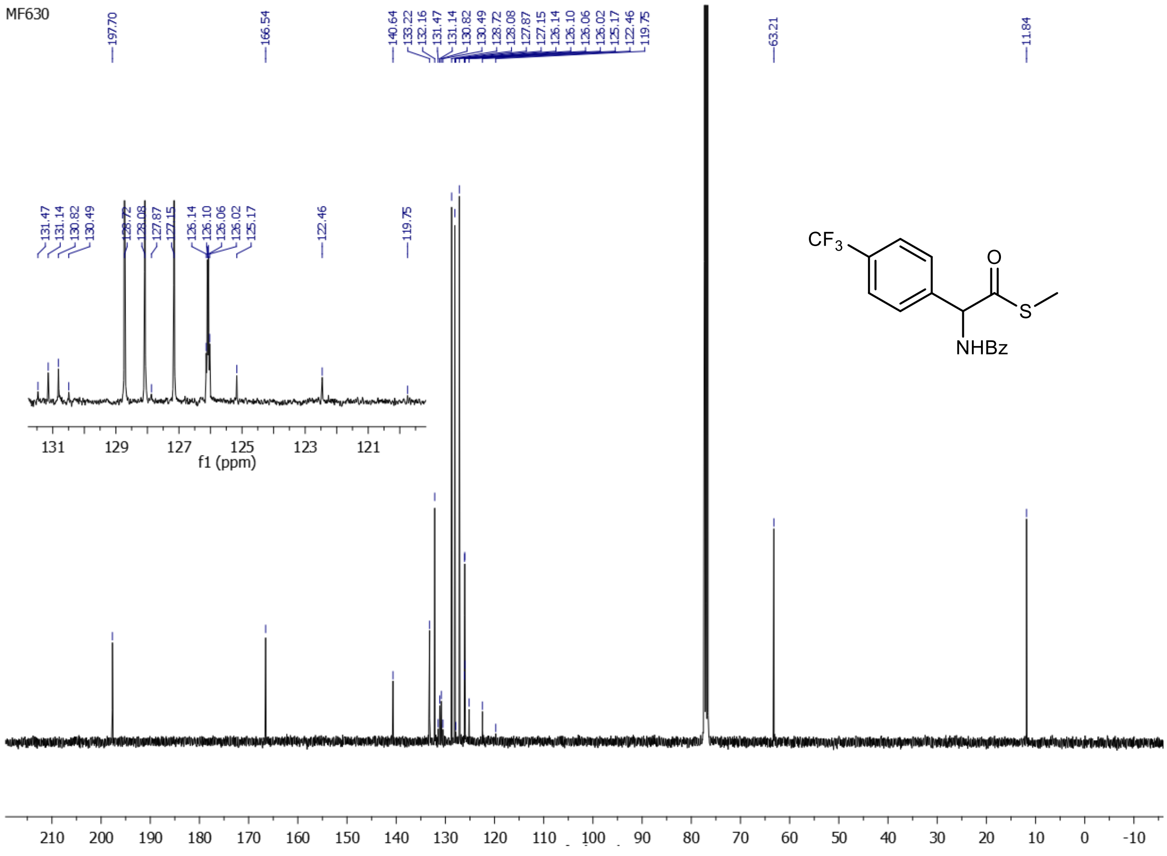
MF630
197.70
166.54
140.64
133.22
132.16
131.47
131.14
130.82
130.49
128.72
128.08
127.87
127.15
126.14
126.10
126.06
126.02
125.17
122.46
119.75
63.21
11.84
131 129 127 125 123 121
f1 (ppm)
CF3
O
S
NHBz
210 200 190 180 170 160 150 140 130 120 110 100 90 80 70 60 50 40 30 20 10 0 -10

MF630

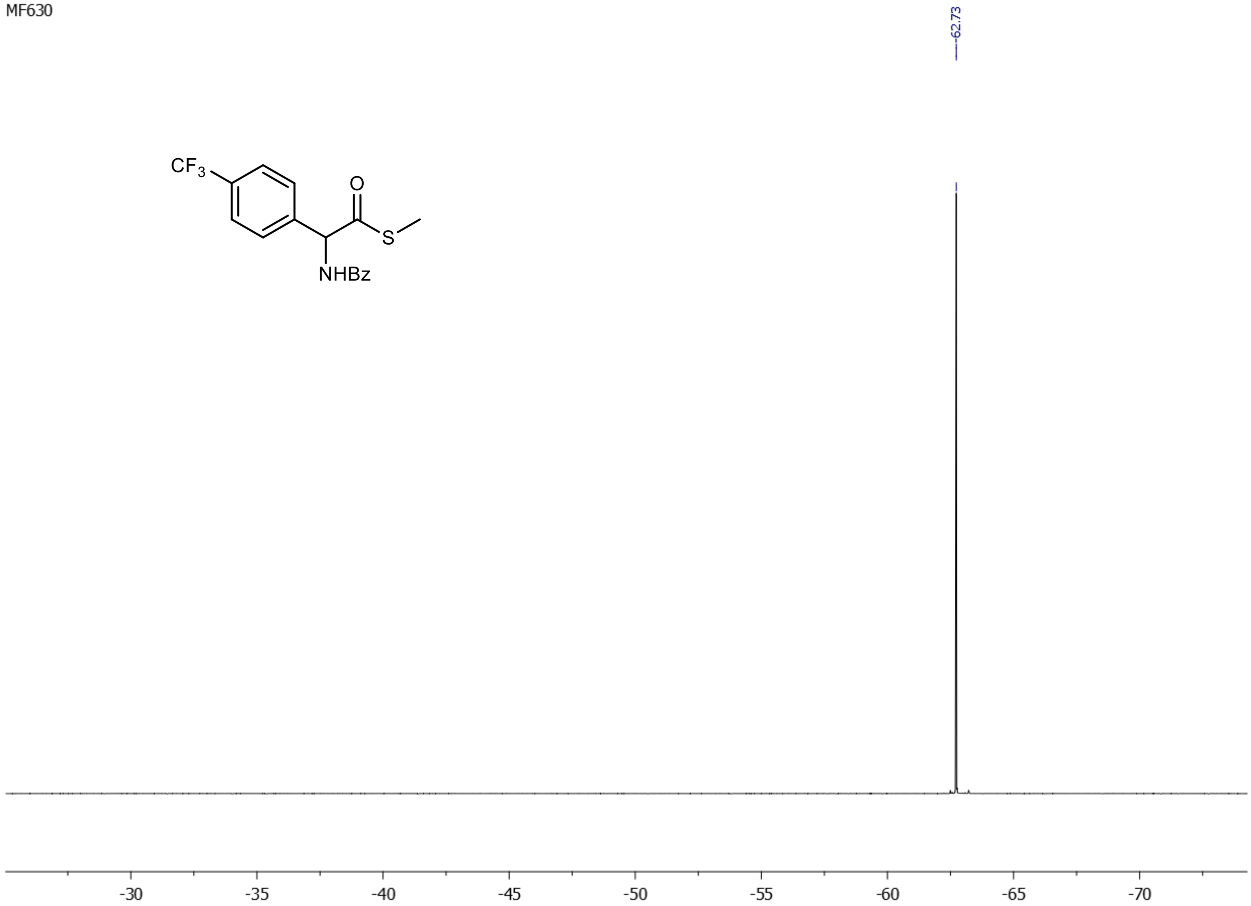

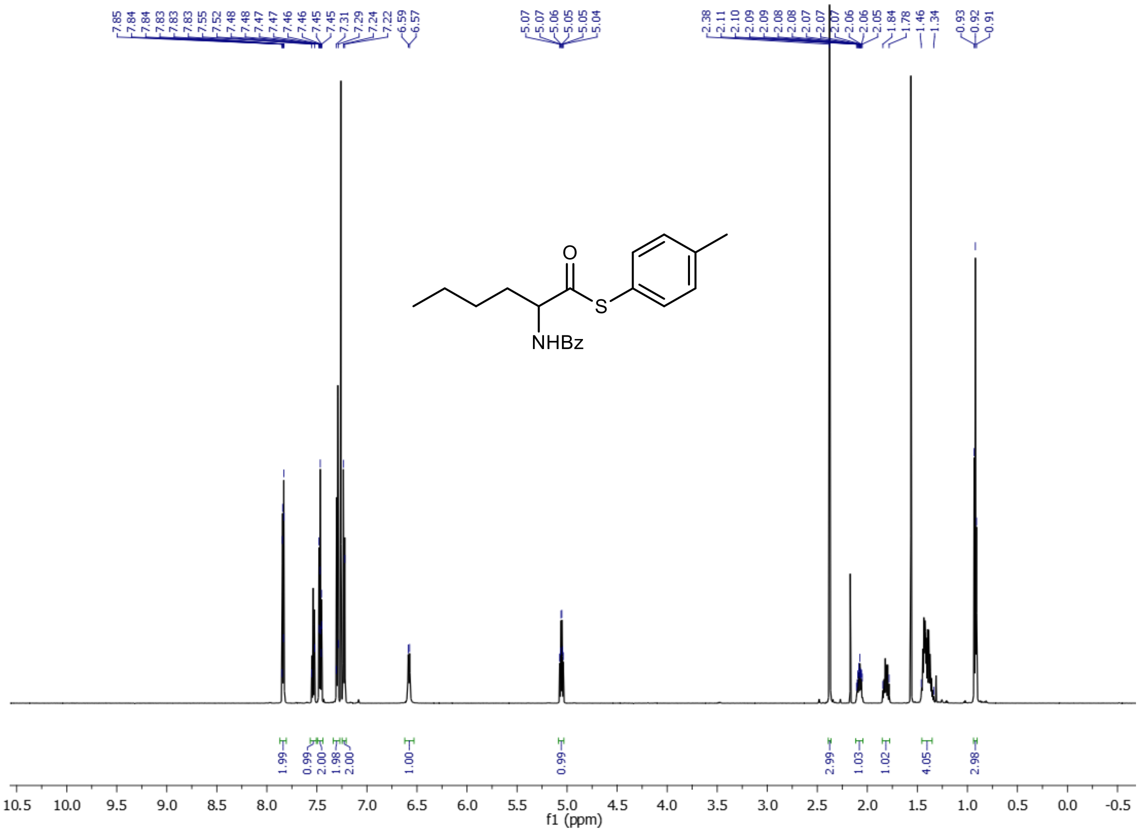
NHBz
f1 (ppm)

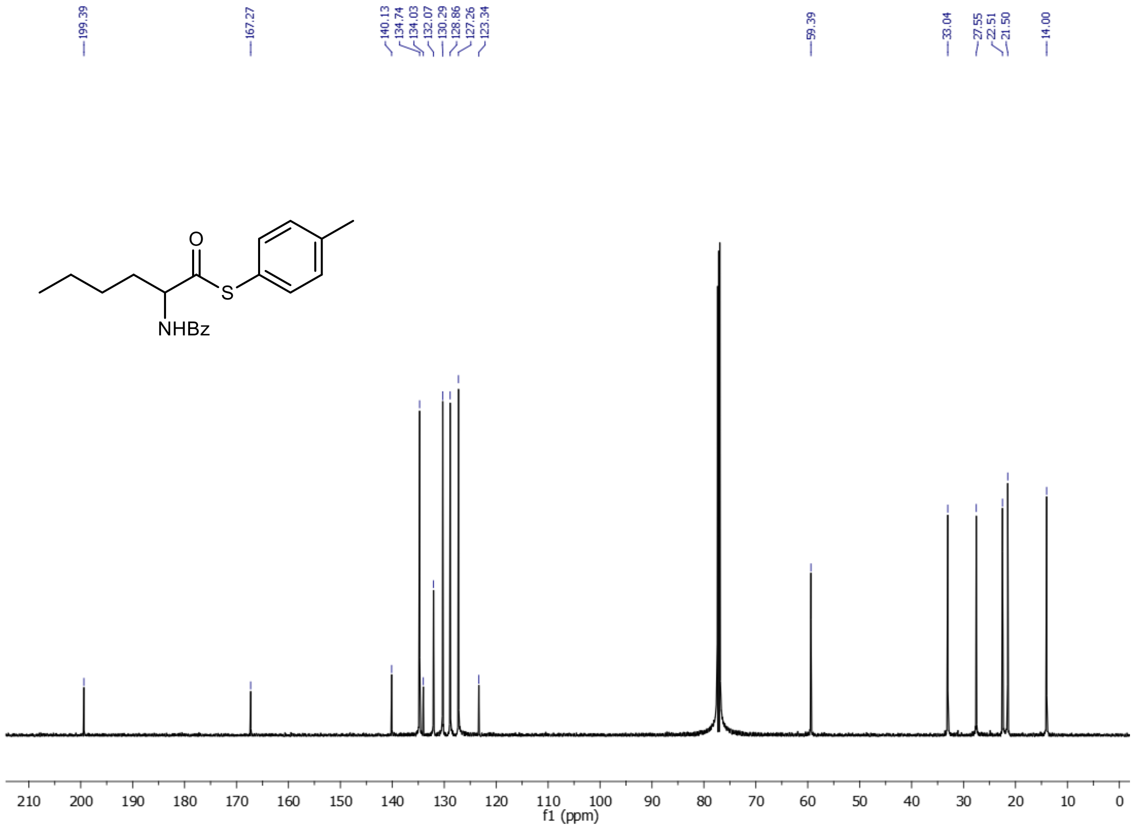
NHBz
f1 (ppm)

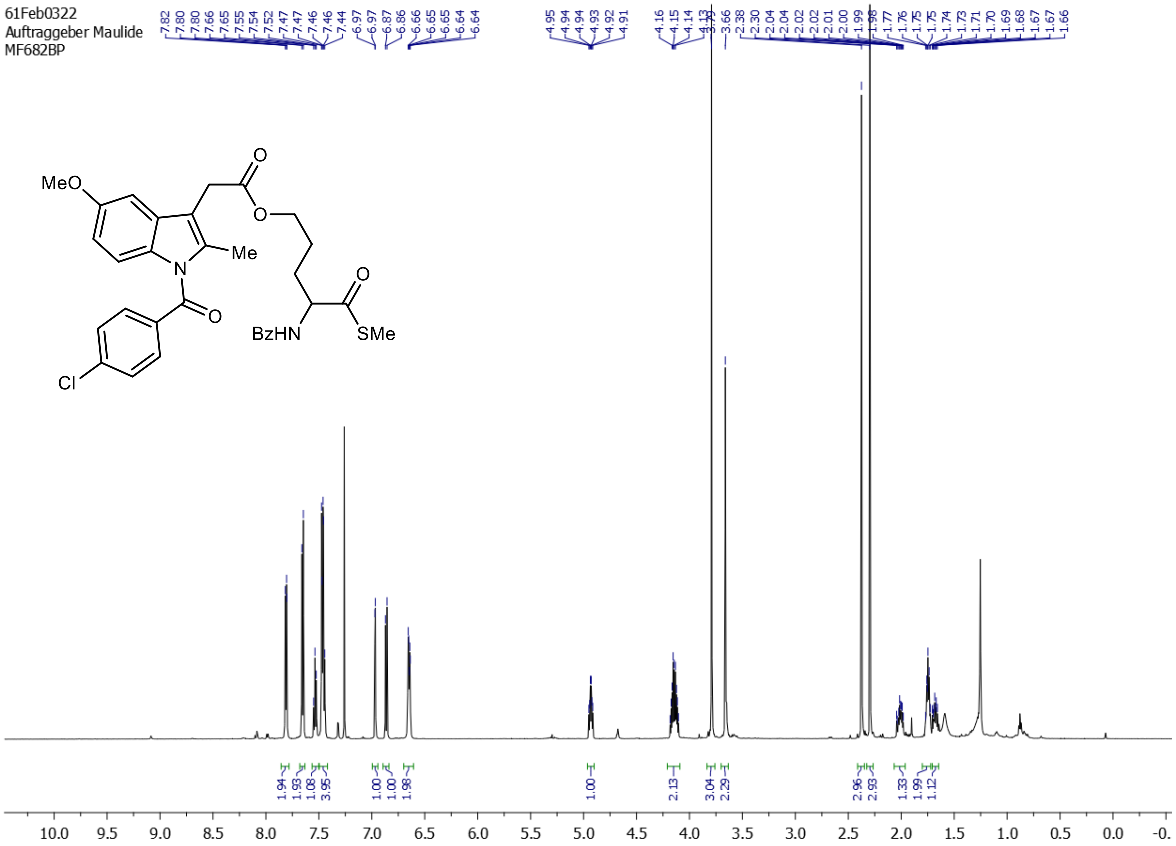
61Feb0322
Auftraggeber Maulide
MF682BP
MeO
Me
N
O
Cl
BzHN
SMe
7.82 7.80 7.80 7.66 7.65 7.55 7.54 7.52 7.47 7.47 7.46 7.46 7.44 6.97 6.97 6.87 6.86 6.66 6.65 6.65 6.64 6.64
4.95 4.94 4.94 4.93 4.92 4.91 4.16 4.15 4.14 4.13 3.79 3.66 2.38 2.30 2.04 2.04 2.02 2.02 2.01 2.00 1.99 1.98 1.77 1.76 1.75 1.75 1.74 1.73 1.71 1.70 1.69 1.68 1.67 1.66
1.94 1.93 1.08 3.95 1.00 1.00 1.98 1.00 2.13 3.04 2.29 2.96 2.93 1.33 1.99 1.12
10.0 9.5 9.0 8.5 8.0 7.5 7.0 6.5 6.0 5.5 5.0 4.5 4.0 3.5 3.0 2.5 2.0 1.5 1.0 0.5 0.0

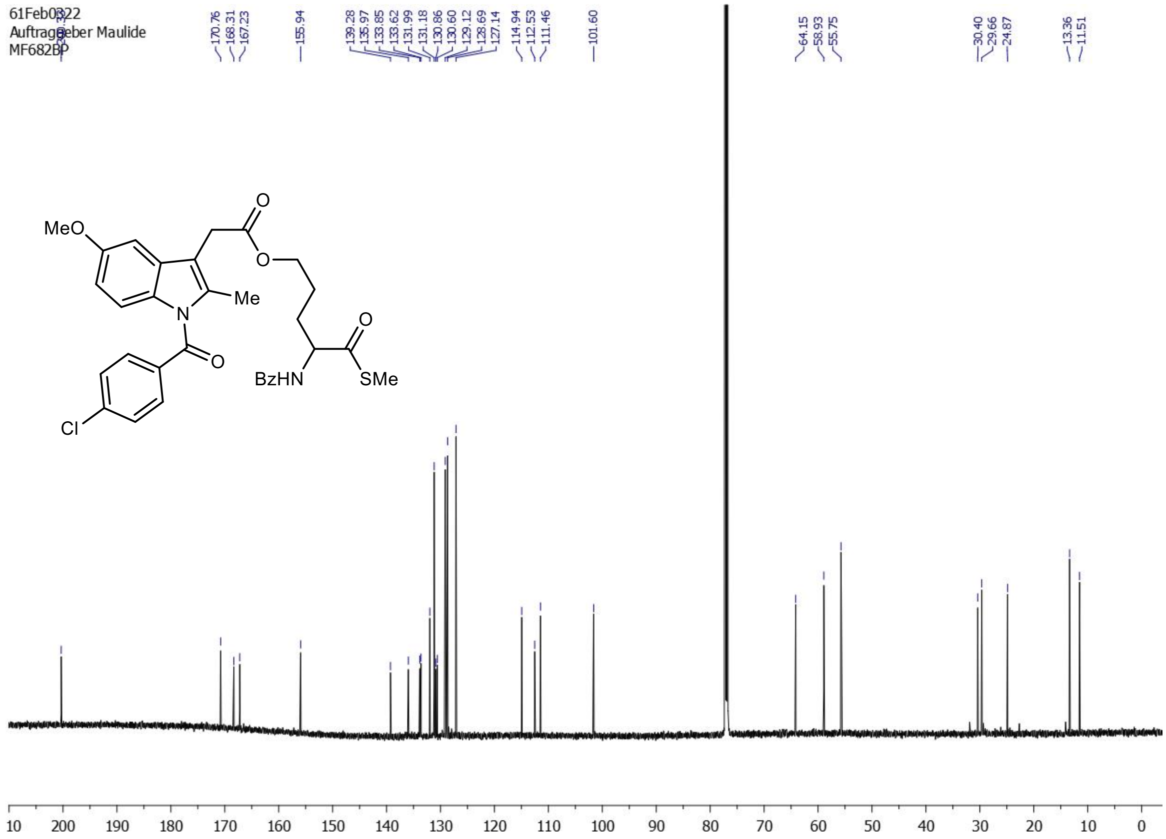
61Feb0322
Auftraggeber Maulide
MF682BP
170.76 168.31 167.23 155.94 139.28 135.97 133.85 133.62 131.99 131.18 130.86 130.60 129.12 128.69 127.14 114.94 112.53 111.46 101.60
64.15 58.93 55.75 30.40 29.66 24.87 13.36 11.51
MeO
Me
N
O
Cl
BzHN
SMe
200 190 180 170 160 150 140 130 120 110 100 90 80 70 60 50 40 30 20 10 0

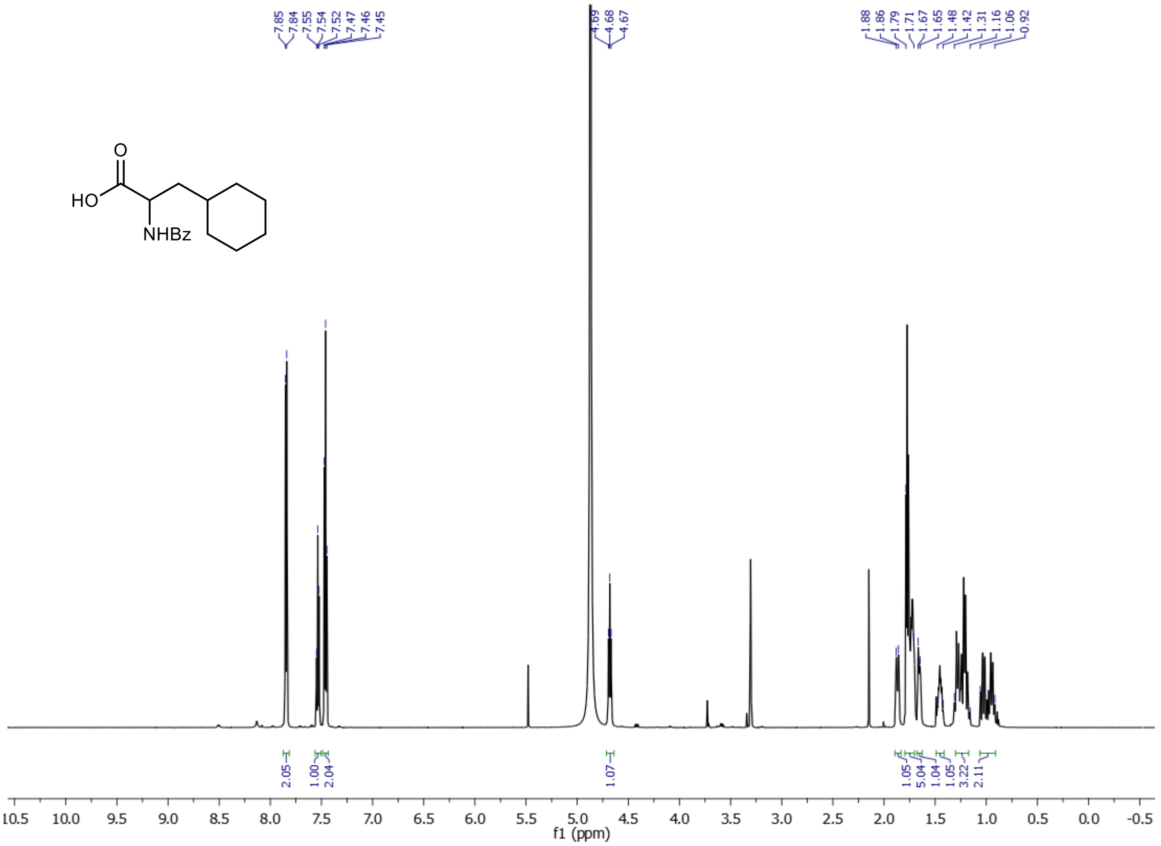

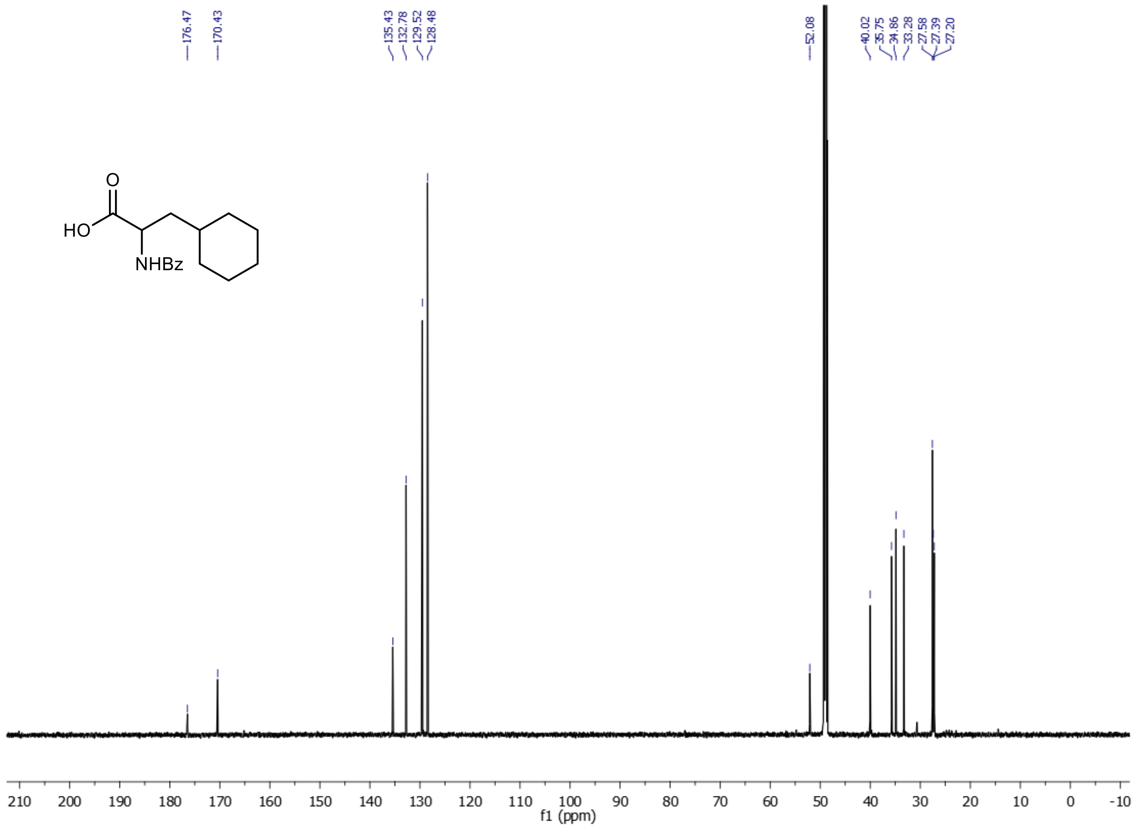

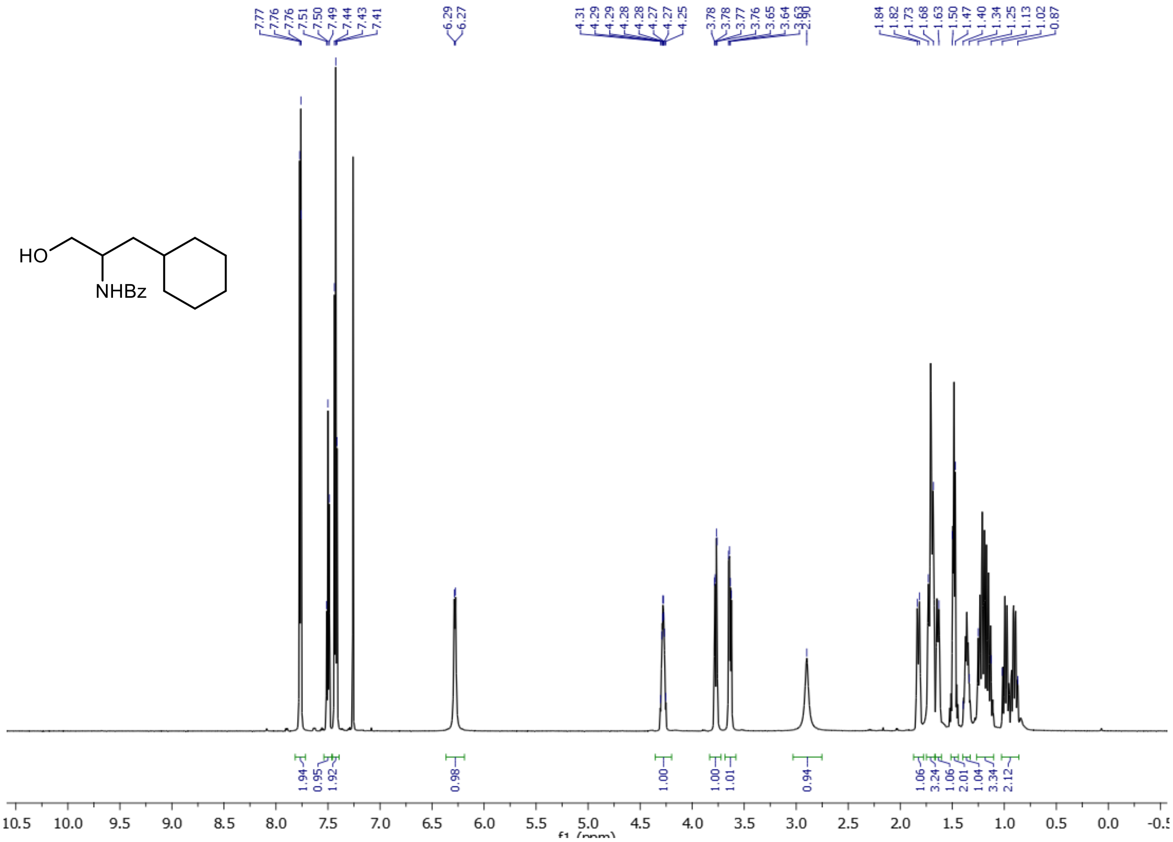
HO
NHBz
7.77 7.76 7.76 7.51 7.50 7.49 7.44 7.43 7.41
6.29 6.27
4.31 4.29 4.29 4.28 4.28 4.27 4.27 4.27 4.25
3.78 3.78 3.77 3.76 3.65 3.64 3.63 2.90
1.84 1.82 1.73 1.68 1.63 1.50 1.47 1.40 1.34 1.25 1.13 1.02 0.87
1.94 0.95 1.92 0.98 1.00 1.00 1.01 0.94 1.06 3.24 1.06 2.01 1.04 3.34 2.12
f1 (ppm)

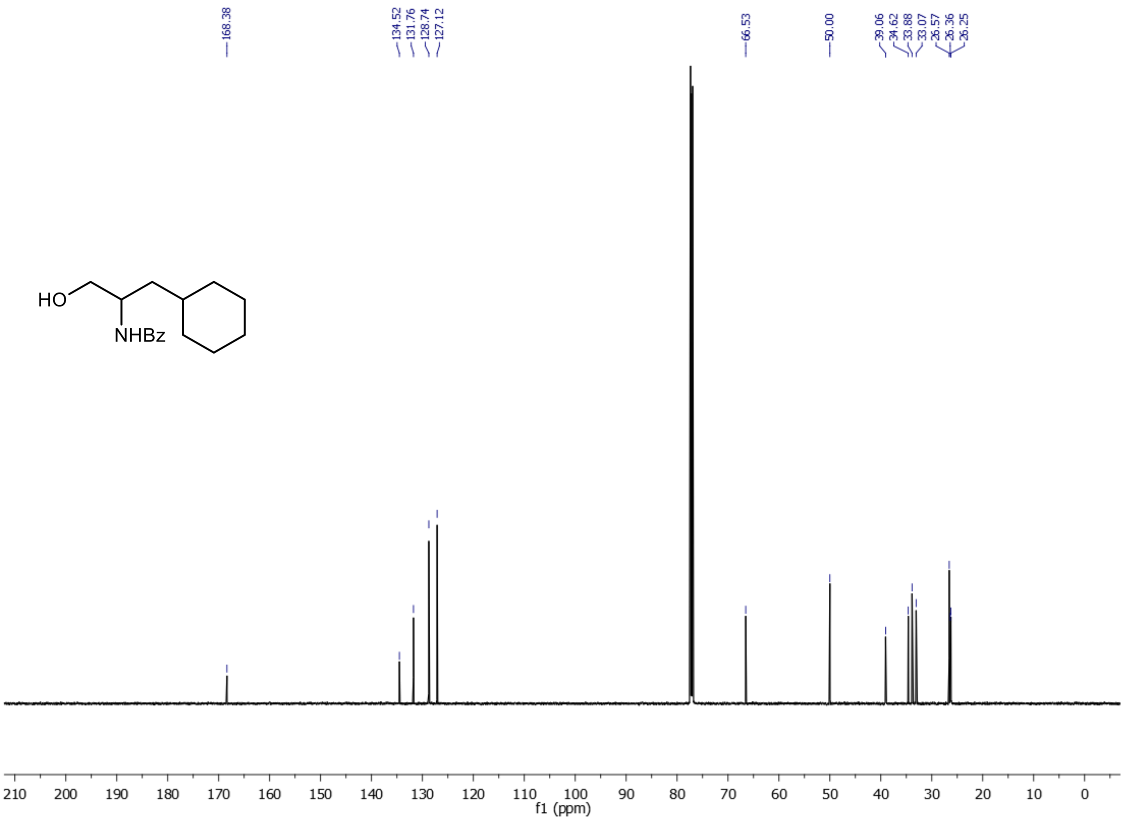
HO
NHBz
168.38
134.52 131.76 128.74 127.12
66.53
50.00
39.06 34.62 33.88 33.07 26.57 26.36 26.25
f1 (ppm)

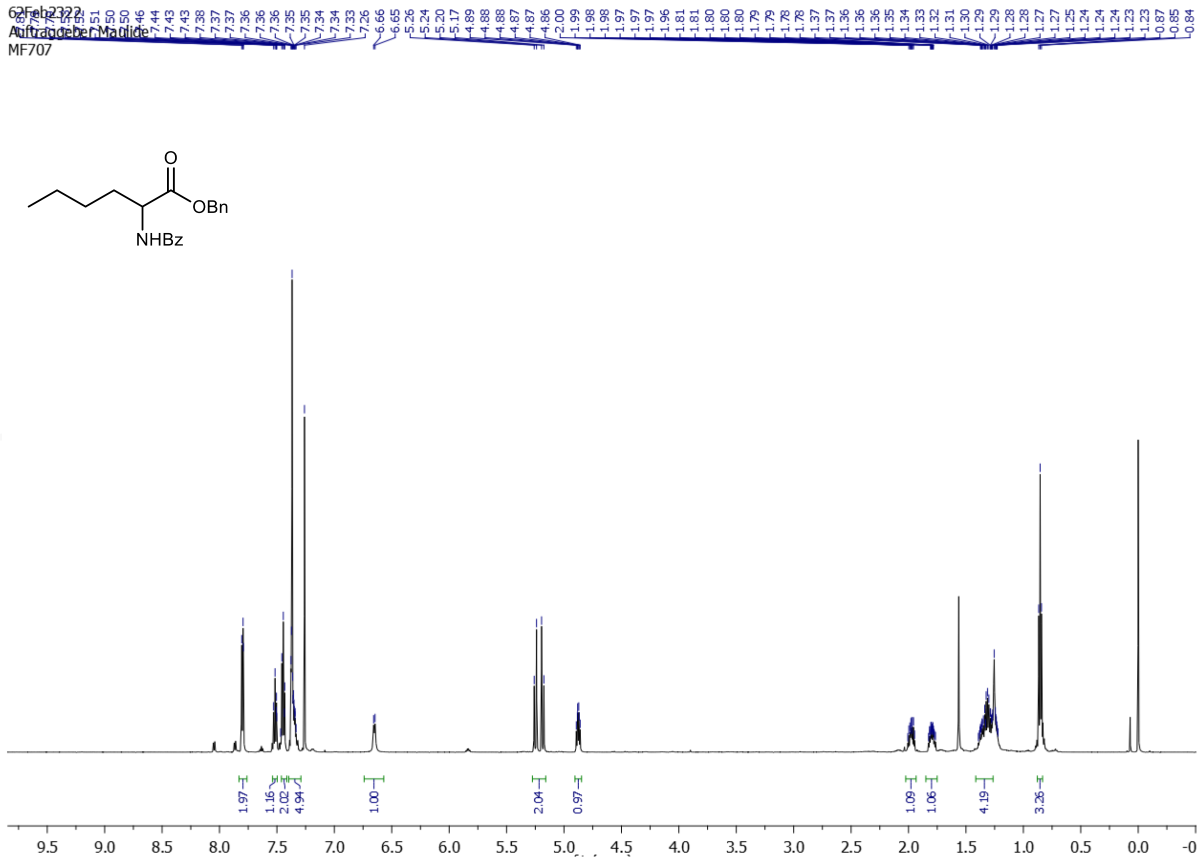
MF707
O
OBn
NHBz
1.97
1.16
2.02
4.94
1.00
2.04
0.97
1.09
1.06
4.19
3.26

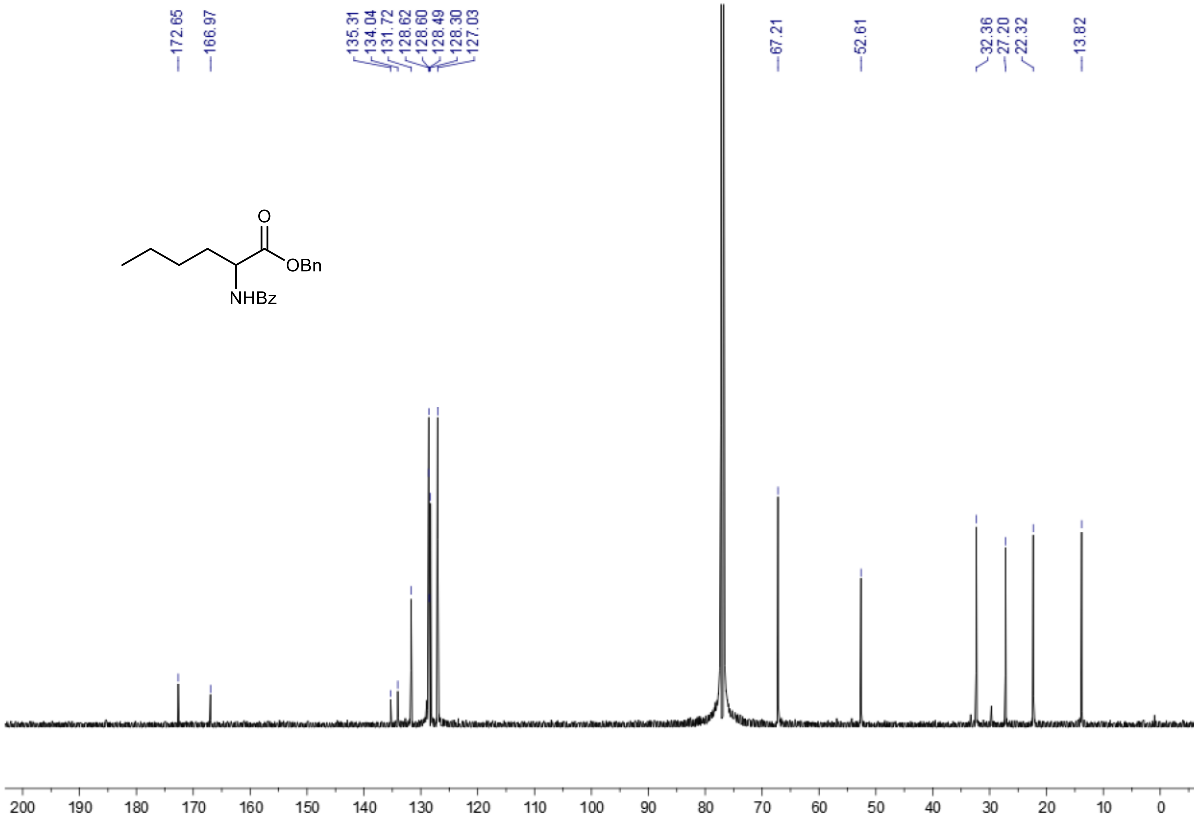
172.65
166.97
135.31
134.04
131.72
128.62
128.60
128.49
128.30
127.03
67.21
52.61
32.36
27.20
22.32
13.82
O
OBn
NHBz

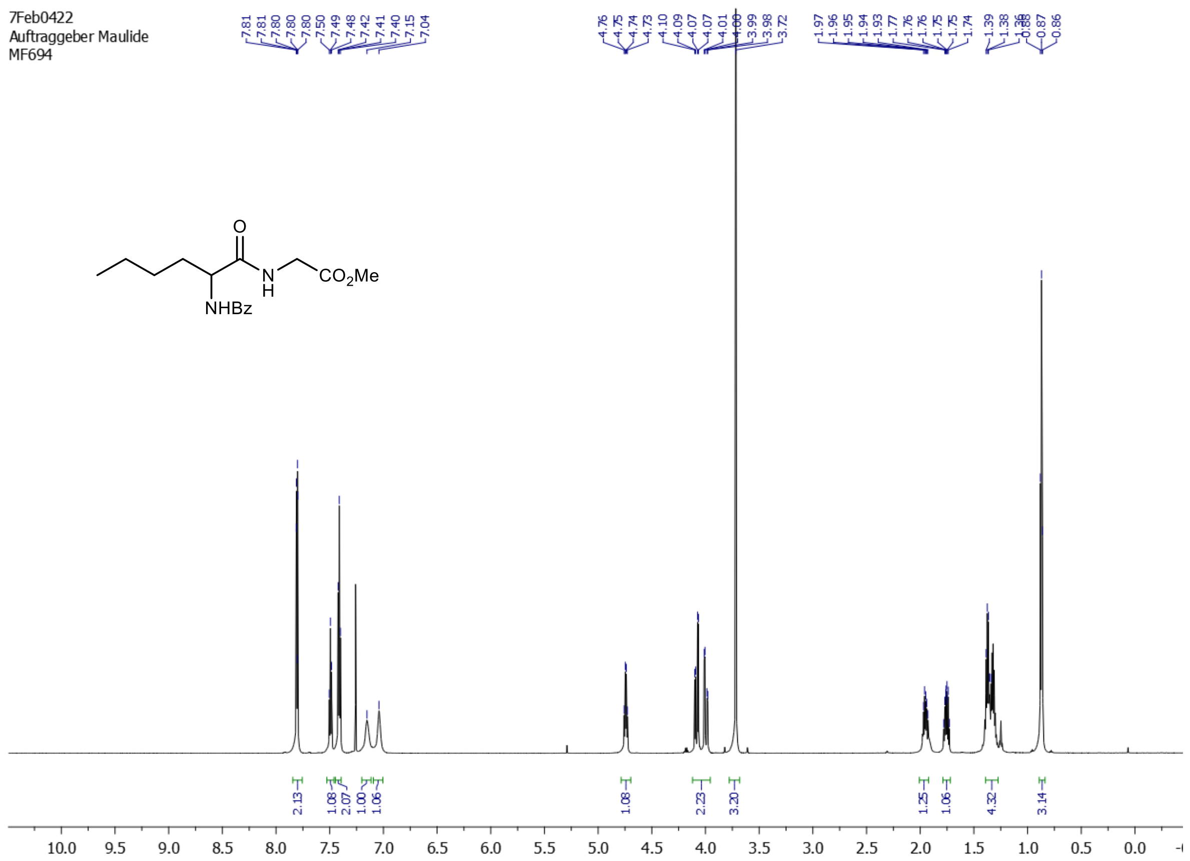
7Feb0422
Auftraggeber Maulide
MF694
7.81 7.81 7.80 7.80 7.80 7.50 7.49 7.48 7.42 7.41 7.40 7.15 7.04
4.76 4.75 4.74 4.73 4.10 4.09 4.07 4.07 4.01 4.00 3.99 3.98 3.72
1.97 1.96 1.95 1.94 1.93 1.77 1.76 1.76 1.75 1.75 1.74 1.39 1.38 1.36 0.88 0.87 0.86
O
N
H
CO2Me
NHBz
2.13 1.08 2.07 1.00 1.06 1.08 2.23 3.20 1.25 1.06 4.32 3.14
10.0 9.5 9.0 8.5 8.0 7.5 7.0 6.5 6.0 5.5 5.0 4.5 4.0 3.5 3.0 2.5 2.0 1.5 1.0 0.5 0.0

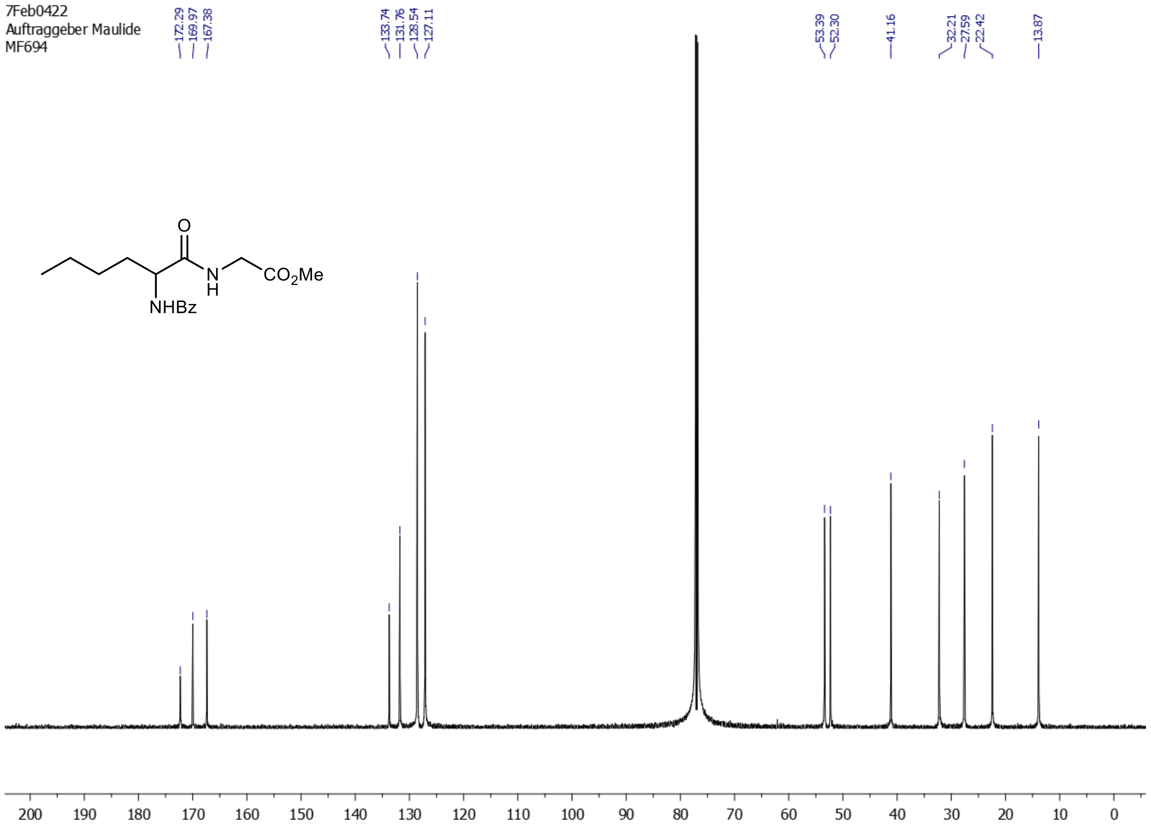
7Feb0422
Auftraggeber Maulide
MF694
172.29 169.97 167.38
133.74 131.76 128.54 127.11
53.39 52.30
41.16
32.21 27.59 22.42
13.87
O
N
H
CO2Me
NHBz
200 190 180 170 160 150 140 130 120 110 100 90 80 70 60 50 40 30 20 10 0

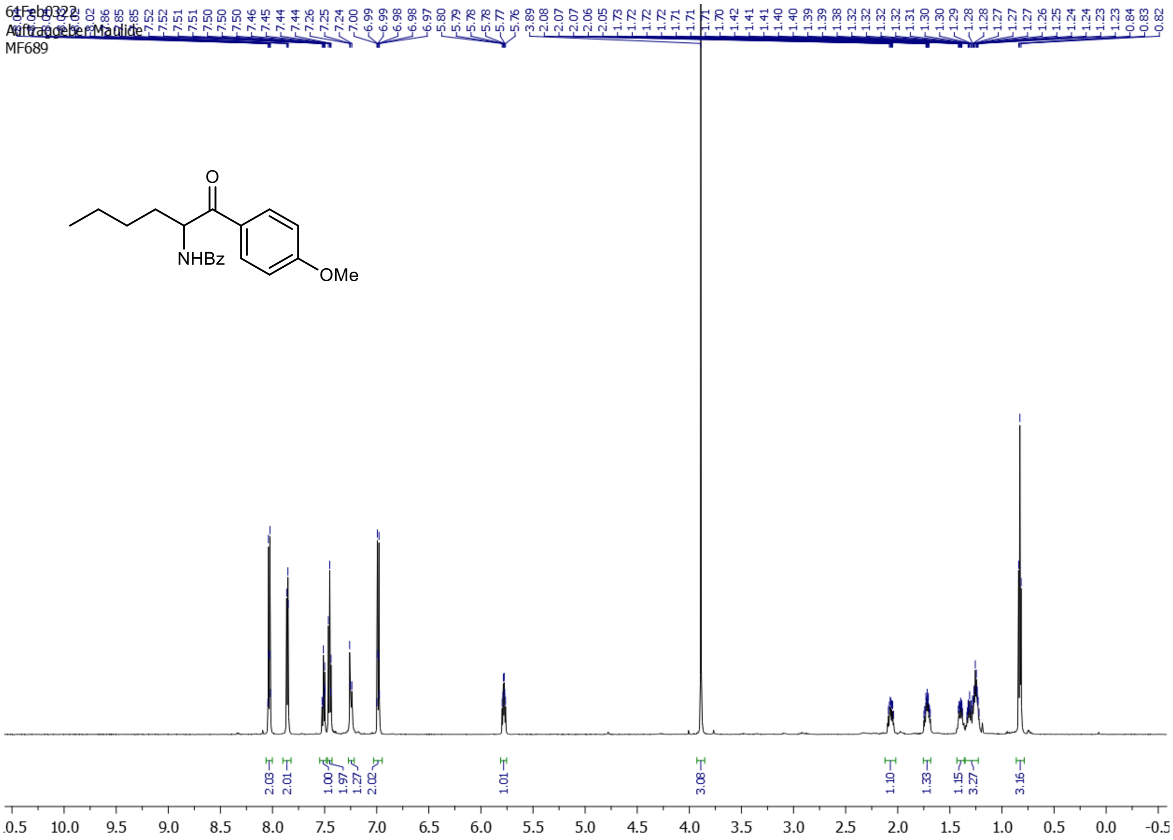
61Feb0322
Auftraggeber Maulide
MF689
NHBz
OMe
2.03
2.01
1.00
1.97
1.27
2.02
1.01
3.08
1.10
1.33
1.15
3.27
3.16

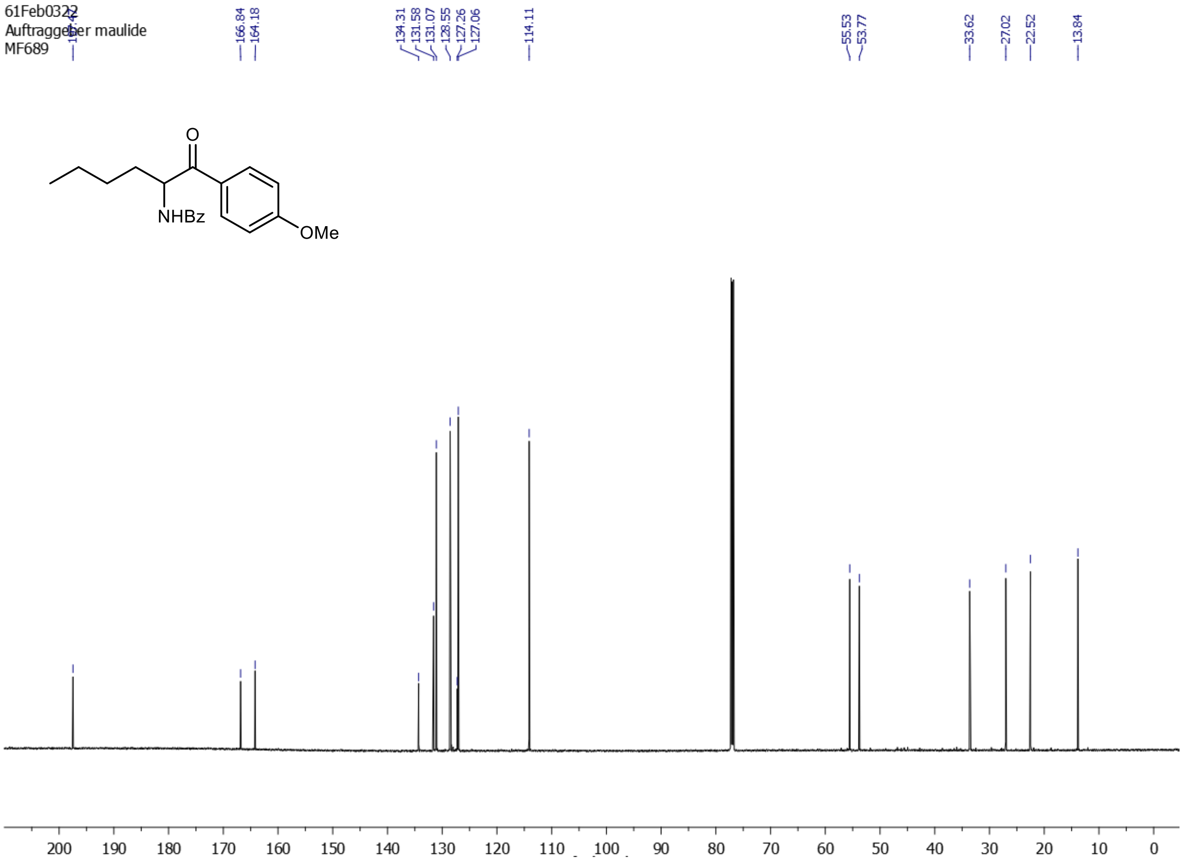
61Feb0322
Auftraggeber maulide
MF689
166.84
164.18
134.31
131.58
131.07
128.55
127.26
127.06
114.11
55.53
53.77
33.62
27.02
22.52
13.84
NHBz
OMe

10.References

(1) Starkov, P.; Moore, J. T.; Duquette, D. C.; Stoltz, B. M.; Marek, I. *J Am Chem Soc* **2017**, *139*, 9615–9620.

(2) Peng, B.; Huang, X.; Xie, L. G.; Maulide, N. *Angewandte Chemie - International Edition* **2014**, *53*, 8718–8721.

(3) Meng, S.; Wang, Y.; Liu, J.; Zheng, J.; Qian, X.; Wang, Q. *Organic Letters* **2022**, *24*, 757–761.

(4) Pinto, A.; Kaiser, D.; Maryasin, B.; di Mauro, G.; González, L.; Maulide, N. *Chemistry - A European Journal* **2018**, *24*, 2515–2519.

(5) Kaldre, D.; Klose, I.; Maulide, N. *Science* **2018**, *361*, 664–667.

(6) Xie, L. G.; Niyomchon, S.; Mota, A. J.; González, L.; Maulide, N. *Nature Communications* **2016**, *7*. https://doi.org/10.1038/ncomms10914.

(7) Singh, H.; Sahoo, T.; Sen, C.; Galani, S. M.; Ghosh, S. C. *Catalysis Science and Technology* **2019**, *9*, 1691–1698.

(8) Gao, Y.; Wu, G.; Zhou, Q.; Wang, J. *Angewandte Chemie - International Edition* **2018**, *57*, 2716–2720.

(9) Wen, H.; Cao, W.; Liu, Y.; Wang, L.; Chen, P.; Tang, Y. *Journal of Organic Chemistry* **2018**, *83*, 13308–13324.

(10) Baldassari, L. L.; de la Torre, A.; Li, J.; Lüdtke, D. S.; Maulide, N. *Angew. Chem. Int. Ed.* **2017**, *56*, 15723 –15727.

(11) Jouvin, K.; Heimburger, J.; Evano, G. *Chemical Science* **2012**, *3*, 756–760.

(12) Xie, L.-G.; Shaaban, S.; Chen, X.; Maulide, N. *Angew. Chem. Int. Ed.* **2016**, *55*, 12864–12867.

(13) Kaldre, D.; Maryasin, B.; Kaiser, D.; Gajsek, O.; González, L.; Maulide, N. *Angew. Chem. Int. Ed.* **2017**, *56*, 2212–2215.

(14) di Mauro, G.; Drescher, M.; Tkaczyk, S.; Maulide, N. *Synlett* **2020**, *31*, 592–594.

(15) Zhou, Y.; Qin, Y.; Wang, Q.; Zhang, Z.; Zhu, G. *Angew. Chem. Int. Ed.* **2022**, *61*, https://doi.org/10.1002/anie.202110864.

(16) Li, M.; Song, W.; Dong, K.; Zheng, Y. *Tetrahedron Letters* **2020**, *61*, 151503.

(17) Chen, Y.; Wen, S.; Tian, Q.; Zhang, Y.; Cheng, G. *Organic Letters* **2021**, *23*, 7905–7909.

(18) Pracht, P.; Bohle, F.; Grimme, S. *Phys. Chem. Chem. Phys.* **2020**, 22, 7169–7192

(19) Grimme, S. *J. Chem. Theory Comput.* **2019**, *15*, 2847–2862.

(20) Becke, D. *J. Chem. Phys.*, **1993**, *98*, 5648.

(21) Adamo, C.; Barone, V.; *J. Chem. Phys.* **1999**, *110*, 6158.

(22) Grimme, S.; Antony, J.; Ehrlich, S.; Krieg, H.; *J. Chem. Phys.* **2010**, *132*, 154104.

(23) Grimme, S.; Ehrlich, S.; Goerigk, L.; *J. Comput. Chem.* **2011**, *32*, 1456.

(24) Lee C, Yang W, Parr RG. *Phys. Rev. B: Condens. Matter Mater. Phys*. **1988**, 37, 785.

(25) Vosko SH, Wilk L, Nusair M. *Can. J. Phys*. **1980**, 58, 1200

(26) Stephens PJ, Devlin FJ, Chabalowski CF, Frisch MJ. *J. Phys. Chem*. **1994**, 98, 11623

(27) Weigend F, Ahlrichs R. *Phys. Chem. Chem. Phys*. **2005**, 7, 3297

(28) Pracht P, Bohle F, Grimme S. *Phys. Chem. Chem. Phys*. **2020**, 22, 7169

(29) Grimme S. *J. Chem. Theory Comput*. **2019**, 15, 2847

(30) Frisch, M. J.; Trucks, G. W.; *et al.* Gaussian 16, Revision C.01, Gaussian, Inc., Wallingford CT, **2019**

(31) Cancès, E.; Mennucci, B.; Tomasi, J.; *J. Chem. Phys.* **1997**, *107*, 3032.

(32) Marenich, V.; Cramer, C. J.; Truhlar, D. G. *J. Phys. Chem. B* **2009**, *113*, 6378.

(33) Bruker SAINT v8.38B Copyright © 2005-2019 Bruker AXS

(34) Sheldrick, G. M. (1996). *SADABS*. University of Göttingen, Germany.

(35) Dolomanov, O.V.; Bourhis, L.J.; Gildea, R.J.; Howard, J. A. K.; Puschmann, H.; *J. Appl. Cryst.* **2009**, *42*, 339-341.

(36) Huebschle, C. B.; Sheldrick, G. M.; Dittrich, B.; *J. Appl. Cryst.*, **2011**, *44*, 1281-1284.

(37) Sheldrick, G. M. (2015). *SHELXS v 2016/4* University of Göttingen, Germany.

(38) Spek, A. L. *Acta Cryst.* **2009**, *D65*, 148-155.
