# Supplementary material for: Direct Synthesis of α‐Amino Acid Derivatives by Hydrative Amination of Alkynes
Source: Angew Chem Int Ed Engl. 2022 Nov 29;62(1):e202212399. doi: 10.1002/anie.202212399 (PMC10098499; doi:10.1002/anie.202212399)
Supplement: Supplementary file 3 — Supporting Information [file ANIE-62-0-s002.html]

checkCIF/PLATON page 2


# checkCIF (basic structural check) running

---

  
*Checking for embedded fcf data in CIF ...*   
*Found embedded fcf data in CIF. Extracting fcf data from uploaded CIF, please wait* **.** **.** **.** **.** **.** 

# checkCIF/PLATON (basic structural check)

---

Structure factors have been supplied for datablock(s) mf806

THIS REPORT IS FOR GUIDANCE ONLY. IF USED AS PART OF A REVIEW PROCEDURE FOR PUBLICATION, IT SHOULD NOT REPLACE THE EXPERTISE OF AN EXPERIENCED CRYSTALLOGRAPHIC REFEREE.

```
No syntax errors found. CIF dictionary  
Please wait while processing ....  Interpreting this report
```

Structure factor report  
  

**Datablock: mf806**


---

|  |  |  |
| --- | --- | --- |
| Bond precision: | C-C = 0.0057 A | Wavelength=0.71073 |

|  |  |  |  |
| --- | --- | --- | --- |
| Cell: | a=9.8950(3) | b=13.6424(4) | c=10.8666(4) |
|  | alpha=90 | beta=99.994(3) | gamma=90 |
| Temperature: | 100 K |  |  |

|  |  |  |
| --- | --- | --- |
|  | Calculated | Reported |
| Volume | 1444.64(8) | 1444.64(8) |
| Space group | P 21 | P 1 21 1 |
| Hall group | P 2yb | P 2yb |
| Moiety formula | C14 H19 N O2 S | C14 H19 N O2 S |
| Sum formula | C14 H19 N O2 S | C14 H19 N O2 S |
| Mr | 265.36 | 265.36 |
| Dx,g cm-3 | 1.220 | 1.220 |
| Z | 4 | 4 |
| Mu (mm-1) | 0.219 | 0.219 |
| F000 | 568.0 | 568.0 |
| F000' | 568.72 |  |
| h,k,lmax | 14,19,15 | 13,19,15 |
| Nref | 9016[ 4681] | 7771 |
| Tmin,Tmax | 0.947,0.974 | 0.936,0.974 |
| Tmin' | 0.936 |  |

|  |  |
| --- | --- |
| Correction method= # Reported T Limits: Tmin=0.936 Tmax=0.974 AbsCorr = MULTI-SCAN |  |

|  |  |
| --- | --- |
| Data completeness= 1.66/0.86 | Theta(max)= 30.768 |

|  |  |
| --- | --- |
| R(reflections)= 0.0423( 5982) | wR2(reflections)= 0.1332( 7771) |
| |  |  | | --- | --- | | S = 1.178 | Npar= 334 | |

---

```
The following ALERTS were generated. Each ALERT has the format
       test-name_ALERT_alert-type_alert-level.
Click on the hyperlinks for more details of the test.


---

Alert level B
PLAT196_ALERT_1_B No TEMP record and _measurement_temperature .NE.        293 Degree


---

Alert level C
PLAT340_ALERT_3_C Low Bond Precision on  C-C Bonds ...............    0.00567 Ang.  
PLAT911_ALERT_3_C Missing FCF Refl Between Thmin & STh/L=    0.600          2 Report
PLAT915_ALERT_3_C No Flack x Check Done: Low Friedel Pair Coverage         76 %     
PLAT918_ALERT_3_C Reflection(s) with I(obs) much Smaller I(calc) .          1 Check 


---

Alert level G
PLAT007_ALERT_5_G Number of Unrefined Donor-H Atoms ..............          2 Report
PLAT066_ALERT_1_G Predicted and Reported Tmin&Tmax Range Identical          ? Check 
PLAT720_ALERT_4_G Number of Unusual/Non-Standard Labels ..........          2 Note  
PLAT791_ALERT_4_G Model has Chirality at C1A         (Sohnke SpGr)          S Verify
PLAT791_ALERT_4_G Model has Chirality at C1B         (Sohnke SpGr)          S Verify
PLAT912_ALERT_4_G Missing # of FCF Reflections Above STh/L=  0.600        171 Note  
PLAT933_ALERT_2_G Number of HKL-OMIT Records in Embedded .res File          8 Note  
PLAT978_ALERT_2_G Number C-C Bonds with Positive Residual Density.          1 Info  


---

   0 ALERT level A = Most likely a serious problem - resolve or explain
   1 ALERT level B = A potentially serious problem, consider carefully
   4 ALERT level C = Check. Ensure it is not caused by an omission or oversight
   8 ALERT level G = General information/check it is not something unexpected

   2 ALERT type 1 CIF construction/syntax error, inconsistent or missing data
   2 ALERT type 2 Indicator that the structure model may be wrong or deficient
   4 ALERT type 3 Indicator that the structure quality may be low
   4 ALERT type 4 Improvement, methodology, query or suggestion
   1 ALERT type 5 Informative message, check
```

---

---

It is advisable to attempt to resolve as many as possible of the alerts in all categories. Often the minor alerts point to easily fixed oversights, errors and omissions in your CIF or refinement strategy, so attention to these fine details can be worthwhile. In order to resolve some of the more serious problems it may be necessary to carry out additional measurements or structure refinements. However, the purpose of your study may justify the reported deviations and the more serious of these should normally be commented upon in the discussion or experimental section of a paper or in the "special\_details" fields of the CIF. checkCIF was carefully designed to identify outliers and unusual parameters, but every test has its limitations and alerts that are not important in a particular case may appear. Conversely, the absence of alerts does not guarantee there are no aspects of the results needing attention. It is up to the individual to critically assess their own results and, if necessary, seek expert advice. **Publication of your CIF in IUCr journals** A basic structural check has been run on your CIF. These basic checks will be run on all CIFs submitted for publication in IUCr journals (*Acta Crystallographica*, *Journal of Applied Crystallography*, *Journal of Synchrotron Radiation*); however, if you intend to submit to *Acta Crystallographica Section C* or *E* or *IUCrData*, you should make sure that full publication checks are run on the final version of your CIF prior to submission. **Publication of your CIF in other journals** Please refer to the *Notes for Authors* of the relevant journal for any special instructions relating to CIF submission. |

---

**PLATON version of 12/09/2022; check.def file version of 09/08/2022**

|  |
| --- |
| **Datablock mf806** - ellipsoid plot |
|  |

---

 Download CIF editor (publCIF) from the IUCr   
 Download CIF editor (enCIFer) from the CCDC   
 Test a new CIF entry 
