# Supplementary material for: Direct Synthesis of α‐Amino Acid Derivatives by Hydrative Amination of Alkynes
Source: Angew Chem Int Ed Engl. 2022 Nov 29;62(1):e202212399. doi: 10.1002/anie.202212399 (PMC10098499; doi:10.1002/anie.202212399)
Supplement: Supplementary file 5 — Supporting Information [file ANIE-62-0-s005.html]

checkCIF/PLATON report


```
No syntax errors found.                               CIF dictionary  
Please wait while processing ....                     Interpreting this report
```

**Datablock: mf\_198\_c2c**


---

|  |  |  |
| --- | --- | --- |
| Bond precision: | C-C = 0.0084 A | Wavelength=0.71073 |

|  |  |  |  |
| --- | --- | --- | --- |
| Cell: | a=22.674(7) | b=5.1412(12) | c=25.909(9) |
|  | alpha=90 | beta=97.083(13) | gamma=90 |
| Temperature: | 100 K |  |  |

|  |  |  |
| --- | --- | --- |
|  | Calculated | Reported |
| Volume | 2997.2(16) | 2997.2(15) |
| Space group | C 2/c | C 2/c |
| Hall group | -C 2yc | -C 2yc |
| Moiety formula | C16 H20 N2 O4 | C16 H20 N2 O4 |
| Sum formula | C16 H20 N2 O4 | C16 H20 N2 O4 |
| Mr | 304.34 | 304.34 |
| Dx,g cm-3 | 1.349 | 1.349 |
| Z | 8 | 8 |
| Mu (mm-1) | 0.098 | 0.098 |
| F000 | 1296.0 | 1296.0 |
| F000' | 1296.64 |  |
| h,k,lmax | 31,7,36 | 31,6,34 |
| Nref | 4377 | 4036 |
| Tmin,Tmax | 0.996,0.999 | 0.340,0.648 |
| Tmin' | 0.990 |  |

|  |  |
| --- | --- |
| Correction method= # Reported T Limits: Tmin=0.340 Tmax=0.648 AbsCorr = MULTI-SCAN |  |

|  |  |
| --- | --- |
| Data completeness= 0.922 | Theta(max)= 30.015 |

|  |  |
| --- | --- |
| R(reflections)= 0.1281( 1422) | wR2(reflections)= 0.3941( 4036) |
| |  |  | | --- | --- | | S = 0.997 | Npar= 200 | |

---

```
The following ALERTS were generated. Each ALERT has the format
       test-name_ALERT_alert-type_alert-level.
Click on the hyperlinks for more details of the test.


---

Alert level B
PLAT026_ALERT_3_B Ratio Observed / Unique Reflections (too) Low ..        35% Check 
PLAT084_ALERT_3_B High wR2 Value (i.e. > 0.25) ...................       0.39 Report


---

Alert level C
RINTA01_ALERT_3_C  The value of Rint is greater than 0.12
            Rint given   0.169
PLAT020_ALERT_3_C The Value of Rint is Greater Than 0.12 .........      0.169 Report
PLAT082_ALERT_2_C High R1 Value ..................................       0.13 Report
PLAT250_ALERT_2_C Large U3/U1 Ratio for Average U(i,j) Tensor ....        2.1 Note  
PLAT340_ALERT_3_C Low Bond Precision on  C-C Bonds ...............    0.00838 Ang.  
PLAT906_ALERT_3_C Large K Value in the Analysis of Variance ......     60.064 Check

And 5 other PLAT906 Alerts

PLAT906_ALERT_3_C Large K Value in the Analysis of Variance ......      2.856 Check 
PLAT906_ALERT_3_C Large K Value in the Analysis of Variance ......      7.376 Check 
PLAT906_ALERT_3_C Large K Value in the Analysis of Variance ......      2.294 Check 
PLAT906_ALERT_3_C Large K Value in the Analysis of Variance ......      3.920 Check 
PLAT906_ALERT_3_C Large K Value in the Analysis of Variance ......      2.605 Check

PLAT911_ALERT_3_C Missing FCF Refl Between Thmin & STh/L=    0.600         15 Report


---

Alert level G
PLAT007_ALERT_5_G Number of Unrefined Donor-H Atoms ..............          1 Report
PLAT072_ALERT_2_G SHELXL First  Parameter in WGHT  Unusually Large       0.16 Report
PLAT398_ALERT_2_G Deviating  C-O-C   Angle From 120 for O3       .      108.6 Degree
PLAT793_ALERT_4_G Model has Chirality at C1          (Centro SPGR)          S Verify
PLAT910_ALERT_3_G Missing # of FCF Reflection(s) Below Theta(Min).          3 Note  
PLAT912_ALERT_4_G Missing # of FCF Reflections Above STh/L=  0.600        250 Note  
PLAT941_ALERT_3_G Average HKL Measurement Multiplicity ...........        2.6 Low   
PLAT952_ALERT_5_G Calculated (ThMax) and CIF-Reported Lmax Differ.          2 Units 
PLAT958_ALERT_1_G Calculated (ThMax) and Actual (FCF) Lmax Differ.          2 Units 
PLAT978_ALERT_2_G Number C-C Bonds with Positive Residual Density.          0 Info  


---

   0 ALERT level A = Most likely a serious problem - resolve or explain
   2 ALERT level B = A potentially serious problem, consider carefully
  12 ALERT level C = Check. Ensure it is not caused by an omission or oversight
  10 ALERT level G = General information/check it is not something unexpected

   1 ALERT type 1 CIF construction/syntax error, inconsistent or missing data
   5 ALERT type 2 Indicator that the structure model may be wrong or deficient
  14 ALERT type 3 Indicator that the structure quality may be low
   2 ALERT type 4 Improvement, methodology, query or suggestion
   2 ALERT type 5 Informative message, check
```

---

---

It is advisable to attempt to resolve as many as possible of the alerts in all categories. Often the minor alerts point to easily fixed oversights, errors and omissions in your CIF or refinement strategy, so attention to these fine details can be worthwhile. In order to resolve some of the more serious problems it may be necessary to carry out additional measurements or structure refinements. However, the purpose of your study may justify the reported deviations and the more serious of these should normally be commented upon in the discussion or experimental section of a paper or in the "special\_details" fields of the CIF. checkCIF was carefully designed to identify outliers and unusual parameters, but every test has its limitations and alerts that are not important in a particular case may appear. Conversely, the absence of alerts does not guarantee there are no aspects of the results needing attention. It is up to the individual to critically assess their own results and, if necessary, seek expert advice. **Publication of your CIF in IUCr journals** A basic structural check has been run on your CIF. These basic checks will be run on all CIFs submitted for publication in IUCr journals (*Acta Crystallographica*, *Journal of Applied Crystallography*, *Journal of Synchrotron Radiation*); however, if you intend to submit to *Acta Crystallographica Section C* or *E* or *IUCrData*, you should make sure that full publication checks are run on the final version of your CIF prior to submission. **Publication of your CIF in other journals** Please refer to the *Notes for Authors* of the relevant journal for any special instructions relating to CIF submission. |

---

**PLATON version of 19/02/2022; check.def file version of 19/02/2022**

|  |
| --- |
| **Datablock mf\_198\_c2c** - ellipsoid plot |
|  |

---

 Download CIF editor (publCIF) from the IUCr   
 Download CIF editor (enCIFer) from the CCDC   
 Test a new CIF entry 
